# THC exposure of human iPSC neurons impacts genes associated with neuropsychiatric disorders

**DOI:** 10.1038/s41398-018-0137-3

**Published:** 2018-04-25

**Authors:** Boris Guennewig, Maina Bitar, Ifeanyi Obiorah, James Hanks, Elizabeth A. O’Brien, Dominik C. Kaczorowski, Yasmin L. Hurd, Panos Roussos, Kristen J. Brennand, Guy Barry

**Affiliations:** 1 Sydney Medical School, Brain and Mind Centre, The University of Sydney, Camperdown, Sydney, NSW Australia; 2 St. Vincent’s Clinical School and School of Biotechnology and Biomolecular Sciences, University of New South Wales, Kensington, NSW Australia; 3 Garvan Institute of Medical Research, Darlinghurst, NSW Australia; 4 QIMR Berghofer Medical Research Institute, Herston, QLD Australia; 5 Department of Psychiatry, Icahn School of Medicine at Mount Sinai, New York, NY USA; 6 Department of Neuroscience and Friedman Brain Institute, New York, NY USA; 7 Department of Genetics and Genomic Science and Institute for Multiscale Biology, New York, NY USA; 8 Mental Illness Research, Education, and Clinical Center (VISN 2), James J. Peters VA Medical Center, Bronx, NY USA; 9 Fishberg Department of Neuroscience, Icahn School of Medicine at Mount Sinai, New York, NY USA

## Abstract

There is a strong association between cannabis use and schizophrenia but the underlying cellular links are poorly understood. Neurons derived from human-induced pluripotent stem cells (hiPSCs) offer a platform for investigating both baseline and dynamic changes in human neural cells. Here, we exposed neurons derived from hiPSCs to Δ^9^-tetrahydrocannabinol (THC), and identified diagnosis-specific differences not detectable in vehicle-controls. RNA transcriptomic analyses revealed that THC administration, either by acute or chronic exposure, dampened the neuronal transcriptional response following potassium chloride (KCl)-induced neuronal depolarization. THC-treated neurons displayed significant synaptic, mitochondrial, and glutamate signaling alterations that may underlie their failure to activate appropriately; this blunted response resembles effects previously observed in schizophrenia hiPSC- derived neurons. Furthermore, we show a significant alteration in THC-related genes associated with autism and intellectual disability, suggesting shared molecular pathways perturbed in neuropsychiatric disorders that are exacerbated by THC.

## Introduction

Human-induced pluripotent stem cells (hiPSCs) serve as a tool for the study of developmental processes and disease-relevant models. This has been especially valuable for the study of the human brain where primary tissue for study has been the most difficult to obtain. hiPSCs have provided mechanistic insights into both neurodevelopmental disorders^1^ and neurodegenerative diseases^2,3^. Research into psychiatric disorders such as autism^4^, bipolar disease^5^, and schizophrenia^6^ have greatly benefited from the insights afforded by hiPSCs, as these are largely considered human- specific disorders. hiPSC-based models facilitate isogenic investigations into molecular and environmental factors that may exacerbate or ameliorate disease predisposition.

The widespread use of cannabis calls for a concerted effort into increased understanding of both the positive and negative effects of the drug. Brain imaging studies of the primary psychoactive component of cannabis, Δ^9^-tetrahydrocannabinol (THC), demonstrated structural and functional changes following regular cannabis use^7^, while molecular studies uncovered signaling pathways downstream of the two cannabinoid (CB) receptors, CB1, and CB2. Depression of glutamate signaling is a common feature of THC-induced effects via the CB1 receptor in both humans and in animal models^8,9^.

There is a significant association between cannabis use and schizophrenia in human subjects^10–14^, however, whether this reflects patient self-medication of prodromal symptoms or an environmental modulation of genetic susceptibility remains an ongoing discussion^15,16^. We recently reported molecular abnormalities in schizophrenia patient hiPSC-derived neurons in response to neural activity^6^; here we describe a distinct overlap in hypo-excitability, particularly in the glutamate system, between schizophrenia patient-derived neurons and those treated with THC. THC exposure seems to deregulate glutamate receptors and other genes involved in synaptic function. We observe significant THC-dependent changes in postsynaptic density, ion channel and WNT pathway genes, and epigenetic regulators; and molecular connections to autism and intellectual disability. Although the molecular mechanisms may not be precisely the same, the convergence of glutamatergic hypo-function may partially explain the increased risk for psychiatric disorders amongst those exposed to cannabis.

## Materials and methods

### Generation of hiPSC neurons and RNA sequencing

Human fibroblasts were obtained from ATCC (CRL-2522) and Coriell (control: GM03440, GM03651, GM04506, AG09319, AG09429; SZ: GM01792, GM02038, GM01835, and GM02497). Limited phenotypic information for each donor is available from the Coriell Cell Repository, and summarized in the methods of Topol et al^17^. Unfortunately, THC exposure status for each donor is unknown. hiPSCs were reprogrammed using tetracycline-inducible lentiviral vectors and differentiated to neural precursor cells (NPCs) as previously described^18^. NPCs were differentiated on poly- ornithine/laminin coated plates for 6 weeks. Passage-matched NPCs were used for all experiments. All hiPSC and NPCs used were mycoplasma-free. Forebrain neural progenitor cells were generated from five control and four case hiPSCs as previously reported^6,18,19^ and neurons were differentiated according to a 6-week maturation protocol. Samples used in RNA sequencing or quantitative RT–PCR can be found in Supplementary Table S1. THC was dissolved in DMSO to 1 mg/ml and prepared as previously described;^20^ in all experiments, an equivalent volume of DMSO was used as a vehicle control. Acute (1 μM THC for 24 h) and chronic (50 nM THC; five treatments over 7 days) THC exposure (and DMSO-vehicle control) occurred immediately prior to collection. KCl was dissolved in PBS as previously described^6^; in all experiments, an equivalent volume of PBS was used as a vehicle control. 50 mM KCl treatment occurred for the final three hours prior to collection; consistent with our previous molecular^6^ and neurotransmitter release^21^ studies. For RNA-seq experiments, two wells per individual were treated. The RNA Integrity Number (RIN) was determined using an RNA Nano chip (Agilent Technologies) on the Agilent 2100 Bioanalyzer. All samples have high RIN (mean ± SD: 9.54 ± 0.21). 500 ng of total RNA was used as input material for library preparation using the TruSeq Stranded Total RNA Kit (Illumina, USA).

### Processing of RNA sequencing data and analyses

RNA sequencing data has been deposited into Sequence Read Archive (SRA; PRNJA419702, “RNA-Seq of iPSC-derived neurons”). Reads were mapped to GRCh38.p5 reference genome using STAR (version 2.5.1a). Known Gencode gene levels (version 24) were quantified by RSEM (version 1.3.0). To facilitate inter-dataset comparisons, we performed ranked (Spearman) and unranked correlation (Pearson) analysis of the controls in both the ±KCl and ±THC datasets, and confirmed that the samples are highly comparable (all control comparisons are ≥ 97%). Differentially expressed genes were identified with edgeR in R after TMM normalization and filtering. *p*-values and false discovery rate (FDR) were calculated and differentially expressed genes (DEG) were determined as those with an estimated *p*-value ≤ 0.05 and FDR ≤ 0.01.

### Gene sets for enrichment analyses

To further characterize the DEGs we performed enrichment analysis, using a group of gene sets for known molecular pathways and biological processes, including: Gene Ontology (GO) sets of molecular functions (MF), biological processes (BP), and cellular components (CC) (http://www.geneontology.org); the KEGG dataset (http://www.genome.jp/kegg/pathway.html); and the HUGO Gene Nomenclature Committee (HGNC) gene families (http://www.genenames.org). The genes in each gene set were tested for overlap using Fisher’s exact test and FDR correction. Differential expressed genes were (i) separated in upregulated and downregulated genes; (ii) analyzed for full GO overrepresentation according to hypergeometric testing with a significance cutoff FDR = 0.05 in BiNGO (version 3.0.3); (iii) processed with the enrichment map pipeline (https://f1000research.com/posters/5-1235) a *p*-value cutoff = 0.001, *q*-value cutoff = 0.05 and Jaccard coefficient cutoff = 0.25 and (iv) visualized in Cytoscape (version 3.5.1).

### Quantitative RT–PCR

For qPCR experiments, three wells per individual were treated with either DMSO- vehicle control for 7 days, acute THC exposure (1 μM THC for 24 h) or chronic THC treatment (five treatments with 50 nM THC over 7 days) immediately prior to collection at 6 weeks. Candidate genes were validated for THC-treated and activity-treated alterations using quantitative RT–PCR. cDNA synthesis was performed using the SuperScript III First-Strand Synthesis System (ThermoFisher Scientific, USA). Briefly, 500 ng of total RNA was used and random hexamer primed protocol was followed. Each cDNA sample was amplified in triplicate using SYBR Green PCR Master Mix (ThermoFisher Scientific, USA). Primer pairs used for this analysis are described in Supplementary Table S2.

### Generation of gene datasets

As no up to date datasets for associated genes were available for autism, intellectual disability or schizophrenia, we generated our own through extensive literature and database searches. Specific details are available in the Supplemental Information ‘Generation of Gene Databases’.

## Results

### hiPSC-derived neurons as a model for THC biology

**Fig. 1:**
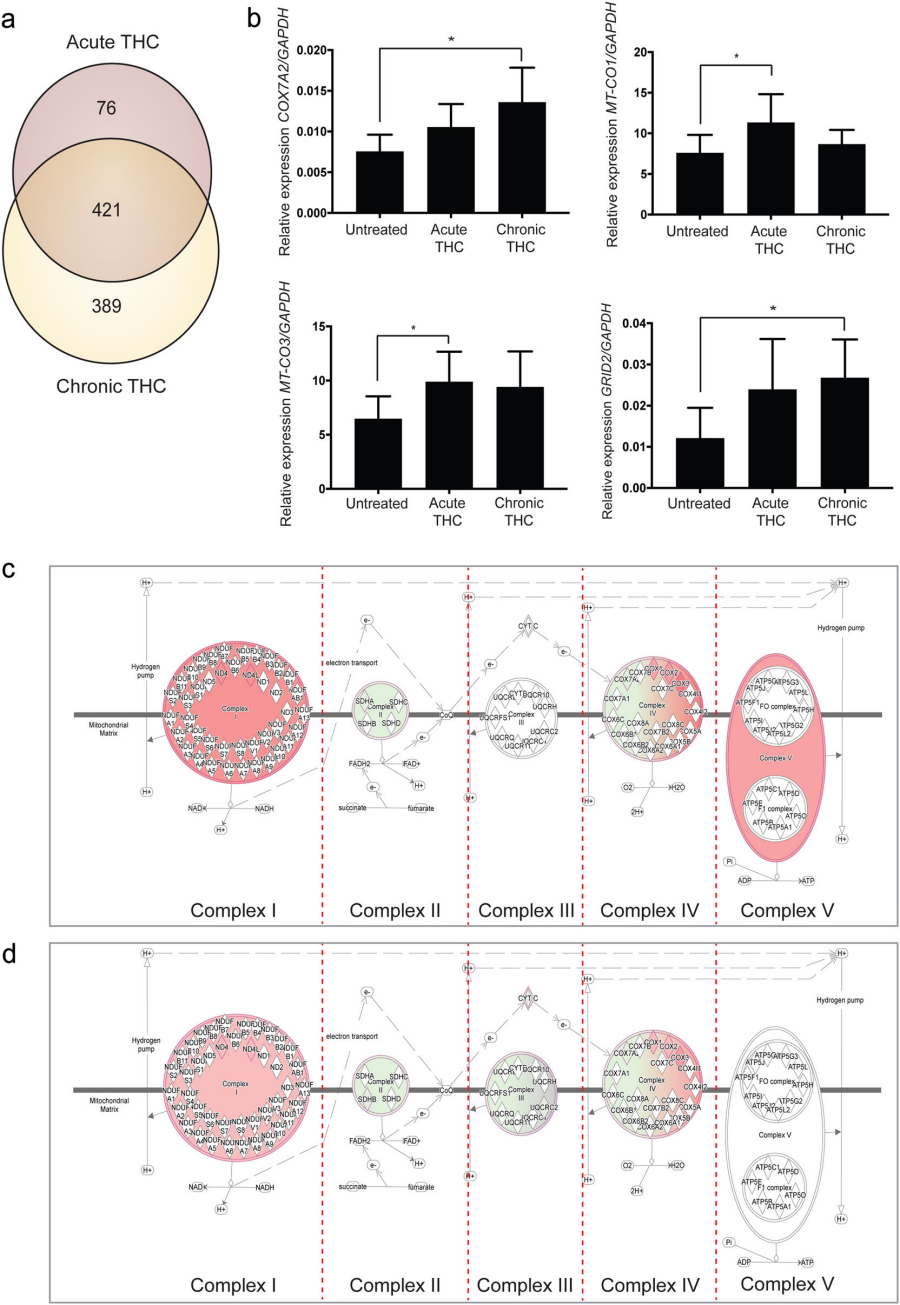
THC treatment regulates genes involved in mitochondrial and glutamate pathways. **a** RNA sequencing of hiPSC-derived neurons reveals 497 genes (acute) and 810 genes (chronic) are significantly changed following THC exposure, including. **b** genes involved in mitochondrial (e.g., *COX7A2*, *MT-CO1*, and *MT-CO3*) and glutamate (e.g., *GRID2*) pathways (Quantitative RT–PCR (qRT–PCR); Ordinary one-way ANOVA with Tukey’s multiple comparisons test: *p < 0.05. *n* = 5 (see qRT–PCR, Ca–Ce, Supplementary Table S1)). Ingenuity pathway analysis shows that mitochondrial oxidative phosphorylation is strongly altered after both acute **c** and chronic **d** THC exposure

To gain further insight into THC-related molecular mechanisms we utilized hiPSC-derived neurons from four controls as previously reported^6^. THC (or vehicle control) was added to hiPSC-derived neurons from each individual as acute (1 μM THC for 24 h) or chronic (50 nM THC; five treatments over 7 days) treatments. Acute and chronic THC concentrations were rationally selected from studies of primary mouse neurons^22^ and experimentally validated in hiPSC neurons^23^. RNA was extracted and subjected to RNA sequencing (RNA-seq) using the Illumina platform. Our bioinformatic analyses pipeline combined integrated genome/transcriptome alignment using STAR, quantification using RSEM and differential expression using EdgeR. Relative to vehicle treatment, acute THC exposure resulted in 497 genes significantly altered in hiPSC- derived neurons compared to untreated controls, while chronic THC exposure perturbed 810 genes (Fig. 1a; Supplementary Table S3; Supplementary Figure 1). The overlap between acute and chronic exposures was highly significant (421 genes; *p*- value = 0e + 00, odds ratio = 586.5, Fisher’s exact test). Specific subsets of genes involved in the glutamate receptor pathway and mitochondrial function were altered in response to acute or chronic THC exposure (Supplementary Table S4; Supplementary Figure 2; Fig. 1b–d) and have previously been implicated in THC biology^8,24,25^. These results provide data to support the use of hiPSC-derived neurons as a model for investigating THC responses in an in vitro human neuronal system.

### RNA sequencing implicates synaptic function, demethylation and ion channel function in THC-treated hiPSC neurons

**Fig. 2:**
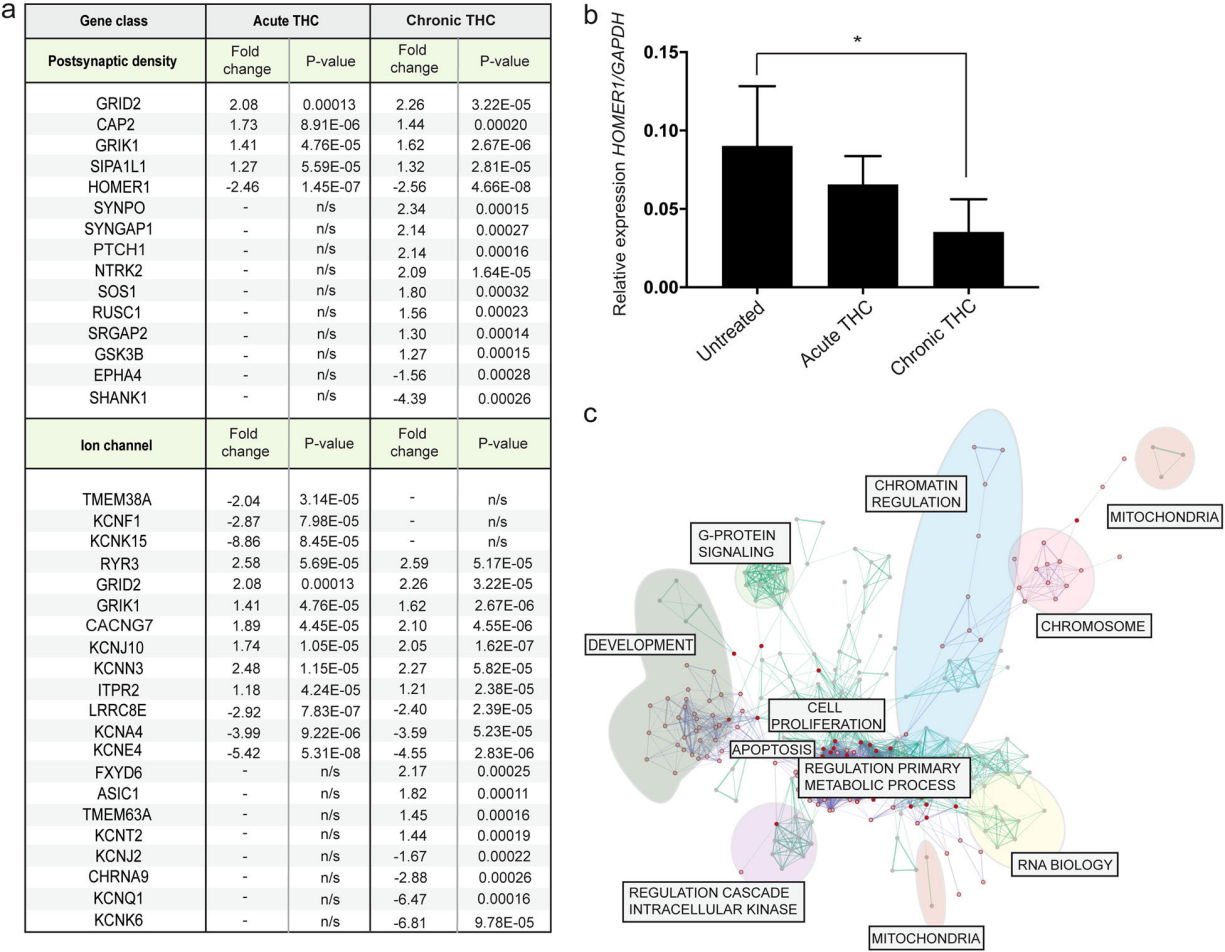
Postsynaptic density and ion channel genes are regulated by THC treatment. **a**, **b** Multiple postsynaptic density and ion channel genes are significantly altered in hiPSC-derived neurons following acute or chronic THC exposure, including the postsynaptic gene *HOMER1* (Quantitative RT–PCR (qRT–PCR); Ordinary one-way ANOVA with Tukey’s multiple comparisons test: *p < 0.05. *n* = 5 (see qRT–PCR, Ca–Ce, Supplementary Table S1)). **c** Network analysis combining all THC-related genes from acute and chronic THC treatment shows broad changes to fundamental cellular functions such as RNA biology, chromatin regulation and development

Closer inspection of functional gene clusters associated with THC treatment revealed the potential contribution of genes involved at the postsynaptic density such as *HOMER1, GRID2, GRIK1*, and *SIPA1L1* (Fig. 2a, b). Moreover, chronic THC treatments resulted in the alteration of additional synaptic related genes such as *SYNGAP1* and *SHANK1*. Ion channel genes, especially potassium voltage-gated channel genes (*KCNE4, KCNA4*, *KCNJ10*, and *KCNN3*) are also responsive to both THC treatments with further ion channel genes (*KCNJ2*, *KCNA2*, and *KCNT2*) associated following chronic THC exposure (Fig. 2a). These results strongly implicate synaptic function as a key target of THC-mediated responses. Interestingly, we found epigenetic related transcriptional responses evident in both acute and chronic THC exposures that included the alterations of genes involved in the dynamic methylation/demethylation process (*DNMT1*, *GADD45B*, and *APOBEC3C*); chronic THC exposure resulted in further decreases of histone modification-related proteins such as *SETD1A, SETD5*, *CBX6, KMT2A*, *KMT2C*, and *NCOA6* and methyl binding proteins *MECP2* and *MBD5* (Supplementary Table S5). Network analyses (Enrichment map pipeline in Cytoscape) using the genes altered in response to THC exposure reinforce the involvement of pathways linked to developmental, chromatin regulation and mitochondrial biology (Fig. 2c; Supplementary Table S6).

### THC exposure significantly alters genes implicated in autism and intellectual disability

**Fig. 3:**
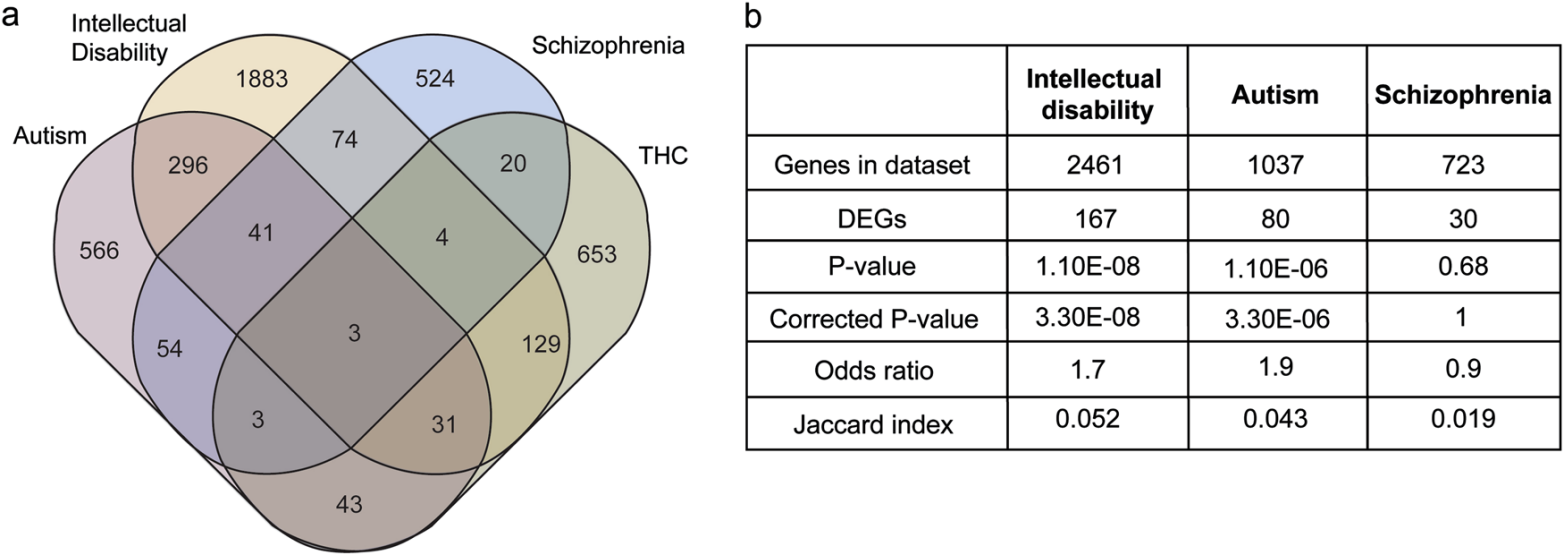
Genes altered by THC treatment in hiPSC-derived neurons are significantly associated with autism and intellectual disability. **a** Venn diagram showing the overlap between THC-related genes and autism, intellectual disability and schizophrenia. **b** THC-related genes are significantly related to autism and intellectual disability (*p*-value < 0.05)

We noticed that many genes implicated in psychiatric disease coincided with genes altered in response to THC treatments. In order to calculate statistical relevance we needed to first update the numbers of genes associated with these disorders and found genes related to autism spectrum disorder (1037 genes), intellectual disability (2461 genes) and schizophrenia (723 genes; see Supplementary Information ‘Generation of Gene Databases’ for details; Supplementary Table S7). Included in our list of significantly altered transcripts following THC exposure is a substantial number of genes linked to autism (80 genes) and intellectual disability (167 genes), with fewer overlapping with schizophrenia (Fig. 3a); autism and intellectual disability associated genes are significant for both *p*-value and odds ratio using the Fisher’s exact test (Fig. 3b). These data suggest that endogenous THC responsive pathways include many psychiatric disease-associated genes and that changes in these genes, either genetically or epigenetically, may contribute to cannabis-related adverse reactions such as psychosis in some users.

### Overlapping signaling pathways between THC and schizophrenia

We compared the bioinformatic results from our current THC studies to data obtained from our previous studies of schizophrenia patient hiPSC-derived neurons^6,18^ to ensure that the quality of the differentiations were comparable across experiments (Supplementary Figure 3). Raw data from all subjects from Roussos et al^6^. was applied to our bioinformatic pipeline. WNT and mitochondrial pathways (Supplementary Table S8) were significantly altered in both our current THC and previous schizophrenia studies^18,26^. Genes related to ion channel function were also highly represented in both the THC and schizophrenia gene lists (Supplementary Table S8). Although these pathways were conserved, specific genes related to altered ion channel, WNT or mitochondrial function did not overlap.

### Blunted activity-dependent transcriptional response shared between THC and schizophrenia

**Fig. 4:**
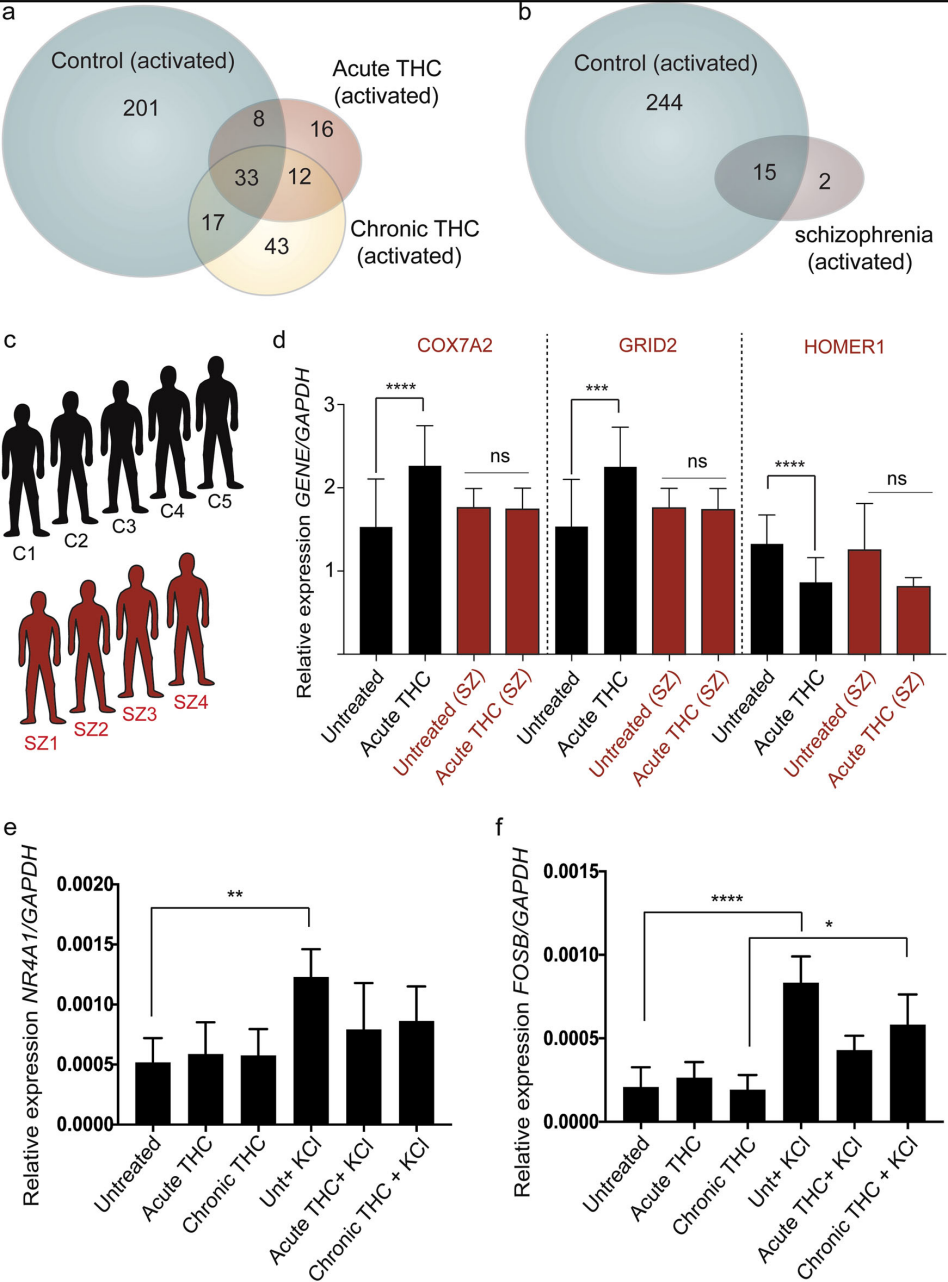
THC treatment results in neuronal hypo-excitability similar to observations using schizophrenia-associated neurons. **a** Venn diagram showing impaired transcriptional response following 50 mM KCl treatment for 3 h in THC exposure hiPSC-derived neurons. **b** A similar decrease in significantly regulated transcripts following 50 mM KCl for 3 h is observed in schizophrenia-associated hiPSC-derived neurons. **c** A cohort of 5 control (C1–5) and 4 schizophrenia-associated (SZ1-4) cases were used for (**d**) candidate qRT–PCR analysis investigating *COX7A2*, *GRID2* and *HOMER1* following acute THC exposure. **e** Blunted effect of THC treatment can be seen in immediate early gene transcripts such as *NR4A1* and (**f**) *FOSB* following KCl-induced activation (Quantitative RT–PCR (qRT–PCR); Ordinary one-way ANOVA with Tukey’s multiple comparisons test: *p < 0.05, **p < 0.01, ***p < 0.001, ****p < 0.0001. *n* = 5 controls (see qRT–PCR, Ca–Ce, Supplementary Table S1); *n* = 4 schizophrenia (see qRT–PCR, S1–S4, Supplementary Table S1))

In our previous study^6^, we demonstrated that schizophrenia-associated hiPSC-derived neurons had a blunted transcriptomic response to KCl relative to controls. We repeated this experimental design on control hiPSC-derived neurons from four individuals, providing either acute (1 μM THC, 24 h), chronic (50 nM THC, 7 days) or vehicle treatment, after which cells were activated using 50 mM KCl (or vehicle) for 3 h as before. We saw a significantly blunted transcriptomic response, more prominent with the acute (75% reduction compared to KCl-activated control neurons; *p*-value = 1.3e−73, odds ratio = 278.6, Fisher’s exact test) than the chronic exposure (60% reduction compared to KCl-activated control neurons; *p*-value = 4.4e−83, odds ratio = 181.6, Fisher’s exact test) of THC (Fig. 4a; Supplementary Table S9; Supplementary Figure 4).

After re-running the raw schizophrenia-associated data from activity-dependent experiments conducted in Roussos et al^6^., we again saw a dramatic reduction (~93%; *p*-value = 4.3e−27, odds ratio = 605.7, Fisher’s exact test) in the schizophrenia-associated transcriptomic response (Fig. 4b; Supplementary Table S10). We tested candidate genes on a cohort of schizophrenia-associated hiPSC-derived neurons (Fig. 4c) and found blunted expression profiles for *COX7A2*, *GRID2* and *HOMER1* (Fig. 4d) using quantitative PCR. Quantitative PCR further confirmed this blunting effect of THC exposure; significantly reduced expression of immediate early genes such as *NR4A1* and *FOSB* was observed following KCl treatment (Fig. 4e, f), consistent with what we found previously for these genes in schizophrenia-associated hiPSC-derived neurons^6^.

## Discussion

Our results show that the endogenous response to THC operates through molecular pathways that have been strongly associated with psychiatric disease. This implies that genetic and epigenetic variation present in these specific pathways in individuals might determine the extent of individual susceptibility to adverse response to THC. Genes involved in autism and intellectual disability are prominently involved in THC signaling, while schizophrenia risk may be more linked to similarities when activity- dependent pathways are disrupted.

Understanding human brain subtleties requires a manipulable neuronal model; here we validate hiPSC-derived neuronal networks as a viable system for these types of studies. Others have similarly reported that treatment of hiPSC-derived dopaminergic neurons with THC reproduces effects observed in other mouse and human models^27^. Mitochondrial pathway dysfunction has been linked to THC exposure^24,25,28–30^ and schizophrenia^26,31^, a convergence that is captured in our system (Figs. 1b–d; 2c). Acute and chronic stress have differing effects on mitochondrial genes^32^ and the different respiratory chain complexes have unique functions during stress responses;^33,34^ this may explain the parallel differences between acute and chronic THC exposures observed here. Furthermore, links between the glutamatergic and ion channel pathways and THC reported here (Fig. 1b; 2a) are supported by previous studies where both glutamate and ion channel pathways are regulated via cannabinoid receptors^8,35^. Many of the ion channel proteins identified here are critical for acute synaptic activity^36^ but are also involved in later stage neural proliferation and differentiation^37,38^, suggesting that THC exposure may exert short- and/or long-term effects in the developing human brain.

Our experimental system enables relevant comparisons to known disease-causing genes in humans and led us to the significant overlaps in THC-induced genes and those involved in autism and intellectual disability (Fig. 3b). Moreover, by studying activity-related changes instead of baseline differences, we detected similarities between THC-induced hypo-function and schizophrenia (Fig. 4a, b), which would have otherwise been missed in a static system or post-mortem tissue. This is important as we found no overlap of significantly altered ion channel, WNT or mitochondrial genes between THC and schizophrenia datasets (Supplementary Table S8), suggesting that THC- and schizophrenia-related signaling pathways are different with respect to specific genes but convergent in function.

To test whether there was a significant enrichment for psychiatric disease genes in our THC results, we first constructed a list of disease-related genes from the literature, as there were no complete and up to date collections available. We generated comprehensive lists of currently known genes implicated in schizophrenia, autism and intellectual disability, finding significant correlations between THC treatment and autism and intellectual disability (Fig. 3b; Supplementary Table S7). Interestingly, a large recent GWAS uncovered four genes that were significantly associated with lifetime cannabis use^39^: one, *KCNT2*, showed significant THC-responsiveness in our hiPSC-derived neurons (Fig. 2a), while two others, *NCAM1* and *CADM2* (also known as *SynCAM2*) are important for postsynaptic function^40,41^, consistent with the enrichment of postsynaptic density genes in THC response (Fig. 2a).

Although we found abrogation of THC-induced changes in schizophrenia-associated hiPSC-derived neurons in candidate genes that were responsive to THC in our study, comprehensive biochemical and functional validation of our THC-induced effects are necessary, across both control and SZ neurons. Moreover, to confirm that these effects are mediated via cannabinoid signaling, future studies should attempt to recapitulate our observed effects using cannabinoid agonists (e.g., anandamide) or block them with selective antagonists (i.e., SR141716A). Consistent with this, we previously reported that THC-induced changes in gene expression in hiPSC neurons were blocked by concurrent 20 nM SR141716A treatment^23^.

In summary, we found significant associations of THC-related pathways to autism and intellectual disability. Furthermore, we have used a dynamic, human-relevant system to demonstrate a phenotypic link between THC treatment and schizophrenia. We hypothesize that THC exposure, by impacting many of the same synaptic and epigenetic pathways already associated with psychiatric disorders, may serve as an additive risk to existing genetic/epigenetic risk factors.

## Electronic supplementary material

Supplemental Information

Supplementary Table 1

Supplementary Table 2

Supplementary Table 3

Supplementary Table 4

Supplementary Table 5

Supplementary Table 6

Supplementary Table 7

Supplementary Table 8

Supplementary Table 9

Supplementary Table 10
