# Supplementary material for: THC exposure of human iPSC neurons impacts genes associated with neuropsychiatric disorders
Source: Transl Psychiatry. 2018 Apr 25;8:89. doi: 10.1038/s41398-018-0137-3 (PMC5915454; doi:10.1038/s41398-018-0137-3)
Supplement: Supplementary file 1 — Supplemental Information [file 41398_2018_137_MOESM1_ESM.pdf]

GENERATION OF GENE DATABASES

Mental Disorders

We have extensively (although not exhaustively) reviewed the literature and available databases in order to retrieve information on genes linked to three distinct mental disorders: autism (used here as a synonym autism spectrum disorders or ASDs), intellectual disabilities (ID), schizophrenia. Each of these are represented by variable clinical manifestations and thus many genes have been implicated as underlying causes in a vast number of studies. According to the WHO (Mental Disorders Fact Sheet of April 2016), the list of mental disorders also include depression, bipolar affective disorder, psychoses (in which schizophrenia is included), dementia, and developmental disorders (in which autism is included). The report states that “the burden of mental disorders continues to grow with significant impacts on health and major social, human rights and economic consequences in all countries of the world”. Estimated numbers agree with this, reporting 1 in 160 children to have an autism spectrum disorder and more than 21 million people worldwide.

Autism-Related Gene Database

We retrieved genes from different datasets: i) the set of 171 genes previously reported by Xu et al., 2012 [1] in the AutismKB database; ii) the 845 genes contained in the recently updated (September 2016) AutDB database [2]; iii) the 859 genes available in the Simons Foundation Autism Research Initiative (SFARI) gene database [3]; iv) data from the recently published article from the MSSNG project reporting 55 genes [4]; v) the 97 genes found to be mutated in the Whole Exome Sequencing project performed by Sener et al. [5] (which contain the 21 genes reported by O’Roak et al. [6]); vi) the 127 genes identified by DAWN, a novel algorithm developed by Liu et al. [7]; vii) the set of 17 genes identified by a genome-wide approach for copy number variation detection published by Glessner et al. [8] and viii) the 19 canonical Wnt pathway genes shown to be mutated in autism by Kalkman [9].

Many genes are redundantly reported in the literature: in total 131 genes are shared among the AutismKB gene list and the AutDB dataset, only 2 of the 845 genes from AutDB are not contained in SFARI (which comprises 16 other genes), 39 of the 55 genes from the latest MSSNG project report were also retrieved from at least one of the previously cited datasets, as mentioned the short list from O’Roak et al. is entirely contained in the longer list of Sener et al., at least 38 of the 127 genes identified by the new DAWN algorithm have been reported elsewhere, the genome-wide study from Glessner et al. reported 17 genes but only 6 were absent in previous datasets and also only 6 from the 19 genes reported by Kalkman were novel.

A non-redundant set of genes was generated from the aforementioned sources, resulting in a comprehensive (perhaps the most comprehensive to date) set of 1051 autism-related genes. Interestingly, studies have focused on protein-coding genes and there is a total absence of non-coding RNAs in these datasets. A perhaps more unexpected bias is that while 91 autism-related genes are located in the X chromosome (the highest number of genes per chromosome), the Y chromosome bears the lowest number of genes, only 2. This counts do not correlate with total gene content, at least for the X chromosome, which is only the 12th in number of protein-coding genes. One could suggest this discrepancy to be at some level linked to the higher prevalence of autism in males, or to reflect a bias on research studies available to date.

Intellectual Disability-Related Gene Database

We retrieved recent data from Chiurazzia and Pirozzi 2016 [10], containing 898 ID-related genes retrieved by using OMIM (Online Mendelian Inheritance in Man, a comprehensive, authoritative compendium of human genes which focuses on the relationship between phenotype and genotype) and the National Center for Biotechnology Information (NCBI) GENE databases. Interestingly, from these, 173 (nearly 20%) genes were also present in the previously described autism-related gene database of 1051 genes.

Additional databases were also consulted when retrieving information. Namely we have considered relevant: i) the set of 525 “confirmed” pathogenic genes reported by Gilissen et al. [11]; ii) the 1497 validated *de novo* single nucleotide variants and indels and the 65 *de novo* copy number variants reported by the Deciphering Developmental Disorders Study [12]; iii) the set of 923 confirmed developmental disorder genes reported in the updated version of DDG2P from 2013 [13]; and iv) the 253 genes validated by Grozeva et al. [14]. All of these additional databases add up to 2237 non-redundant genes, 544 of which are also present in the dataset from Chiurazzia and Pirozzi. In total we retrieved 2591 ID-related genes from these 5 datasets.

Schizophrenia-Related Gene Database

Wu et al. [15] have recently published a database (SZDB) with over 13 thousand entries of genes related to schizophrenia at some level (genes identified by GWAS, affected by CNVs, identified by linkage and association study, genes identified by convergent functional genomics, by Sherlock integrative analysis, identified by Pascal gene based test, and genes expressed differentially in schizophrenia). Different priority scores were given to genes listed from different sources and the higher priority was given to 489 genes derived from GWAS studies. We therefore decided to use those as a reliable dataset to narrow our reports.

The SZGR2 database [16] has multiple "sub-datasets". One is GWASScat, which includes 603 entries from the NHGRI-EBI GWAS Catalog. Some entries contain multiple genes and 70 represent intergenic regions. When every gene is considered and counted only once and intergenic regions excluded, there are 643 genes. The GWASdb sub-dataset has 100 additional entries, representing 42 unique genes. Together, the GWASScat and GWASdb sub-datasets represent 657 genes. We will focus on this list during our analyses. Together and non-redundantly, the SZDB plus the SZGR2 datasets comprise 754 genes (489 from the former and additional 286 from the latter).

The OMIM entries related to schizophrenia (181500 SCHIZOPHRENIA; SCZD / 600850 SCHIZOPHRENIA 4; SCZD4 / 604906 SCHIZOPHRENIA 9; SCZD9 / 613950 SCHIZOPHRENIA 15; SCZD15 / 615232 SCHIZOPHRENIA 18; SCZD18) comprise 26 unique entries, just one of which was contained in the previous list of 754 genes, thus adding 25 new genes to the final list of 779 genes.

GENE ANNOTATION

All three databases comprise together and non-redundantly 3869 genes, from which 372 genes are shown to be implicated in both autism and ID, 123 in both ID and schizophrenia and 101 in both autism and schizophrenia. Only ~1% of these (44) are common to all three conditions, according to our datasets. All genes were further tentatively mapped back to the human transcriptome as to retrieve their gene ID, chromosomal location. Annotation was successfully to 3671 (from the total of 3869) genes: 1037 from the autism-related list, 2461 from the ID-related list and 723 from the schizophrenia-related list. The remaining genes were possibly re-annotated or discontinued after further investigation. When these annotated genes are considered, the X chromosome is the 5th most populated (with more than 200 genes) and the Y is the least (with only 3 genes).

DIFFERENTIAL EXPRESSION OF DISEASE-RELATED GENES UPON THC TREATMENT

Using the set of 886 annotated differentially expressed genes (DEGs) upon THC treatment, we investigated the ones related to each of the aforementioned conditions and results are as follows:

Condition	Autism	Intellectual Disabilities	Schizophrenia
Single-dose	51	87	12
Multiple-dose	72	154	29
Total	80	167	30

In total and taking both the single-dose and multiple-dose experiments into account and only annotated genes, there are 80 autism-associated DEGs, 167 ID-associated DEGs and 30 schizophrenia-associated DEGs, comprising 233 unique genes. As we have reported a total of 1442 genes (annotated or not) to be differentially expressed upon THC exposure, we conclude that >15% of these are related to at least one of the investigated mental disorders. This number is much higher, over 25%, if we only take the 886 annotated DEGs into account. This means that when we only consider annotated genes, over one quarter of the genes for which the expression is affected by THC is related to at least one of these mental disorders. Six genes are in both the autism-related and schizophrenia-related DEGs, 34 shared between autism-related and ID-related DEGs and 7 present in both the ID-related and schizophrenia-related DEGs lists. Three genes are shared among all three DEG sets: EP300, MECP2 and ZBTB20. Interestingly, McCarthy et al. [17] also report MECP2 as a common genetic factor underlying all three conditions and further suggests epigenetic factors to play a central role in susceptibility.

STATISTICAL SIGNIFICANCE OF OUR OBSERVED RESULTS

Results of Fisher exact test show the correlation between differential expression after THC treatment and disease-related genes in our data is statistically relevant. Random datasets of the same size lead to very different results, none of which is above the significance threshold set by a p-value of less than 0.01 (data not shown).

Datasets of differentially expressed genes (DEGs) after single-dose and-multiple dose of THC exposure were merged to yield the complete dataset of DEGs. The dataset contains hundreds of unnamed genes (denoted by NA, being 394 for single-dose and 462 for multiple-dose), which were removed from further analyses. A resulting set of 898 genes is generated, only one (LINC01420) being non-protein-coding, which was also excluded from this statistical analysis. The exclusion resulted in a dataset of 897 genes, 886 of which were annotated based on the human genome version GRCh38rel79. Because virtually all genes are protein-coding (the reason for the exclusion of the ncRNA described above), we have only considered the protein-coding portion of the human genome, which in the GRCh38rel79 version contains 19903 genes. To test the occurrence of positive association between the genes related to disease and the genes that are differentially expressed, we used the Fisher exact test analysis in the R package, which is implemented in the GeneOverlap library as described below.

The GeneOverlap class formulates the problem as testing whether two variables are independent, which can be represented as a contingency table, and then uses Fisher's exact test to find the statistical significance. Here the P-value indicates whether the overlap is significant and how much. The Fisher's exact test also gives an odds ratio which represents the strength of association. If an odds ratio is equal to or less than 1, there is no association between the two lists. If the odds ratio

is much larger than 1, then the association is strong. The class also calculates the Jaccard index which measures the similarity between two lists. The Jaccard index varies between 0 and 1, with 0 meaning there is no similarity between the two and 1 meaning the two are identical. Results are as follows:

Condition	Genes in Dataset	DEGs	P-value	Corrected P-value	Odds Ratio	Jaccard Index
Int. Disability	2461	167	1.1e-08	3.3e-08	1.7	0.1
Autism	1037	80	1.1e-06	3.3e-06	1.9	0.0
Schizophrenia	723	30	0.68	1	0.9	0.0

REFERENCES

[1] Xu LM, Li JR, Huang Y, Zhao M, Tang X, Wei L.(2012) AutismKB: an evidence-based knowledgebase of autism genetics. Nucleic Acids Res, 40:D1016-1022

[2] Basu SN, Kollu R, Banerjee-Basu S (2009) Nucleic Acids Res. Database issue:D832-836

[3] Banerjee-Basu S, Packer A. (2010) SFARI Gene: an evolving database for the autism research community. Disease Models & Mechanisms, 3:133-135

[4] Yuen RKC, Merico D, Bookman M, et al. (2017) Whole genome sequencing resource identifies 18 new candidate genes for autism spectrum disorder. Nature Neuroscience, Published online 06 March 2017

[5] Sener EF, Canatan H, Ozkul Y (2016) Recent Advances in Autism Spectrum Disorders: Applications of Whole Exome Sequencing Technology. Psychiatry Investig, 13(3):255-264

[6] O'Roak BJ, Deriziotis P, Lee C, Vives L, Schwartz JJ, Girirajan S, Karakoc E, MacKenzie AP, Ng SB, Baker C, Rieder MJ, Nickerson DA, Bernier R, Fisher SE, Shendure J, Eichler EE (2011) Exome sequencing in sporadic autism spectrum disorders identifies severe de novo mutations. Nat Genet. 43(6):585–589.

[7] Liu L, Lei J, Sanders SJ, Willsey AJ, Kou Y, Cicek AE, Klei L, Lu C, He X, Li M, Muhle RA, Ma'ayan A, Noonan JP, Šestan N, McFadden KA, State MW, Buxbaum JD, Devlin B, Roeder K (2014) DAWN: a framework to identify autism genes and subnetworks using gene expression and genetics. Molecular Autism, 22

[8] Glessner JT, Wang K, Cai G, Korvatska O, Kim CE, Wood S, Zhang H, Estes A, Brune CW, Bradfield JP, Imielinski M, Frackelton EC, Reichert J, Crawford EL, Munson J, Sleiman PM, Chiavacci R, Annaiah K, Thomas K, Hou C, Glaberson W, Flory J, Otieno F, Garris M, Soorya L, Klei L, Piven J, Meyer KJ, Anagnostou E, Sakurai T, Game RM, Rudd DS, Zurawiecki D, McDougle CJ, Davis LK, Miller J, Posey DJ, Michaels S, Kolevzon A, Silverman JM, Bernier R, Levy SE, Schultz RT, Dawson G, Owley T, McMahon WM, Wassink TH, Sweeney JA, Nurnberger JI, Coon H, Sutcliffe JS, Minshew NJ, Grant SF, Bucan M, Cook EH, Buxbaum JD, Devlin B, Schellenberg GD, Hakonarson H (2009) Autism genome-wide copy number variation reveals ubiquitin and neuronal genes. Nature, 459(7246):569-73

[9] Hans Otto Kalkman. A review of the evidence for the canonical Wnt pathway in autism spectrum disorders. Molecular Autism20123:10. DOI: 10.1186/2040-2392-3-10

[10] Chiurazzi P, Pirozzi F. Advances in understanding – genetic basis of intellectual disability (2016) F1000Research, 5:F1000 Faculty Rev-599

[11] Gilissen C, Hehir-Kwa JY, Thung DT, van de Vorst M, van Bon BW, Willemsen MH, Kwint M, Janssen IM, Hoischen A, Schenck A, Leach R, Klein R, Tearle R, Bo T, Pfundt R, Yntema HG, de Vries BB, Kleefstra T, Brunner HG, Vissers LE, Veltman JA (2014) Genome sequencing identifies major causes of severe intellectual disability. Nature, 511(7509):344-7

[12] Deciphering Developmental Disorders Study (2015) Large-scale discovery of novel genetic causes of developmental disorders. Nature, 519(7542):223-228.

[13] Wright CF, Fitzgerald TW, Jones WD, Clayton S, McRae JF, van Kogelenberg M, King DA, Ambridge K, Barrett DM, Bayzetinova T, Bevan AP, Bragin E, Chatzimichali EA, Gribble S, Jones P, Krishnappa N, Mason LE, Miller R, Morley KI, Parthiban V, Prigmore E, Rajan D, Sifrim A, Swaminathan GJ, Tivey AR, Middleton A, Parker M, Carter NP, Barrett JC, Hurles ME, FitzPatrick DR, Firth HV, Deciphering Developmental Disorders Study (2015) Genetic diagnosis of developmental disorders in the DDD study: a scalable analysis of genome-wide research data. Lancet, 385(9975):1305-14.

[14] Grozeva D, Carss K, Spasic-Boskovic O, Tejada MI, Gecz J, Shaw M, Corbett M, Haan E, Thompson E, Friend K, Hussain Z, Hackett A, Field M, Renieri A, Stevenson R, Schwartz C, Floyd JA, Bentham J, Cosgrove C, Keavney B, Bhattacharya S, Italian X-linked Mental Retardation Project, UK10K Consortium, GOLD Consortium, Hurles M, Raymond FL. (2015) Targeted Next-Generation Sequencing Analysis of 1,000 Individuals with Intellectual Disability. Hum Mutat., 36(12):1197-204.

[15] Wu Y, Yao YG, Luo. XJ (2017) SZDB: A Database for Schizophrenia Genetic Research. Schizophr Bull, 43(2):459-471

[16] Jia P, Han G, Zhao J, Lu P, Zhao Z (2017) SZGR 2.0: a one-stop shop of schizophrenia candidate genes. Nucleic Acids Res, 45 (D1): D915-D924

[17] McCarthy SE, Gillis J, Kramer M, Lihm J, Yoon S, Berstein Y, Mistry M, Pavlidis P, Solomon R, Ghiban E, Antoniou E, Kelleher E, O'Brien C, Donohoe G, Gill M, Morris DW, McCombie WR, Corvin A. (2014) De novo Mutations in Schizophrenia Implicate Chromatin Remodeling and Support a Genetic Overlap with Autism and Intellectual Disability. Mol Psychiatry,19(6):652

A

B

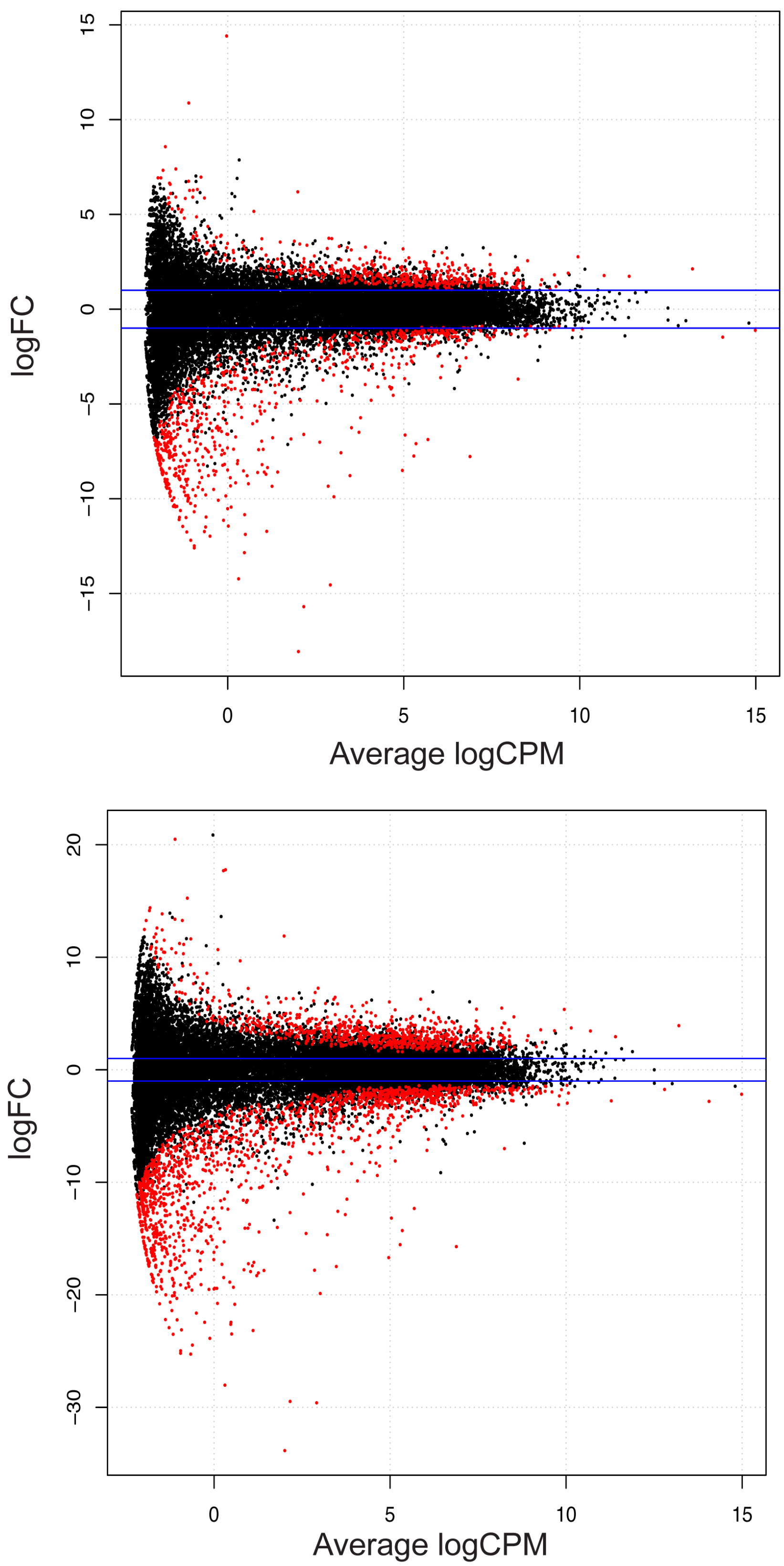

Supplementary Figure 1: MA plots showing the distribution of genes following either (A) acute or (B) chronic THC treatment compared with untreated iPSC-derived neurons. Significantly altered genes are depicted as red dots.

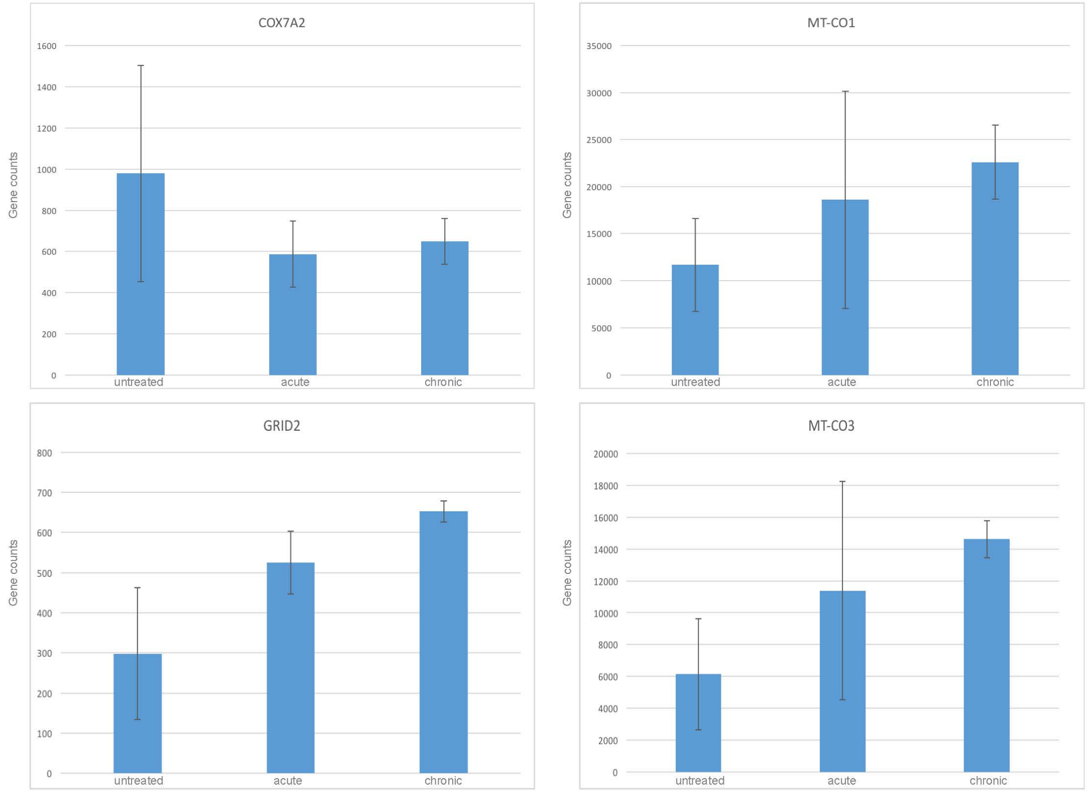

Supplementary Figure 2: RNA sequencing quantification results for candidate genes following acute or chronic THC treatment.

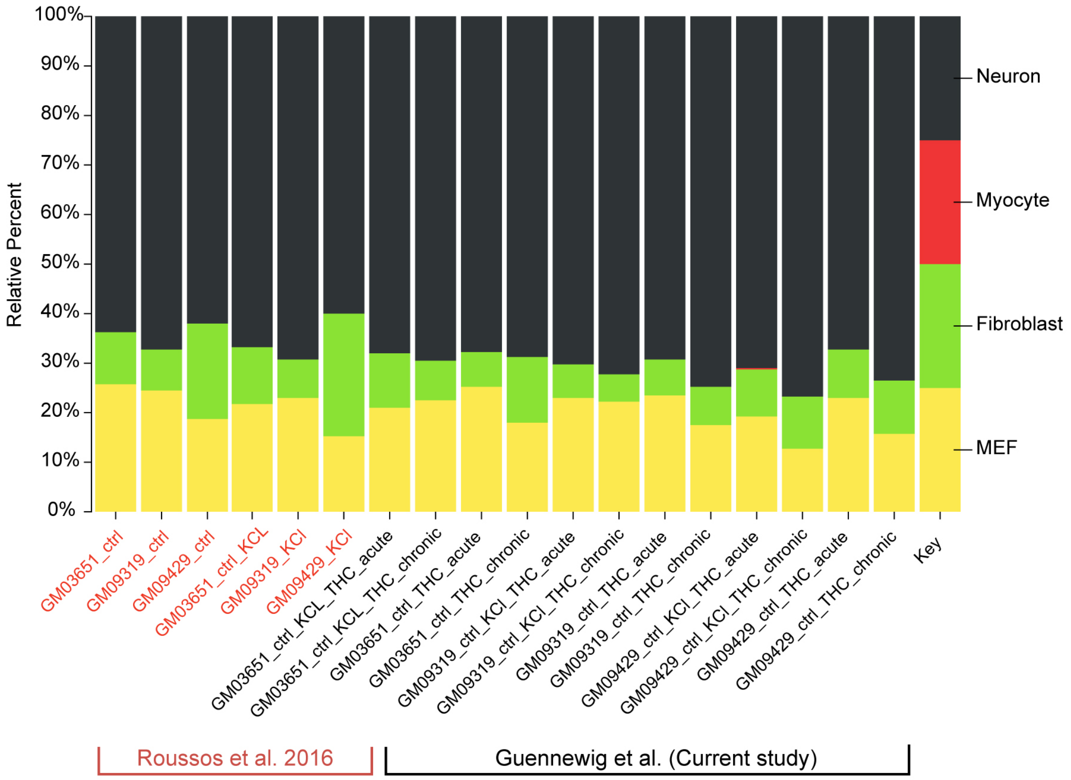

Supplementary Figure 3: Cell distribution is similar across samples between Roussos et al. study and our current study.

We depict the estimate of the bulk RNA sequencing cell tissue composition based on single cell RNA sequencing (e.g. Hoffman et al., **doi:** https://doi.org/10.1101/185546). The reference panel is based on Zhang et al and cell culture of single neural cells (Treutlein et al). The simulation was built using the online tool CIBERSORT (Newman et al).

Cell-type decomposition is a computational inference of cell type, not based on the physical counting of cells and only as accurate as the source single cell RNAseq datasets. The percentage composition should not be considered equivalent to cell number; the fibroblast/MEF signature could reflect non-neuronal cells and/or neural crest, which has an overlapping gene signature (e.g. Hoffman et al., **doi:** https://doi.org/10.1101/185546).

Zhang, Y. et al. An RNA-sequencing transcriptome and splicing database of glia, neurons and vascular cells of the cerebral cortex. J. Neurosci 34, 11929-47 (2014).

Treutlin, B. et al. Dissecting direct reprogramming from fibroblast to neuron using single-cell RNA-seq. Nature 534, 391-5 (2016).

Newman, A.M. et al. Robust enumeration of cell subsets from tissue expression profiles. Nat. Methods 12, 453-7 (2015).

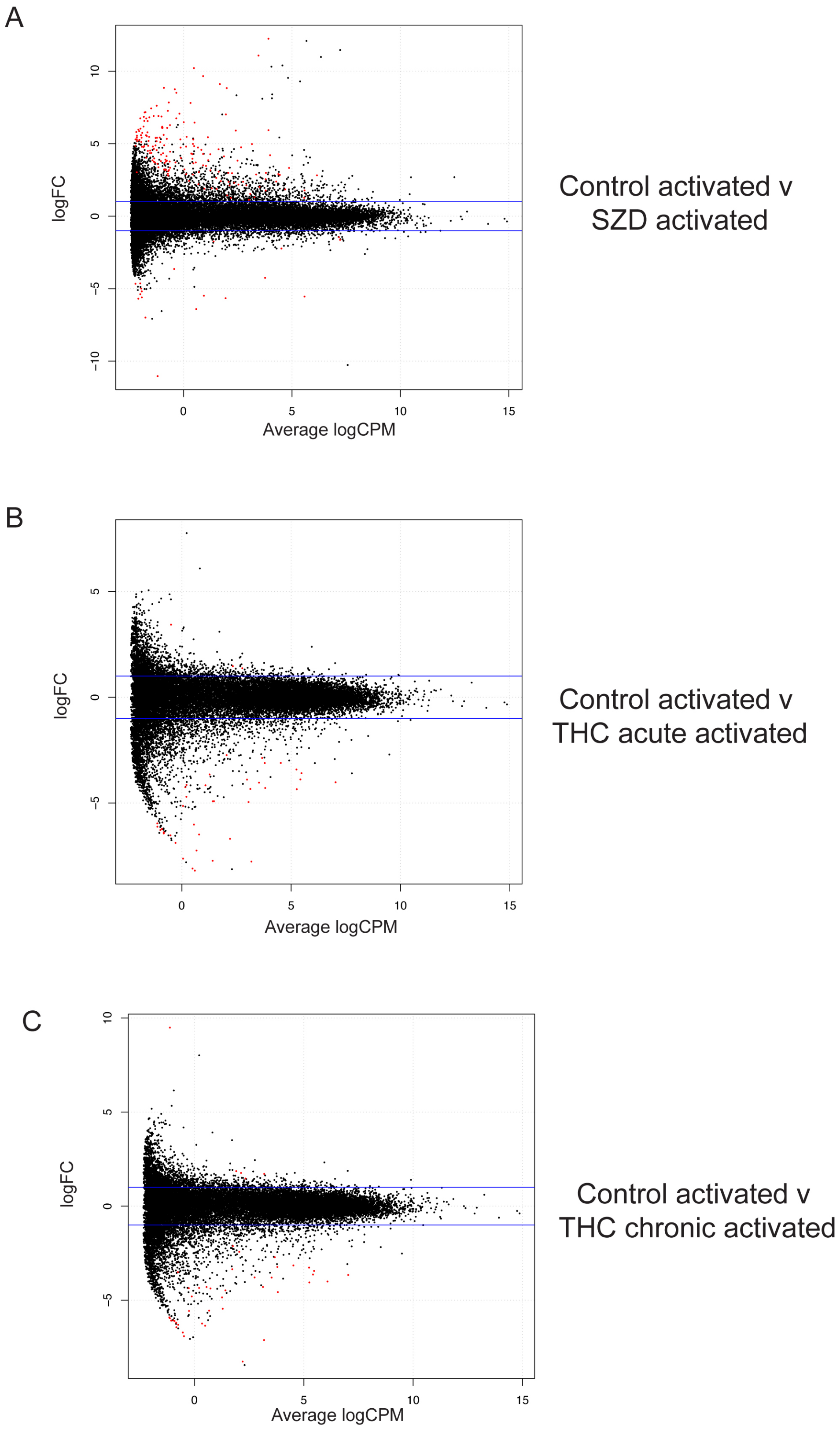

Supplementary Figure 4: MA plots showing the distribution of genes following KCl-mediated activation of iPSC-derived neurons that were either (A) schizophrenia (SZD)-associated or (B) treated acutely with THC (C) or chronically with THC. Significantly altered genes are depicted as red dots.
