# Supplementary material for: THC exposure of human iPSC neurons impacts genes associated with neuropsychiatric disorders
Source: Transl Psychiatry. 2018 Apr 25;8:89. doi: 10.1038/s41398-018-0137-3 (PMC5915454; doi:10.1038/s41398-018-0137-3)
Supplement: Supplementary file 2 — Supplementary Table 1 [file 41398_2018_137_MOESM2_ESM.pdf]

Supplementary Table 1: Patient samples used in RNA sequencing or quantitative RT-PCR.

**Experiment**	**Individual**	**hiPSC / NPC line ID**
RNAseq	Ca	BJ hiPSC#2 NPC#A
RNAseq	Cb	GM03440 hiPSC#5 NPC#A
RNAseq	Cc	GM03651 hiPSC#A NPC#A
RNAseq	Cd	AG09319 hiPSC#2 NPC#A
RNAseq	Ce	AG09429 hiPSC#3 NPC#A
qRT-PCR	Ca	BJ hiPSC#2 NPC#A
qRT-PCR	Cb	GM03440 hiPSC#5 NPC#A
qRT-PCR	Cc	GM03651 hiPSC#A NPC#A
qRT-PCR	Cd	GM04506 hiPSC#B NPC#A
qRT-PCR	Ce	AG09319 hiPSC#2 NPC#A
qRT-PCR	S1	GM01792 hiPSC#1 NPC#A, GM01792 hiPSC#1 NPC#E
qRT-PCR	S2	GM02038 hiPSC#1 NPC#A, GM02038 hiPSC#1 NPC#B
qRT-PCR	S3	GM01835 hiPSC#1 NPC#5
qRT-PCR	S4	GM02497 hiPSC#1 NPC#C

(In the individual column, ‘C’ denotes control while ‘S’ denotes schizophrenia)
