# Supplementary material for: THC exposure of human iPSC neurons impacts genes associated with neuropsychiatric disorders
Source: Transl Psychiatry. 2018 Apr 25;8:89. doi: 10.1038/s41398-018-0137-3 (PMC5915454; doi:10.1038/s41398-018-0137-3)
Supplement: Supplementary file 3 — Supplementary Table 2 [file 41398_2018_137_MOESM3_ESM.pdf]

Supplementary Table 2: Quantitative PCR primers

Gene	Forward primer (5'-3')	Reverse primer (5'-3')	Accession #
COX7A2	CCCCAAAGAAGAGCTCGGTT	AGAAGCAGGAGTGACTTCAGTC	NM_001865.3
MT-CO1	GGAGGAGGAGACCCCATTCT	TATGGGAGATTATTCCGAAGCCTG	MF441747.1
MT-CO3	CAGCCCATGACCCCTAACAG	AGGCCTAGTATGAGGAGCGT	NC_012920.1
GRID2	AACAAGAGGAAAGGCTCCCG	TCGTCATCTGTGCACAAGCT	NM_001510.3
HOMER1	TAACGAAGGCAGCTGAGTCC	AGACCAGCCTGTCAATGACG	NM_004272.4
NR4A1	GGTGACCCCACGATTTGTCT	GGCTTATTTACAGCACGGCG	NM_002135.4
hFOSB	CACTCCCGAAAGCCTTTCCT	CCCTTCTCGGTTCTGTCACC	NM_001114171.1
