# Supplementary material for: THC exposure of human iPSC neurons impacts genes associated with neuropsychiatric disorders
Source: Transl Psychiatry. 2018 Apr 25;8:89. doi: 10.1038/s41398-018-0137-3 (PMC5915454; doi:10.1038/s41398-018-0137-3)
Supplement: Supplementary file 4 — Supplementary Table 3 [file 41398_2018_137_MOESM4_ESM.pdf]

Supplementary Table 3: Significant gene expression changes in response to THC treatments

Significantly altered genes between control and THC (acute) treated iPSC-derived neurons.

Ensembl gene ID	logFC	logCPM	PValue	FDR	hgnc_symbol	chromosome	start_position	end_position	strand
ENSG00000160179	-2.04564374	5.997429796	4.00E-05	0.002656	ABCG1	21	42199689	42297244	1
ENSG00000146109	-1.29907356	4.517272084	0.00014994	0.00673224	ABT1	6	26596952	26600744	1
ENSG00000014257	-9.43564432	-1.54540333	2.46E-05	0.0019672	ACPP	3	132317367	132368298	1
ENSG00000129467	-7.3830549	-1.571050763	3.24E-05	0.00233695	ADCY4	14	24318349	24335093	-1
ENSG00000116863	-1.33570742	5.252789003	0.00010503	0.00529669	ADPRHL2	1	36088875	36093932	1
ENSG00000181026	-2.00750461	6.132771524	0.00011648	0.00570547	AEN	15	88621296	88632282	1
ENSG00000187546	-8.72253033	-0.781421738	0.00010983	0.00545072	AGMO	7	15200318	15562015	-1
ENSG00000092847	1.212345024	6.834515835	0.0002674	0.00999396	AGO1	1	35869808	35930528	1
ENSG00000169692	-2.0167907	2.625411825	3.92E-05	0.00264455	AGPAT2	9	136673143	136687423	-1
ENSG00000126705	2.377874775	3.452013963	0.00019524	0.00795576	AHDC1	1	27534035	27604431	-1
ENSG00000059573	-1.2741398	6.708504603	0.0001531	0.00683759	ALDH18A1	10	95605929	95656706	-1
ENSG00000184254	-9.33511111	0.707051955	9.21E-06	0.00099012	ALDH1A3	15	100877714	100916626	1
ENSG00000091542	-0.96135455	6.762190485	0.0001989	0.00807612	ALKBH5	17	18183078	18209954	1
ENSG00000123505	-1.52205232	7.522791073	5.68E-05	0.00340453	AMD1	6	110814621	110895713	1
ENSG00000104899	2.108769934	1.28558715	0.00024624	0.00943333	AMH	19	2249309	2252073	1
ENSG00000169604	2.172723956	6.248975281	0.00010517	0.00529669	ANTXR1	2	69013178	69249327	1
ENSG00000264230	-5.22801928	-0.866766224	6.25E-06	0.00073461	ANXA8L1	10	46375627	46537864	1
ENSG00000134982	1.492284138	9.264868086	3.99E-05	0.002656	APC	5	112707498	112846239	1
ENSG00000244509	1.953321838	2.784880164	0.00016865	0.00721021	APOBEC3C	22	39014083	39020352	1
ENSG00000169083	1.378261946	4.784891511	5.44E-05	0.00331826	AR	X	67544032	67730619	1
ENSG00000109321	-9.21131185	-1.655630195	5.22E-05	0.00324328	AREG	4	74445134	74455009	1
ENSG00000168374	-1.62939031	7.748531487	1.04E-05	0.00106708	ARF4	3	57571363	57598220	-1
ENSG00000187951	2.289647306	0.925473827	0.00011841	0.005775	ARHGAP11B	15	30624494	30772993	1
ENSG00000180801	-1.97591999	5.092245047	0.00018919	0.00779372	ARSJ	4	113900284	113979727	-1
ENSG00000153317	1.533228116	4.890277877	0.00022375	0.00877031	ASAP1	8	130052104	130443660	-1
ENSG00000162772	-2.68995189	4.100110753	0.00015243	0.00682609	ATF3	1	212565334	212620777	1
ENSG00000128272	-1.20983088	8.583576392	0.00022618	0.00885558	ATF4	22	39519695	39522685	1
ENSG00000170653	2.011416681	3.284127878	8.51E-05	0.0045862	ATF7	12	53507856	53626410	-1
ENSG00000171681	1.936147914	7.824670977	5.38E-05	0.0032959	ATF7IP	12	14365632	14502935	1
ENSG00000123395	-1.45938162	5.134100158	3.76E-05	0.0025859	ATG101	12	52069246	52077494	1
ENSG00000117410	-1.54387162	6.234005492	1.52E-05	0.00136165	ATP6V0B	1	43974487	43978295	1
ENSG00000183778	2.026807493	3.808794102	5.80E-06	0.00070264	B3GALT5	21	39556442	39673137	1
ENSG00000266074	2.56603418	2.275766652	1.87E-05	0.00160169	BAHCC1	17	81395475	81466332	1
ENSG00000258643	2.040905642	3.774967539	3.86E-05	0.00262916	BCL2L2-PABPN1	14	23306835	23325369	1
ENSG00000176697	-3.39606855	4.716966021	1.22E-05	0.00119578	BDNF	11	27654893	27722058	-1
ENSG00000125378	-3.57786062	2.089552973	8.67E-06	0.0009617	BMP4	14	53949736	53958761	-1
ENSG00000169594	-9.14338629	0.718516128	3.77E-06	0.00051868	BNC1	15	83255903	83284716	-1
ENSG00000139618	2.213787091	4.558045861	0.00016699	0.00717547	BRCA2	13	32315474	32400266	1
ENSG00000174744	-1.56403198	4.981349918	1.61E-05	0.0014259	BRMS1	11	66337333	66345125	-1
ENSG00000166167	1.120818799	6.113200555	0.00023875	0.00923033	BTRC	10	101354033	101557321	1
ENSG00000173237	-4.66712549	1.385861746	8.90E-05	0.00472765	C11orf86	11	66975277	66977004	1
ENSG00000103544	1.139984867	5.988212709	0.00022096	0.00868116	C16orf62	16	19555240	19706793	1
ENSG00000116922	-1.06549011	5.110172111	0.00023524	0.00912581	C1orf109	1	37681570	37692249	-1
ENSG00000162757	-1.78565816	2.801233188	0.00010852	0.00541712	C1orf74	1	209779208	209784559	-1
ENSG00000184208	1.654476439	4.617715987	7.34E-05	0.00415125	C22orf46	22	41688939	41698136	1
ENSG00000153790	2.709801317	2.16531769	3.90E-05	0.00263676	C7orf31	7	25134697	25180356	-1
ENSG00000270024	-4.0923518	0.063378313	0.00017977	0.00756188	C8orf44-SGK3	8	66667596	66860472	1
ENSG00000105605	1.886469575	3.461360566	4.45E-05	0.00286491	CACNG7	19	53909335	53943941	1
ENSG00000112186	1.728776194	5.003458437	8.91E-06	0.00097836	CAP2	6	17393216	17557792	1
ENSG00000106144	2.172075416	3.832153993	7.67E-06	0.00087131	CASP2	7	143288215	143307696	1
ENSG00000110395	1.910078933	6.152916062	1.25E-05	0.00121351	CBL	11	119206276	119308149	1
ENSG00000141668	-3.53096834	0.410709741	0.00015635	0.00689271	CBLN2	18	72536680	72638521	-1
ENSG00000094916	1.6259289	9.256712441	5.57E-06	0.00068225	CBX5	12	54230940	54280133	-1
ENSG00000171097	-1.45073622	3.481666145	1.54E-05	0.00137668	CCBL1	9	128832942	128882494	-1
ENSG00000115355	1.815413816	8.0772405	3.81E-05	0.00260886	CCDC88A	2	55287842	55419921	-1
ENSG00000100814	-1.24832962	5.535003948	0.00015925	0.00693964	CCNB1IP1	14	20311368	20333312	-1
ENSG00000112576	-2.14112492	5.205687999	6.76E-05	0.0038677	CCND3	6	41934933	42050357	-1
ENSG00000085117	-2.11408192	2.220962502	2.62E-05	0.00204894	CD82	11	44564427	44620363	1
ENSG00000062038	-2.78576871	1.508043531	9.38E-05	0.00490504	CDH3	16	68636189	68722616	1
ENSG00000124762	-2.19978251	6.758797041	3.99E-07	0.00010033	CDKN1A	6	36676460	36687339	1
ENSG00000140743	-2.14525865	4.306373817	4.83E-05	0.00305784	CDR2	16	22345936	22437165	-1
ENSG00000245848	-4.85551539	1.506866743	6.16E-07	0.00013407	CEBPA	19	33299934	33302564	-1
ENSG00000048740	1.466101778	6.380695923	0.00018647	0.00774334	CELF2	10	11005321	11336675	1
ENSG00000138135	-7.99977526	0.813848965	2.50E-06	0.00038285	CH25H	10	89205629	89207314	-1
ENSG00000171316	1.600673077	7.136992552	9.00E-05	0.00474939	CHD7	8	60678778	60868028	1
ENSG00000175505	-6.82721243	2.750227796	4.95E-09	3.80E-06	CLCF1	11	67364168	67374177	-1
ENSG00000189143	-6.39939026	-1.018906761	3.16E-05	0.00231871	CLDN4	7	73799542	73832693	1
ENSG00000182372	-2.08814669	1.36839799	7.96E-05	0.00440915	CLN8	8	1755778	1786572	1
ENSG00000205423	-1.14268393	4.977347113	0.00012065	0.00586239	CNEP1R1	16	50024410	50037088	1
ENSG00000137161	1.347814595	4.780228075	0.000158	0.00693816	CNPY3	6	42929192	42939287	1
ENSG00000136152	-1.09486431	6.201055456	6.44E-05	0.00374578	COG3	13	45464898	45536630	1
ENSG00000133103	-1.102308	6.200708431	0.00021724	0.00857445	COG6	13	39655627	39791665	1
ENSG00000129083	-1.24412521	8.166996322	6.07E-05	0.00359113	COPB1	11	14443440	14500027	-1
ENSG00000115520	-2.10057354	5.625102204	7.88E-07	0.00015736	COQ10B	2	197453423	197475308	1
ENSG00000112695	-1.38459647	6.297261301	6.76E-06	0.00078638	COX7A2	6	75237675	75250323	-1
ENSG00000143320	-3.9138906	3.074196642	2.32E-05	0.00187929	CRABP2	1	156699606	156705816	-1

ENSG00000107175	-1.08138338	6.041241525	8.22E-05	0.00448861	CREB3	9	35732320	35737007	1
ENSG00000006016	2.492247603	3.74777982	0.00018685	0.00774414	CRLF1	19	18572220	18607741	-1
ENSG00000144655	-3.0103787	5.184234624	3.01E-06	0.00043461	CSRNP1	3	39141855	39154562	-1
ENSG00000175215	1.762802896	6.584629159	5.66E-05	0.00340453	CTDSP2	12	57819927	57846739	-1
ENSG00000180891	1.397876847	5.922072132	2.34E-05	0.00189191	CUEDC1	17	57861243	57955323	-1
ENSG00000163739	-8.59403292	-0.298110196	2.73E-05	0.00212454	CXCL1	4	73869393	73871242	1
ENSG00000169429	-6.78689405	-0.798133808	1.20E-06	0.00021257	CXCL8	4	73740506	73743716	1
ENSG00000166394	-4.01790763	-0.358530741	3.36E-05	0.00238179	CYB5R2	11	7665100	7677222	-1
ENSG00000070190	-6.29202623	-1.150277891	4.90E-05	0.00309892	DAPP1	4	99816833	99870154	1
ENSG00000136485	1.500070624	6.981807686	2.28E-05	0.00186093	DCAF7	17	63550461	63594266	1
ENSG00000167986	-0.90655258	9.066884553	0.00024539	0.00941805	DDB1	11	61299451	61342596	-1
ENSG00000134574	-2.15804994	4.604915871	4.51E-06	0.00058714	DDB2	11	47214465	47239240	1
ENSG00000175197	-2.45721933	4.93268177	9.58E-07	0.00018083	DDIT3	12	57516588	57520517	-1
ENSG00000168209	-2.20429252	5.410296971	0.00012125	0.00587163	DDIT4	10	72273920	72276036	1
ENSG00000175984	-2.63887951	0.717835649	0.00014324	0.00654413	DENND2C	1	114582848	114670422	-1
ENSG00000139734	2.075711292	3.722667039	0.00016085	0.00698207	DIAPH3	13	59665583	60163987	-1
ENSG00000165023	-1.66084565	7.080962029	0.0001055	0.00529771	DIRAS2	9	90609832	90643104	-1
ENSG00000050165	-1.93919907	10.0698347	0.0001425	0.00651906	DKK3	11	11963106	12009769	-1
ENSG00000104936	1.412358732	4.537357012	3.14E-05	0.0023122	DMPK	19	45769717	45782552	-1
ENSG00000185800	1.264830051	4.700760747	3.25E-05	0.00233695	DMWD	19	45782947	45792802	-1
ENSG00000135392	-1.32298542	5.496769375	0.00013467	0.00626229	DNAJC14	12	55820960	55830824	-1
ENSG00000130816	1.308082321	5.500927292	0.00025442	0.00960713	DNMT1	19	10133345	10231286	-1
ENSG00000135905	1.597838215	6.67383479	0.00012973	0.0060851	DOCK10	2	224765090	225042445	-1
ENSG00000111817	-2.19040635	5.487341487	4.35E-07	0.00010542	DSE	6	116254173	116444860	1
ENSG00000096696	-3.48779062	4.511778525	0.00023391	0.00909506	DSP	6	7541575	7586717	1
ENSG00000120129	-2.02862233	4.799963199	4.30E-05	0.00280619	DUSP1	5	172768090	172771195	-1
ENSG00000165891	1.786569315	3.682364473	0.00010739	0.00537663	E2F7	12	77021247	77065580	-1
ENSG00000145194	-3.18789806	1.353649407	1.09E-06	0.00019959	ECE2	3	184249650	184293031	1
ENSG00000127129	-7.64916025	-1.506669986	8.16E-05	0.0044757	EDN2	1	41478775	41484673	-1
ENSG00000124205	-9.65202379	-0.247906413	0.00022781	0.00890887	EDN3	20	59300427	59325992	1
ENSG00000169242	-3.33172059	2.492452693	2.36E-08	1.23E-05	EFNA1	1	155127460	155134857	1
ENSG00000146648	2.4241309	4.978863659	1.17E-05	0.00114891	EGFR	7	55019021	55256620	1
ENSG00000122877	-5.17289297	2.20986425	9.04E-06	0.0009804	EGR2	10	62811996	62919900	-1
ENSG00000179388	-5.92338554	0.400184636	0.00012528	0.00598653	EGR3	8	22687659	22693302	-1
ENSG00000024422	2.037455454	2.220456855	0.00015342	0.00683759	EHD2	19	47713343	47743134	1
ENSG00000172071	-1.77917633	6.1911559	2.88E-06	0.00042189	EIF2AK3	2	88556741	88627576	-1
ENSG00000148730	1.403908856	6.534875967	3.19E-05	0.00233505	EIF4EBP2	10	70404379	70428618	1
ENSG00000132205	-5.24781728	1.30162427	7.24E-06	0.00083309	EMILIN2	18	2847030	2915993	1
ENSG00000134531	-2.39908352	7.083612685	7.46E-05	0.00421402	EMP1	12	13196716	13219939	1
ENSG00000142627	-3.10555777	3.236182694	3.06E-05	0.00229336	EPHA2	1	16124337	16156087	-1
ENSG00000143819	2.637277523	4.879749349	9.06E-07	0.00017382	EPHX1	1	225810092	225845563	1
ENSG00000063245	1.775378209	3.954092936	9.74E-05	0.00502481	EPN1	19	55675226	55709858	1
ENSG00000082805	1.273105701	6.625043031	0.00021554	0.00854065	ERC1	12	990509	1495933	1
ENSG00000124882	-8.96577946	-0.542882529	2.10E-05	0.00175307	EREG	4	74365143	74388751	1
ENSG00000115363	-3.1673647	4.602192499	3.30E-05	0.00235482	EVA1A	2	75469302	75569722	-1
ENSG00000168040	-2.0567543	4.572728357	7.83E-07	0.00015731	FADD	11	70203163	70207390	1
ENSG00000048828	1.431698779	6.439713467	0.00012979	0.0060851	FAM120A	9	93451722	93566107	1
ENSG00000175182	-1.66419857	6.116488376	0.00018793	0.00776991	FAM131A	3	184335926	184346275	1
ENSG00000170264	1.294686552	4.68921851	0.0001436	0.00655153	FAM161A	2	61824854	61854143	-1
ENSG00000143340	-6.45941544	-1.087414026	7.84E-05	0.00435654	FAM163A	1	179743163	179816198	1
ENSG00000164125	1.948660687	6.376637239	0.00015793	0.00693816	FAM198B	4	158124474	158173318	-1
ENSG00000185614	-4.85952159	-0.569596941	4.02E-05	0.00266289	FAM212A	3	49803254	49805030	1
ENSG00000157870	1.942754008	1.446245656	9.42E-05	0.00490504	FAM213B	1	2586491	2591469	1
ENSG00000158246	-2.39053937	3.569520685	2.90E-05	0.00220173	FAM46B	1	27005020	27012836	-1
ENSG00000166595	-1.19281525	5.283341797	0.0001087	0.00541814	FAM96B	16	66932055	66934423	-1
ENSG00000165323	1.466231319	8.294316082	0.0001091	0.00542955	FAT3	11	92352096	92896470	1
ENSG00000068078	-1.77396867	4.988305361	0.00025495	0.00961574	FGFR3	4	1793307	1808872	1
ENSG00000115641	-2.83340984	5.077366105	5.69E-05	0.00340453	FHL2	2	105357712	105438513	-1
ENSG00000119686	-2.19609569	3.609216288	0.00010835	0.00541648	FLVCR2	14	75578617	75663214	1
ENSG00000170345	-3.02139677	6.131075147	3.58E-06	0.00049746	FOS	14	75278774	75282230	1
ENSG00000125740	-4.91784987	0.545861888	0.00014913	0.00672185	FOSB	19	45467995	45475179	1
ENSG00000111206	2.742403777	3.394718124	0.00023077	0.00899367	FOXM1	12	2857681	2877155	-1
ENSG00000053254	1.514785847	5.868857338	0.00018919	0.00779372	FOXN3	14	89124871	89619149	-1
ENSG00000100350	1.817256167	3.540113279	8.27E-05	0.00449769	FOXRED2	22	36487190	36507101	-1
ENSG00000183090	-8.02528964	-1.839209675	0.00019462	0.00794041	FREM3	4	143577302	143700675	-1
ENSG00000130383	-8.5172783	-0.344833082	1.59E-08	9.02E-06	FUT5	19	5865826	5870540	-1
ENSG00000129245	-1.45815453	6.313926083	7.38E-07	0.00015277	FXR2	17	7591230	7614871	-1
ENSG00000157240	-2.27651034	4.87220357	0.00012126	0.00587163	FZD1	7	91264364	91271326	1
ENSG00000141349	1.516591566	5.011770258	0.00011464	0.00563136	G6PC3	17	44070735	44076344	1
ENSG00000099860	-2.26290176	3.517477303	0.00026691	0.0099893	GADD45B	19	2476122	2478259	1
ENSG00000185340	1.690427378	4.228045083	0.00020864	0.00838135	GAS2L1	22	29306582	29312785	1
ENSG00000130283	3.057149765	2.13113686	8.04E-08	2.87E-05	GDF1	19	18868545	18896096	-1
ENSG00000164949	-2.83629104	6.093384966	7.39E-08	2.73E-05	GEM	8	94249253	94262350	-1
ENSG00000198380	-1.15618117	7.315628599	0.00023424	0.00909766	GFPT1	2	69319769	69387254	-1
ENSG00000138604	-1.29897918	5.547006869	0.0002196	0.00863775	GLCE	15	69160584	69272217	1
ENSG00000074047	3.06137469	1.911911388	7.57E-07	0.00015485	GLI2	2	120735623	120992653	1
ENSG00000065325	-6.84203546	-1.614563761	0.00017638	0.00743751	GLP2R	17	9822206	9892102	1
ENSG00000134697	-1.17462934	6.182138566	0.00010718	0.00537383	GNL2	1	37566816	37595935	-1
ENSG00000130119	1.457849632	4.481831178	5.37E-05	0.00329555	GNL3L	X	54530211	54561071	1
ENSG00000111711	-1.41836632	6.243292148	5.88E-06	0.0007061	GOLT1B	12	21501781	21518408	1
ENSG00000152208	2.078920186	5.609443028	0.00012906	0.00606772	GRID2	4	92303622	93774556	1

ENSG00000171189	1.410812466	5.628320155	4.76E-05	0.00303238	GRIK1	21	29536933	29940033	-1
ENSG00000109519	-1.15673548	5.165000191	6.86E-05	0.0039178	GRPEL1	4	7058906	7068197	-1
ENSG00000137947	-1.56930888	5.126177731	1.67E-05	0.00145893	GTF2B	1	88852932	88891944	-1
ENSG00000125812	-1.34306804	5.337951767	0.00012593	0.0059955	GZF1	20	23362182	23373063	1
ENSG00000132475	-1.15475316	9.198621302	3.29E-06	0.00047103	H3F3B	17	75776434	75785893	-1
ENSG00000188375	-9.35723204	1.316069564	4.24E-09	3.51E-06	H3F3C	12	31791185	31792241	-1
ENSG00000063854	-1.34499027	5.140519307	0.0001557	0.00688199	HAGH	16	1795620	1827194	-1
ENSG00000103044	-3.10739989	2.4045056	0.00012791	0.00605553	HAS3	16	69105564	69118719	1
ENSG00000113070	-3.01131798	5.214624364	0.00014039	0.00645746	HBEGF	5	140332843	140346631	-1
ENSG00000172534	2.173588374	4.422691812	0.00018495	0.00772003	HCFC1	X	153947553	153971807	-1
ENSG00000108840	-1.47942637	5.044302612	8.61E-05	0.00461885	HDAC5	17	44076746	44123702	-1
ENSG00000119969	1.777722709	4.744569487	1.95E-05	0.00164709	HELLS	10	94501434	94613905	1
ENSG00000165478	2.245251328	5.297683204	9.33E-05	0.00489682	HEPACAM	11	124919193	124936412	-1
ENSG00000051108	-1.69218227	6.538008819	4.04E-06	0.00054398	HERPUD1	16	56932048	56944863	1
ENSG00000177374	-3.82497699	1.073253342	1.47E-05	0.00133803	HIC1	17	2054154	2063241	1
ENSG00000064393	1.993153699	6.721978169	1.38E-05	0.00129343	HIPK2	7	139561570	139777778	-1
ENSG00000276903	2.565333105	2.588717964	0.0001144	0.0056282	HIST1H2AL	6	27865355	27865747	1
ENSG00000170144	1.491748759	6.694731971	8.25E-05	0.0044961	HNRNPA3	2	177212563	177223958	1
ENSG00000105323	1.559483572	6.525202414	3.22E-05	0.00233695	HNRNPUL1	19	41262496	41307598	1
ENSG00000152413	-2.46492465	5.43160967	1.45E-07	4.73E-05	HOMER1	5	79372636	79514217	-1
ENSG00000134709	-2.71142384	2.899131662	4.86E-06	0.00062005	HOOK1	1	59814786	59876378	1
ENSG00000257017	-5.66076164	5.467698387	2.25E-05	0.0018486	HP	16	72054592	72061055	1
ENSG00000166189	-1.41525806	4.00329844	0.00015884	0.00693964	HPS6	10	102065390	102068038	1
ENSG00000102878	-4.03320197	0.812078997	3.35E-05	0.00238041	HSF4	16	67164681	67169945	1
ENSG00000106211	2.069228644	3.271859312	0.00026201	0.00981679	HSPB1	7	76302544	76304295	1
ENSG00000086758	1.358460632	8.196181528	0.00015857	0.00693964	HUWE1	X	53532096	53686729	-1
ENSG00000068001	-1.40529516	5.581848179	0.00025134	0.00955387	HYAL2	3	50317790	50322906	-1
ENSG00000105376	-4.79464077	-0.078460229	1.40E-05	0.00129343	ICAM5	19	10289981	10296778	1
ENSG00000125968	-7.30965818	2.792491849	1.01E-05	0.00105155	ID1	20	31605283	31606515	1
ENSG00000115738	-2.56257989	6.528157348	9.24E-05	0.00485715	ID2	2	8678845	8684453	1
ENSG00000117318	-7.09077778	2.304687587	1.42E-05	0.00129733	ID3	1	23557918	23559794	-1
ENSG00000010404	1.767427932	7.420639745	8.96E-05	0.00474235	IDS	X	149476990	149521096	-1
ENSG00000137331	-4.11605617	-0.116805466	2.95E-06	0.00042734	IER3	6	30743199	30744554	-1
ENSG00000134049	-0.97818234	5.620019925	0.00022976	0.00897441	IER3IP1	18	47152998	47176374	-1
ENSG00000017427	-5.9068136	1.296953989	1.40E-05	0.00129343	IGF1	12	102395867	102480645	-1
ENSG00000159217	1.890558385	3.921673694	0.00021532	0.00854065	IGF2BP1	17	48997412	49055650	1
ENSG00000146674	-3.82439349	5.430971409	2.78E-07	7.48E-05	IGFBP3	7	45912245	45921874	-1
ENSG00000141753	-3.1712335	5.655942907	1.10E-05	0.00110192	IGFBP4	17	40443461	40457731	1
ENSG00000123411	2.086249249	1.985795465	7.54E-05	0.00424242	IKZF4	12	56007659	56038435	1
ENSG00000095752	-5.84370557	4.525540108	1.39E-09	1.48E-06	IL11	19	55364389	55370463	-1
ENSG00000115008	-4.67722257	-0.444811074	2.81E-05	0.00215999	IL1A	2	112773915	112784590	-1
ENSG00000139269	-8.30880392	-0.001162428	2.78E-05	0.00214881	INHBE	12	57452323	57459280	1
ENSG00000127080	-1.74806872	4.741255773	1.67E-05	0.00145893	IPPK	9	92613184	92670265	-1
ENSG00000126456	-1.50352636	4.947145701	3.33E-05	0.00237152	IRF3	19	49659569	49665875	-1
ENSG00000063241	-1.41175261	3.845029601	0.00017807	0.00749966	ISOC2	19	55452985	55462343	-1
ENSG00000123104	1.177638702	8.34927576	4.24E-05	0.00277542	ITPR2	12	26336515	26833198	-1
ENSG00000081692	-2.19496727	4.722943878	0.00010463	0.00529669	JMJD4	1	227730425	227735411	-1
ENSG00000171223	-3.39945454	3.461049978	0.00016899	0.00721021	JUNB	19	12791496	12793315	1
ENSG00000186994	-3.51112663	0.241720465	1.03E-05	0.0010646	KANK3	19	8322584	8343262	-1
ENSG00000132854	-4.91402242	0.535239883	5.28E-05	0.00327854	KANK4	1	62236979	62319414	-1
ENSG00000107821	-3.20728536	1.803094983	1.56E-05	0.00139392	KAZALD1	10	101061841	101068131	1
ENSG00000182255	-3.98695401	3.987678844	9.22E-06	0.00099012	KCNA4	11	30009741	30017023	-1
ENSG00000152049	-5.41903719	4.911773659	5.31E-08	2.16E-05	KCNE4	2	223051814	223198399	1
ENSG00000162975	-2.87070618	4.835595192	7.98E-05	0.00441293	KCNF1	2	10911937	10914225	1
ENSG00000177807	1.743318258	3.792576151	1.05E-05	0.0010721	KCNJ10	1	160037467	160070248	-1
ENSG00000124249	-8.86291478	-1.635222015	8.45E-05	0.00456227	KCNK15	20	44745780	44752313	1
ENSG00000143603	2.484524994	6.399417611	1.15E-05	0.00113967	KCNN3	1	154697455	154870280	-1
ENSG00000100196	-2.88294561	4.307270058	9.87E-07	0.00018517	KDELR3	22	38468062	38483447	1
ENSG00000166783	1.982313244	4.349340043	3.26E-05	0.00233695	KIAA0430	16	15594386	15643166	-1
ENSG00000100578	1.336521153	5.067540341	4.65E-05	0.0029706	KIAA0586	14	58427385	58551289	1
ENSG00000186185	2.505089299	2.313012922	0.00020731	0.00836511	KIF18B	17	44924709	44947711	-1
ENSG00000155090	-4.18157766	4.836430542	1.31E-06	0.00022977	KLF10	8	102648779	102655902	-1
ENSG00000118922	1.413175346	6.318967689	6.32E-05	0.00370673	KLF12	13	73686089	74133905	-1
ENSG00000109787	1.695730415	5.681356265	4.59E-05	0.00294558	KLF3	4	38664196	38701042	1
ENSG00000198841	-2.13479727	3.399760417	2.56E-05	0.00202231	KTI12	1	52032103	52033816	-1
ENSG00000131023	1.570653785	5.39659299	0.00014648	0.00662965	LATS1	6	149658153	149718256	-1
ENSG00000204381	-3.05841167	3.386424509	0.00013312	0.00620696	LAYN	11	111540280	111561745	1
ENSG00000168806	-1.94433065	4.578901244	6.07E-05	0.00359113	LCMT2	15	43323649	43330605	-1
ENSG00000179241	1.468471938	5.426171319	3.12E-05	0.00230997	LDLRAD3	11	35943981	36232136	1
ENSG00000105617	-2.08818194	0.996353435	0.00012821	0.00605776	LENG1	19	54155161	54159882	-1
ENSG00000116678	-1.90008272	3.542200403	2.03E-06	0.0003289	LEPR	1	65420652	65641559	1
ENSG00000128342	-5.2604163	3.672557436	6.16E-10	8.29E-07	LIF	22	30240447	30246851	-1
ENSG00000160789	-1.93346438	6.865071882	0.00021305	0.00849887	LMNA	1	156082573	156140089	1
ENSG00000147650	-1.20053277	5.789800405	0.00021307	0.00849887	LRP12	8	104489231	104589024	-1
ENSG00000137269	-1.42814017	3.340284454	0.00025999	0.0097626	LRRC1	6	53794497	53924121	1
ENSG00000171017	-2.92153668	1.955668614	7.83E-07	0.00015731	LRRC8E	19	7888505	7902021	1
ENSG00000133800	5.877576911	-0.833070442	9.58E-05	0.00496392	LYVE1	11	10556966	10611689	-1
ENSG00000124688	-1.30724973	4.476429677	0.00021024	0.00843023	MAD2L1BP	6	43629540	43640952	1
ENSG00000178573	-1.29848391	4.725195677	0.00025617	0.00965125	MAF	16	79585843	79600714	-1
ENSG00000185022	-3.8746162	4.759718361	0.00015362	0.00683759	MAFF	22	38200767	38216511	1
ENSG00000198042	-1.42753957	5.38855014	0.00012831	0.00605776	MAK16	8	33484750	33501260	1

ENSG00000166963	2.182288515	7.338060072	2.42E-05	0.00194391	MAP1A	15	43510958	43531620	1
ENSG00000168175	1.229825148	6.374797731	8.45E-05	0.00456227	MAPK1IP1L	14	55051631	55070192	1
ENSG00000166603	-3.02867101	1.759825548	8.93E-06	0.00097836	MC4R	18	60371110	60372775	-1
ENSG00000128285	-3.08545537	3.554483127	9.46E-07	0.00018031	MCHR1	22	40678750	40682814	1
ENSG00000137337	2.163474395	1.392086492	0.00016064	0.00698207	MDC1	6	30699807	30717889	-1
ENSG00000130772	-1.32789956	3.427520908	0.00024518	0.00941805	MED18	1	28329002	28335967	1
ENSG00000159479	-1.27876504	4.784326246	5.37E-05	0.00329555	MED8	1	43383917	43389808	-1
ENSG00000197614	-5.25770941	1.128999911	0.00026138	0.00980387	MFAP5	12	8637346	8662888	-1
ENSG00000168282	-1.60848125	5.795975297	2.30E-06	0.00036157	MGAT2	14	49620795	49623481	1
ENSG00000008394	2.197397001	6.254232942	1.41E-06	0.00024549	MGST1	12	16347142	16609259	1
ENSG00000148773	3.239150817	5.790455073	1.66E-05	0.00145247	MKI67	10	128096659	128126385	-1
ENSG00000196549	-6.99295658	2.940942471	2.56E-07	7.04E-05	MME	3	155024124	155183729	1
ENSG00000166670	-4.48452004	1.729904568	1.66E-06	0.00027877	MMP10	11	102770503	102780628	-1
ENSG00000115275	-1.20552891	5.266500301	0.0002413	0.00930772	MOGS	2	74461057	74465410	-1
ENSG00000123562	-0.95357428	8.513411243	0.00022151	0.00869247	MORF4L2	X	103675496	103688158	-1
ENSG00000106330	1.898094033	1.649845716	6.05E-05	0.00358759	MOSPD3	7	100612102	100615384	1
ENSG00000134042	3.251427351	3.192156201	5.86E-09	4.32E-06	MRO	18	50795120	50825402	-1
ENSG00000180992	-1.57895257	4.389274862	1.38E-05	0.00129343	MRPL14	6	44113454	44127457	-1
ENSG00000149792	-1.42892034	6.082068531	3.81E-06	0.00052091	MRPL49	11	65122183	65127371	1
ENSG00000183617	-1.38946856	3.639489571	0.00024904	0.00948795	MRPL54	19	3762664	3768575	1
ENSG00000125445	-1.1422854	5.617903736	7.76E-05	0.00433154	MRPS7	17	75261674	75266373	1
ENSG00000198899	1.933128921	6.387264215	5.19E-05	0.00323831	MT-ATP6	MT	8527	9207	1
ENSG00000198804	1.904501523	10.65560654	8.31E-05	0.00451148	MT-CO1	MT	5904	7445	1
ENSG00000198712	2.073428106	8.428428078	2.05E-06	0.00033041	MT-CO2	MT	7586	8269	1
ENSG00000198938	2.719280324	9.927588903	3.44E-07	8.79E-05	MT-CO3	MT	9207	9990	1
ENSG00000198886	2.285916951	7.503272563	2.85E-05	0.00217732	MT-ND4	MT	10760	12137	1
ENSG00000212907	2.277970041	4.537491705	1.90E-05	0.0016151	MT-ND4L	MT	10470	10766	1
ENSG00000242114	-3.88426566	1.062594791	4.30E-06	0.0005617	MTFP1	22	30425530	30429053	1
ENSG00000065911	-2.83787849	5.484605682	0.00012743	0.00604972	MTHFD2	2	74198562	74217565	1
ENSG00000256045	-10.9130676	-0.286914849	9.72E-06	0.00102748	MTRNR2L10	X	55181391	55182920	-1
ENSG00000255823	-7.29708937	-0.45795975	4.06E-06	0.00054522	MTRNR2L8	11	10507887	10509189	-1
ENSG00000179820	2.055829836	3.599112849	2.10E-05	0.00175307	MYADM	19	53866223	53876437	1
ENSG00000138347	-6.7635639	-1.129679754	9.83E-05	0.00506575	MYPN	10	68106117	68212017	1
ENSG00000138386	-1.69796144	6.928091398	1.48E-05	0.00133952	NAB1	2	190646746	190692766	1
ENSG00000166886	-3.8086387	6.244569362	2.77E-08	1.38E-05	NAB2	12	57088894	57095476	1
ENSG00000253506	-7.10904062	-0.255816936	7.82E-09	5.08E-06	NACA2	17	61590426	61591202	-1
ENSG00000188613	-2.12659369	2.508065309	0.00019352	0.00791454	NANOS1	10	119029716	119033732	1
ENSG00000095380	-1.41453924	4.682386711	3.37E-05	0.00238504	NANS	9	98056739	98083075	1
ENSG00000166833	2.140522013	5.561078449	4.81E-06	0.00061806	NAV2	11	19350724	20121598	1
ENSG00000176771	2.351896837	4.005827119	0.00012772	0.00605504	NCKAP5	2	132671799	133568463	-1
ENSG00000131507	1.665043672	7.509093845	5.20E-06	0.00064704	NDFIP1	5	142108505	142154443	1
ENSG00000070614	1.472042757	6.012448843	5.41E-05	0.00330642	NDST1	5	150485818	150558211	1
ENSG00000196712	1.356949613	7.370046821	4.81E-05	0.0030547	NF1	17	31094927	31382116	1
ENSG00000162599	2.149309499	8.147739538	4.30E-05	0.00280619	NFIA	1	60865259	61462793	1
ENSG00000008441	2.484092085	4.167389969	1.73E-05	0.00149413	NFIX	19	12995608	13098796	1
ENSG00000163293	-4.56839555	0.010539215	6.44E-05	0.00374578	NIPAL1	4	47914142	48040173	1
ENSG00000196338	1.685624047	4.869668443	8.54E-05	0.00459351	NLGN3	X	71144831	71171201	1
ENSG00000197696	-1.60472422	3.530652885	7.87E-05	0.00436451	NMB	15	84655129	84658563	-1
ENSG00000167207	-3.31767047	0.837425688	0.00015896	0.00693964	NOD2	16	50693603	50733077	1
ENSG00000111641	-1.25856792	5.097285419	0.00015926	0.00693964	NOP2	12	6556863	6568691	-1
ENSG00000197893	-6.64613673	-0.383084477	9.72E-05	0.00502222	NRAP	10	113588716	113664127	-1
ENSG00000137404	3.486097001	-0.492784149	7.70E-05	0.00431577	NRM	6	30688047	30691420	-1
ENSG00000198400	-9.99181236	-0.057261167	3.24E-05	0.00233695	NTRK1	1	156815640	156881850	1
ENSG00000120526	-1.30343671	5.024782104	0.00021404	0.00852776	NUDCD1	8	109240919	109334385	-1
ENSG00000136159	-1.43332267	3.797808279	3.89E-05	0.00263676	NUDT15	13	48037567	48047222	1
ENSG00000126883	1.896314926	4.84672007	9.26E-06	0.00099171	NUP214	9	131125561	131234670	1
ENSG00000104904	-0.97794197	7.956639036	8.15E-05	0.0044757	OAZ1	19	2269509	2273490	1
ENSG00000184221	6.545950184	1.720486702	6.13E-07	0.00013407	OLIG1	21	33070144	33072420	1
ENSG00000072682	-3.44856228	4.374085453	2.64E-05	0.00205761	P4HA2	5	132191838	132295315	-1
ENSG00000165912	-1.68040785	3.956672635	1.63E-05	0.00143762	PACSIN3	11	47177525	47186443	-1
ENSG00000180370	1.418621756	6.243794312	3.61E-05	0.00251287	PAK2	3	196739857	196832647	1
ENSG00000132570	1.395293007	3.454684066	0.00013723	0.00635488	PCBD2	5	134904906	135007959	1
ENSG00000280165	-2.52154474	3.195631231	0.00018364	0.00767682	PCDH20	13	61409685	61415522	-1
ENSG00000171815	2.766502359	1.885473828	8.56E-05	0.0045979	PCDHB1	5	141051135	141059344	1
ENSG00000240184	3.281044059	4.172172857	0.00012072	0.00586239	PCDHGC3	5	141475947	141512979	1
ENSG00000126226	-1.2726798	5.598907529	5.84E-06	0.00070535	PCID2	13	113177539	113208715	-1
ENSG00000102230	1.604600696	5.553946828	0.00017626	0.00743751	PCYT1B	X	24558087	24672677	-1
ENSG00000005882	1.577701226	4.163452611	7.65E-05	0.00430042	PDK2	17	50094737	50112152	1
ENSG00000179889	-1.52315469	5.59330175	3.23E-05	0.00233695	PDXDC1	16	14974591	15139339	1
ENSG00000162734	1.087326797	9.070553306	8.82E-05	0.00469395	PEA15	1	160205337	160215376	1
ENSG00000152684	-1.41615009	4.932374915	0.00014479	0.00657076	PELO	5	52787940	52804046	1
ENSG00000226784	-6.09461593	-1.38889734	8.43E-05	0.00456227	PGAM4	X	77968874	77969638	-1
ENSG00000243251	-7.2723137	-0.234884493	9.40E-05	0.00490504	PGBD3	10	49515105	49524227	-1
ENSG00000013375	-1.11320424	7.711906633	0.00016068	0.00698207	PGM3	6	83161150	83193936	-1
ENSG00000204138	2.305030757	4.43887893	2.32E-05	0.00187929	PHACTR4	1	28369582	28500369	1
ENSG00000109118	1.91268476	4.391225623	4.81E-05	0.0030547	PHF12	17	28905250	28951771	-1
ENSG00000174307	-2.36425816	5.149481922	7.10E-06	0.00081962	PHLDA3	1	201464383	201469237	-1
ENSG00000198355	-2.24460802	2.985518488	0.00025403	0.00960713	PIM3	22	49960513	49964080	1
ENSG00000087842	2.212336005	5.094754405	4.09E-05	0.00269748	PIR	X	15384799	15493564	-1
ENSG00000197181	-3.60086316	-0.102234364	1.17E-05	0.00114891	PIWIL2	8	22275297	22357563	1
ENSG00000162878	-3.10259411	3.212613213	2.42E-05	0.00194391	PKDCC	2	42048020	42058528	1

ENSG00000103066	-1.55872676	4.67715187	1.88E-05	0.00160535	PLA2G15	16	68245304	68261062	1
ENSG00000145632	-2.10453864	6.67558345	2.16E-05	0.0017873	PLK2	5	58453982	58460260	-1
ENSG00000173846	-2.96630221	3.086990399	0.0002075	0.00836511	PLK3	1	44800225	44805990	1
ENSG00000198753	2.808394041	2.332424369	1.32E-09	1.45E-06	PLXNB3	X	153764196	153779346	1
ENSG00000141682	-3.11249641	4.170559633	8.75E-07	0.00017077	PMAIP1	18	59899948	59904306	1
ENSG00000124225	-2.03610994	5.909600276	4.03E-05	0.00266904	PMEPA1	20	57648392	57711536	-1
ENSG00000102978	-1.36422481	6.290083909	4.94E-06	0.00062837	POLR2C	16	57462387	57472010	1
ENSG00000163882	-1.2565548	4.994729242	1.33E-05	0.00127185	POLR2H	3	184361718	184368596	1
ENSG00000104356	-1.60999823	3.766861816	6.65E-05	0.00381969	POP1	8	98117297	98159834	1
ENSG00000077157	1.459657205	6.045516823	0.00012473	0.00598008	PPP1R12B	1	202348699	202592706	1
ENSG00000027075	-8.61777255	-1.48250452	4.42E-05	0.00284957	PRKCH	14	61187559	61550976	1
ENSG00000253729	1.250429435	8.849845544	0.00015861	0.00693964	PRKDC	8	47773108	47960183	-1
ENSG00000172179	-9.97799476	-1.435334666	5.52E-06	0.0006791	PRL	6	22287244	22297501	-1
ENSG00000126457	-1.13970293	6.88721548	0.00016819	0.00721021	PRMT1	19	49675786	49689029	1
ENSG00000117707	1.533438159	4.79791808	0.00012188	0.00589367	PROX1	1	213983181	214041502	1
ENSG00000101911	-2.44757558	5.110762377	0.00015508	0.0068724	PRPS2	X	12791355	12824222	1
ENSG00000131467	1.307000044	5.1379558	3.08E-05	0.00230095	PSME3	17	42824385	42843758	1
ENSG00000073756	-7.78867239	1.310720215	1.42E-05	0.00129733	PTGS2	1	186671791	186680427	-1
ENSG00000087494	-4.9767337	1.667737001	1.16E-06	0.00020768	PTHLH	12	27958084	27972705	-1
ENSG00000153707	1.415317664	7.784981017	9.42E-05	0.00490504	PTPRD	9	8314246	10612723	-1
ENSG00000060656	-3.58737224	1.928764615	0.00024448	0.00940905	PTPRU	1	29236516	29326813	1
ENSG00000106278	1.722299691	9.546350196	5.92E-06	0.00070756	PTPRZ1	7	121873089	122062036	1
ENSG00000116260	-1.82696595	4.60666702	2.62E-05	0.00204894	QSOX1	1	180154834	180204030	1
ENSG00000156675	-2.9129198	5.532052676	3.25E-05	0.00233695	RAB11FIP1	8	37858618	37899467	-1
ENSG00000117280	-1.50030096	5.13828877	2.86E-05	0.00218032	RAB29	1	205767986	205775460	-1
ENSG00000172007	-1.10310221	4.990111023	9.46E-05	0.00491265	RAB33B	4	139453232	139476609	1
ENSG00000075785	1.388901187	6.073198611	4.98E-05	0.00314168	RAB7A	3	128726122	128814796	1
ENSG00000103710	-6.41280922	-0.31635466	0.00020812	0.00837564	RASL12	15	65053337	65076690	-1
ENSG00000068028	-1.51489044	4.322005324	4.85E-06	0.00062005	RASSF1	3	50329782	50340980	-1
ENSG00000076067	1.745224259	4.314372802	0.00013507	0.00627224	RBMS2	12	56521929	56596196	1
ENSG00000173653	-1.68581841	2.937442952	6.93E-05	0.00394914	RCE1	11	66842835	66846546	1
ENSG00000054967	-2.33348769	2.982079757	0.00017624	0.00743751	RELT	11	73376264	73397474	1
ENSG00000182175	2.321520388	6.052036305	0.00018883	0.00779372	RGMA	15	93035273	93089204	-1
ENSG00000153165	-9.10640659	0.000417695	6.20E-08	2.39E-05	RGPD3	2	106391290	106468376	-1
ENSG00000183054	-10.03785	-1.410500157	1.97E-06	0.00032139	RGPD6	2	110513812	110577185	-1
ENSG00000116741	-3.14593582	4.390126315	1.57E-05	0.00139945	RGS2	1	192809039	192812283	1
ENSG00000117152	-5.38253684	3.791370948	3.03E-05	0.00227447	RGS4	1	163068775	163076802	1
ENSG00000124784	-1.70186304	4.736193495	9.96E-05	0.00509994	RIOK1	6	7389496	7418037	1
ENSG00000125352	-1.57393612	2.883876983	6.62E-05	0.00381969	RNF113A	X	119870475	119871827	-1
ENSG00000188050	-3.48180195	-0.246135988	0.0002465	0.00943333	RNF133	7	122697712	122699156	-1
ENSG00000163481	-1.44327135	4.781325103	1.06E-05	0.0010761	RNF25	2	218663864	218672411	-1
ENSG00000181852	-1.42771503	6.452198456	9.76E-06	0.00102922	RNF41	12	56202175	56221933	-1
ENSG00000135119	1.480423512	5.034988052	8.91E-05	0.00472787	RNFT2	12	116738178	116853631	1
ENSG00000100316	-1.15545963	9.783016765	6.09E-05	0.00359638	RPL3	22	39312882	39320389	-1
ENSG00000122406	-1.04125953	9.173072626	5.36E-05	0.00329555	RPL5	1	92832025	92841924	1
ENSG00000137818	2.439547719	7.383458521	0.00017095	0.00725355	RPLP1	15	69452784	69456194	1
ENSG00000163902	-1.00062239	8.529793392	0.00019622	0.00797685	RPN1	3	128619970	128681075	-1
ENSG00000163125	2.116045925	4.893564883	2.53E-05	0.00201141	RPRD2	1	150363091	150476566	1
ENSG00000142534	-1.02053995	9.260388923	0.00011093	0.00548981	RPS11	19	49496365	49499689	1
ENSG00000185088	-1.40228701	7.05629005	9.10E-06	0.00098343	RPS27L	15	63125872	63158021	-1
ENSG00000149273	-1.2978532	8.460387391	2.98E-05	0.00224972	RPS3	11	75399486	75422280	1
ENSG00000166133	-1.49096142	3.704663724	0.00014169	0.00649096	RPUSD2	15	40569300	40574943	1
ENSG00000048392	-1.47649267	7.668626701	3.94E-06	0.00053256	RRM2B	8	102204502	102239118	-1
ENSG00000198838	2.57957853	5.770734907	5.69E-05	0.00340453	RYR3	15	33310945	33866121	1
ENSG00000170989	-1.6713098	6.693864173	0.00021477	0.00854065	S1PR1	1	101236888	101241518	1
ENSG00000165821	2.168438811	6.494602644	1.29E-05	0.00124017	SALL2	14	21521081	21537216	-1
ENSG00000136715	1.654025165	4.118319048	0.00012571	0.00599332	SAP130	2	127941217	128028120	-1
ENSG00000168077	1.809650698	6.458527773	2.32E-05	0.00187929	SCARA3	8	27633868	27676776	1
ENSG00000168079	-10.0789129	-1.375023118	5.06E-06	0.00063743	SCARA5	8	27869882	27992727	-1
ENSG00000162512	1.995879812	8.029561004	5.22E-05	0.00324328	SDC3	1	30869467	30908761	-1
ENSG00000073578	-0.99654748	6.432324065	0.00023001	0.00897441	SDHA	5	218241	256700	1
ENSG00000100934	-1.50803418	7.460183546	2.22E-06	0.00035389	SEC23A	14	39031919	39109646	-1
ENSG00000075223	-2.87487658	4.236890905	3.72E-05	0.00256199	SEMA3C	7	80742538	80922359	-1
ENSG00000010319	-6.4009627	-0.499987153	1.25E-07	4.21E-05	SEMA3G	3	52433053	52445085	-1
ENSG00000119231	-1.16216911	6.75523747	9.90E-05	0.00508219	SENP5	3	196867856	196934714	1
ENSG00000106366	-4.01516667	4.332359459	0.00015023	0.00673672	SERPINE1	7	101127089	101139266	1
ENSG00000149131	2.363434827	4.439053952	7.80E-05	0.00434508	SERPING1	11	57597387	57614853	1
ENSG00000197019	-4.02162425	4.647554378	3.23E-09	2.81E-06	SERTAD1	19	40421592	40426025	-1
ENSG00000152217	2.381554772	5.83263294	3.12E-05	0.00230997	SETBP1	18	44680173	45068510	1
ENSG00000230667	-6.69721703	0.213961652	3.62E-08	1.66E-05	SETSIP	1	92074533	92075441	-1
ENSG00000143368	1.955980053	3.715871414	3.97E-05	0.00265504	SF3B4	1	149923317	149928344	-1
ENSG00000118515	-4.27985649	3.238568702	5.57E-05	0.00337068	SGK1	6	134169246	134318112	-1
ENSG00000164023	-2.95875624	4.976230492	3.59E-05	0.00250827	SGMS2	4	107824563	107915047	1
ENSG00000172985	2.064991897	3.380765727	0.00012662	0.00601975	SH3RF3	2	109129348	109504632	1
ENSG00000197555	1.266960262	6.89859982	5.59E-05	0.00337447	SIPA1L1	14	71320449	71741229	1
ENSG00000165480	2.346185081	2.911943328	0.00016396	0.007081	SKA3	13	21153595	21176602	-1
ENSG00000141526	-6.42971842	0.509690668	3.89E-05	0.00263676	SLC16A3	17	82228397	82261129	1
ENSG00000119899	-1.534484	4.882943162	0.00020641	0.00834132	SLC17A5	6	73593379	73654155	-1
ENSG00000162383	-10.7187525	-1.259442171	5.06E-07	0.00011611	SLC1A7	1	53087179	53142632	-1
ENSG00000089057	1.271938719	5.853034296	0.00020825	0.00837564	SLC23A2	20	4852356	5010293	-1
ENSG00000164933	-2.24703441	4.504771934	7.04E-05	0.00400219	SLC25A32	8	103398635	103415189	-1

ENSG00000170385	-2.0533362	7.002632531	2.88E-06	0.00042189	SLC30A1	1	211571568	211578742	-1
ENSG00000176087	1.510628784	4.328388738	5.51E-05	0.00333982	SLC35A4	5	140564456	140569103	1
ENSG00000157593	-1.33131051	5.940178556	4.56E-05	0.0029329	SLC35B2	6	44254096	44257890	-1
ENSG00000157800	-1.25097845	6.406915095	0.00011715	0.00572981	SLC37A3	7	140293693	140404433	-1
ENSG00000104635	-1.70120088	6.23792486	5.45E-06	0.00067553	SLC39A14	8	22367249	22434129	1
ENSG00000168003	-1.54556912	7.374443281	4.65E-05	0.0029706	SLC3A2	11	62856102	62888875	1
ENSG00000131389	-2.34271896	4.40121635	1.44E-06	0.00024792	SLC6A6	3	14402576	14489349	1
ENSG00000170365	2.090074356	5.464078598	2.27E-05	0.00185339	SMAD1	4	145481194	145558079	1
ENSG00000101665	-2.14737991	2.768727841	2.86E-05	0.00218032	SMAD7	18	48919853	48950711	-1
ENSG00000072501	1.363443298	6.780377388	2.76E-05	0.00214034	SMC1A	X	53374149	53422728	-1
ENSG00000167447	-1.07067633	5.704574779	0.00018275	0.0076587	SMG8	17	59209400	59215247	1
ENSG00000256235	-1.77033499	4.003651885	1.48E-05	0.00133952	SMIM3	5	150777946	150796734	1
ENSG00000077312	1.671741528	4.165105743	9.08E-05	0.00478764	SNRPA	19	40750637	40765389	1
ENSG00000171150	-1.14013317	5.791052316	0.00024554	0.00941805	SOCS5	2	46698952	46763129	1
ENSG00000177732	2.206978072	3.657749776	4.23E-05	0.00277416	SOX12	20	325401	330224	1
ENSG00000134532	1.948017089	6.351748515	0.00023641	0.00916105	SOX5	12	23529500	23951032	-1
ENSG00000135899	2.765602517	1.420635123	2.13E-05	0.00176029	SP110	2	230167293	230225729	-1
ENSG00000158792	-3.34652672	3.532737754	9.19E-08	3.25E-05	SPATA2L	16	89696343	89701705	-1
ENSG00000152253	2.297568506	1.261890104	0.00019547	0.00795576	SPC25	2	168834132	168913371	-1
ENSG00000072195	1.319487693	5.056220556	0.00016239	0.00703093	SPEG	2	219434846	219498287	1
ENSG00000176170	-4.9808298	3.255663801	1.72E-07	5.41E-05	SPHK1	17	76376584	76387860	1
ENSG00000063176	1.566165183	3.478851582	1.37E-05	0.00129343	SPHK2	19	48619291	48630717	1
ENSG00000166145	-6.20168795	-1.498426022	0.00016936	0.00721021	SPINT1	15	40844018	40858207	1
ENSG00000203772	2.330677961	2.912491759	7.64E-06	0.00087131	SPRN	10	133420666	133424572	-1
ENSG00000179119	-1.6023308	6.326291754	5.77E-05	0.00344157	SPTY2D1	11	18606401	18634791	-1
ENSG00000196935	2.267223361	5.057415562	8.22E-06	0.00092659	SRGAP1	12	63844293	64162221	1
ENSG00000101638	2.825870215	3.008765149	6.47E-05	0.00374988	ST8SIA5	18	46667821	46759257	-1
ENSG00000138378	-6.03500083	1.713545108	3.57E-05	0.00250557	STAT4	2	191029576	191151596	-1
ENSG00000159167	-5.31605663	5.269528412	8.70E-10	1.06E-06	STC1	8	23841915	23854807	-1
ENSG00000164543	-1.87736919	5.390200924	9.21E-05	0.00484901	STK17A	7	43582758	43626786	1
ENSG00000165416	1.093176984	7.298816364	6.38E-05	0.00372571	SUGT1	13	52652709	52700909	1
ENSG00000078269	-2.53142334	3.756377909	0.00024689	0.00943775	SYNJ2	6	157981887	158099176	1
ENSG00000157625	1.142482562	4.981406958	0.0001389	0.0064022	TAB3	X	30827442	30975084	-1
ENSG00000120948	1.486265975	7.210814171	0.00013274	0.00619774	TARDBP	1	11012344	11026420	1
ENSG00000113407	-1.38721761	7.677930352	1.37E-05	0.00129343	TARS	5	33440696	33469539	1
ENSG00000212127	-6.21192098	-0.760508886	9.35E-06	0.00099787	TAS2R14	12	10937406	11171573	-1
ENSG00000104946	2.040296642	2.906725637	0.00014395	0.00655909	TBC1D17	19	49877425	49888749	1
ENSG00000106638	-1.3392945	5.037975489	0.00012458	0.00598008	TBL2	7	73568932	73578791	-1
ENSG00000109927	2.072255178	2.007606753	0.00010296	0.00523965	TECTA	11	121101173	121191493	1
ENSG00000162851	-1.27426313	3.98281665	0.00010925	0.00542955	TFB2M	1	246540560	246566324	-1
ENSG00000105825	-2.19192121	10.45951111	5.51E-05	0.00333982	TFPI2	7	93885397	93890991	-1
ENSG00000184436	-1.84274559	4.252633059	3.94E-05	0.00265504	THAP7	22	20999104	21002196	-1
ENSG00000137801	-3.63572423	8.520912674	1.45E-06	0.00024792	THBS1	15	39581079	39599466	1
ENSG00000140534	2.559934869	2.64336246	1.23E-05	0.00119877	TICRR	15	89575482	89631056	1
ENSG00000171914	1.830767639	5.76810808	0.00012017	0.0058525	TLN2	15	62390526	62844631	1
ENSG00000136869	-3.0519208	5.487077242	5.34E-05	0.00329555	TLR4	9	117704332	117716871	1
ENSG00000086598	-1.02024627	8.221307532	0.00014131	0.0064822	TMED2	12	123584531	123598577	1
ENSG00000075568	1.499528882	5.32154546	6.52E-05	0.00377216	TMEM131	2	97756333	97995891	-1
ENSG00000164112	-3.03636617	1.476920812	0.00012318	0.00593952	TMEM155	4	121758933	121765427	-1
ENSG00000168701	-1.32412145	4.394306314	0.00019926	0.00808118	TMEM208	16	67227103	67229278	1
ENSG00000072954	-2.04231443	3.448338199	3.14E-05	0.0023122	TMEM38A	19	16661127	16690029	1
ENSG00000120889	-1.49595302	6.394080942	8.22E-05	0.00448861	TNFRSF10B	8	23020133	23069179	-1
ENSG00000006327	-5.95615589	6.084876982	5.03E-09	3.80E-06	TNFRSF12A	16	3018445	3022383	1
ENSG00000000005	-6.1174929	1.706510637	7.81E-05	0.00434593	TNMD	X	100584802	100599885	1
ENSG00000159173	-7.15970613	-0.769397837	8.28E-06	0.00092922	TNNI1	1	201403768	201429866	-1
ENSG00000182095	1.795599203	5.026765717	1.62E-05	0.00142605	TNRC18	7	5306790	5425414	-1
ENSG00000100354	1.351992399	7.530498879	0.00021955	0.00863775	TNRC6B	22	40044817	40335808	1
ENSG00000132773	-1.9110782	3.528197309	0.00016166	0.00700852	TOE1	1	45339670	45343975	1
ENSG00000136816	-1.27341214	5.003982872	1.59E-05	0.0014106	TOR1B	9	129803153	129811281	1
ENSG00000101255	-2.54310345	4.408619413	5.79E-06	0.00070264	TRIB3	20	362835	397559	1
ENSG00000173113	-1.26575335	6.189536868	2.56E-05	0.00202286	TRMT112	11	64316460	64318084	-1
ENSG00000100154	1.355509773	5.800218427	0.00016326	0.00705988	TTC28	22	27978014	28679865	-1
ENSG00000117143	-1.49017031	5.498146549	8.80E-06	0.0009731	UAP1	1	162561506	162599842	1
ENSG00000150991	-1.15777505	10.12023517	3.81E-05	0.00260886	UBC	12	124911604	124917368	-1
ENSG00000078967	1.774354119	3.997427392	0.00015369	0.00683759	UBE2D4	7	43926438	43956136	1
ENSG00000130725	-1.71466681	5.015563946	0.00011127	0.00548981	UBE2M	19	58555712	58558960	-1
ENSG00000244687	1.988983693	5.487930261	1.15E-05	0.00114021	UBE2V1	20	50081124	50115959	-1
ENSG00000130939	1.313660702	5.370765772	0.00020338	0.00822868	UBE4B	1	10032832	10181239	1
ENSG00000164332	1.292362646	5.01540675	0.00021613	0.00854065	UBLCP1	5	159263081	159286040	1
ENSG00000059145	1.416256239	3.550078908	0.00018595	0.00774313	UNKL	16	1363205	1414751	-1
ENSG00000147679	-1.37394062	5.113142571	2.38E-05	0.00191928	UTP23	8	116766503	116849463	1
ENSG00000162738	1.802010954	6.239615719	0.00015558	0.00688199	VANGL2	1	160400586	160428678	1
ENSG00000170162	-8.96440839	-1.179536984	4.13E-06	0.00054941	VGLL2	6	117265558	117273565	1
ENSG00000131871	-1.18818036	5.976787901	0.00018723	0.00775073	VIMP	15	101270817	101277500	-1
ENSG00000132549	1.429269267	7.079373882	0.0001055	0.00529771	VPS13B	8	99013266	99877580	1
ENSG00000134258	-3.8988538	-0.121560742	0.00011095	0.00548981	VTCN1	1	117143587	117210960	-1
ENSG00000084463	1.821114065	5.578693239	6.00E-06	0.00071261	WBP11	12	14784579	14803540	-1
ENSG00000139668	1.067596885	6.280012005	0.00023148	0.00901086	WDFY2	13	51584455	51767707	1
ENSG00000163625	1.216410991	8.14717875	0.00025966	0.0097626	WDFY3	4	84669610	84966391	-1
ENSG00000130733	-1.56489513	4.951795849	1.92E-05	0.00162926	YIPF2	19	10922185	10928681	-1
ENSG00000108953	1.820987461	7.459818873	1.40E-05	0.00129343	YWHAE	17	1344272	1400378	-1

ENSG00000132846	2.752236654	1.894153689	0.00012451	0.00598008	ZBED3	5	77072072	77087323	-1
ENSG00000181722	1.232954823	5.790657588	0.00012497	0.00598322	ZBTB20	3	114338094	115147271	-1
ENSG00000168826	-1.55268941	3.201014875	0.00016915	0.00721021	ZBTB49	4	4290251	4321786	1
ENSG00000178951	2.073137208	2.658497165	1.68E-05	0.00146565	ZBTB7A	19	4044364	4066945	-1
ENSG00000123200	1.828441163	6.863099064	0.00014873	0.00671369	ZC3H13	13	45954465	46052759	-1
ENSG00000169946	2.224387019	2.53617114	1.86E-05	0.00159465	ZFPM2	8	104590733	105804532	1
ENSG00000072121	1.423537875	6.087016948	5.17E-06	0.00064588	ZFYVE26	14	67727374	67816590	-1
ENSG00000155256	-2.2741084	5.435230079	7.41E-09	4.94E-06	ZFYVE27	10	97737121	97760907	1
ENSG00000160094	1.563808346	3.903498028	3.27E-05	0.00233913	ZNF362	1	33256545	33300719	1
ENSG00000126746	1.433818158	4.84057562	0.00013374	0.00622737	ZNF384	12	6666477	6689572	-1
ENSG00000204604	-1.56011156	4.832209405	6.66E-05	0.00381969	ZNF468	19	52838008	52857649	-1
ENSG00000074657	1.280747204	7.461574253	5.13E-05	0.00321396	ZNF532	18	58862600	58986480	1
ENSG00000196357	-1.44497179	3.179208723	6.37E-05	0.00372571	ZNF565	19	36182060	36246257	-1
ENSG00000164684	1.372474467	5.651904787	3.68E-05	0.00254744	ZNF704	8	80628451	80874781	-1
ENSG00000151612	1.718010616	4.907036524	1.35E-05	0.00128518	ZNF827	4	145757627	145938635	-1
ENSG00000132801	-1.50858037	2.965491009	0.00010488	0.00529669	ZSWIM3	20	45857617	45879122	1

Significantly altered genes between control and THC (chronic) treated iPSC-derived neurons.

Ensembl_gene_id	logFC	logCPM	PValue	FDR	hgnc_symbol	chromosome	start position	end position	strand
ENSG00000160179	-2.2488807	5.997429796	6.64E-06	0.00059421	ABCG1	21	42199689	42297244	1
ENSG00000136379	1.655839123	3.396538392	0.00034581	0.00935339	ABHD17C	15	80679684	80755621	1
ENSG00000100439	2.272354989	5.135091444	0.00022195	0.00689294	ABHD4	14	22598237	22613215	1
ENSG00000146109	-1.34164144	4.517272084	8.13E-05	0.00350464	ABT1	6	26596952	26600744	1
ENSG00000014257	-7.54107618	-1.54540333	8.24E-05	0.00353867	ACPP	3	132317367	132368298	1
ENSG00000107796	-2.32771356	8.970239561	8.62E-05	0.00361584	ACTA2	10	88935074	88991390	-1
ENSG00000154734	-3.69236107	5.347865131	5.04E-05	0.00249017	ADAMTS1	21	26835747	26845409	-1
ENSG00000129467	-6.79335852	-1.571050763	2.58E-05	0.0015655	ADCY4	14	24318349	24335093	-1
ENSG00000078549	1.831775448	6.378219798	0.00021163	0.0066834	ADCYAP1R1	7	31052461	31111479	1
ENSG00000116863	-1.44541959	5.252789003	2.60E-05	0.00156997	ADPRHL2	1	36088875	36093932	1
ENSG00000181026	-1.85272041	6.132771524	0.00035683	0.00955464	AEN	15	88621296	88632282	1
ENSG00000144218	1.406570121	7.102603747	2.81E-05	0.00165602	AFF3	2	99545419	100142739	-1
ENSG00000092847	1.29811499	6.834515835	9.28E-05	0.00382844	AGO1	1	35869808	35930528	1
ENSG00000126705	2.674137628	3.452013963	2.69E-05	0.00160412	AHDC1	1	27534035	27604431	-1
ENSG00000059573	-1.30275139	6.708504603	0.00010632	0.00418187	ALDH18A1	10	95605929	95656706	-1
ENSG00000184254	-10.0747854	0.707051955	2.31E-06	0.00027955	ALDH1A3	15	100877714	100916626	1
ENSG00000178038	-2.98615034	0.279192905	0.00037562	0.00988392	ALS2CL	3	46668997	46693704	-1
ENSG00000123505	-1.62846609	7.522791073	1.68E-05	0.00117212	AMD1	6	110814621	110895713	1
ENSG00000136938	2.096029909	3.511917446	5.41E-05	0.0026139	ANP32B	9	97983361	98015943	1
ENSG00000169604	2.471105089	6.248975281	1.03E-05	0.00081374	ANTXR1	2	69013178	69249327	1
ENSG00000264230	-4.96221986	-0.866766224	4.91E-06	0.00047938	ANXA8L1	10	46375627	46537864	1
ENSG00000183020	1.32958793	4.823772984	0.00027852	0.00809396	AP2A2	11	924894	1012245	1
ENSG00000006125	1.524498942	8.331576658	2.01E-05	0.00133381	AP2B1	17	35578046	35726409	1
ENSG00000134982	1.44890066	9.264868086	6.63E-05	0.00300712	APC	5	112707498	112846239	1
ENSG00000244509	2.299221954	2.784880164	7.71E-06	0.00065784	APOBEC3C	22	39014083	39020352	1
ENSG00000109321	-7.41665463	-1.655630195	0.00017624	0.00590567	AREG	4	74445134	74455009	1
ENSG00000168374	-1.7268289	7.748531487	3.02E-06	0.00034214	ARF4	3	57571363	57598220	-1
ENSG00000241484	-6.74307397	-0.486394105	0.00020647	0.00656355	ARHGAP8	22	44752558	44862788	1
ENSG00000175906	-2.39171727	1.856503946	0.00014725	0.00519565	ARL4D	17	43398959	43401137	1
ENSG00000242498	1.38739665	5.806499602	9.68E-05	0.00392908	ARPIN	15	89895006	89912956	-1
ENSG00000180801	-2.16413294	5.092245047	4.44E-05	0.00229152	ARSJ	4	113900284	113979727	-1
ENSG00000153317	1.851113061	4.890277877	7.75E-06	0.00065822	ASAP1	8	130052104	130443660	-1
ENSG00000110881	1.824164139	4.372228159	0.00011024	0.0043063	ASIC1	12	50057548	50083611	1
ENSG00000162772	-2.84208178	4.100110753	6.36E-05	0.00292456	ATF3	1	212565334	212620777	1
ENSG00000128272	-1.19860872	8.583576392	0.00025585	0.00766538	ATF4	22	39519695	39522685	1
ENSG00000170653	2.140828958	3.284127878	2.58E-05	0.00156669	ATF7	12	53507856	53626410	-1
ENSG00000171681	2.057884455	7.824670977	1.76E-05	0.00121164	ATF7IP	12	14365632	14502935	1
ENSG00000123395	-1.5802088	5.134100158	7.76E-06	0.00065822	ATG101	12	52069246	52077494	1
ENSG00000058668	1.987427352	7.35084324	0.00011408	0.0043906	ATP2B4	1	203626561	203744081	1
ENSG00000117410	-1.48503221	6.234005492	2.98E-05	0.00172833	ATP6V0B	1	43974487	43978295	1
ENSG00000143515	-1.44448052	5.549407885	0.00033088	0.00909445	ATP8B2	1	154325553	154351307	1
ENSG00000183778	1.937651181	3.808794102	1.36E-05	0.00099987	B3GALT5	21	39556442	39673137	1
ENSG00000123810	-1.55697126	2.998290229	0.00020524	0.00656355	B9D2	19	41354421	41364173	-1
ENSG00000186318	1.490735635	5.454826492	0.00031382	0.00884021	BACE1	11	117285207	117316259	-1
ENSG00000266074	2.694187674	2.275766652	5.93E-06	0.00054245	BAHCC1	17	81395475	81466332	1
ENSG00000076108	1.782338088	5.09338512	0.00013365	0.00484175	BAZ2A	12	56595596	56636816	-1
ENSG00000258643	2.168683282	3.774967539	1.10E-05	0.00085245	BCL2L2-PABPN1	14	23306835	23325369	1
ENSG00000116128	1.56050973	3.795118043	0.00026333	0.00782027	BCL9	1	147541412	147626216	1
ENSG00000168398	-3.57521721	-0.029107091	0.00031413	0.00884182	BDKRB2	14	96204679	96244166	1
ENSG00000176697	-3.31467252	4.716966021	1.83E-05	0.00124407	BDNF	11	27654893	27722058	-1
ENSG00000123095	1.457996995	5.705394601	0.00021977	0.00684871	BHLHE41	12	26120026	26125127	-1
ENSG00000117475	-1.28229975	5.58007944	0.00010631	0.00418187	BLZF1	1	169367970	169396540	1
ENSG00000125378	-3.34299595	2.089552973	2.33E-05	0.0014782	BMP4	14	53949736	53958761	-1
ENSG00000169594	-10.1721964	0.718516128	4.74E-07	8.47E-05	BNC1	15	83255903	83284716	-1
ENSG00000163141	3.768881052	0.306652473	0.00023235	0.00711822	BNIPL	1	151036570	151047600	1
ENSG00000171634	1.35748677	7.594654465	1.92E-05	0.00128428	BPTF	17	67825524	67984378	1
ENSG00000104221	-1.23660933	4.363816977	0.00010984	0.00429538	BRF2	8	37843268	37849904	-1
ENSG00000174744	-1.81033664	4.981349918	5.99E-07	0.00010108	BRMS1	11	66337333	66345125	-1
ENSG00000133243	1.258727567	5.106078467	0.00019839	0.00643216	BTBD2	19	1985438	2034881	-1
ENSG00000166167	1.224106272	6.113200555	5.71E-05	0.0026985	BTRC	10	101354033	101557321	1

ENSG00000137656	-1.01512615	4.729239289	0.00019726	0.00640267	BUD13	11	116748170	116772988	-1
ENSG00000173088	3.984967301	-1.110261662	0.0001173	0.00447374	C10orf131	10	95907603	95938723	1
ENSG00000103544	1.162789761	5.988212709	0.00015834	0.00547319	C16orf62	16	19555240	19706793	1
ENSG00000174109	-1.35120146	3.048732977	8.63E-05	0.00361584	C16orf91	16	1419744	1420800	-1
ENSG00000239887	2.183700086	3.178660614	0.00012595	0.00467136	C1orf226	1	162366908	162386818	1
ENSG00000162757	-1.59012019	2.801233188	0.00033791	0.00920237	C1orf74	1	209779208	209784559	-1
ENSG00000184208	1.798386351	4.617715987	1.51E-05	0.0010872	C22orf46	22	41688939	41698136	1
ENSG00000178776	-7.26608997	-1.581154373	4.36E-05	0.00227577	C5orf46	5	147880726	147906538	-1
ENSG00000203872	2.1412555	0.82178387	0.00032737	0.00904929	C6orf163	6	87344849	87365463	1
ENSG00000153790	3.016414937	2.16531769	3.84E-06	0.00040873	C7orf31	7	25134697	25180356	-1
ENSG00000105605	2.10394451	3.461360566	4.55E-06	0.00045567	CACNG7	19	53909335	53943941	1
ENSG00000112186	1.443570367	5.003458437	0.00020361	0.00654429	CAP2	6	17393216	17557792	1
ENSG00000132357	3.705882864	-0.560186246	0.00021992	0.00684871	CARD6	5	40841184	40860175	1
ENSG00000177303	1.606226065	2.987353758	0.00014494	0.00514115	CASKIN2	17	75500261	75515583	-1
ENSG00000106144	2.14699269	3.832153993	8.99E-06	0.00072827	CASP2	7	143288215	143307696	1
ENSG00000067955	-1.32805732	5.674017042	3.69E-05	0.00198949	CBFB	16	67029116	67101058	1
ENSG00000110395	2.200707852	6.152916062	4.72E-07	8.47E-05	CBL	11	119206276	119308149	1
ENSG00000094916	1.69813213	9.256712441	2.09E-06	0.00026198	CBX5	12	54230940	54280133	-1
ENSG00000183741	1.05204044	6.841818094	0.00023778	0.00724759	CBX6	22	38861450	38872314	-1
ENSG00000007080	1.671902089	3.517847885	0.00019969	0.00645572	CCDC124	19	17933016	17943991	1
ENSG00000177352	-1.6267426	3.608950005	0.00017145	0.00580251	CCDC71	3	49162535	49166321	-1
ENSG00000169515	-1.7752508	5.188046762	0.00033133	0.00909936	CCDC8	19	46410372	46413584	-1
ENSG00000115355	1.703006114	8.0772405	0.00011124	0.00432525	CCDC88A	2	55287842	55419921	-1
ENSG00000172156	-11.7585793	-0.920047342	1.80E-05	0.00122742	CCL11	17	34285668	34288334	1
ENSG00000100814	-1.36131178	5.535003948	3.68E-05	0.00198949	CCNB1IP1	14	20311368	20333312	-1
ENSG00000112576	-2.26738003	5.205687999	2.46E-05	0.0015186	CCND3	6	41934933	42050357	-1
ENSG00000085117	-2.19688893	2.220962502	9.59E-06	0.00076029	CD82	11	44564427	44620363	1
ENSG00000179604	1.221759992	5.947362343	0.00032794	0.00905772	CDC42EP4	17	73283624	73312175	-1
ENSG00000040731	1.971652694	3.863466363	0.00023433	0.00717254	CDH10	5	24487100	24644978	-1
ENSG00000124762	-1.92221738	6.758797041	8.31E-06	0.00068343	CDKN1A	6	36676460	36687339	1
ENSG00000140743	-2.40841571	4.306373817	5.21E-06	0.00049805	CDR2	16	22345936	22437165	-1
ENSG00000113722	-9.83487112	-1.491002092	1.56E-05	0.00110989	CDX1	5	150166795	150184558	1
ENSG00000245848	-4.83296316	1.506866743	5.24E-07	9.04E-05	CEBPA	19	33299934	33302564	-1
ENSG00000153879	-0.96064838	6.771100064	0.00033554	0.00916312	CEBPG	19	33373330	33382686	1
ENSG00000099954	2.176295464	4.084354276	1.84E-05	0.0012492	CECR2	22	17359949	17558149	1
ENSG00000048740	1.640528277	6.380695923	2.83E-05	0.00165976	CELF2	10	11005321	11336675	1
ENSG00000223802	1.216436796	5.634343872	1.91E-05	0.00128428	CERS1	19	18868545	18896727	-1
ENSG00000138135	-6.0604066	0.813848965	0.00010848	0.00425687	CH25H	10	89205629	89207314	-1
ENSG00000198824	1.516973596	3.295337868	0.0002757	0.00804697	CHAMP1	13	114314513	114327328	1
ENSG00000171316	1.866630203	7.136992552	4.88E-06	0.00047743	CHD7	8	60678778	60868028	1
ENSG00000133048	5.272552308	-0.0855488	0.00035968	0.00959926	CHI3L1	1	203178931	203186749	-1
ENSG00000174343	-2.87775374	1.863500495	0.00026359	0.00782132	CHRNA9	4	40335329	40355217	1
ENSG00000213341	-1.12033245	6.146408084	0.00031995	0.00893885	CHUK	10	100188298	100229619	-1
ENSG00000005194	-1.09662641	5.711726512	0.00013104	0.00480318	CIAPIN1	16	57428169	57447528	-1
ENSG00000185043	-1.23475789	5.103367423	0.00010313	0.00411203	CIB1	15	90229975	90234047	-1
ENSG00000175505	-7.34448615	2.750227796	4.19E-10	5.08E-07	CLCF1	11	67364168	67374177	-1
ENSG00000182372	-2.07458336	1.36839799	5.36E-05	0.00259874	CLN8	8	1755778	1786572	1
ENSG00000205423	-1.23640128	4.977347113	2.88E-05	0.00168405	CNEP1R1	16	50024410	50037088	1
ENSG00000148842	1.729020357	4.156324561	3.12E-05	0.00177299	CNNM2	10	102918293	103090221	1
ENSG00000137161	1.412985577	4.780228075	6.98E-05	0.00313033	CNPY3	6	42929192	42939287	1
ENSG00000136152	-1.11379137	6.201055456	4.52E-05	0.00231993	COG3	13	45464898	45536630	1
ENSG00000133103	-1.11373845	6.200708431	0.0001778	0.00594036	COG6	13	39655627	39791665	1
ENSG00000204291	-8.23605336	0.70468951	5.08E-06	0.0004899	COL15A1	9	98943179	99070792	1
ENSG00000101203	3.861715439	-0.636458953	0.0001054	0.00416982	COL20A1	20	63293186	63334851	1
ENSG00000050767	-3.87612895	1.588654313	0.00027506	0.0080422	COL23A1	5	178237618	178590555	-1
ENSG00000080573	-4.25570411	4.985447071	9.50E-05	0.00388936	COL5A3	19	9959561	10010471	-1
ENSG00000129083	-1.43827169	8.166996322	3.73E-06	0.00040359	COPB1	11	14443440	14500027	-1
ENSG00000115520	-2.01814186	5.625102204	1.89E-06	0.00023994	COQ10B	2	197453423	197475308	1
ENSG00000103647	3.185655418	4.136437548	7.55E-05	0.0033289	CORO2B	15	68578969	68727806	1
ENSG00000112695	-1.57974166	6.297261301	2.86E-07	5.93E-05	COX7A2	6	75237675	75250323	-1
ENSG00000109625	-5.51041861	-1.105891179	3.06E-05	0.00175391	CPZ	4	8592660	8619759	1
ENSG00000143320	-3.49093592	3.074196642	0.00012858	0.00474405	CRABP2	1	156699606	156705816	-1
ENSG00000107175	-1.32856929	6.041241525	1.33E-06	0.00018217	CREB3	9	35732320	35737007	1
ENSG00000006016	3.409764625	3.74777982	3.20E-07	6.59E-05	CRLF1	19	18572220	18607741	-1
ENSG00000176390	-1.17770918	3.951531314	0.00036191	0.00964281	CRLF3	17	30769388	30824776	-1
ENSG00000140577	1.384047082	5.083305626	0.00013723	0.00495097	CRTC3	15	90529925	90645345	1
ENSG00000062485	1.702612611	6.211282708	0.00029393	0.00838344	CS	12	56271699	56300392	-1
ENSG00000070770	0.952772362	5.360707574	0.00024846	0.00749689	CSNK2A2	16	58157907	58197920	-1
ENSG00000144655	-2.47750402	5.184234624	9.74E-05	0.00394008	CSRNP1	3	39141855	39154562	-1
ENSG00000101138	-1.0351978	5.581403569	0.00026072	0.00776173	CSTF1	20	56392371	56406369	1
ENSG00000175215	1.925631497	6.584629159	1.07E-05	0.00083728	CTDSP2	12	57819927	57846739	-1
ENSG00000119326	-1.54589407	5.584700961	0.00020684	0.00656355	CTNNAL1	9	108942569	109013529	-1
ENSG00000064601	1.295565664	5.122813296	0.00012424	0.00463654	CTSA	20	45890144	45898820	1
ENSG00000103811	-2.93669207	4.08695683	0.00025541	0.00765902	CTSH	15	78921058	78949574	-1
ENSG00000180891	1.241830017	5.922072132	0.00016841	0.00574375	CUEDC1	17	57861243	57955323	-1
ENSG00000169429	-4.56816486	-0.798133808	0.00010506	0.00416603	CXCL8	4	73740506	73743716	1
ENSG00000166394	-3.78815863	-0.358530741	3.14E-05	0.00177684	CYB5R2	11	7665100	7677222	-1
ENSG00000100243	1.732839706	4.996203604	2.57E-05	0.00156101	CYB5R3	22	42617840	42649568	-1
ENSG00000179091	-1.18889808	5.764053646	5.83E-05	0.0027395	CYC1	8	144095027	144097525	1
ENSG00000105443	-1.40429478	6.822611283	0.00014911	0.0052505	CYTH2	19	48469032	48482314	1
ENSG00000129562	-1.04405883	6.859085401	4.30E-05	0.00225789	DAD1	14	22564905	22589269	-1

ENSG00000173402	1.252972187	7.360142027	0.00027945	0.00809396	DAG1	3	49468703	49535618	1
ENSG00000136485	1.783300495	6.981807686	4.62E-07	8.35E-05	DCAF7	17	63550461	63594266	1
ENSG00000167986	-0.88963267	9.066884553	0.00031635	0.00888198	DDB1	11	61299451	61342596	-1
ENSG00000134574	-1.65818298	4.604915871	0.00034883	0.00941264	DDB2	11	47214465	47239240	1
ENSG00000175197	-1.88884669	4.93268177	0.0001319	0.00481674	DDIT3	12	57516588	57520517	-1
ENSG00000182810	-2.00260372	3.636564636	7.94E-05	0.0034531	DDX28	16	68021274	68023442	-1
ENSG00000184047	-1.06892797	6.472988534	0.00027737	0.00808862	DIABLO	12	122207662	122227534	-1
ENSG00000165023	-2.0693599	7.080962029	1.57E-06	0.00020351	DIRAS2	9	90609832	90643104	-1
ENSG00000107984	-4.37966848	2.352375497	0.00028311	0.00815571	DKK1	10	52314296	52318042	1
ENSG00000050165	-1.8320225	10.0698347	0.00031788	0.00890305	DKK3	11	11963106	12009769	-1
ENSG00000104936	1.507996994	4.537357012	7.86E-06	0.00066351	DMPK	19	45769717	45782552	-1
ENSG00000185800	1.293505453	4.700760747	1.92E-05	0.00128428	DMWD	19	45782947	45792802	-1
ENSG00000103423	-1.0314708	4.508551428	0.00037033	0.00980479	DNAJA3	16	4425805	4456775	1
ENSG00000135392	-1.37176842	5.496769375	7.17E-05	0.00318146	DNAJC14	12	55820960	55830824	-1
ENSG00000130816	1.314915649	5.500927292	0.00022896	0.00703446	DNMT1	19	10133345	10231286	-1
ENSG00000135905	1.662772549	6.67383479	6.72E-05	0.00303733	DOCK10	2	224765090	225042445	-1
ENSG00000111817	-1.97443057	5.487341487	4.49E-06	0.00045074	DSE	6	116254173	116444860	1
ENSG00000096696	-3.83694018	4.511778525	5.72E-05	0.0026985	DSP	6	7541575	7586717	1
ENSG00000120129	-1.75199249	4.799963199	0.00036409	0.00967049	DUSP1	5	172768090	172771195	-1
ENSG00000276023	-2.15017505	2.41101814	0.00013138	0.00480554	DUSP14	17	37489831	37513501	1
ENSG00000105204	1.965098681	2.187206121	0.00034296	0.00929843	DYRK1B	19	39825350	39834201	-1
ENSG00000145194	-2.88341848	1.353649407	4.85E-06	0.00047589	ECE2	3	184249650	184293031	1
ENSG00000127129	-5.98511419	-1.506669986	0.00029917	0.00850592	EDN2	1	41478775	41484673	-1
ENSG00000159658	1.959129944	6.850885291	0.00021188	0.00668418	EFCAB14	1	46675159	46719064	-1
ENSG00000169242	-2.96665073	2.492452693	3.38E-07	6.68E-05	EFNA1	1	155127460	155134857	1
ENSG00000146648	2.83801156	4.978863659	2.84E-07	5.92E-05	EGFR	7	55019021	55256620	1
ENSG00000120738	-3.83286808	3.501963466	0.00030958	0.00874943	EGR1	5	138465490	138469315	1
ENSG00000122877	-5.34416134	2.20986425	4.40E-06	0.00044571	EGR2	10	62811996	62919900	-1
ENSG00000024422	2.310205765	2.220456855	1.39E-05	0.00102037	EHD2	19	47713343	47743134	1
ENSG00000103966	-2.57620117	3.530209537	7.11E-06	0.00062079	EHD4	15	41895939	41972578	-1
ENSG00000086232	1.017931463	6.397108925	0.00020689	0.00656355	EIF2AK1	7	6022244	6059230	-1
ENSG00000172071	-1.9436669	6.1911559	3.24E-07	6.62E-05	EIF2AK3	2	88556741	88627576	-1
ENSG00000125977	-1.0160362	6.769911191	0.00032137	0.00895366	EIF2S2	20	34088298	34112332	-1
ENSG00000149100	-0.86247186	7.4851187	0.00022275	0.00691168	EIF3M	11	32583798	32606262	1
ENSG00000148730	1.647886981	6.534875967	9.99E-07	0.00014515	EIF4EBP2	10	70404379	70428618	1
ENSG00000158417	1.871607522	5.798555796	0.00012208	0.00459939	EIF5B	2	99337353	99401326	1
ENSG00000242372	-1.26802418	5.593493109	2.62E-05	0.00157627	EIF6	20	35278907	35284985	-1
ENSG00000132205	-4.52994656	1.30162427	6.15E-05	0.00286963	EMILIN2	18	2847030	2915993	1
ENSG00000134531	-3.11829322	7.083612685	3.81E-07	7.36E-05	EMP1	12	13196716	13219939	1
ENSG00000100393	1.857477807	5.543869087	4.72E-05	0.00237768	EP300	22	41091786	41180079	1
ENSG00000086289	1.356613378	5.517862968	0.00013085	0.00480318	EPDR1	7	37683843	37951941	1
ENSG00000142627	-3.03340197	3.236182694	4.16E-05	0.00220399	EPHA2	1	16124337	16156087	-1
ENSG00000116106	-1.5605402	5.744883926	0.00027556	0.00804697	EPHA4	2	221418027	221574202	-1
ENSG00000143819	2.968225323	4.879749349	3.23E-08	1.12E-05	EPHX1	1	225810092	225845563	1
ENSG00000063245	2.085472963	3.954092936	4.17E-06	0.00043604	EPN1	19	55675226	55709858	1
ENSG00000082805	1.448434866	6.625043031	2.47E-05	0.00152253	ERC1	12	990509	1495933	1
ENSG00000124882	-8.38365278	-0.542882529	3.00E-05	0.00173217	EREG	4	74365143	74388751	1
ENSG00000115363	-2.96729948	4.602192499	8.95E-05	0.00370678	EVA1A	2	75469302	75569722	-1
ENSG00000072840	1.421700294	3.878738607	9.76E-05	0.00394348	EVC	4	5711197	5814305	1
ENSG00000182197	-2.25541903	6.137220128	6.65E-06	0.00059421	EXT1	8	117794490	118111853	-1
ENSG00000168040	-1.87871252	4.572728357	5.29E-06	0.00050275	FADD	11	70203163	70207390	1
ENSG00000184731	-2.5780933	3.219948094	2.42E-05	0.00150818	FAM110C	2	38814	46870	-1
ENSG00000048828	1.341637458	6.439713467	0.00033205	0.0091045	FAM120A	9	93451722	93566107	1
ENSG00000175182	-1.65215512	6.116488376	0.00020378	0.00654429	FAM131A	3	184335926	184346275	1
ENSG00000189292	-2.59742193	1.192339412	3.95E-05	0.00210698	FAM150B	2	279558	288851	-1
ENSG00000170264	1.248733171	4.68921851	0.00023027	0.00706741	FAM161A	2	61824854	61854143	-1
ENSG00000163322	1.668780392	3.882880507	1.88E-05	0.0012709	FAM175A	4	83459517	83523348	-1
ENSG00000135063	1.56971899	3.59864259	0.0001965	0.00639108	FAM189A2	9	69324572	69392455	1
ENSG00000164125	2.085724503	6.376637239	5.20E-05	0.00254336	FAM198B	4	158124474	158173318	-1
ENSG00000185614	-4.06513554	-0.569596941	0.00018021	0.00600313	FAM212A	3	49803254	49805030	1
ENSG00000157870	1.828180948	1.446245656	0.00019916	0.00644481	FAM213B	1	2586491	2591469	1
ENSG00000173065	2.173739601	2.140895862	6.61E-05	0.00300448	FAM222B	17	28755978	28855232	-1
ENSG00000158246	-2.51804971	3.569520685	1.00E-05	0.00079125	FAM46B	1	27005020	27012836	-1
ENSG00000166595	-1.37147503	5.283341797	8.32E-06	0.00068343	FAM96B	16	66932055	66934423	-1
ENSG00000165323	1.556848588	8.294316082	3.95E-05	0.00210698	FAT3	11	92352096	92896470	1
ENSG00000105202	-1.16433733	4.907253878	0.00014879	0.00524485	FBL	19	39834458	39846414	-1
ENSG00000156860	1.409182198	4.127602511	0.00013005	0.00479326	FBRS	16	30658431	30670814	1
ENSG00000108306	1.619627709	5.360220378	0.00011975	0.00455688	FBXL20	17	39252644	39402523	-1
ENSG00000118496	0.967774178	6.870229038	0.00017605	0.00590529	FBXO30	6	145793502	145814753	-1
ENSG00000115641	-2.66725278	5.077366105	0.0001401	0.00501588	FHL2	2	105357712	105438513	-1
ENSG00000151702	-3.49789455	0.373744654	2.85E-06	0.00032862	FLI1	11	128686535	128813267	1
ENSG00000119686	-2.22100482	3.609216288	8.30E-05	0.0035472	FLVCR2	14	75578617	75663214	1
ENSG00000170345	-2.84816151	6.131075147	1.14E-05	0.00087697	FOS	14	75278774	75282230	1
ENSG00000129514	-8.66736401	1.916592621	0.00028048	0.00810969	FOXA1	14	37589984	37596059	-1
ENSG00000164916	1.462389067	5.357609201	0.00011183	0.00433357	FOXK1	7	4682309	4771443	1
ENSG00000111206	3.144500082	3.394718124	2.37E-05	0.00149947	FOXM1	12	2857681	2877155	-1
ENSG00000053254	2.017186257	5.868857338	6.26E-07	0.00010366	FOXN3	14	89124871	89619149	-1
ENSG00000100350	2.297676933	3.540113279	5.30E-07	9.09E-05	FOXRED2	22	36487190	36507101	-1
ENSG00000183090	-8.02528964	-1.839209675	6.67E-05	0.00301954	FREM3	4	143577302	143700675	-1
ENSG00000167996	2.523375602	8.432461333	4.98E-05	0.00247574	FTH1	11	61959718	61967660	-1
ENSG00000130383	-4.34034158	-0.344833082	2.94E-05	0.00171298	FUT5	19	5865826	5870540	-1

ENSG00000129245	-1.35389918	6.313926083	3.85E-06	0.00040873	FXR2	17	7591230	7614871	-1
ENSG00000137726	2.173637151	7.0671267	0.0002537	0.00762111	FXYD6	11	117836976	117877486	-1
ENSG00000157240	-2.59240557	4.87220357	1.31E-05	0.0009715	FZD1	7	91264364	91271326	1
ENSG00000180340	2.03535312	1.807574729	5.33E-06	0.00050549	FZD2	17	44557459	44559570	1
ENSG00000141349	1.564122947	5.011770258	6.65E-05	0.00301286	G6PC3	17	44070735	44076344	1
ENSG00000099860	-3.11708965	3.517477303	7.50E-07	0.00011841	GADD45B	19	2476122	2478259	1
ENSG00000185340	1.714049432	4.228045083	0.00016095	0.00552921	GAS2L1	22	29306582	29312785	1
ENSG00000107862	1.248154865	5.934578238	0.0001904	0.00625063	GBF1	10	102245532	102382899	1
ENSG00000130283	3.483955523	2.13113686	6.17E-10	6.75E-07	GDF1	19	18868545	18896096	-1
ENSG00000135414	1.552697411	5.068855964	9.62E-05	0.00392093	GDF11	12	55743280	55757278	1
ENSG00000164949	-2.67843772	6.093384966	3.29E-07	6.62E-05	GEM	8	94249253	94262350	-1
ENSG00000198380	-1.20595156	7.315628599	0.00012296	0.00461208	GFPT1	2	69319769	69387254	-1
ENSG00000139436	1.199710072	5.238001657	0.00011231	0.00434703	GIT2	12	109929792	109996389	-1
ENSG00000138604	-1.4575728	5.547006869	3.33E-05	0.00185595	GLCE	15	69160584	69272217	1
ENSG00000074047	3.607959627	1.911911388	3.53E-09	2.58E-06	GLI2	2	120735623	120992653	1
ENSG00000106571	1.824122732	3.215982524	5.18E-05	0.00253706	GLI3	7	41960950	42237870	-1
ENSG00000112624	1.628190865	4.390059568	3.72E-05	0.00200358	GLTSCR1L	6	42746958	42868560	1
ENSG00000134697	-1.14500607	6.182138566	0.0001504	0.00528503	GNL2	1	37566816	37595935	-1
ENSG00000130119	1.484365302	4.481831178	3.60E-05	0.00196323	GNL3L	X	54530211	54561071	1
ENSG00000135052	1.145248953	8.338056743	9.06E-05	0.00374467	GOLM1	9	86026146	86100173	-1
ENSG00000111711	-1.27908892	6.243292148	3.99E-05	0.00212407	GOLT1B	12	21501781	21518408	1
ENSG00000186566	1.480176331	5.399024588	0.00010345	0.00411203	GPATCH8	17	44395284	44503430	-1
ENSG00000170075	1.74466466	2.961841306	5.51E-05	0.00264248	GPR37L1	1	202122858	202133592	1
ENSG00000152208	2.256103602	5.609443028	3.22E-05	0.00180507	GRID2	4	92303622	93774556	1
ENSG00000171189	1.623472377	5.628320155	2.67E-06	0.0003118	GRIK1	21	29536933	29940033	-1
ENSG00000109519	-1.05580498	5.165000191	0.00024707	0.00746811	GRPEL1	4	7058906	7068197	-1
ENSG00000082701	1.269264315	6.723815839	0.00015441	0.00538684	GSK3B	3	119821323	120094417	-1
ENSG00000137947	-1.56191586	5.126177731	1.66E-05	0.00116727	GTF2B	1	88852932	88891944	-1
ENSG00000107937	-1.42320765	6.289136697	4.18E-05	0.00220514	GTPBP4	10	988019	1019936	1
ENSG00000125812	-1.326678	5.337951767	0.00014177	0.00504424	GZF1	20	23362182	23373063	1
ENSG00000184897	1.284121926	4.082998091	0.00028609	0.00820892	H1FX	3	129314771	129316277	-1
ENSG00000132475	-1.18388668	9.198621302	1.84E-06	0.00023659	H3F3B	17	75776434	75785893	-1
ENSG00000188375	-8.52691169	1.316069564	2.31E-08	8.82E-06	H3F3C	12	31791185	31792241	-1
ENSG00000138796	1.707433195	4.177384749	0.00011368	0.00438513	HADH	4	107989714	108035175	1
ENSG00000063854	-1.37834641	5.140519307	0.00010063	0.00402813	HAGH	16	1795620	1827194	-1
ENSG00000187664	3.488517439	0.402465869	3.01E-05	0.00173706	HAPLN4	19	19255641	19262796	-1
ENSG00000103044	-2.99988826	2.4045056	0.0001793	0.00597854	HAS3	16	69105564	69118719	1
ENSG00000143575	-0.99993591	5.763107424	0.0001218	0.00459906	HAX1	1	154272511	154275875	1
ENSG00000113070	-3.53984012	5.214624364	9.43E-06	0.00075178	HBEGF	5	140332843	140346631	-1
ENSG00000172534	2.434338578	4.422691812	2.74E-05	0.00162729	HCFC1	X	153947553	153971807	-1
ENSG00000165478	2.735926725	5.297683204	1.93E-06	0.00024497	HEPACAM	11	124919193	124936412	-1
ENSG00000051108	-1.54608867	6.538008819	2.33E-05	0.0014782	HERPUD1	16	56932048	56944863	1
ENSG00000177374	-4.25303389	1.073253342	1.51E-06	0.00019722	HIC1	17	2054154	2063241	1
ENSG00000064393	2.361321165	6.721978169	2.61E-07	5.65E-05	HIPK2	7	139561570	139777778	-1
ENSG00000184678	1.100025575	6.24588559	0.00033072	0.00909445	HIST2H2BE	1	149842204	149886652	-1
ENSG00000135486	1.39249474	8.897262664	0.00032399	0.00898522	HNRNPA1	12	54280193	54287088	1
ENSG00000170144	1.476496588	6.694731971	9.61E-05	0.00392093	HNRNPA3	2	177212563	177223958	1
ENSG00000096746	2.845441265	6.052152988	2.43E-05	0.00150818	HNRNPH3	10	68331174	68343191	1
ENSG00000105323	1.61005597	6.525202414	1.73E-05	0.00119523	HNRNPUL1	19	41262496	41307598	1
ENSG00000152413	-2.55950233	5.43160967	4.66E-08	1.52E-05	HOMER1	5	79372636	79514217	-1
ENSG00000134709	-2.42241641	2.899131662	3.07E-05	0.00175391	HOOK1	1	59814786	59876378	1
ENSG00000257017	-5.48334222	5.467698387	3.67E-05	0.00198781	HP	16	72054592	72061055	1
ENSG00000166189	-1.65833566	4.00329844	8.93E-06	0.00072523	HPS6	10	102065390	102068038	1
ENSG00000106211	2.170318395	3.271859312	0.00012244	0.00460787	HSPB1	7	76302544	76304295	1
ENSG00000086758	1.621398575	8.196181528	6.43E-06	0.0005808	HUWE1	X	53532096	53686729	-1
ENSG00000068001	-1.38058392	5.581848179	0.0003079	0.00872423	HYAL2	3	50317790	50322906	-1
ENSG00000105376	-3.82826835	-0.078460229	0.00020334	0.00654429	ICAM5	19	10289981	10296778	1
ENSG00000112144	1.678993159	4.725653032	0.00011086	0.00432525	ICK	6	53001279	53061802	-1
ENSG00000125968	-6.21500347	2.792491849	9.73E-05	0.00394008	ID1	20	31605283	31606515	1
ENSG00000115738	-2.44485766	6.528157348	0.00018304	0.00606759	ID2	2	8678845	8684453	1
ENSG00000117318	-6.80509349	2.304687587	2.42E-05	0.00150818	ID3	1	23557918	23559794	-1
ENSG00000010404	2.109878823	7.420639745	2.91E-06	0.00033308	IDS	X	149476990	149521096	-1
ENSG00000137331	-3.1270617	-0.116805466	8.84E-05	0.00367939	IER3	6	30743199	30744554	-1
ENSG00000134049	-1.03822932	5.620019925	8.55E-05	0.00361584	IER3IP1	18	47152998	47176374	-1
ENSG00000163565	1.742420254	5.174561145	0.00012727	0.00471123	IFI16	1	158999968	159055155	1
ENSG00000119917	2.445663611	3.014530153	9.86E-07	0.0001442	IFIT3	10	89327894	89340971	1
ENSG00000027697	-1.03184267	6.210460682	0.00014577	0.00515422	IFNGR1	6	137197484	137219449	-1
ENSG00000174498	1.720906975	3.273296738	0.00018818	0.00618702	IGDCC3	15	65327127	65378040	-1
ENSG00000017427	-5.19690782	1.296953989	7.99E-05	0.00346999	IGF1	12	102395867	102480645	-1
ENSG00000159217	1.998882931	3.921673694	8.59E-05	0.00361584	IGF2BP1	17	48997412	49055650	1
ENSG00000146674	-4.21018274	5.430971409	2.01E-08	7.83E-06	IGFBP3	7	45912245	45921874	-1
ENSG00000204866	-7.79678231	-1.823553185	0.00011108	0.00432525	IGFL2	19	46143106	46161299	1
ENSG00000263528	1.956301102	2.254484317	7.02E-05	0.00314012	IKBKE	1	206470476	206496889	1
ENSG00000095752	-5.57361743	4.525540108	5.54E-09	3.48E-06	IL11	19	55364389	55370463	-1
ENSG00000144730	1.87433389	6.209815724	7.25E-05	0.00320928	IL17RD	3	57089982	57170306	-1
ENSG00000115008	-3.96299157	-0.444811074	0.00013333	0.00483647	IL1A	2	112773915	112784590	-1
ENSG00000178035	-0.88691122	7.238684955	0.00034206	0.00928884	IMPDH2	3	49024325	49029408	-1
ENSG00000139269	-9.22007783	-0.001162428	4.34E-06	0.0004445	INHBE	12	57452323	57459280	1
ENSG00000143624	1.34058535	5.763502591	0.00014551	0.00515043	INTS3	1	153728067	153774808	1
ENSG00000127080	-1.68246224	4.741255773	3.07E-05	0.00175391	IPPK	9	92613184	92670265	-1
ENSG00000126456	-1.44753334	4.947145701	5.87E-05	0.00275364	IRF3	19	49659569	49665875	-1

ENSG00000063241	-1.60097873	3.845029601	1.92E-05	0.00128607	ISOC2	19	55452985	55462343	-1
ENSG00000132470	2.065709795	5.94734225	4.34E-05	0.0022717	ITGB4	17	75721328	75757818	1
ENSG00000162267	-7.8394808	-1.398851575	2.98E-05	0.00172804	ITIH3	3	52794768	52809009	1
ENSG00000143772	2.126254403	7.419261206	1.35E-05	0.00099677	ITPKB	1	226631690	226739323	-1
ENSG00000123104	1.214811005	8.34927576	2.38E-05	0.0015012	ITPR2	12	26336515	26833198	-1
ENSG00000081692	-2.25831689	4.722943878	6.43E-05	0.00295174	JMJD4	1	227730425	227735411	-1
ENSG00000186994	-3.05089472	0.241720465	4.30E-05	0.00225862	KANK3	19	8322584	8343262	-1
ENSG00000132854	-5.18257015	0.535239883	1.69E-05	0.00117412	KANK4	1	62236979	62319414	-1
ENSG00000156650	1.660828977	4.327836566	5.66E-05	0.00268866	KAT6B	10	74825582	75032622	1
ENSG00000107821	-2.82925121	1.803094983	8.62E-05	0.00361584	KAZALD1	10	101061841	101068131	1
ENSG00000177301	2.072584153	3.802730366	8.54E-05	0.00361584	KCNA2	1	110593580	110631474	-1
ENSG00000182255	-3.59310818	3.987678844	5.23E-05	0.00254912	KCNA4	11	30009741	30017023	-1
ENSG00000152049	-4.55339179	4.911773659	2.83E-06	0.00032799	KCNE4	2	223051814	223198399	1
ENSG00000177807	2.054296757	3.792576151	1.62E-07	3.90E-05	KCNJ10	1	160037467	160070248	-1
ENSG00000123700	-1.66956341	4.618274293	0.00021919	0.006845	KCNJ2	17	70168673	70180048	1
ENSG00000099337	-6.80958573	0.068448864	9.78E-05	0.00394764	KCNK6	19	38319844	38332076	1
ENSG00000143603	2.274287193	6.399417611	5.82E-05	0.0027395	KCNN3	1	154697455	154870280	-1
ENSG00000053918	-6.47427945	-1.544392327	0.00015588	0.00539927	KCNQ1	11	2444684	2849109	1
ENSG00000162687	1.444778523	5.250381656	0.00019097	0.00626019	KCNT2	1	196225779	196609225	-1
ENSG00000100196	-2.85799263	4.307270058	1.09E-06	0.00015406	KDELR3	22	38468062	38483447	1
ENSG00000088247	1.298349012	4.949291949	0.00018584	0.00614751	KHSRP	19	6413348	6424794	-1
ENSG00000166783	2.370602784	4.349340043	6.15E-07	0.00010235	KIAA0430	16	15594386	15643166	-1
ENSG00000121210	1.410879947	3.772193145	0.00010526	0.00416921	KIAA0922	4	153466346	153636711	1
ENSG00000164976	1.970778019	4.627180118	7.38E-06	0.00063421	KIAA1161	9	34366670	34376853	-1
ENSG00000109265	1.366294422	6.072748044	0.00033645	0.00918082	KIAA1211	4	56049073	56328625	1
ENSG00000196872	-3.26199879	0.764922503	1.14E-05	0.00087697	KIAA1211L	2	98793846	98936259	-1
ENSG00000122778	1.871290905	5.454455533	4.53E-05	0.00232289	KIAA1549	7	138831381	138981318	-1
ENSG00000130518	3.391452809	2.67088612	0.00023534	0.00719049	KIAA1683	19	18257097	18274509	-1
ENSG00000155090	-4.07083447	4.836430542	2.23E-06	0.00027347	KLF10	8	102648779	102655902	-1
ENSG00000172059	2.216094672	2.532108154	3.23E-05	0.00180653	KLF11	2	10042849	10054836	1
ENSG00000118922	1.585850507	6.318967689	6.86E-06	0.00060923	KLF12	13	73686089	74133905	-1
ENSG00000109787	1.962202875	5.681356265	2.29E-06	0.00027889	KLF3	4	38664196	38701042	1
ENSG00000205809	6.188125684	-1.349693247	5.64E-05	0.00267919	KLRC2	12	10426854	10442300	-1
ENSG00000213809	5.465821041	-1.798362488	0.00032134	0.00895366	KLRK1	12	10372353	10391874	-1
ENSG00000118058	2.041071042	6.696454042	0.0001243	0.00463654	KMT2A	11	118436490	118526832	1
ENSG00000055609	1.483666009	7.020656186	0.00010595	0.00417704	KMT2C	7	152134922	152436005	-1
ENSG00000198841	-2.24778439	3.399760417	8.28E-06	0.00068343	KTI12	1	52032103	52033816	-1
ENSG00000115919	-8.94188166	-1.223982721	0.00017249	0.00582023	KYNU	2	142877498	143055832	1
ENSG00000196976	-1.41302779	3.461420185	3.17E-05	0.00178987	LAGE3	X	154477769	154479257	-1
ENSG00000155506	1.346645802	6.750738776	0.00037073	0.00980479	LARP1	5	154712902	154817607	1
ENSG00000138709	-1.23249737	5.641217955	0.00022428	0.00694628	LARP1B	4	128061268	128222931	1
ENSG00000131023	1.631178	5.39659299	7.77E-05	0.00340182	LATS1	6	149658153	149718256	-1
ENSG00000172954	1.747046221	5.655100996	0.00034782	0.00939277	LCLAT1	2	30447226	30644225	1
ENSG00000168806	-2.08238599	4.578901244	1.72E-05	0.0011915	LCMT2	15	43323649	43330605	-1
ENSG00000179241	1.528123703	5.426171319	1.38E-05	0.00101665	LDLRAD3	11	35943981	36232136	1
ENSG00000182195	3.334329063	1.893994691	1.03E-06	0.00014769	LDOC1	X	141175745	141177125	-1
ENSG00000105617	-2.05500376	0.996353435	9.92E-05	0.00398458	LENG1	19	54155161	54159882	-1
ENSG00000167615	1.309808722	3.606211172	0.00021697	0.00679522	LENG8	19	54448887	54462037	1
ENSG00000128342	-4.74872787	3.672557436	1.26E-08	5.93E-06	LIF	22	30240447	30246851	-1
ENSG00000050405	-1.67063025	8.234021067	9.82E-05	0.00395848	LIMA1	12	50175788	50283546	-1
ENSG00000160789	-1.90835226	6.865071882	0.00025336	0.00761769	LMNA	1	156082573	156140089	1
ENSG00000134013	-3.59611937	4.2508507	0.00014037	0.00501588	LOXL2	8	23297189	23425328	-1
ENSG00000197324	1.421245796	6.026890354	0.0002918	0.00833765	LRP10	14	22871613	22881580	1
ENSG00000147650	-1.21946356	5.789800405	0.00016105	0.00552921	LRP12	8	104489231	104589024	-1
ENSG00000204052	-2.42695915	1.389659109	0.00018748	0.00617851	LRRC73	6	43506969	43510686	-1
ENSG00000171017	-2.39986553	1.955668614	2.39E-05	0.0015012	LRRC8E	19	7888505	7902021	1
ENSG00000170382	1.883725485	4.017312909	0.00014524	0.00514612	LRRN2	1	204617170	204685733	-1
ENSG00000185565	1.631459276	7.136890465	5.40E-05	0.00261135	LSAMP	3	115802363	117139389	-1
ENSG00000168056	1.841957192	4.256739549	0.00018602	0.00614751	LTBP3	11	65538560	65558930	-1
ENSG00000011009	-1.37289808	5.132999159	0.00010423	0.00413824	LYPLA2	1	23790970	23795539	1
ENSG00000133800	5.976490341	-0.833070442	3.47E-05	0.00190274	LYVE1	11	10556966	10611689	-1
ENSG00000124688	-1.25537318	4.476429677	0.00032982	0.00907997	MAD2L1BP	6	43629540	43640952	1
ENSG00000178573	-1.26461048	4.725195677	0.00033346	0.00912354	MAF	16	79585843	79600714	-1
ENSG00000185022	-4.40607708	4.759718361	2.08E-05	0.00135436	MAFF	22	38200767	38216511	1
ENSG00000198042	-1.82545122	5.38855014	1.08E-06	0.00015281	MAK16	8	33484750	33501260	1
ENSG00000184384	1.540711307	6.140561618	2.28E-05	0.00145333	MAML2	11	95976598	96343180	-1
ENSG00000013619	2.461277369	2.376946735	8.00E-06	0.00067084	MAMLD1	X	150361422	150514178	1
ENSG00000145050	-1.65728461	5.012513061	0.00026957	0.00794305	MANF	3	51385047	51389397	1
ENSG00000166963	2.540391951	7.338060072	9.08E-07	0.0001368	MAP1A	15	43510958	43531620	1
ENSG00000168175	1.398142865	6.374797731	7.42E-06	0.00063641	MAPK1IP1L	14	55051631	55070192	1
ENSG00000247626	-1.57436459	3.692415461	0.00014225	0.00505632	MARS2	2	197705369	197708387	1
ENSG00000088888	1.188940804	6.606678778	0.000132	0.00481674	MAVS	20	3846799	3876123	1
ENSG00000204406	1.911704022	5.121438467	8.39E-05	0.00357101	MBD5	2	148021011	148516971	1
ENSG00000166603	-3.26274846	1.759825548	1.42E-06	0.00019047	MC4R	18	60371110	60372775	-1
ENSG00000137337	2.133558128	1.392086492	0.00016936	0.00576048	MDC1	6	30699807	30717889	-1
ENSG00000198625	1.343971231	6.381950926	5.55E-05	0.00264926	MDM4	1	204516379	204558120	1
ENSG00000085276	-4.638038	-0.477888914	3.38E-05	0.00187483	MECOM	3	169083499	169663618	-1
ENSG00000169057	1.28391432	5.766204089	0.00016696	0.00570742	MECP2	X	154021573	154137103	-1
ENSG00000180182	1.952875624	5.262160994	0.00027868	0.00809396	MED14	X	40648306	40735858	-1
ENSG00000175221	1.64270147	3.647174157	0.00033977	0.00924164	MED16	19	867630	893218	-1
ENSG00000159479	-1.40822716	4.784326246	7.92E-06	0.00066747	MED8	1	43383917	43389808	-1

ENSG00000116604	2.001127012	3.971001295	0.00035971	0.00959926	MEF2D	1	156463727	156500828	-1
ENSG00000105429	1.547692135	6.496236851	3.37E-05	0.00187438	MEGF8	19	42325609	42378769	1
ENSG00000005102	-7.70907698	-1.89266857	0.00033767	0.00920237	MEOX1	17	43640388	43661954	-1
ENSG00000254726	1.567234806	5.340219005	0.00013491	0.00488211	MEX3A	1	156072013	156081998	-1
ENSG00000168389	-4.67786174	5.078343575	8.12E-06	0.00067558	MFSD2A	1	39955112	39969968	1
ENSG00000168282	-1.66097334	5.795975297	1.00E-06	0.00014515	MGAT2	14	49620795	49623481	1
ENSG00000008394	2.449506952	6.254232942	7.51E-08	2.28E-05	MGST1	12	16347142	16609259	1
ENSG00000271605	-7.67264315	-1.890901767	0.00015519	0.00539346	MILR1	17	64449037	64468643	1
ENSG00000148773	3.040137316	5.790455073	5.15E-05	0.00253238	MKI67	10	128096659	128126385	-1
ENSG00000186260	1.589515886	5.335544425	8.28E-05	0.00354495	MKL2	16	14071321	14266773	1
ENSG00000168288	-1.24175501	6.494254049	1.53E-05	0.00109505	MMADHC	2	149569634	149587816	-1
ENSG00000196549	-5.63897969	2.940942471	1.47E-05	0.00106482	MME	3	155024124	155183729	1
ENSG00000166670	-5.69146398	1.729904568	4.18E-09	2.84E-06	MMP10	11	102770503	102780628	-1
ENSG00000070444	1.814912653	3.190465678	5.30E-05	0.0025751	MNT	17	2384060	2401118	-1
ENSG00000124217	-1.39788262	4.012507455	0.00021386	0.00673372	MOCS3	20	50958826	50963931	1
ENSG00000123562	-1.01287893	8.513411243	8.74E-05	0.00365111	MORF4L2	X	103675496	103688158	-1
ENSG00000106330	2.003175283	1.649845716	1.58E-05	0.00112247	MOSPD3	7	100612102	100615384	1
ENSG00000134042	3.483421119	3.192156201	3.82E-10	4.98E-07	MRO	18	50795120	50825402	-1
ENSG00000180992	-1.80709622	4.389274862	6.05E-07	0.00010164	MRPL14	6	44113454	44127457	-1
ENSG00000158042	-1.17702494	5.168617289	0.0003718	0.00982131	MRPL17	11	6680782	6683401	-1
ENSG00000086504	-1.10639987	5.2307011	0.00026536	0.00784645	MRPL28	16	367384	370527	-1
ENSG00000149792	-1.45795862	6.082068531	2.26E-06	0.00027641	MRPL49	11	65122183	65127371	1
ENSG00000183617	-1.74111662	3.639489571	4.17E-06	0.00043604	MRPL54	19	3762664	3768575	1
ENSG00000204568	-1.38927007	3.589028642	0.00020547	0.00656355	MRPS18B	6	30617709	30626395	1
ENSG00000125445	-1.05282156	5.617903736	0.0002464	0.00745444	MRPS7	17	75261674	75266373	1
ENSG00000198804	2.011796935	10.65560654	3.23E-05	0.00180653	MT-CO1	MT	5904	7445	1
ENSG00000198712	2.043605488	8.428428078	2.86E-06	0.00032862	MT-CO2	MT	7586	8269	1
ENSG00000198938	2.88884148	9.927588903	6.29E-08	1.96E-05	MT-CO3	MT	9207	9990	1
ENSG00000198886	2.027349888	7.503272563	0.00020326	0.00654429	MT-ND4	MT	10760	12137	1
ENSG00000212907	1.991883623	4.537491705	0.00018039	0.00600313	MT-ND4L	MT	10470	10766	1
ENSG00000125148	2.193715983	1.227641261	0.00030099	0.0085434	MT2A	16	56608199	56609497	1
ENSG00000057935	1.025339077	5.917035223	0.0001965	0.00639108	MTA3	2	42494569	42756947	1
ENSG00000242114	-3.26955562	1.062594791	5.23E-05	0.00254835	MTFP1	22	30425530	30429053	1
ENSG00000065911	-3.04713096	5.484605682	4.12E-05	0.00218824	MTHFD2	2	74198562	74217565	1
ENSG00000139505	-1.00754168	6.257910215	0.00018448	0.00610939	MTMR6	13	25246201	25288009	-1
ENSG00000256045	-9.29501186	-0.286914849	2.83E-05	0.00166049	MTRNR2L10	X	55181391	55182920	-1
ENSG00000255823	-6.06108579	-0.45795975	2.68E-05	0.00160188	MTRNR2L8	11	10507887	10509189	-1
ENSG00000132613	1.254733742	6.353346953	0.00015283	0.00534863	MTSS1L	16	70661204	70686066	-1
ENSG00000179820	1.889934268	3.599112849	8.76E-05	0.00365316	MYADM	19	53866223	53876437	1
ENSG00000101057	3.588211922	2.730723455	4.38E-05	0.00227914	MYBL2	20	43667019	43716496	1
ENSG00000118680	-0.94802782	7.073641299	0.00028379	0.00815692	MYL12B	18	3261909	3278284	1
ENSG00000162601	1.353547109	5.509654455	8.31E-05	0.0035472	MYSM1	1	58654739	58700092	-1
ENSG00000138386	-1.65135482	6.928091398	2.43E-05	0.00150818	NAB1	2	190646746	190692766	1
ENSG00000166886	-3.86333281	6.244569362	1.77E-08	7.33E-06	NAB2	12	57088894	57095476	1
ENSG00000173559	-2.64810143	5.292852795	1.21E-05	0.0009138	NABP1	2	191678068	191696659	1
ENSG00000253506	-7.19707433	-0.255816936	1.49E-09	1.30E-06	NACA2	17	61590426	61591202	-1
ENSG00000160877	1.20019474	4.946727154	0.00028351	0.00815571	NACC1	19	13118103	13141141	1
ENSG00000095380	-1.46105875	4.682386711	1.64E-05	0.00115659	NANS	9	98056739	98083075	1
ENSG00000166833	2.567308591	5.561078449	4.06E-08	1.36E-05	NAV2	11	19350724	20121598	1
ENSG00000176771	2.867388972	4.005827119	2.89E-06	0.00033105	NCKAP5	2	132671799	133568463	-1
ENSG00000198646	1.949216651	4.230106233	3.44E-05	0.00189487	NCOA6	20	34689097	34825649	-1
ENSG00000162736	1.464219851	6.03169034	0.00030982	0.00874943	NCSTN	1	160343272	160358952	1
ENSG00000131507	1.817479105	7.509093845	6.47E-07	0.00010614	NDFIP1	5	142108505	142154443	1
ENSG00000070614	1.65302063	6.012448843	5.54E-06	0.00051693	NDST1	5	150485818	150558211	1
ENSG00000178057	-1.4325975	3.979408727	0.00028929	0.00829376	NDUFAF3	3	49020459	49023495	1
ENSG00000196712	1.456778802	7.370046821	1.26E-05	0.00093671	NF1	17	31094927	31382116	1
ENSG00000162599	2.330660683	8.147739538	9.23E-06	0.00074335	NFIA	1	60865259	61462793	1
ENSG00000141905	3.064650325	1.786548838	4.57E-05	0.0023292	NFIC	19	3359563	3469217	1
ENSG00000008441	2.880955954	4.167389969	6.08E-07	0.00010164	NFIX	19	12995608	13098796	1
ENSG00000129460	-1.10011838	5.356944836	5.06E-05	0.0024972	NGDN	14	23469688	23509862	1
ENSG00000170113	1.137472529	5.908480568	0.00017416	0.00586269	NIPA1	15	22773063	22829791	1
ENSG00000163293	-6.16423619	0.010539215	3.49E-07	6.81E-05	NIPAL1	4	47914142	48040173	1
ENSG00000167034	-5.35736799	-0.433275463	4.13E-05	0.00218824	NKX3-1	8	23678693	23682927	-1
ENSG00000196338	2.232026956	4.869668443	1.78E-07	4.15E-05	NLGN3	X	71144831	71171201	1
ENSG00000197696	-1.82248025	3.530652885	6.55E-06	0.00058976	NMB	15	84655129	84658563	-1
ENSG00000167207	-3.20736905	0.837425688	0.00015539	0.00539346	NOD2	16	50693603	50733077	1
ENSG00000130935	-0.92203126	6.343402916	0.00033491	0.00915333	NOL11	17	67717833	67744531	1
ENSG00000197183	1.541466857	3.835162637	0.00037992	0.00997398	NOL4L	20	32443059	32585074	-1
ENSG00000111641	-1.40332199	5.097285419	2.42E-05	0.00150818	NOP2	12	6556863	6568691	-1
ENSG00000074181	1.783682676	4.905725906	7.19E-06	0.00062436	NOTCH3	19	15159038	15200981	-1
ENSG00000196408	-3.80277739	-0.968006591	0.00024543	0.00743842	NOXO1	16	1978917	1984192	-1
ENSG00000169246	5.923733324	-0.96860619	7.93E-08	2.36E-05	NPIPB3	16	21402237	21448567	-1
ENSG00000243716	4.435425229	-1.091247872	4.18E-05	0.00220514	NPIPB5	16	22479121	22536521	1
ENSG00000175745	1.912586876	5.930265386	2.60E-05	0.00156997	NR2F1	5	93583337	93594615	1
ENSG00000185551	1.837925229	3.223024877	0.00023948	0.00728406	NR2F2	15	96325938	96340263	1
ENSG00000148572	-1.56203058	4.260365623	1.79E-05	0.00122696	NRBF2	10	63133247	63155031	1
ENSG00000137404	3.207959928	-0.492784149	0.00023785	0.00724759	NRM	6	30688047	30691420	-1
ENSG00000125841	1.817487615	4.230180862	0.00033806	0.00920237	NRSN2	20	346782	359660	1
ENSG00000198400	-7.95491477	-0.057261167	0.00023855	0.0072623	NTRK1	1	156815640	156881850	1
ENSG00000148053	2.094028893	11.31289531	1.64E-05	0.00115659	NTRK2	9	84668551	85027070	1
ENSG00000120526	-1.51633211	5.024782104	1.61E-05	0.00114262	NUDCD1	8	109240919	109334385	-1

ENSG00000136159	-1.4678289	3.797808279	2.02E-05	0.00133381	NUDT15	13	48037567	48047222	1
ENSG00000272325	1.116453201	8.11010248	0.00033355	0.00912354	NUDT3	6	34279679	34392674	-1
ENSG00000126883	1.940770077	4.84672007	5.40E-06	0.00050594	NUP214	9	131125561	131234670	1
ENSG00000132661	-1.42766901	3.373238304	2.55E-05	0.0015581	NXT1	20	23350736	23354777	1
ENSG00000183801	-6.18897482	-0.311721874	0.00018657	0.00615454	OLFML1	11	7485388	7511377	1
ENSG00000184221	7.044656597	1.720486702	9.41E-08	2.68E-05	OLIG1	21	33070144	33072420	1
ENSG00000126861	1.732562331	3.490496231	7.16E-05	0.00318146	OMG	17	31272013	31297539	-1
ENSG00000116885	-1.35763398	4.93778184	0.00012175	0.00459906	OSCP1	1	36415827	36450451	-1
ENSG00000262664	-1.49125702	4.026363701	0.00012097	0.00458808	OVCA2	17	2041936	2043430	1
ENSG00000117385	-1.81506397	5.162620697	0.00021787	0.00681631	P3H1	1	42746335	42767084	-1
ENSG00000072682	-2.87636186	4.374085453	0.00037478	0.00987733	P4HA2	5	132191838	132295315	-1
ENSG00000070756	1.56552946	6.300853914	2.48E-05	0.00152527	PABPC1	8	100685816	100722809	-1
ENSG00000180370	1.723377436	6.243794312	4.89E-07	8.56E-05	PAK2	3	196739857	196832647	1
ENSG00000187867	2.825534961	1.969945813	7.08E-05	0.00315062	PALM3	19	14053365	14059159	-1
ENSG00000073150	3.039230597	0.771412405	0.00024578	0.00744245	PANX2	22	50170731	50180294	1
ENSG00000170915	1.356861476	4.673954436	0.00029735	0.00846772	PAQR8	6	52361421	52407777	1
ENSG00000166889	1.738895433	3.666478045	0.00019267	0.00628962	PATL1	11	59636716	59668980	-1
ENSG00000132570	1.344788653	3.454684066	0.00021014	0.00664797	PCBD2	5	134904906	135007959	1
ENSG00000171815	2.594565729	1.885473828	0.00020824	0.00659994	PCDHB1	5	141051135	141059344	1
ENSG00000253305	2.315874321	5.623079361	0.00034472	0.00933142	PCDHGB6	5	141408021	141512979	1
ENSG00000240184	3.275296592	4.172172857	0.00012275	0.00461208	PCDHGC3	5	141475947	141512979	1
ENSG00000126226	-1.1755123	5.598907529	2.49E-05	0.00152533	PCID2	13	113177539	113208715	-1
ENSG00000100982	1.320359164	3.388623438	0.0003425	0.0092936	PCIF1	20	45934628	45948023	1
ENSG00000248485	-5.57548938	-1.286768625	9.36E-05	0.00385383	PCP4L1	1	161258727	161285450	1
ENSG00000102230	1.585408157	5.553946828	0.00020566	0.00656355	PCYT1B	X	24558087	24672677	-1
ENSG00000005882	1.879656528	4.163452611	2.09E-06	0.00026198	PDK2	17	50094737	50112152	1
ENSG00000179889	-1.48351854	5.59330175	4.80E-05	0.0024049	PDXDC1	16	14974591	15139339	1
ENSG00000162734	1.144778469	9.070553306	3.62E-05	0.00196798	PEA15	1	160205337	160215376	1
ENSG00000152684	-1.39199961	4.932374915	0.00017308	0.00583445	PELO	5	52787940	52804046	1
ENSG00000100029	-1.15763527	6.095482074	0.00027175	0.00798873	PES1	22	30576625	30607083	-1
ENSG00000148985	1.364361264	3.673280139	0.00018801	0.00618702	PGAP2	11	3797724	3826371	1
ENSG00000013375	-1.17785148	7.711906633	6.49E-05	0.00297087	PGM3	6	83161150	83193936	-1
ENSG00000204138	2.672832865	4.43887893	8.84E-07	0.00013514	PHACTR4	1	28369582	28500369	1
ENSG00000111752	1.363297454	5.183001452	0.00018285	0.00606734	PHC1	12	8913896	8941467	1
ENSG00000109118	2.33025275	4.391225623	6.66E-07	0.00010869	PHF12	17	28905250	28951771	-1
ENSG00000135365	1.814504325	4.846219699	0.00031522	0.00886501	PHF21A	11	45929323	46121178	-1
ENSG00000100410	-1.12908964	4.954931845	0.00019877	0.00643817	PHF5A	22	41459717	41468725	-1
ENSG00000174307	-2.47785207	5.149481922	2.54E-06	0.00030096	PHLDA3	1	201464383	201469237	-1
ENSG00000198355	-2.41680668	2.985518488	7.76E-05	0.00340182	PIM3	22	49960513	49964080	1
ENSG00000087842	2.199549844	5.094754405	4.43E-05	0.00229108	PIR	X	15384799	15493564	-1
ENSG00000162878	-2.92235859	3.212613213	5.76E-05	0.00271685	PKDCC	2	42048020	42058528	1
ENSG00000165495	1.476915619	3.33582006	0.00035327	0.00950976	PKNOX2	11	125164687	125433389	1
ENSG00000103066	-1.38018282	4.67715187	0.00013049	0.00480231	PLA2G15	16	68245304	68261062	1
ENSG00000126003	1.697585153	3.243417472	8.34E-05	0.00355616	PLAGL2	20	32192503	32207791	-1
ENSG00000145632	-1.78842745	6.67558345	0.00027946	0.00809396	PLK2	5	58453982	58460260	-1
ENSG00000173846	-3.33521206	3.086990399	3.41E-05	0.00188291	PLK3	1	44800225	44805990	1
ENSG00000198753	2.942767321	2.332424369	1.25E-10	2.35E-07	PLXNB3	X	153764196	153779346	1
ENSG00000141682	-3.25255277	4.170559633	2.73E-07	5.78E-05	PMAIP1	18	59899948	59904306	1
ENSG00000124225	-2.10626828	5.909600276	2.15E-05	0.00138268	PMEPA1	20	57648392	57711536	-1
ENSG00000140650	-2.89258908	4.204327238	0.00011151	0.00432579	PMM2	16	8788823	8849331	1
ENSG00000147588	2.629426607	9.904386753	0.00021503	0.0067584	PMP2	8	81440326	81447523	-1
ENSG00000171453	-1.1568204	5.263153951	0.00016988	0.00577246	POLR1C	6	43509702	43529585	1
ENSG00000102978	-1.37443527	6.290083909	3.95E-06	0.00041654	POLR2C	16	57462387	57472010	1
ENSG00000163882	-1.51477248	4.994729242	1.46E-07	3.63E-05	POLR2H	3	184361718	184368596	1
ENSG00000104356	-1.67577151	3.766861816	2.92E-05	0.00170381	POP1	8	98117297	98159834	1
ENSG00000077157	1.503073644	6.045516823	7.58E-05	0.00333613	PPP1R12B	1	202348699	202592706	1
ENSG00000243279	2.35404367	2.09235193	0.00026063	0.00776173	PRAF2	X	49071156	49074071	-1
ENSG00000138073	-1.24116462	5.150712103	5.21E-05	0.00254336	PREB	2	27130756	27134675	-1
ENSG00000163932	-2.69438681	0.784228852	8.87E-05	0.00368662	PRKCD	3	53156009	53192717	1
ENSG00000253729	1.214503859	8.849845544	0.00024316	0.00737619	PRKDC	8	47773108	47960183	-1
ENSG00000172179	-9.97799476	-1.435334666	1.43E-06	0.00019083	PRL	6	22287244	22297501	-1
ENSG00000126457	-1.27006184	6.88721548	2.77E-05	0.0016325	PRMT1	19	49675786	49689029	1
ENSG00000117707	1.612048857	4.79791808	5.04E-05	0.00249017	PROX1	1	213983181	214041502	1
ENSG00000174231	1.645615936	7.116231649	0.00028527	0.00819246	PRPF8	17	1650629	1684882	-1
ENSG00000205352	1.528573585	3.766457897	0.00014953	0.00525981	PRR13	12	53441605	53446645	1
ENSG00000204469	2.083163278	5.028457753	4.42E-05	0.00228853	PRRC2A	6	31620720	31637771	1
ENSG00000117523	1.364259213	7.212812751	0.00014045	0.00501588	PRRC2C	1	171485551	171593511	1
ENSG00000150687	-2.16939273	5.827821718	0.0001611	0.00552921	PRSS23	11	86791059	86952910	1
ENSG00000146250	-3.86526236	7.217914925	1.88E-05	0.0012709	PRSS35	6	83512538	83525704	1
ENSG00000143106	-1.11078338	6.389970278	0.00014601	0.00515758	PSMA5	1	109399031	109426427	-1
ENSG00000159377	-0.89010449	7.20719322	9.67E-05	0.00392908	PSMB4	1	151399534	151401944	1
ENSG00000100519	-0.95975712	6.747090827	0.00016066	0.00552921	PSMC6	14	52707172	52728587	1
ENSG00000099341	-1.42594319	6.717392157	0.00013783	0.0049667	PSMD8	19	38374536	38383824	1
ENSG00000131467	1.250959282	5.1379558	6.25E-05	0.0029002	PSME3	17	42824385	42843758	1
ENSG00000119314	1.532235259	7.238390227	6.26E-05	0.00290045	PTBP3	9	112217716	112333667	-1
ENSG00000185920	2.139365941	5.034738368	0.00015526	0.00539346	PTCH1	9	95442980	95517057	-1
ENSG00000125384	-8.35413952	-1.138681866	1.38E-06	0.00018812	PTGER2	14	52314305	52328606	1
ENSG00000073756	-7.64392335	1.310720215	1.54E-05	0.00110204	PTGS2	1	186671791	186680427	-1
ENSG00000160801	-5.47035026	0.048997973	0.00025026	0.00753768	PTH1R	3	46877746	46903799	1
ENSG00000087494	-4.73694507	1.667737001	2.47E-06	0.00029429	PTHLH	12	27958084	27972705	-1
ENSG00000153707	1.61646658	7.784981017	8.07E-06	0.00067439	PTPRD	9	8314246	10612723	-1

ENSG00000060656	-3.99892114	1.928764615	4.55E-05	0.00232462	PTPRU	1	29236516	29326813	1
ENSG00000106278	2.055142757	9.546350196	6.54E-08	2.00E-05	PTPRZ1	7	121873089	122062036	1
ENSG00000177469	-2.00113496	7.766083868	0.00020153	0.00650902	PTRF	17	42402452	42423517	-1
ENSG00000185129	1.742054649	5.128425754	0.00036239	0.00964798	PURA	5	140107777	140125619	1
ENSG00000110060	-1.23743462	3.664446336	0.00013893	0.00499566	PUS3	11	125893485	125903221	-1
ENSG00000089159	2.081489937	3.643761531	5.69E-06	0.00052593	PXN	12	120210439	120265771	-1
ENSG00000116260	-1.59919737	4.60666702	0.00020372	0.00654429	QSOX1	1	180154834	180204030	1
ENSG00000156675	-2.59467662	5.532052676	0.00019106	0.00626019	RAB11FIP1	8	37858618	37899467	-1
ENSG00000107560	1.454224339	6.319920345	0.00012296	0.00461208	RAB11FIP2	10	118004916	118046603	-1
ENSG00000172007	-0.99820877	4.990111023	0.0003574	0.00956024	RAB33B	4	139453232	139476609	1
ENSG00000075785	1.488834676	6.073198611	1.31E-05	0.00097254	RAB7A	3	128726122	128814796	1
ENSG00000108352	2.001598055	2.461981313	6.99E-06	0.00061431	RAPGEFL1	17	40177010	40195656	1
ENSG00000113643	-0.91777606	6.857126728	0.00021587	0.00677848	RARS	5	168486445	168519299	1
ENSG00000122035	-4.69354509	0.852592483	0.00033329	0.00912354	RASL11A	13	27270327	27273690	1
ENSG00000068028	-1.25128768	4.322005324	0.00012163	0.00459906	RASSF1	3	50329782	50340980	-1
ENSG00000184863	1.503056392	5.397632132	9.84E-05	0.00395884	RBM33	7	155644451	155781485	1
ENSG00000076067	1.753852645	4.314372802	0.00011832	0.00450754	RBMS2	12	56521929	56596196	1
ENSG00000173653	-1.65948157	2.937442952	7.05E-05	0.00314551	RCE1	11	66842835	66846546	1
ENSG00000117625	1.406940435	5.391158302	0.00026899	0.00793304	RCOR3	1	211258377	211316385	1
ENSG00000054967	-2.25274003	2.982079757	0.00025749	0.00768745	RELT	11	73376264	73397474	1
ENSG00000079313	1.901257635	2.422284736	8.36E-05	0.00355921	REXO1	19	1815246	1848463	-1
ENSG00000111783	2.143426876	8.011183167	9.65E-07	0.00014309	RFX4	12	106582907	106762803	1
ENSG00000182175	2.72241039	6.052036305	1.22E-05	0.00091842	RGMA	15	93035273	93089204	-1
ENSG00000153165	-6.47206195	0.000417695	9.74E-07	0.00014318	RGPD3	2	106391290	106468376	-1
ENSG00000183054	-8.14629487	-1.410500157	5.89E-06	0.00053997	RGPD6	2	110513812	110577185	-1
ENSG00000116741	-2.94410751	4.390126315	4.73E-05	0.00237768	RGS2	1	192809039	192812283	1
ENSG00000117152	-5.33392517	3.791370948	3.39E-05	0.00187483	RGS4	1	163068775	163076802	1
ENSG00000188672	-4.21203446	-1.041597788	0.00017136	0.00580251	RHCE	1	25362249	25430192	-1
ENSG00000125352	-1.60505998	2.883876983	3.37E-05	0.00187438	RNF113A	X	119870475	119871827	-1
ENSG00000170881	-0.98647024	6.760414726	0.00022477	0.00695378	RNF139	8	124474738	124487914	1
ENSG00000235631	-3.03114357	0.165995296	0.00037535	0.00988392	RNF148	7	122701664	122702967	-1
ENSG00000108523	1.460322744	5.081940199	9.97E-05	0.00399458	RNF167	17	4940008	4945222	1
ENSG00000168894	-1.10408318	4.819714589	8.11E-05	0.0035021	RNF181	2	85595725	85597613	1
ENSG00000173821	1.414624642	6.830165358	0.00012399	0.00463512	RNF213	17	80260866	80398786	1
ENSG00000163481	-1.41150747	4.781325103	1.44E-05	0.00104095	RNF25	2	218663864	218672411	-1
ENSG00000173456	2.019891645	3.529244173	0.00012174	0.00459906	RNF26	11	119334527	119337313	1
ENSG00000181852	-1.62220784	6.452198456	5.19E-07	8.99E-05	RNF41	12	56202175	56221933	-1
ENSG00000135119	1.67910661	5.034988052	8.16E-06	0.00067742	RNFT2	12	116738178	116853631	1
ENSG00000100316	-1.02341446	9.783016765	0.00037312	0.00984109	RPL3	22	39312882	39320389	-1
ENSG00000137818	2.82382038	7.383458521	1.40E-05	0.00102403	RPLP1	15	69452784	69456194	1
ENSG00000163902	-1.03543873	8.529793392	0.00011597	0.00444545	RPN1	3	128619970	128681075	-1
ENSG00000163125	2.226743162	4.893564883	9.01E-06	0.00072845	RPRD2	1	150363091	150476566	1
ENSG00000185088	-1.47905312	7.05629005	2.83E-06	0.00032799	RPS27L	15	63125872	63158021	-1
ENSG00000149273	-1.16175667	8.460387391	0.00017893	0.00597221	RPS3	11	75399486	75422280	1
ENSG00000166133	-1.67830985	3.704663724	1.66E-05	0.00116727	RPUSD2	15	40569300	40574943	1
ENSG00000048392	-1.39469144	7.668626701	1.25E-05	0.00093379	RRM2B	8	102204502	102239118	-1
ENSG00000133318	1.601244265	8.285631732	5.36E-06	0.0005058	RTN3	11	63681446	63759891	1
ENSG00000185924	-7.00315995	-1.70376112	0.00024139	0.0073357	RTN4RL1	17	1934677	2025345	-1
ENSG00000160753	1.560573412	3.441396839	0.00022838	0.00702848	RUSC1	1	155320896	155331114	1
ENSG00000198838	2.592766953	5.770734907	5.17E-05	0.00253706	RYR3	15	33310945	33866121	1
ENSG00000170989	-1.72564383	6.693864173	0.00013252	0.00482654	S1PR1	1	101236888	101241518	1
ENSG00000103449	1.951500192	6.850027691	5.98E-05	0.00279721	SALL1	16	51135975	51151367	-1
ENSG00000165821	2.283372854	6.494602644	4.35E-06	0.0004445	SALL2	14	21521081	21537216	-1
ENSG00000179134	1.541656202	4.702122634	0.00013336	0.00483647	SAMD4B	19	39342396	39385710	1
ENSG00000136715	1.878983055	4.118319048	1.18E-05	0.00089668	SAP130	2	127941217	128028120	-1
ENSG00000168077	1.915325983	6.458527773	7.38E-06	0.00063421	SCARA3	8	27633868	27676776	1
ENSG00000079689	4.887852899	0.550555262	9.70E-05	0.00393425	SCGN	6	25652201	25701783	1
ENSG00000162512	2.382325994	8.029561004	1.39E-06	0.00018939	SDC3	1	30869467	30908761	-1
ENSG00000117118	-0.97255146	5.801637659	0.00011326	0.004374	SDHB	1	17018722	17054170	-1
ENSG00000100934	-1.46984507	7.460183546	3.82E-06	0.0004073	SEC23A	14	39031919	39109646	-1
ENSG00000132432	-1.35686376	5.811144819	0.00021944	0.00684645	SEC61G	7	54752250	54759974	-1
ENSG00000075223	-2.7322868	4.236890905	8.03E-05	0.00348259	SEMA3C	7	80742538	80922359	-1
ENSG00000001617	-3.45288174	0.919829441	8.70E-05	0.00363904	SEMA3F	3	50155045	50189075	1
ENSG00000010319	-4.50197815	-0.499987153	1.68E-05	0.00117212	SEMA3G	3	52433053	52445085	-1
ENSG00000092421	2.056258283	5.835341631	0.00035605	0.00954862	SEMA6A	5	116443616	116574934	-1
ENSG00000161956	-1.08016929	5.907817422	0.00011146	0.00432579	SENP3	17	7561875	7571969	1
ENSG00000119231	-1.11669728	6.75523747	0.00017554	0.00589398	SENP5	3	196867856	196934714	1
ENSG00000162430	2.203699126	4.864932999	8.63E-05	0.00361584	SEPN1	1	25800176	25818224	1
ENSG00000168528	1.839477397	1.959845842	0.00010899	0.00427214	SERINC2	1	31409565	31434680	1
ENSG00000166401	-2.26379409	4.039550975	0.00022899	0.00703446	SERPINB8	18	63969925	64005667	1
ENSG00000106366	-4.03851172	4.332359459	0.00013725	0.00495097	SERPINE1	7	101127089	101139266	1
ENSG00000149131	2.857000029	4.439053952	1.77E-06	0.00022826	SERPING1	11	57597387	57614853	1
ENSG00000197019	-3.94773868	4.647554378	5.24E-09	3.35E-06	SERTAD1	19	40421592	40426025	-1
ENSG00000152217	2.651076401	5.83263294	3.57E-06	0.00039086	SETBP1	18	44680173	45068510	1
ENSG00000099381	1.95724336	2.585439557	7.20E-05	0.00319144	SETD1A	16	30957294	30985116	1
ENSG00000168137	1.596007616	6.357768226	1.22E-05	0.00091773	SETD5	3	9397615	9479240	1
ENSG00000230667	-7.99070118	0.213961652	1.53E-10	2.60E-07	SETSIP	1	92074533	92075441	-1
ENSG00000143368	1.964576208	3.715871414	3.39E-05	0.00187527	SF3B4	1	149923317	149928344	-1
ENSG00000213064	1.144738148	6.836235623	0.00020436	0.00655057	SFT2D2	1	168225938	168253025	1
ENSG00000118515	-4.05855976	3.238568702	0.00011561	0.00444449	SGK1	6	134169246	134318112	-1
ENSG00000164023	-3.28424571	4.976230492	5.04E-06	0.000489	SGMS2	4	107824563	107915047	1

ENSG00000027869	-7.10798662	-1.547875024	0.00027302	0.00799627	SH2D2A	1	156806243	156816862	-1
ENSG00000172985	2.539312761	3.380765727	2.13E-06	0.00026604	SH3RF3	2	109129348	109504632	1
ENSG00000161681	-4.38948714	0.174614614	0.0002609	0.00776173	SHANK1	19	50661827	50719450	-1
ENSG00000197555	1.315000927	6.89859982	2.81E-05	0.00165602	SIPA1L1	14	71320449	71741229	1
ENSG00000105738	1.972667589	3.49290858	0.00021671	0.00679522	SIPA1L3	19	37907228	38208372	1
ENSG00000005020	1.815477114	3.283041019	0.00022789	0.00701982	SKAP2	7	26667062	26995239	-1
ENSG00000157933	2.207973103	4.149836632	2.12E-05	0.00137291	SKI	1	2228695	2310119	1
ENSG00000140199	1.407099925	5.257220535	4.54E-05	0.00232462	SLC12A6	15	34229996	34338060	-1
ENSG00000110446	-2.63773598	2.348069339	0.00037773	0.00992424	SLC15A3	11	60937083	60952530	-1
ENSG00000147100	1.631972149	5.79673252	7.07E-05	0.00314875	SLC16A2	X	74421461	74533917	1
ENSG00000117479	-1.80200576	4.515820281	0.00013211	0.00481674	SLC19A2	1	169463909	169486003	-1
ENSG00000162383	-8.82950364	-1.259442171	1.43E-06	0.00019083	SLC1A7	1	53087179	53142632	-1
ENSG00000089057	1.391533815	5.853034296	4.75E-05	0.00238277	SLC23A2	20	4852356	5010293	-1
ENSG00000125648	1.542525628	4.508568976	8.12E-06	0.00067558	SLC25A23	19	6436079	6465203	-1
ENSG00000164933	-2.26052997	4.504771934	6.01E-05	0.00280813	SLC25A32	8	103398635	103415189	-1
ENSG00000151729	-1.10269517	6.415459536	0.00032896	0.00906384	SLC25A4	4	185143241	185150382	1
ENSG00000170385	-2.12124761	7.002632531	1.34E-06	0.00018383	SLC30A1	1	211571568	211578742	-1
ENSG00000176087	1.565164144	4.328388738	2.68E-05	0.00160175	SLC35A4	5	140564456	140569103	1
ENSG00000157593	-1.33076642	5.940178556	4.37E-05	0.00227594	SLC35B2	6	44254096	44257890	-1
ENSG00000157800	-1.17450672	6.406915095	0.00028339	0.00815571	SLC37A3	7	140293693	140404433	-1
ENSG00000104635	-1.48540281	6.23792486	6.50E-05	0.00297087	SLC39A14	8	22367249	22434129	1
ENSG00000168003	-1.4621449	7.374443281	0.00011279	0.0043607	SLC3A2	11	62856102	62888875	1
ENSG00000131389	-1.69556027	4.40121635	0.00037208	0.00982131	SLC6A6	3	14402576	14489349	1
ENSG00000090020	2.206772272	1.312507985	0.00028223	0.00813947	SLC9A1	1	27098815	27166981	-1
ENSG00000197818	1.086063211	4.627430937	0.00030101	0.0085434	SLC9A8	20	49812713	49892242	1
ENSG00000170365	2.19329414	5.464078598	8.55E-06	0.00069966	SMAD1	4	145481194	145558079	1
ENSG00000101665	-2.25921951	2.768727841	8.81E-06	0.00071764	SMAD7	18	48919853	48950711	-1
ENSG00000072501	1.26160737	6.780377388	0.00010342	0.00411203	SMC1A	X	53374149	53422728	-1
ENSG00000070366	1.329296709	5.305976589	2.19E-05	0.00140287	SMG6	17	2059839	2303771	-1
ENSG00000116698	1.80938928	4.840041068	5.50E-05	0.00264248	SMG7	1	183472216	183598246	1
ENSG00000167447	-1.07758095	5.704574779	0.000155	0.00539346	SMG8	17	59209400	59215247	1
ENSG00000256235	-1.46984991	4.003651885	0.00026005	0.00775694	SMIM3	5	150777946	150796734	1
ENSG00000136699	1.114252046	4.584488443	0.00020873	0.00660937	SMPD4	2	130151392	130182750	-1
ENSG00000144028	1.088545189	8.196444851	0.00012489	0.00464828	SNRNP200	2	96274336	96305515	-1
ENSG00000077312	1.830048894	4.165105743	1.68E-05	0.00117212	SNRPA	19	40750637	40765389	1
ENSG00000125835	-1.97288701	5.702424905	2.38E-05	0.0015012	SNRPB	20	2461634	2470853	-1
ENSG00000143376	1.378922117	5.963710728	4.29E-05	0.00225789	SNX27	1	151612065	151699091	1
ENSG00000120833	-1.16056108	5.523328463	0.00026509	0.00784521	SOCS2	12	93569814	93583487	1
ENSG00000171150	-1.13366272	5.791052316	0.00025087	0.00754939	SOCS5	2	46698952	46763129	1
ENSG00000115904	1.797147834	5.8194492	0.00031747	0.00889883	SOS1	2	38981396	39124345	-1
ENSG00000177732	2.794593524	3.657749776	1.91E-07	4.35E-05	SOX12	20	325401	330224	1
ENSG00000134532	2.238200843	6.351748515	2.39E-05	0.0015012	SOX5	12	23529500	23951032	-1
ENSG00000125398	2.08849634	5.443749076	3.69E-05	0.00198983	SOX9	17	72121020	72126420	1
ENSG00000135899	2.878883905	1.420635123	7.26E-06	0.00062842	SP110	2	230167293	230225729	-1
ENSG00000158792	-3.04012988	3.532737754	8.65E-07	0.00013285	SPATA2L	16	89696343	89701705	-1
ENSG00000128487	1.415434468	8.027835598	5.70E-05	0.0026985	SPECC1	17	20009344	20319026	1
ENSG00000072195	1.354657658	5.056220556	0.00010249	0.00409279	SPEG	2	219434846	219498287	1
ENSG00000176170	-5.09287025	3.255663801	8.73E-08	2.53E-05	SPHK1	17	76376584	76387860	1
ENSG00000063176	1.398284318	3.478851582	9.39E-05	0.0038656	SPHK2	19	48619291	48630717	1
ENSG00000166145	-5.97799387	-1.498426022	7.91E-05	0.00344843	SPINT1	15	40844018	40858207	1
ENSG00000157837	1.350490349	4.624655978	0.00020623	0.00656355	SPPL3	12	120762510	120904371	-1
ENSG00000203772	2.805603415	2.912491759	5.40E-08	1.70E-05	SPRN	10	133420666	133424572	-1
ENSG00000137877	-3.69176881	-0.413774587	0.00023498	0.00718581	SPTBN5	15	41848144	41894077	-1
ENSG00000179119	-1.47256328	6.326291754	0.00020656	0.00656355	SPTY2D1	11	18606401	18634791	-1
ENSG00000198911	1.525341961	6.430538556	3.12E-05	0.00177299	SREBF2	22	41833079	41907308	1
ENSG00000196935	2.142906249	5.057415562	2.43E-05	0.00150818	SRGAP1	12	63844293	64162221	1
ENSG00000266028	1.298091844	5.062804272	0.00013997	0.00501588	SRGAP2	1	206203345	206464443	1
ENSG00000133226	1.687922261	5.278449071	5.90E-05	0.00276328	SRRM1	1	24631716	24673267	1
ENSG00000167978	2.491288642	6.305919026	2.77E-05	0.0016325	SRRM2	16	2752329	2772538	1
ENSG00000176101	-1.4113998	4.222523422	0.00030968	0.00874943	SSNA1	9	137188647	137190370	1
ENSG00000101638	3.549184637	3.008765149	4.87E-07	8.56E-05	ST8SIA5	18	46667821	46759257	-1
ENSG00000138378	-5.68124095	1.713545108	7.79E-05	0.00340639	STAT4	2	191029576	191151596	-1
ENSG00000159167	-4.33840953	5.269528412	2.74E-07	5.79E-05	STC1	8	23841915	23854807	-1
ENSG00000164543	-2.36038568	5.390200924	1.04E-06	0.00014925	STK17A	7	43582758	43626786	1
ENSG00000107882	1.884499506	2.650830267	0.00026441	0.00783202	SUFU	10	102503987	102633535	1
ENSG00000165416	1.078666997	7.298816364	7.81E-05	0.00341012	SUGT1	13	52652709	52700909	1
ENSG00000148248	-1.94807145	5.267512911	9.42E-05	0.00386974	SURF4	9	133361449	133376166	-1
ENSG00000197283	2.137018215	2.72289205	0.00026611	0.00785617	SYNGAP1	6	33420070	33457541	1
ENSG00000078269	-2.79660354	3.756377909	5.39E-05	0.00260918	SYNJ2	6	157981887	158099176	1
ENSG00000171992	2.34082133	4.008726311	0.00015496	0.00539346	SYNPO	5	150601080	150659220	1
ENSG00000173227	-4.01780741	3.727337045	2.13E-05	0.001377	SYT12	11	67006778	67050863	1
ENSG00000157625	1.367096171	4.981406958	4.33E-06	0.0004445	TAB3	X	30827442	30975084	-1
ENSG00000138162	2.153852639	4.094198842	0.00029414	0.00838344	TACC2	10	121989174	122254545	1
ENSG00000120656	-0.96550916	4.147091154	0.00021166	0.0066834	TAF12	1	28589323	28643085	-1
ENSG00000122728	-5.10262845	-1.27094036	6.77E-05	0.00305769	TAF1L	9	32629454	32635669	-1
ENSG00000170921	1.555388404	6.130683915	0.00025444	0.00763669	TANC2	17	63009556	63427699	1
ENSG00000120948	1.460316531	7.210814171	0.00017203	0.00581644	TARDBP	1	11012344	11026420	1
ENSG00000113407	-1.50789008	7.677930352	2.31E-06	0.00027955	TARS	5	33440696	33469539	1
ENSG00000104946	2.275984724	2.906725637	1.95E-05	0.00129624	TBC1D17	19	49877425	49888749	1
ENSG00000106638	-1.46951281	5.037975489	2.43E-05	0.00150818	TBL2	7	73568932	73578791	-1
ENSG00000187079	1.445616997	7.782365202	0.00035612	0.00954862	TEAD1	11	12674591	12944483	1

ENSG00000109927	2.212667518	2.007606753	2.69E-05	0.00160448	TECTA	11	121101173	121191493	1
ENSG00000162851	-1.39043037	3.98281665	2.05E-05	0.00134388	TFB2M	1	246540560	246566324	-1
ENSG00000105825	-2.1410476	10.45951111	8.04E-05	0.00348259	TFPI2	7	93885397	93890991	-1
ENSG00000131931	-1.23053116	4.320319574	0.00027262	0.00799627	THAP1	8	42836674	42843325	-1
ENSG00000184436	-1.65544119	4.252633059	0.0001923	0.0062885	THAP7	22	20999104	21002196	-1
ENSG00000137801	-4.00647907	8.520912674	1.39E-07	3.53E-05	THBS1	15	39581079	39599466	1
ENSG00000169231	-1.69436726	5.006890992	4.22E-06	0.00043899	THBS3	1	155195588	155209051	-1
ENSG00000131652	-2.32177103	3.101668911	6.97E-05	0.00313033	THOC6	16	3024027	3027755	1
ENSG00000164296	-2.14781219	3.727747895	8.30E-06	0.00068343	TIGD6	5	149993118	150001167	-1
ENSG00000104067	1.706426538	6.214338742	0.00017235	0.00582023	TJP1	15	29699367	29968865	-1
ENSG00000171914	1.86772183	5.76810808	8.59E-05	0.00361584	TLN2	15	62390526	62844631	1
ENSG00000136869	-3.06668849	5.487077242	4.86E-05	0.00242454	TLR4	9	117704332	117716871	1
ENSG00000086598	-1.02902506	8.221307532	0.00012208	0.00459939	TMED2	12	123584531	123598577	1
ENSG00000158604	-0.98284029	6.762964899	0.00031038	0.00875793	TMED4	7	44577894	44582287	-1
ENSG00000075568	1.828431411	5.32154546	1.02E-06	0.00014769	TMEM131	2	97756333	97995891	-1
ENSG00000164112	-4.24520029	1.476920812	1.69E-07	4.04E-05	TMEM155	4	121758933	121765427	-1
ENSG00000198792	1.552190301	4.517133952	0.00014165	0.00504424	TMEM184B	22	38219291	38273034	-1
ENSG00000168701	-1.38209083	4.394306314	9.46E-05	0.00387791	TMEM208	16	67227103	67229278	1
ENSG00000205544	-1.47165984	4.168665068	2.98E-05	0.00172804	TMEM256	17	7402975	7404137	-1
ENSG00000176142	-1.62995797	4.742739913	7.59E-05	0.00333947	TMEM39A	3	119429500	119468830	-1
ENSG00000196187	1.45422069	4.517223856	0.00016352	0.00560061	TMEM63A	1	225845536	225882369	-1
ENSG00000120889	-1.36681133	6.394080942	0.00030438	0.00863166	TNFRSF10B	8	23020133	23069179	-1
ENSG00000006327	-7.38101945	6.084876982	2.98E-12	1.65E-08	TNFRSF12A	16	3018445	3022383	1
ENSG00000125657	-8.46038114	-1.221232636	3.20E-06	0.00035512	TNFSF9	19	6530999	6535928	1
ENSG00000173273	1.126022207	7.22677347	0.00012606	0.00467136	TNKS	8	9555914	9782346	1
ENSG00000000005	-6.73970173	1.706510637	1.72E-05	0.0011915	TNMD	X	100584802	100599885	1
ENSG00000159173	-12.0136308	-0.769397837	1.70E-09	1.38E-06	TNNI1	1	201403768	201429866	-1
ENSG00000182095	1.894773675	5.026765717	5.11E-06	0.00049111	TNRC18	7	5306790	5425414	-1
ENSG00000100354	1.350443689	7.530498879	0.0002213	0.00688353	TNRC6B	22	40044817	40335808	1
ENSG00000132773	-2.39660624	3.528197309	2.44E-06	0.00029134	TOE1	1	45339670	45343975	1
ENSG00000136816	-1.05932231	5.003982872	0.00028106	0.00811966	TOR1B	9	129803153	129811281	1
ENSG00000078804	2.39315257	2.912258341	4.62E-06	0.00046003	TP53INP2	20	34704290	34713439	1
ENSG00000196655	-1.07475183	4.543669934	0.00036267	0.00964798	TRAPPC4	11	119018432	119025454	1
ENSG00000170855	-1.27096149	4.598250086	7.82E-05	0.00341012	TRIAP1	12	120443961	120446412	-1
ENSG00000101255	-2.20815405	4.408619413	6.99E-05	0.00313207	TRIB3	20	362835	397559	1
ENSG00000204713	-1.26495384	4.044025494	0.00018064	0.00600585	TRIM27	6	28903002	28923989	-1
ENSG00000171206	1.531753027	4.654197465	0.00010968	0.00429402	TRIM8	10	102644496	102658407	1
ENSG00000173113	-1.32166269	6.189536868	1.06E-05	0.00082943	TRMT112	11	64316460	64318084	-1
ENSG00000165699	1.113449388	6.652975977	0.00032175	0.00895366	TSC1	9	132891348	132944633	-1
ENSG00000104522	-1.26552668	4.342774296	0.000153	0.00534863	TSTA3	8	143612618	143618048	-1
ENSG00000100154	1.528723882	5.800218427	2.03E-05	0.00133758	TTC28	22	27978014	28679865	-1
ENSG00000105849	-1.07631492	5.234445052	0.00034612	0.00935419	TWISTNB	7	19695462	19709087	-1
ENSG00000184470	-1.90200789	3.853472778	6.33E-05	0.00291839	TXNRD2	22	19875517	19941992	-1
ENSG00000117143	-1.53544495	5.498146549	4.31E-06	0.00044445	UAP1	1	162561506	162599842	1
ENSG00000150991	-1.14232551	10.12023517	4.79E-05	0.00240368	UBC	12	124911604	124917368	-1
ENSG00000078967	1.677392566	3.997427392	0.00033176	0.009104	UBE2D4	7	43926438	43956136	1
ENSG00000130725	-1.77115176	5.015563946	6.31E-05	0.00291795	UBE2M	19	58555712	58558960	-1
ENSG00000244687	2.135495262	5.487930261	2.39E-06	0.00028817	UBE2V1	20	50081124	50115959	-1
ENSG00000130939	1.544101052	5.370765772	1.17E-05	0.00089293	UBE4B	1	10032832	10181239	1
ENSG00000120686	-1.00943801	7.62734126	0.00027836	0.00809396	UFM1	13	38349849	38363619	1
ENSG00000132478	0.953231375	4.63480785	0.00017533	0.00589258	UNK	17	75784771	75825799	1
ENSG00000059145	1.479111356	3.550078908	8.28E-05	0.00354495	UNKL	16	1363205	1414751	-1
ENSG00000140740	-0.94760674	7.159636045	0.00023205	0.0071156	UQCRC2	16	21952660	21983660	1
ENSG00000147679	-1.32927848	5.113142571	3.85E-05	0.002062	UTP23	8	116766503	116849463	1
ENSG00000162738	2.028847107	6.239615719	2.03E-05	0.00133848	VANGL2	1	160400586	160428678	1
ENSG00000071246	1.638069715	5.540591122	6.94E-05	0.00313033	VASH1	14	76762189	76783015	1
ENSG00000170162	-8.36933616	-1.179536984	3.30E-06	0.00036381	VGLL2	6	117265558	117273565	1
ENSG00000131871	-1.30598614	5.976787901	3.94E-05	0.0021039	VIMP	15	101270817	101277500	-1
ENSG00000132549	1.567397386	7.079373882	2.07E-05	0.00135428	VPS13B	8	99013266	99877580	1
ENSG00000048707	1.280378387	7.322578987	0.00011991	0.00455798	VPS13D	1	12230067	12512047	1
ENSG00000134258	-6.49118672	-0.121560742	1.02E-08	5.23E-06	VTCN1	1	117143587	117210960	-1
ENSG00000158195	1.80856179	5.919807451	6.32E-05	0.00291839	WASF2	1	27404226	27490158	-1
ENSG00000106299	1.39262751	5.676357205	0.00031824	0.00890561	WASL	7	123681935	123749067	-1
ENSG00000084463	1.931293983	5.578693239	1.51E-06	0.00019722	WBP11	12	14784579	14803540	-1
ENSG00000132471	1.481964297	4.248578466	0.000236	0.00720427	WBP2	17	75845699	75856507	-1
ENSG00000139668	1.205196895	6.280012005	3.07E-05	0.00175391	WDFY2	13	51584455	51767707	1
ENSG00000163625	1.332197293	8.14717875	6.25E-05	0.0029002	WDFY3	4	84669610	84966391	-1
ENSG00000071127	-1.2660763	8.503988808	4.86E-05	0.00242454	WDR1	4	10074339	10116949	-1
ENSG00000105583	-1.05269178	6.109606779	3.62E-05	0.00196798	WDR83OS	19	12668071	12671356	-1
ENSG00000115935	1.684336101	3.803334343	0.00032201	0.00895366	WIPF1	2	174559572	174682916	-1
ENSG00000011451	2.173369522	3.525207433	6.36E-05	0.00292456	WIZ	19	15419980	15449951	-1
ENSG00000060237	1.771025437	6.780794416	2.61E-06	0.00030661	WNK1	12	752593	911452	1
ENSG00000002745	-7.51435902	0.136775241	0.00016862	0.00574375	WNT16	7	121325367	121341104	1
ENSG00000018408	1.356617531	7.556339966	9.96E-05	0.00399458	WWTR1	3	149517235	149736714	-1
ENSG00000132530	-8.59732483	-1.363366831	0.00018617	0.00614751	XAF1	17	6755447	6775647	1
ENSG00000130733	-1.77033418	4.951795849	1.31E-06	0.00018109	YIPF2	19	10922185	10928681	-1
ENSG00000145817	-1.45002467	6.418839559	6.61E-05	0.00300448	YIPF5	5	144158159	144170714	-1
ENSG00000196449	-1.80633842	3.972695244	0.00010581	0.0041763	YRDC	1	37802944	37808185	-1
ENSG00000108953	1.97097468	7.459818873	2.57E-06	0.00030234	YWHAE	17	1344272	1400378	-1
ENSG00000181722	1.212624067	5.790657588	0.00015574	0.00539927	ZBTB20	3	114338094	115147271	-1
ENSG00000178951	2.117987293	2.658497165	9.28E-06	0.00074335	ZBTB7A	19	4044364	4066945	-1

ENSG00000123200	2.008096879	6.863099064	3.08E-05	0.00175784	ZC3H13	13	45954465	46052759	-1
ENSG00000130749	1.868799284	4.086240517	5.83E-05	0.0027395	ZC3H4	19	47064187	47113752	-1
ENSG00000177054	-1.46825774	5.01178103	8.60E-05	0.00361584	ZDHHC13	11	19117099	19176422	1
ENSG00000169946	2.470211094	2.53617114	1.48E-06	0.00019501	ZFPM2	8	104590733	105804532	1
ENSG00000072121	1.315023504	6.087016948	2.48E-05	0.00152378	ZFYVE26	14	67727374	67816590	-1
ENSG00000155256	-2.17805718	5.435230079	2.60E-08	9.58E-06	ZFYVE27	10	97737121	97760907	1
ENSG00000101040	2.014972103	6.23587393	6.20E-06	0.00056397	ZMYND8	20	47209214	47356889	-1
ENSG00000006194	-0.89289966	5.728691758	0.00035691	0.00955464	ZNF263	16	3263800	3301401	1
ENSG00000166526	-0.87586756	6.139458952	0.00034159	0.00928366	ZNF3	7	100064033	100082548	-1
ENSG00000171467	1.793224687	5.649084654	1.72E-05	0.0011915	ZNF318	6	43307134	43369478	-1
ENSG00000160094	1.68239431	3.903498028	6.78E-06	0.00060402	ZNF362	1	33256545	33300719	1
ENSG00000126746	1.642232459	4.84057562	1.10E-05	0.000852	ZNF384	12	6666477	6689572	-1
ENSG00000148143	1.53928571	7.796437836	0.00013117	0.00480318	ZNF462	9	106863097	107013634	1
ENSG00000204604	-1.45750553	4.832209405	0.00017427	0.00586269	ZNF468	19	52838008	52857649	-1
ENSG00000074657	1.400138248	7.461574253	9.33E-06	0.00074528	ZNF532	18	58862600	58986480	1
ENSG00000167962	1.178171428	3.876149697	0.00028036	0.00810969	ZNF598	16	1997654	2009823	-1
ENSG00000173545	-1.5086097	4.797768514	0.00025635	0.00767364	ZNF622	5	16451519	16465792	-1
ENSG00000198740	1.410830663	5.343311545	8.25E-05	0.00353867	ZNF652	17	49289206	49362473	-1
ENSG00000164684	1.522594024	5.651904787	4.38E-06	0.00044571	ZNF704	8	80628451	80874781	-1
ENSG00000151612	1.810562803	4.907036524	4.23E-06	0.00043899	ZNF827	4	145757627	145938635	-1
ENSG00000066379	-1.53903015	2.616672947	0.00018828	0.00618702	ZNRD1	6	30058899	30064909	1
