# Supplementary material for: THC exposure of human iPSC neurons impacts genes associated with neuropsychiatric disorders
Source: Transl Psychiatry. 2018 Apr 25;8:89. doi: 10.1038/s41398-018-0137-3 (PMC5915454; doi:10.1038/s41398-018-0137-3)
Supplement: Supplementary file 5 — Supplementary Table 4 [file 41398_2018_137_MOESM5_ESM.pdf]

Supplementary Table 4: Glutamate receptor and mitochondrial associated genes altered in response to acute or chronic doses of THC

Mitochondrial genes	Acute THC dose	Chronic THC doses
MT-ATP6	✔	
UQCRBP1	✔	
SDHA	✔	
DDIT4	✔	
DDX28		✔
DNAJA3		✔
HAX1		✔
KIAA1683		✔
TRIAP1		✔
RARS		✔
CS		✔
CYB5R3		✔
CYC1		✔
DIABLO		✔
GBF1		✔
HADH		✔
MRPL28		✔
MRPS18B		✔
PTRF		✔
SDHB		✔
UQCRC2		✔
YRDC		✔
MARS2		✔
MAVS		✔
SLC25A4		✔
MRPL17		✔
MT-ND4	✔	✔
MT-CO1	✔	✔
MT-CO2	✔	✔
MT-CO3	✔	✔
MT-ND4L	✔	✔
ADPRHL2	✔	✔
GRPEL1	✔	✔
ALDH18A1	✔	✔
COQ10B	✔	✔
COX7A2	✔	✔
DMPK	✔	✔
HAGH	✔	✔
MTHFD2	✔	✔
MGST1	✔	✔
MTFP1	✔	✔

MRPL14	✔	✔
MRPL49	✔	✔
MRPL54	✔	✔
MRPS7	✔	✔
PMAIP1	✔	✔
PDK2	✔	✔
RGS2	✔	✔
RPS3	✔	✔
SGK1	✔	✔
SLC25A32	✔	✔
TFB2M	✔	✔
**Glutamate receptor genes**		
GRID2	✔	✔
GRIK1	✔	✔
