# Supplementary material for: THC exposure of human iPSC neurons impacts genes associated with neuropsychiatric disorders
Source: Transl Psychiatry. 2018 Apr 25;8:89. doi: 10.1038/s41398-018-0137-3 (PMC5915454; doi:10.1038/s41398-018-0137-3)
Supplement: Supplementary file 6 — Supplementary Table 5 [file 41398_2018_137_MOESM6_ESM.pdf]

Supplementary Table 5: Genes associated with epigenetic regulation altered in response to acute or chronic doses of THC

Gene class	Acute THC dose	Chronic THC doses
**Epigenetic regulation**		
CBX5	✔	✔
DNMT1	✔	✔
GADD45B	✔	✔
APOBEC3C	✔	✔
SETD1A		✔
SETD5		✔
CBX6		✔
KMT2C		✔
NCOA6		✔
MECP2		✔
MBD5		✔
CECR2		✔
MYSM1		✔
PHF21A		✔
BPTF		✔
BAZ2A		✔
FAM175A		✔
FOXA1		✔
KAT6B		✔
KMT2A		✔
LOXL2		✔
