# Supplementary material for: THC exposure of human iPSC neurons impacts genes associated with neuropsychiatric disorders
Source: Transl Psychiatry. 2018 Apr 25;8:89. doi: 10.1038/s41398-018-0137-3 (PMC5915454; doi:10.1038/s41398-018-0137-3)
Supplement: Supplementary file 7 — Supplementary Table 6 [file 41398_2018_137_MOESM7_ESM.pdf]

Supplementary Table 6: Network analyses (Enrichment map pipeline in Cytoscape)

Group Name	P-value	FDR_qvalue
GTPASE ACTIVATOR ACTIVITY	7.37E-04	0.018338
TELENCEPHALON DEVELOPMENT	2.50E-04	0.0079216
POSITIVE REGULATION OF NITROGEN COMPOUND METABOLIC PROCESS	3.60E-09	3.46E-07
CHORDATE EMBRYONIC DEVELOPMENT	1.80E-06	1.04E-04
CHROMOSOME	2.81E-06	1.49E-04
REGULATION OF CELLULAR METABOLIC PROCESS	1.47E-10	2.63E-08
RNA POLYMERASE II TRANSCRIPTION FACTOR ACTIVITY	1.78E-06	1.04E-04
REGULATION OF NUCLEOBASE, NUCLEOSIDE, NUCLEOTIDE AND NUCLEIC ACID METABOLIC PROCESS	2.53E-11	7.50E-09
POSITIVE REGULATION OF TRANSCRIPTION, DNA-DEPENDENT	6.75E-10	8.85E-08
RENAL SYSTEM DEVELOPMENT	6.94E-04	0.018106
NEGATIVE REGULATION OF MACROMOLECULE METABOLIC PROCESS	1.19E-10	2.23E-08
CHROMATIN REMODELING	6.84E-04	0.018106
NEGATIVE REGULATION OF MACROMOLECULE BIOSYNTHETIC PROCESS	9.55E-12	5.37E-09
PROTEIN DOMAIN SPECIFIC BINDING	7.14E-04	0.018106
POSITIVE REGULATION OF TRANSCRIPTION	2.44E-09	2.66E-07
CELL CYCLE	7.60E-06	3.66E-04
NEGATIVE REGULATION OF BIOSYNTHETIC PROCESS	5.67E-11	1.17E-08
EMBRYONIC DEVELOPMENT ENDING IN BIRTH OR EGG HATCHING	2.27E-06	1.26E-04
REGULATION OF CELLULAR BIOSYNTHETIC PROCESS	2.11E-10	3.62E-08
REGULATION OF CELLULAR COMPONENT ORGANIZATION	1.63E-04	0.0054743
TISSUE MORPHOGENESIS	2.10E-05	9.19E-04
CONDENSIN COMPLEX	7.04E-04	0.018106
CHROMATIN ORGANIZATION	1.50E-06	8.95E-05
NEGATIVE REGULATION OF TRANSCRIPTION	4.31E-11	9.41E-09
KINETOCHORE	9.19E-05	0.0034439
NEGATIVE REGULATION OF NUCLEOBASE, NUCLEOSIDE, NUCLEOTIDE AND NUCLEIC ACID METABOLIC PROCESS	2.67E-11	7.50E-09
PRIMARY NEURAL TUBE FORMATION	1.69E-04	0.005622
DNA BINDING	4.70E-08	3.56E-06
POSITIVE REGULATION OF BIOSYNTHETIC PROCESS	2.90E-07	1.87E-05
PROXIMAL/DISTAL PATTERN FORMATION	6.95E-04	0.018106
CHROMOSOME ORGANIZATION	3.03E-09	2.98E-07
POSITIVE REGULATION OF CELLULAR COMPONENT ORGANIZATION	1.92E-04	0.0063463
LIMB DEVELOPMENT	7.53E-04	0.018508
DNA CONFORMATION CHANGE	7.88E-04	0.019128
CHROMOSOMAL PART	4.29E-07	2.72E-05
NEGATIVE REGULATION OF CELLULAR METABOLIC PROCESS	1.01E-09	1.13E-07
MRNA METABOLIC PROCESS	7.67E-04	0.018735
NEGATIVE REGULATION OF TRANSCRIPTION FROM RNA POLYMERASE II PROMOTER	2.74E-09	2.84E-07
M PHASE	3.69E-04	0.011264
REGULATION OF SIGNALING PATHWAY	1.38E-04	0.0047691
TRANSCRIPTION REGULATOR ACTIVITY	2.17E-12	1.71E-09
TRANSCRIPTION COACTIVATOR ACTIVITY	4.05E-06	2.07E-04
FOREBRAIN DEVELOPMENT	1.10E-04	0.0038569
DNA METABOLIC PROCESS	7.48E-05	0.0028565
CENTRAL NERVOUS SYSTEM DEVELOPMENT	7.35E-06	3.61E-04
REGULATION OF CHONDROCYTE DIFFERENTIATION	9.72E-04	0.022755
CHROMOSOME SEGREGATION	2.07E-05	9.16E-04
SULFURIC ESTER HYDROLASE ACTIVITY	1.28E-05	5.92E-04
NUCLEAR PART	2.15E-07	1.43E-05
REGULATION OF MACROMOLECULE METABOLIC PROCESS	4.56E-10	6.48E-08
REGULATION OF RNA METABOLIC PROCESS	1.50E-08	1.20E-06
REGULATION OF TRANSCRIPTION FROM RNA POLYMERASE II PROMOTER	3.69E-10	6.05E-08
NUCLEIC ACID BINDING	8.94E-10	1.11E-07
REGULATION OF BIOSYNTHETIC PROCESS	3.89E-10	6.11E-08
KAINATE SELECTIVE GLUTAMATE RECEPTOR ACTIVITY	7.04E-04	0.018106
SPLICEOSOMAL COMPLEX	6.40E-04	0.017483
CYTOSKELETAL PROTEIN BINDING	5.20E-04	0.015023
MORPHOGENESIS OF AN EPITHELIUM	2.23E-05	9.53E-04
ORGANELLE ORGANIZATION	1.13E-06	6.82E-05
POSITIVE REGULATION OF NUCLEOBASE, NUCLEOSIDE, NUCLEOTIDE AND NUCLEIC ACID METABOLIC PROCESS	8.78E-09	7.68E-07
CELL	7.18E-04	0.018106
CELL PART	7.09E-04	0.018106
CHROMATIN MODIFICATION	2.05E-05	9.14E-04
DNA DAMAGE RESPONSE, SIGNAL TRANSDUCTION	1.37E-05	6.28E-04
REGULATION OF MACROMOLECULE BIOSYNTHETIC PROCESS	4.40E-10	6.48E-08
UROGENITAL SYSTEM DEVELOPMENT	2.38E-05	0.0010078
HINDGUT MORPHOGENESIS	7.04E-04	0.018106
TRANSCRIPTION ACTIVATOR ACTIVITY	1.73E-07	1.20E-05
REGIONALIZATION	9.87E-05	0.0035603
EMBRYONIC EPITHELIAL TUBE FORMATION	8.09E-04	0.019511
BETA-CATENIN BINDING	2.37E-06	1.29E-04
VINCULIN BINDING	1.40E-04	0.0047869
CELL CYCLE PROCESS	7.99E-05	0.0030198
TRANSCRIPTION COFACTOR ACTIVITY	2.44E-07	1.60E-05
POSITIVE REGULATION OF RNA METABOLIC PROCESS	9.93E-10	1.13E-07
CELL CYCLE PHASE	8.45E-04	0.020269
BRAIN DEVELOPMENT	2.55E-04	0.0080325
DNA REPLICATION	5.91E-05	0.0023031
NERVOUS SYSTEM DEVELOPMENT	1.02E-04	0.0036129
REGULATION OF GENE EXPRESSION	2.98E-09	2.98E-07
REGULATION OF TRANSCRIPTION	6.44E-10	8.74E-08
NEGATIVE REGULATION OF GENE EXPRESSION	5.83E-12	3.82E-09
NUCLEOBASE, NUCLEOSIDE, NUCLEOTIDE AND NUCLEIC ACID METABOLIC PROCESS	9.58E-05	0.0034886
SPINAL CORD PATTERNING	7.13E-04	0.018106
SMOOTHENED SIGNALING PATHWAY	5.53E-04	0.015509

NEGATIVE REGULATION OF RNA METABOLIC PROCESS	2.51E-11	7.50E-09
REGULATION OF GENE EXPRESSION, EPIGENETIC	9.62E-04	0.022646
TUBE CLOSURE	9.42E-05	0.0034632
TRANSCRIPTION FACTOR BINDING	7.92E-09	7.08E-07
ACTIN BINDING	2.60E-04	0.0081052
CHROMOSOME, CENTROMERIC REGION	6.33E-06	3.15E-04
ANTERIOR/POSTERIOR PATTERN FORMATION	1.33E-04	0.0046259
DNA METHYLATION	2.30E-04	0.00734
REGULATION OF METABOLIC PROCESS	9.05E-10	1.11E-07
EMBRYONIC APPENDAGE MORPHOGENESIS	6.75E-04	0.018106
REGULATION OF TRANSCRIPTION, DNA-DEPENDENT	2.73E-08	2.10E-06
NUCLEOPLASM	2.10E-06	1.18E-04
NEURAL TUBE CLOSURE	9.42E-05	0.0034632
NUCLEIC ACID METABOLIC PROCESS	9.02E-06	4.27E-04
NUCLEOPLASM PART	3.29E-06	1.73E-04
REGULATION OF CELL DEVELOPMENT	4.06E-05	0.0016282
TRANSCRIPTION REPRESSOR ACTIVITY	2.59E-05	0.001083
NUCLEUS	5.64E-17	2.22E-13
ENZYME BINDING	1.02E-04	0.0036129
EMBRYONIC DEVELOPMENT	3.40E-05	0.0013776
REGULATION OF PRIMARY METABOLIC PROCESS	3.93E-11	9.19E-09
CELLULAR COMPONENT ORGANIZATION	1.21E-05	5.69E-04
POSITIVE REGULATION OF TRANSCRIPTION FROM RNA POLYMERASE II PROMOTER	1.27E-07	9.10E-06
RNA SPLICING, VIA TRANSESTERIFICATION REACTIONS	4.95E-04	0.014415
NEURAL TUBE FORMATION	5.31E-04	0.015247
NEURAL TUBE DEVELOPMENT	2.69E-05	0.0011015
PATTERN SPECIFICATION PROCESS	4.81E-05	0.0019107
KIDNEY DEVELOPMENT	5.39E-04	0.015374
EPITHELIAL TUBE MORPHOGENESIS	2.21E-05	9.53E-04
TUBE DEVELOPMENT	2.62E-05	0.0010853
CELL FATE COMMITMENT	5.01E-05	0.0019711
APPENDAGE DEVELOPMENT	7.53E-04	0.018508
REGULATION OF DNA METABOLIC PROCESS	4.46E-04	0.013383
SPINAL CORD DEVELOPMENT	6.75E-05	0.0026036
TRANSCRIPTION FACTOR ACTIVITY	1.98E-06	1.13E-04
EPITHELIAL TUBE FORMATION	9.23E-04	0.021867
NEGATIVE REGULATION OF TRANSCRIPTION, DNA-DEPENDENT	1.41E-11	6.18E-09
NEGATIVE REGULATION OF MAPKKK CASCADE	7.24E-04	0.018126
NEGATIVE REGULATION OF CELLULAR BIOSYNTHETIC PROCESS	3.05E-11	7.99E-09
POSITIVE REGULATION OF MACROMOLECULE BIOSYNTHETIC PROCESS	5.81E-08	4.31E-06
POSITIVE REGULATION OF GENE EXPRESSION	4.15E-09	3.89E-07
NEGATIVE REGULATION OF NITROGEN COMPOUND METABOLIC PROCESS	3.97E-11	9.19E-09
EMBRYONIC LIMB MORPHOGENESIS	6.75E-04	0.018106
NEGATIVE REGULATION OF METABOLIC PROCESS	9.69E-10	1.13E-07
REGULATION OF NITROGEN COMPOUND METABOLIC PROCESS	1.35E-11	6.18E-09
CONDENSED CHROMOSOME	4.14E-04	0.012514
G2/M TRANSITION CHECKPOINT	1.45E-04	0.0049311
DNA ALKYLATION	2.30E-04	0.00734
INTRACELLULAR SIGNALING PATHWAY	0.0025611	0.04517
MOLECULAR_ FUNCTION	2.03E-04	0.0066578
INTRACELLULAR ORGANELLE PART	2.59E-06	1.39E-04
ORGANELLE	2.05E-11	7.33E-09
REGULATION OF CELLULAR PROCESS	1.08E-10	2.12E-08
INTRACELLULAR ORGANELLE LUMEN	4.67E-04	0.013814
ORGANELLE LUMEN	5.58E-04	0.015509
REGULATION OF CELL CYCLE	2.69E-04	0.0083233
MEMBRANE-ENCLOSED LUMEN	0.0010446	0.024166
ORGANELLE PART	4.73E-06	2.39E-04
POSITIVE REGULATION OF CELLULAR BIOSYNTHETIC PROCESS	1.81E-07	1.23E-05
REGULATION OF BIOLOGICAL PROCESS	4.61E-10	6.48E-08
NEGATIVE REGULATION OF CELLULAR PROCESS	2.60E-09	2.76E-07
NUCLEAR LUMEN	1.58E-05	7.13E-04
GENE EXPRESSION	0.0027105	0.046638
CELLULAR PROCESS	7.04E-07	4.33E-05
NEGATIVE REGULATION OF CELL PROLIFERATION	2.18E-04	0.0070748
INTRACELLULAR ORGANELLE	1.58E-11	6.20E-09
SKELETAL SYSTEM DEVELOPMENT	0.002048	0.038912
CELLULAR NITROGEN COMPOUND METABOLIC PROCESS	0.001609	0.032959
EPITHELIUM DEVELOPMENT	4.81E-04	0.014131
INTRACELLULAR MEMBRANE-BOUNDED ORGANELLE	7.88E-13	8.93E-10
MACROMOLECULAR COMPLEX	3.48E-04	0.010684
BIOLOGICAL REGULATION	1.28E-08	1.07E-06
REGULATION OF INTRACELLULAR PROTEIN KINASE CASCADE	6.28E-04	0.017259
PROTEIN BINDING	7.84E-14	1.54E-10
NITROGEN COMPOUND METABOLIC PROCESS	9.03E-04	0.021526
INTRACELLULAR PART	1.80E-08	1.42E-06
POSITIVE REGULATION OF CELLULAR METABOLIC PROCESS	1.58E-07	1.11E-05
INTRACELLULAR	6.31E-09	5.77E-07
POSITIVE REGULATION OF METABOLIC PROCESS	4.71E-07	2.94E-05
POSITIVE REGULATION OF CELLULAR PROCESS	3.96E-06	2.05E-04
POSITIVE REGULATION OF BIOLOGICAL PROCESS	7.64E-06	3.66E-04
ORGAN MORPHOGENESIS	4.57E-04	0.013607
MORPHOGENESIS OF A BRANCHING EPITHELIUM	0.0019794	0.038186
REGULATION OF CELL PROLIFERATION	5.60E-04	0.015509
NEGATIVE REGULATION OF BIOLOGICAL PROCESS	1.45E-08	1.19E-06
BINDING	1.11E-08	9.53E-07
RNA METABOLIC PROCESS	5.53E-04	0.015509
POSITIVE REGULATION OF MACROMOLECULE METABOLIC PROCESS	6.08E-08	4.43E-06
MEMBRANE-BOUNDED ORGANELLE	9.08E-13	8.93E-10
