# Supplementary material for: THC exposure of human iPSC neurons impacts genes associated with neuropsychiatric disorders
Source: Transl Psychiatry. 2018 Apr 25;8:89. doi: 10.1038/s41398-018-0137-3 (PMC5915454; doi:10.1038/s41398-018-0137-3)
Supplement: Supplementary file 8 — Supplementary Table 7 [file 41398_2018_137_MOESM8_ESM.pdf]

Supplementary Table 7: Disease-related genes

Autism

Gene Number	Chromosome	Gene ID	Gene Name
1	16	ENSG00000183044	ABAT
2	9	ENSG00000165029	ABCA1
3	17	ENSG00000154263	ABCA10
4	19	ENSG00000064687	ABCA7
5	17	ENSG00000159640	ACE
6	7	ENSG00000087085	ACHE
7	X	ENSG00000068366	ACSL4
8	3	ENSG00000136518	ACTL6A
9	19	ENSG00000130402	ACTN4
10	3	ENSG00000243989	ACY1
11	20	ENSG00000196839	ADA
12	20	ENSG00000149451	ADAM33
13	16	ENSG00000140873	ADAMTS18
14	21	ENSG00000197381	ADARB1
15	2	ENSG00000138031	ADCY3
16	3	ENSG00000173175	ADCY5
17	10	ENSG00000156110	ADK
18	20	ENSG00000101126	ADNP
19	22	ENSG00000128271	ADORA2A
20	1	ENSG00000121933	ADORA3
21	5	ENSG00000169252	ADRB2
22	22	ENSG00000239900	ADSL
	X	ENSG00000155966	
23	X	ENSG00000281817	AFF2
24	5	ENSG00000072364	AFF4
25	2	ENSG00000157985	AGAP1
26	12	ENSG00000135439	AGAP2
27	1	ENSG00000186094	AGBL4
28	7	ENSG00000006530	AGK
29	7	ENSG00000187546	AGMO
30	X	ENSG00000180772	AGTR2
31	1	ENSG00000126705	AHDC1
32	6	ENSG00000135541	AHI1
33	7	ENSG00000127914	AKAP9
34	15	ENSG00000184254	ALDH1A3
35	6	ENSG00000112294	ALDH5A1
36	5	ENSG00000164904	ALDH7A1
37	1	ENSG00000088035	ALG6
38	13	ENSG00000132965	ALOX5AP
39	1	ENSG00000116748	AMPD1
40	3	ENSG00000145020	AMT
41	4	ENSG00000145362	ANK2
42	10	ENSG00000151150	ANK3

43	16	ENSG00000167522	ANKRD11
44	12	ENSG00000185046	ANKS1B
45	9	ENSG00000135046	ANXA1
46	X	ENSG00000182287	AP1S2
47	15	ENSG00000034053	APBA2
48	5	ENSG00000134982	APC
49	1	ENSG00000117362	APH1A
50	21	ENSG00000142192	APP
51	X	ENSG00000169083	AR
52	15	ENSG00000187951	ARHGAP11B
53	2	ENSG00000075884	ARHGAP15
54	4	ENSG00000138639	ARHGAP24
55	11	ENSG00000134909	ARHGAP32
56	19	ENSG00000004777	ARHGAP33
57	X	ENSG00000129675	ARHGEF6
58	X	ENSG00000131089	ARHGEF9
59	6	ENSG00000049618	ARID1B
60	15	ENSG00000172379	ARNT2
61	5	ENSG00000164291	ARSK
62	X	ENSG00000004848	ARX
63	12	ENSG00000177981	ASB8
64	1	ENSG00000116539	ASH1L
65	X	ENSG00000196433	ASMT
66	9	ENSG00000130707	ASS1
67	9	ENSG00000148219	ASTN2
68	18	ENSG00000141431	ASXL3
69	3	ENSG00000197548	ATG7
70	15	ENSG00000206190	ATP10A
71	3	ENSG00000127249	ATP13A4
72	19	ENSG00000105409	ATP1A3
73	1	ENSG00000143153	ATP1B1
74	3	ENSG00000157087	ATP2B2
75	4	ENSG00000124406	ATP8A1
76	20	ENSG00000088812	ATRN
77	10	ENSG00000107518	ATRNL1
78	X	ENSG00000085224	ATRX
79	3	ENSG00000163635	ATXN7
80	7	ENSG00000158321	AUTS2
81	20	ENSG00000101200	AVP
82	12	ENSG00000166148	AVPR1A
83	17	ENSG00000266074	BAHCC1
84	17	ENSG00000175866	BAIAP2
85	4	ENSG00000153064	BANK1
86	12	ENSG00000179941	BBS10
87	15	ENSG00000140463	BBS4
88	20	ENSG00000064787	BCAS1
89	16	ENSG00000103507	BCKDK
90	2	ENSG00000119866	BCL11A

91	18	ENSG00000171791	BCL2
92	1	ENSG00000116128	BCL9
93	11	ENSG00000176697	BDNF
94	10	ENSG00000165626	BEND7
95	10	ENSG00000122870	BICC1
96	2	ENSG00000136717	BIN1
97	2	ENSG00000115760	BIRC6
98	7	ENSG00000157764	BRAF
99	13	ENSG00000139618	BRCA2
100	9	ENSG00000078725	BRINP1
101	10	ENSG00000095564	BTAF1
102	6	ENSG00000124557	BTN1A1
103	17	ENSG00000005379	BZRAP1
104	11	ENSG00000158636	C11orf30
105	12	ENSG00000111678	C12orf57
106	15	ENSG00000167014	C15orf43
107	12	ENSG00000182326	C1S
108	1	ENSG00000143612	C1orf43
109	1	ENSG00000203685	C1orf95
110	11	ENSG00000168014	C2CD3
111	3	ENSG00000181744	C3orf58
112	6	ENSG00000224389	C4B
113	1	ENSG00000131686	CA6
114	19	ENSG00000141837	CACNA1A
115	9	ENSG00000148408	CACNA1B
116	12	ENSG00000151067	CACNA1C
117	3	ENSG00000157388	CACNA1D
118	1	ENSG00000198216	CACNA1E
119	X	ENSG00000102001	CACNA1F
120	17	ENSG00000006283	CACNA1G
121	16	ENSG00000196557	CACNA1H
122	22	ENSG00000100346	CACNA1I
123	3	ENSG00000157445	CACNA2D3
124	10	ENSG00000165995	CACNB2
125	11	ENSG00000182985	CADM1
126	3	ENSG00000175161	CADM2
127	7	ENSG00000081803	CADPS2
128	5	ENSG00000152495	CAMK4
129	1	ENSG00000118200	CAMSAP2
130	1	ENSG00000171735	CAMTA1
131	19	ENSG00000182472	CAPN12
132	11	ENSG00000135387	CAPRIN1
133	15	ENSG00000166734	CASC4
134	7	ENSG00000127995	CASD1
135	X	ENSG00000147044	CASK
	21	ENSG00000160200	
136	21	ENSG00000274276	CBS
137	17	ENSG00000141582	CBX4

138	19	ENSG00000132024	CC2D1A
139	12	ENSG00000135127	CCDC64
140	14	ENSG00000015133	CCDC88C
141	12	ENSG00000123106	CCDC91
142	2	ENSG00000115484	CCT4
143	17	ENSG00000186074	CD300LF
144	1	ENSG00000174059	CD34
145	4	ENSG00000004468	CD38
146	11	ENSG00000026508	CD44
147	X	ENSG00000102181	CD99L2
148	14	ENSG00000198752	CDC42BPB
149	17	ENSG00000179604	CDC42EP4
150	5	ENSG00000040731	CDH10
151	16	ENSG00000140937	CDH11
152	16	ENSG00000140945	CDH13
153	16	ENSG00000129910	CDH15
154	20	ENSG00000149654	CDH22
155	16	ENSG00000150394	CDH8
156	5	ENSG00000113100	CDH9
157	X	ENSG00000008086	CDKL5
158	12	ENSG00000111276	CDKN1B
159	22	ENSG00000099954	CECR2
160	18	ENSG00000101489	CELF4
	15	ENSG00000140488	
161	15	ENSG00000273025	CELF6
162	12	ENSG00000198707	CEP290
163	7	ENSG00000106477	CEP41
164	15	ENSG00000128849	CGNL1
165	15	ENSG00000128965	CHAC1
166	15	ENSG00000173575	CHD2
167	8	ENSG00000171316	CHD7
168	14	ENSG00000100888	CHD8
169	22	ENSG00000100288	CHKB
170	3	ENSG00000083937	CHMP2B
171	1	ENSG00000133019	CHRM3
172	20	ENSG00000101204	CHRNA4
173	15	ENSG00000175344	CHRNA7
174	1	ENSG00000160716	CHRNB2
175	8	ENSG00000147432	CHRNB3
176	16	ENSG00000135702	CHST5
177	15	ENSG00000136425	CIB2
178	19	ENSG00000079432	CIC
179	2	ENSG00000074054	CLASP1
180	1	ENSG00000011021	CLCN6
181	3	ENSG00000013297	CLDN11
182	8	ENSG00000182372	CLN8
183	12	ENSG00000139182	CLSTN3
184	22	ENSG00000070371	CLTCL1

185	16	ENSG00000153815	CMIP
186	16	ENSG00000125107	CNOT1
187	19	ENSG00000088038	CNOT3
188	6	ENSG00000118432	CNR1
189	1	ENSG00000188822	CNR2
190	3	ENSG00000113805	CNTN3
191	3	ENSG00000144619	CNTN4
192	11	ENSG00000149972	CNTN5
193	3	ENSG00000134115	CNTN6
194	7	ENSG00000174469	CNTNAP2
195	9	ENSG00000106714	CNTNAP3
196	16	ENSG00000152910	CNTNAP4
197	2	ENSG00000155052	CNTNAP5
198	4	ENSG00000188517	COL25A1
199	22	ENSG00000093010	COMT
200	16	ENSG00000005339	CREBBP
201	7	ENSG00000106113	CRHR2
202	12	ENSG00000008405	CRY1
203	1	ENSG00000009307	CSDE1
204	8	ENSG00000183117	CSMD1
205	17	ENSG00000141551	CSNK1D
206	10	ENSG00000177613	CSTF2T
207	16	ENSG00000102974	CTCF
208	10	ENSG00000183230	CTNNA3
209	3	ENSG00000168036	CTNNB1
210	5	ENSG00000169862	CTNND2
211	7	ENSG00000077063	CTTNBP2
212	2	ENSG00000036257	CUL3
213	6	ENSG00000044090	CUL7
214	7	ENSG00000257923	CUX1
215	3	ENSG00000168329	CX3CR1
216	X	ENSG00000186810	CXCR3
217	15	ENSG00000273749	CYFIP1
218	9	ENSG00000155833	CYLC2
219	8	ENSG00000160882	CYP11B1
220	1	ENSG00000173406	DAB1
221	11	ENSG00000134780	DAGLA
222	9	ENSG00000196730	DAPK1
223	4	ENSG00000070190	DAPP1
224	9	ENSG00000123454	DBH
225	14	ENSG00000100897	DCAF11
226	16	ENSG00000166847	DCTN5
227	3	ENSG00000043093	DCUN1D1
228	X	ENSG00000077279	DCX
229	7	ENSG00000132437	DDC
230	12	ENSG00000013573	DDX11
231	X	ENSG00000215301	DDX3X
232	X	ENSG00000184735	DDX53

233	11	ENSG00000177030	DEAF1
234	12	ENSG00000139726	DENR
235	22	ENSG00000100150	DEPDC5
236	X	ENSG00000274588	DGKK
237	11	ENSG00000149091	DGKZ
238	11	ENSG00000172893	DHCR7
239	5	ENSG00000067248	DHX29
240	13	ENSG00000139734	DIAPH3
241	21	ENSG00000160305	DIP2A
242	10	ENSG00000151240	DIP2C
243	1	ENSG00000162946	DISC1
244	11	ENSG00000150764	DIXDC1
245	3	ENSG00000075711	DLG1
246	17	ENSG00000132535	DLG4
247	18	ENSG00000170579	DLGAP1
248	8	ENSG00000198010	DLGAP2
249	1	ENSG00000116544	DLGAP3
250	2	ENSG00000144355	DLX1
251	2	ENSG00000115844	DLX2
252	7	ENSG00000006377	DLX6
253	X	ENSG00000198947	DMD
254	19	ENSG00000104936	DMPK
255	15	ENSG00000104093	DMXL2
256	12	ENSG00000197653	DNAH10
257	5	ENSG00000039139	DNAH5
258	10	ENSG00000136770	DNAJC1
259	3	ENSG00000205981	DNAJC19
260	2	ENSG00000187957	DNER
261	12	ENSG00000087470	DNM1L
262	2	ENSG00000119772	DNMT3A
263	10	ENSG00000150760	DOCK1
264	2	ENSG00000135905	DOCK10
265	7	ENSG00000128512	DOCK4
266	9	ENSG00000107099	DOCK8
267	9	ENSG00000175283	DOLK
268	2	ENSG00000175497	DPP10
269	2	ENSG00000197635	DPP4
270	7	ENSG00000130226	DPP6
271	1	ENSG00000188641	DPYD
272	5	ENSG00000184845	DRD1
273	11	ENSG00000149295	DRD2
274	3	ENSG00000151577	DRD3
275	11	ENSG00000069696	DRD4
276	21	ENSG00000171587	DSCAM
277	6	ENSG00000151914	DST
278	17	ENSG00000276023	DUSP14
279	20	ENSG00000149599	DUSP15
280	6	ENSG00000112679	DUSP22

281	1	ENSG00000107404	DVL1
282	3	ENSG00000161202	DVL3
283	10	ENSG00000170788	DYDC1
284	10	ENSG00000133665	DYDC2
285	14	ENSG00000197102	DYNC1H1
286	2	ENSG00000077380	DYNC1I2
287	21	ENSG00000157540	DYRK1A
288	20	ENSG00000101210	EEF1A2
289	8	ENSG00000132294	EFR3A
290	10	ENSG00000122877	EGR2
291	9	ENSG00000181090	EHMT1
292	19	ENSG00000130811	EIF3G
293	4	ENSG00000151247	EIF4E
294	10	ENSG00000148730	EIF4EBP2
295	9	ENSG00000107105	ELAVL2
296	19	ENSG00000196361	ELAVL3
297	1	ENSG00000066322	ELOVL1
298	11	ENSG00000109911	ELP4
299	14	ENSG00000066629	EML1
300	7	ENSG00000164778	EN2
301	22	ENSG00000100393	EP300
302	12	ENSG00000183495	EP400
303	2	ENSG00000135999	EPC2
304	3	ENSG00000080224	EPHA6
305	1	ENSG00000133216	EPHB2
306	7	ENSG00000106123	EPHB6
307	12	ENSG00000151491	EPS8
308	5	ENSG00000112851	ERBB2IP
309	2	ENSG00000178568	ERBB4
310	21	ENSG00000157554	ERG
311	2	ENSG00000136541	ERMN
312	6	ENSG00000091831	ESR1
313	14	ENSG00000140009	ESR2
314	14	ENSG00000119715	ESRRB
315	19	ENSG00000105379	ETFB
316	5	ENSG00000180104	EXOC3
317	14	ENSG00000070367	EXOC5
318	10	ENSG00000138190	EXOC6
319	2	ENSG00000144036	EXOC6B
320	8	ENSG00000182197	EXT1
321	6	ENSG00000124491	F13A1
322	1	ENSG00000117525	F3
323	1	ENSG00000121769	FABP3
324	8	ENSG00000164687	FABP5
325	6	ENSG00000164434	FABP7
326	8	ENSG00000147724	FAM135B
327	X	ENSG00000185448	FAM47A
328	6	ENSG00000137414	FAM8A1

329	16	ENSG00000153789	FAM92B
330	15	ENSG00000198690	FAN1
331	4	ENSG00000083857	FAT1
332	15	ENSG00000166147	FBN1
333	4	ENSG00000118564	FBXL5
334	18	ENSG00000141665	FBXO15
335	14	ENSG00000165355	FBXO33
336	3	ENSG00000163833	FBXO40
337	19	ENSG00000186431	FCAR
338	1	ENSG00000181036	FCRL6
339	5	ENSG00000151422	FER
340	7	ENSG00000128610	FEZF1
341	3	ENSG00000153266	FEZF2
342	4	ENSG00000171560	FGA
343	X	ENSG00000102302	FGD1
344	10	ENSG00000174721	FGFBP3
345	3	ENSG00000189283	FHIT
346	13	ENSG00000102755	FLT1
347	X	ENSG00000102081	FMR1
348	11	ENSG00000086205	FOLH1
349	14	ENSG00000176165	FOXG1
350	3	ENSG00000114861	FOXP1
351	7	ENSG00000128573	FOXP2
352	4	ENSG00000183090	FREM3
353	6	ENSG00000111816	FRK
354	X	ENSG00000169933	FRMPD4
355	X	ENSG00000068438	FTSJ1
356	7	ENSG00000188763	FZD9
357	5	ENSG00000022355	GABRA1
358	X	ENSG00000011677	GABRA3
359	4	ENSG00000109158	GABRA4
360	15	ENSG00000186297	GABRA5
361	4	ENSG00000163288	GABRB1
362	15	ENSG00000166206	GABRB3
363	15	ENSG00000182256	GABRG3
364	X	ENSG00000268089	GABRQ
365	2	ENSG00000128683	GAD1
366	19	ENSG00000099860	GADD45B
367	2	ENSG00000144278	GALNT13
368	2	ENSG00000158089	GALNT14
369	19	ENSG00000130005	GAMT
370	16	ENSG00000261609	GAN
371	3	ENSG00000172020	GAP43
372	11	ENSG00000148935	GAS2
373	15	ENSG00000171766	GATM
374	9	ENSG00000119125	GDA
375	11	ENSG00000178795	GDPD4
376	7	ENSG00000146830	GIGYF1

377	2	ENSG00000204120	GIGYF2
378	1	ENSG00000174332	GLIS1
379	6	ENSG00000124767	GLO1
380	X	ENSG00000101958	GLRA2
381	X	ENSG00000182890	GLUD2
382	6	ENSG00000112312	GMNN
383	9	ENSG00000156049	GNA14
384	20	ENSG00000087460	GNAS
385	22	ENSG00000185838	GNB1L
386	3	ENSG00000173230	GOLGB1
387	X	ENSG00000076716	GPC4
388	13	ENSG00000183098	GPC6
389	2	ENSG00000115159	GPD2
390	14	ENSG00000171723	GPHN
391	8	ENSG00000020181	GPR124
392	16	ENSG00000180269	GPR139
393	7	ENSG00000170775	GPR37
394	7	ENSG00000164604	GPR85
395	X	ENSG00000158301	GPRASP2
396	3	ENSG00000233276	GPX1
397	X	ENSG00000125675	GRIA3
398	10	ENSG00000182771	GRID1
399	4	ENSG00000152208	GRID2
400	7	ENSG00000215045	GRID2IP
401	6	ENSG00000164418	GRIK2
402	1	ENSG00000163873	GRIK3
403	11	ENSG00000149403	GRIK4
404	19	ENSG00000105737	GRIK5
405	9	ENSG00000176884	GRIN1
406	16	ENSG00000183454	GRIN2A
407	12	ENSG00000273079	GRIN2B
408	19	ENSG00000116032	GRIN3B
409	12	ENSG00000155974	GRIP1
410	6	ENSG00000152822	GRM1
411	6	ENSG00000124493	GRM4
412	11	ENSG00000168959	GRM5
413	3	ENSG00000196277	GRM7
414	7	ENSG00000179603	GRM8
415	X	ENSG00000126010	GRPR
416	3	ENSG00000082701	GSK3B
417	9	ENSG00000148180	GSN
418	1	ENSG00000134184	GSTM1
419	7	ENSG00000263001	GTF2I
420	11	ENSG00000152402	GUCY1A2
421	X	ENSG00000172534	HCFC1
422	12	ENSG00000111727	HCFC2
423	5	ENSG00000164588	HCN1
424	5	ENSG00000171720	HDAC3

425	2	ENSG00000068024	HDAC4
426	X	ENSG00000094631	HDAC6
427	2	ENSG00000138411	HECW2
428	11	ENSG00000165478	HEPACAM
429	15	ENSG00000128731	HERC2
430	6	ENSG00000197409	HIST1H3D
431	6	ENSG00000278705	HIST1H4B
432	6	ENSG00000206503	HLA-A
433	6	ENSG00000234745	HLA-B
434	6	ENSG00000196126	HLA-DRB1
435	21	ENSG00000205581	HMGN1
436	X	ENSG00000126945	HNRNPH2
437	5	ENSG00000152413	HOMER1
438	7	ENSG00000105991	HOXA1
439	17	ENSG00000120094	HOXB1
440	11	ENSG00000174775	HRAS
441	6	ENSG00000249853	HS3ST5
442	1	ENSG00000117594	HSD11B1
443	6	ENSG00000135312	HTR1B
444	13	ENSG00000102468	HTR2A
445	11	ENSG00000166736	HTR3A
446	3	ENSG00000178084	HTR3C
447	10	ENSG00000148680	HTR7
448	X	ENSG00000086758	HUWE1
449	16	ENSG00000157423	HYDIN
450	7	ENSG00000003147	ICA1
451	12	ENSG00000111537	IFNG
452	6	ENSG00000027697	IFNGR1
453	11	ENSG00000167244	IGF2
454	15	ENSG00000172349	IL16
455	6	ENSG00000112115	IL17A
456	2	ENSG00000115590	IL1R2
457	X	ENSG00000169306	IL1RAPL1
458	X	ENSG00000189108	IL1RAPL2
459	7	ENSG00000136244	IL6
460	1	ENSG00000143621	ILF2
461	7	ENSG00000184903	IMMP2L
462	1	ENSG00000132849	INADL
463	2	ENSG00000151689	INPP1
464	13	ENSG00000102786	INTS6
465	X	ENSG00000124313	IQSEC2
466	14	ENSG00000119669	IRF2BPL
467	2	ENSG00000115232	ITGA4
468	12	ENSG00000161638	ITGA5
469	17	ENSG00000259207	ITGB3
470	1	ENSG00000142856	ITGB3BP
471	12	ENSG00000139626	ITGB7
472	4	ENSG00000152969	JAKMIP1

473	6	ENSG00000008083	JARID2
474	10	ENSG00000171988	JMJD1C
475	9	ENSG00000107104	KANK1
476	3	ENSG00000114166	KAT2B
477	8	ENSG00000083168	KAT6A
478	18	ENSG00000167216	KATNAL2
479	7	ENSG00000184408	KCND2
480	1	ENSG00000177807	KCNJ10
481	17	ENSG00000184185	KCNJ12
482	21	ENSG00000157551	KCNJ15
483	17	ENSG00000123700	KCNJ2
484	10	ENSG00000156113	KCNMA1
485	20	ENSG00000075043	KCNQ2
486	8	ENSG00000184156	KCNQ3
487	9	ENSG00000107147	KCNT1
488	8	ENSG00000164794	KCNV1
489	16	ENSG00000174943	KCTD13
490	19	ENSG00000127663	KDM4B
491	1	ENSG00000117139	KDM5B
492	X	ENSG00000126012	KDM5C
493	X	ENSG00000147050	KDM6A
494	17	ENSG00000132510	KDM6B
495	6	ENSG00000112232	KHDRBS2
496	8	ENSG00000131773	KHDRBS3
497	17	ENSG00000007202	KIAA0100
498	18	ENSG00000134444	KIAA1468
499	6	ENSG00000168116	KIAA1586
500	X	ENSG00000050030	KIAA2022
501	8	ENSG00000197892	KIF13B
502	2	ENSG00000168280	KIF5C
503	11	ENSG00000149571	KIRREL3
504	4	ENSG00000157404	KIT
505	11	ENSG00000174996	KLC2
506	1	ENSG00000117009	KMO
507	11	ENSG00000118058	KMT2A
508	7	ENSG00000055609	KMT2C
509	7	ENSG00000005483	KMT2E
510	19	ENSG00000118162	KPTN
511	12	ENSG00000133703	KRAS
512	12	ENSG00000111615	KRR1
513	X	ENSG00000198910	L1CAM
514	18	ENSG00000101680	LAMA1
515	7	ENSG00000091136	LAMB1
516	9	ENSG00000050555	LAMC3
517	7	ENSG00000174697	LEP
518	19	ENSG00000104863	LIN7B
519	3	ENSG00000071282	LMCD1
520	19	ENSG00000142235	LMTK3

521	9	ENSG00000136944	LMX1B
522	8	ENSG00000175445	LPL
523	4	ENSG00000198589	LRBA
524	14	ENSG00000165379	LRFN5
525	12	ENSG00000123384	LRP1
526	2	ENSG00000081479	LRP2
527	4	ENSG00000109771	LRP2BP
528	2	ENSG00000138095	LRPPRC
529	6	ENSG00000137269	LRRC1
530	1	ENSG00000033122	LRRC7
531	5	ENSG00000176018	LYSMD3
532	22	ENSG00000099949	LZTR1
533	10	ENSG00000107816	LZTS2
534	20	ENSG00000172264	MACROD2
535	X	ENSG00000179222	MAGED1
536	15	ENSG00000254585	MAGEL2
537	2	ENSG00000172005	MAL
538	X	ENSG00000189221	MAOA
539	X	ENSG00000069535	MAOB
540	2	ENSG00000078018	MAP2
541	15	ENSG00000169032	MAP2K1
542	22	ENSG00000100030	MAPK1
543	22	ENSG00000188130	MAPK12
544	16	ENSG00000102882	MAPK3
545	18	ENSG00000141639	MAPK4
546	22	ENSG00000008735	MAPK8IP2
547	17	ENSG00000186868	MAPT
548	2	ENSG00000019169	MARCO
549	1	ENSG00000116141	MARK1
550	19	ENSG00000007047	MARK4
551	18	ENSG00000141644	MBD1
552	19	ENSG00000071655	MBD3
553	3	ENSG00000129071	MBD4
554	2	ENSG00000204406	MBD5
555	12	ENSG00000166987	MBD6
556	19	ENSG00000125505	MBOAT7
557	18	ENSG00000166603	MC4R
558	5	ENSG00000171444	MCC
559	8	ENSG00000104738	MCM4
560	22	ENSG00000100297	MCM5
561	8	ENSG00000147316	MCPH1
562	14	ENSG00000139915	MDGA2
563	X	ENSG00000169057	MECP2
564	X	ENSG00000184634	MED12
565	17	ENSG00000108510	MED13
566	12	ENSG00000123066	MED13L
567	5	ENSG00000081189	MEF2C
568	15	ENSG00000157890	MEGF11

569	7	ENSG00000105976	MET
570	4	ENSG00000145388	METTL14
	11	ENSG00000235718	
571	11	ENSG00000259159	MFRP
572	18	ENSG00000101752	MIB1
573	X	ENSG00000101871	MID1
574	20	ENSG00000125863	MKKS
575	16	ENSG00000186260	MKL2
576	17	ENSG00000070444	MNT
577	18	ENSG00000075643	MOCOS
578	13	ENSG00000196199	MPHOSPH8
579	7	ENSG00000105926	MPP6
580	2	ENSG00000135900	MRPL44
581	20	ENSG00000125901	MRPS26
582	X	ENSG00000147065	MSN
583	8	ENSG00000038945	MSR1
584	1	ENSG00000188786	MTF1
585	1	ENSG00000177000	MTHFR
586	1	ENSG00000198793	MTOR
587	1	ENSG00000116984	MTR
588	2	ENSG00000128654	MTX2
589	17	ENSG00000264424	MYH4
590	13	ENSG00000041515	MYO16
591	12	ENSG00000166866	MYO1A
592	15	ENSG00000197535	MYO5A
593	19	ENSG00000099331	MYO9B
594	10	ENSG00000177791	MYOZ1
595	2	ENSG00000186487	MYT1L
596	4	ENSG00000164134	NAA15
597	3	ENSG00000177694	NAALADL2
598	11	ENSG00000166833	NAV2
599	13	ENSG00000172915	NBEA
600	2	ENSG00000061676	NCKAP1
601	2	ENSG00000176771	NCKAP5
602	12	ENSG00000167566	NCKAP5L
603	17	ENSG00000141027	NCOR1
604	15	ENSG00000185115	NDNL2
605	X	ENSG00000124479	NDP
606	7	ENSG00000128609	NDUFA5
607	3	ENSG00000136521	NDUFB5
608	8	ENSG00000277586	NEFL
609	11	ENSG00000165973	NELL1
610	15	ENSG00000067141	NEO1
611	17	ENSG00000196712	NF1
612	1	ENSG00000162599	NFIA
613	19	ENSG00000008441	NFIX
614	X	ENSG00000188158	NHS
615	2	ENSG00000196290	NIF3L1

616	20	ENSG00000101004	NINL
617	15	ENSG00000170113	NIPA1
618	15	ENSG00000140157	NIPA2
619	5	ENSG00000164190	NIPBL
620	3	ENSG00000169760	NLGN1
621	17	ENSG00000169992	NLGN2
622	X	ENSG00000196338	NLGN3
623	X	ENSG00000146938	NLGN4X
624	Y	ENSG00000165246	NLGN4Y
625	12	ENSG00000089250	NOS1
626	1	ENSG00000198929	NOS1AP
627	2	ENSG00000170485	NPAS2
628	5	ENSG00000175745	NR2F1
629	4	ENSG00000151623	NR3C2
630	7	ENSG00000091129	NRCAM
631	8	ENSG00000157168	NRG1
632	2	ENSG00000118257	NRP2
633	2	ENSG00000179915	NRXN1
634	11	ENSG00000110076	NRXN2
635	14	ENSG00000021645	NRXN3
636	5	ENSG00000165671	NSD1
637	12	ENSG00000185652	NTF3
638	19	ENSG00000225950	NTF4
639	1	ENSG00000162631	NTNG1
640	1	ENSG00000198400	NTRK1
641	15	ENSG00000140538	NTRK3
642	12	ENSG00000074590	NUAK1
643	1	ENSG00000069248	NUP133
644	X	ENSG00000126952	NXF5
645	7	ENSG00000122584	NXPH1
646	X	ENSG00000122126	OCRL
647	19	ENSG00000181781	ODF3L2
648	X	ENSG00000147162	OGT
649	X	ENSG00000079482	OPHN1
650	6	ENSG00000112038	OPRM1
651	1	ENSG00000221888	OR1C1
652	1	ENSG00000187080	OR2AK2
653	1	ENSG00000171180	OR2M4
654	1	ENSG00000184022	OR2T10
655	11	ENSG00000197790	OR52M1
656	2	ENSG00000115507	OTX1
657	20	ENSG00000101405	OXT
658	3	ENSG00000180914	OXTR
659	12	ENSG00000135124	P2RX4
660	17	ENSG00000083454	P2RX5
661	5	ENSG00000072682	P4HA2
662	11	ENSG00000175115	PACS1
663	17	ENSG00000007168	PAFAH1B1

664	12	ENSG00000171759	PAH
665	11	ENSG00000149090	PAMR1
666	1	ENSG00000116183	PAPPA2
667	2	ENSG00000116117	PARD3B
668	6	ENSG00000185345	PARK2
669	9	ENSG00000196092	PAX5
670	11	ENSG00000007372	PAX6
671	13	ENSG00000175198	PCCA
672	3	ENSG00000114054	PCCB
673	4	ENSG00000138650	PCDH10
674	X	ENSG00000102290	PCDH11X
675	10	ENSG00000150275	PCDH15
676	X	ENSG00000165194	PCDH19
677	13	ENSG00000136099	PCDH8
678	13	ENSG00000184226	PCDH9
679	5	ENSG00000204970	PCDHA1
680	5	ENSG00000250120	PCDHA10
681	5	ENSG00000249158	PCDHA11
682	5	ENSG00000251664	PCDHA12
683	5	ENSG00000239389	PCDHA13
684	5	ENSG00000204969	PCDHA2
685	5	ENSG00000255408	PCDHA3
686	5	ENSG00000204967	PCDHA4
687	5	ENSG00000204965	PCDHA5
688	5	ENSG00000081842	PCDHA6
689	5	ENSG00000204963	PCDHA7
690	5	ENSG00000204962	PCDHA8
691	5	ENSG00000204961	PCDHA9
692	5	ENSG00000248383	PCDHAC1
693	5	ENSG00000243232	PCDHAC2
694	5	ENSG00000253873	PCDHGA11
695	20	ENSG00000100982	PCIF1
696	14	ENSG00000100731	PCNX
697	7	ENSG00000106333	PCOLCE
698	7	ENSG00000154678	PDE1C
699	19	ENSG00000065989	PDE4A
700	1	ENSG00000184588	PDE4B
701	10	ENSG00000107438	PDLIM1
702	X	ENSG00000067840	PDZD4
703	2	ENSG00000115425	PECR
704	17	ENSG00000179094	PER1
705	6	ENSG00000112357	PEX7
706	19	ENSG00000161031	PGLYRP2
707	9	ENSG00000197724	PHF2
708	6	ENSG00000118482	PHF3
709	X	ENSG00000172943	PHF8
710	6	ENSG00000146247	PHIP
711	11	ENSG00000070047	PHRF1

712	7	ENSG00000105851	PIK3CG
	19	ENSG00000105647	
713	19	ENSG00000268173	PIK3R2
714	8	ENSG00000254093	PINX1
715	X	ENSG00000087842	PIR
716	5	ENSG00000069011	PITX1
717	19	ENSG00000011422	PLAUR
718	20	ENSG00000182621	PLCB1
719	3	ENSG00000187091	PLCD1
720	6	ENSG00000198523	PLN
721	7	ENSG00000221866	PLXNA4
722	1	ENSG00000143442	POGZ
723	1	ENSG00000085998	POMGNT1
724	7	ENSG00000005421	PON1
725	7	ENSG00000128513	POT1
726	6	ENSG00000184486	POU3F2
727	11	ENSG00000131626	PPFIA1
728	17	ENSG00000170836	PPM1D
729	17	ENSG00000131771	PPP1R1B
730	X	ENSG00000049769	PPP1R3F
731	11	ENSG00000137713	PPP2R1B
732	6	ENSG00000112640	PPP2R5D
733	X	ENSG00000102103	PQBP1
734	20	ENSG00000124126	PREX1
735	12	ENSG00000139174	PRICKLE1
736	3	ENSG00000163637	PRICKLE2
737	6	ENSG00000146143	PRIM2
738	16	ENSG00000166501	PRKCB
739	14	ENSG00000184304	PRKD1
740	8	ENSG00000253729	PRKDC
741	X	ENSG00000183943	PRKX
742	22	ENSG00000100033	PRODH
743	14	ENSG00000185246	PRPF39
744	1	ENSG00000185888	PRSS38
745	9	ENSG00000106772	PRUNE2
746	8	ENSG00000156011	PSD3
747	X	ENSG00000101843	PSMD10
748	18	ENSG00000128789	PSMG2
749	1	ENSG00000117569	PTBP2
750	X	ENSG00000165186	PTCHD1
751	10	ENSG00000171862	PTEN
752	1	ENSG00000050628	PTGER3
753	1	ENSG00000073756	PTGS2
754	6	ENSG00000112655	PTK7
755	12	ENSG00000179295	PTPN11
756	12	ENSG00000127329	PTPRB
757	1	ENSG00000081237	PTPRC
758	20	ENSG00000196090	PTPRT

759	7	ENSG00000106278	PTPRZ1
760	22	ENSG00000100362	PVALB
761	2	ENSG00000130508	PXDN
762	1	ENSG00000163564	PYHIN1
763	2	ENSG00000135631	RAB11FIP5
764	7	ENSG00000146955	RAB19
765	8	ENSG00000104388	RAB2A
766	X	ENSG00000155961	RAB39B
767	3	ENSG00000172780	RAB43
768	8	ENSG00000164754	RAD21
769	12	ENSG00000111247	RAD51AP1
770	17	ENSG00000108557	RAI1
771	5	ENSG00000204764	RANBP17
772	2	ENSG00000091428	RAPGEF4
773	17	ENSG00000108551	RASD1
774	1	ENSG00000266094	RASSF5
775	8	ENSG00000023287	RB1CC1
776	20	ENSG00000089050	RBBP9
777	16	ENSG00000078328	RBFOX1
778	5	ENSG00000091009	RBM27
779	1	ENSG00000265241	RBM8A
780	3	ENSG00000144642	RBMS3
781	10	ENSG00000165476	REEP3
782	7	ENSG00000189056	RELN
783	1	ENSG00000142599	RERE
784	19	ENSG00000079313	REXO1
785	1	ENSG00000143207	RFWD2
786	1	ENSG00000182901	RGS7
787	X	ENSG00000101883	RHOXF1
788	6	ENSG00000079841	RIMS1
789	1	ENSG00000117016	RIMS3
790	8	ENSG00000104312	RIPK2
791	18	ENSG00000152214	RIT2
792	X	ENSG00000131263	RLIM
793	17	ENSG00000181481	RNF135
794	3	ENSG00000163961	RNF168
795	9	ENSG00000137075	RNF38
796	16	ENSG00000205937	RNPS1
797	3	ENSG00000169855	ROBO1
798	3	ENSG00000185008	ROBO2
799	15	ENSG00000069667	RORA
800	1	ENSG00000116745	RPE65
801	16	ENSG00000103494	RPGRIP1L
802	X	ENSG00000147403	RPL10
803	15	ENSG00000178718	RPP25
804	10	ENSG00000138326	RPS24
805	6	ENSG00000071242	RPS6KA2
806	X	ENSG00000177189	RPS6KA3

807	10	ENSG00000148484	RSU1
808	19	ENSG00000142230	SAE1
809	2	ENSG00000119042	SATB2
810	22	ENSG00000100241	SBF1
811	10	ENSG00000099194	SCD
812	4	ENSG00000184178	SCFD2
813	2	ENSG00000144285	SCN1A
814	2	ENSG00000136531	SCN2A
815	17	ENSG00000007314	SCN4A
816	3	ENSG00000183873	SCN5A
817	2	ENSG00000136546	SCN7A
818	12	ENSG00000196876	SCN8A
819	1	ENSG00000116171	SCP2
820	8	ENSG00000169439	SDC2
821	7	ENSG00000146555	SDK1
822	5	ENSG00000112902	SEMA5A
823	5	ENSG00000164300	SERINC5
824	7	ENSG00000106366	SERPINE1
825	18	ENSG00000152217	SETBP1
826	3	ENSG00000181555	SETD2
827	3	ENSG00000168137	SETD5
828	1	ENSG00000143379	SETDB1
829	13	ENSG00000136169	SETDB2
830	17	ENSG00000063015	SEZ6
831	16	ENSG00000174938	SEZ6L2
832	17	ENSG00000181523	SGSH
833	22	ENSG00000100359	SGSM3
834	X	ENSG00000147010	SH3KBP1
835	19	ENSG00000161681	SHANK1
836	11	ENSG00000162105	SHANK2
837	22	ENSG00000251322	SHANK3
838	X	ENSG00000185960	SHOX
839	21	ENSG00000142178	SIK1
840	15	ENSG00000169375	SIN3A
841	20	ENSG00000124140	SLC12A5
842	17	ENSG00000141526	SLC16A3
843	12	ENSG00000118596	SLC16A7
844	9	ENSG00000106688	SLC1A1
845	11	ENSG00000110436	SLC1A2
846	1	ENSG00000163393	SLC22A15
847	11	ENSG00000149742	SLC22A9
848	9	ENSG00000155886	SLC24A2
849	2	ENSG00000115840	SLC25A12
850	X	ENSG00000102078	SLC25A14
851	1	ENSG00000085491	SLC25A24
852	6	ENSG00000153291	SLC25A27
853	9	ENSG00000167114	SLC27A4
854	7	ENSG00000164638	SLC29A4

855	2	ENSG00000115194	SLC30A3
856	5	ENSG00000145740	SLC30A5
857	3	ENSG00000169359	SLC33A1
858	1	ENSG00000117620	SLC35A3
859	17	ENSG00000157637	SLC38A10
860	17	ENSG00000133195	SLC39A11
861	2	ENSG00000144290	SLC4A10
862	3	ENSG00000157103	SLC6A1
863	5	ENSG00000142319	SLC6A3
864	17	ENSG00000108576	SLC6A4
865	X	ENSG00000130821	SLC6A8
866	4	ENSG00000151012	SLC7A11
867	X	ENSG00000165349	SLC7A3
868	19	ENSG00000118160	SLC8A2
869	X	ENSG00000198689	SLC9A6
870	3	ENSG00000181804	SLC9A9
871	12	ENSG00000111700	SLCO1B3
872	12	ENSG00000139155	SLCO1C1
873	5	ENSG00000184347	SLIT3
874	13	ENSG00000165300	SLITRK5
875	6	ENSG00000112305	SMAP1
876	9	ENSG00000080503	SMARCA2
877	12	ENSG00000139613	SMARCC2
878	10	ENSG00000108055	SMC3
879	17	ENSG00000070366	SMG6
880	1	ENSG00000116698	SMG7
881	16	ENSG00000103056	SMPD3
882	20	ENSG00000132639	SNAP25
883	7	ENSG00000197157	SND1
884	15	ENSG00000128739	SNRPN
885	2	ENSG00000172554	SNTG2
886	6	ENSG00000135317	SNX14
887	11	ENSG00000120451	SNX19
888	21	ENSG00000142168	SOD1
889	12	ENSG00000134532	SOX5
890	1	ENSG00000155761	SPAG17
891	4	ENSG00000152583	SPARCL1
892	2	ENSG00000021574	SPAST
893	4	ENSG00000164056	SPRY1
894	2	ENSG00000277893	SRD5A2
895	12	ENSG00000139767	SRRM4
896	1	ENSG00000116754	SRSF11
897	7	ENSG00000197558	SSPO
898	7	ENSG00000004866	ST7
899	15	ENSG00000140557	ST8SIA2
900	2	ENSG00000115415	STAT1
901	2	ENSG00000198648	STK39
902	7	ENSG00000106089	STX1A

903	9	ENSG00000136854	STXBP1
904	6	ENSG00000164506	STXBP5
905	12	ENSG00000060140	STYK1
906	3	ENSG00000172340	SUCLG2
907	11	ENSG00000110066	SUV420H1
908	X	ENSG00000169895	SYAP1
909	X	ENSG00000008056	SYN1
910	3	ENSG00000157152	SYN2
911	22	ENSG00000185666	SYN3
912	6	ENSG00000131018	SYNE1
913	6	ENSG00000197283	SYNGAP1
914	16	ENSG00000103528	SYT17
915	19	ENSG00000213023	SYT3
916	X	ENSG00000102362	SYTL4
917	X	ENSG00000147133	TAF1
918	16	ENSG00000103168	TAF1C
919	9	ENSG00000122728	TAF1L
920	7	ENSG00000106290	TAF6
921	8	ENSG00000156787	TBC1D31
922	3	ENSG00000131374	TBC1D5
923	6	ENSG00000145979	TBC1D7
924	X	ENSG00000101849	TBL1X
925	3	ENSG00000177565	TBL1XR1
926	2	ENSG00000136535	TBR1
927	22	ENSG00000184058	TBX1
928	22	ENSG00000100207	TCF20
929	19	ENSG00000071564	TCF3
930	18	ENSG00000196628	TCF4
931	10	ENSG00000148737	TCF7L2
932	4	ENSG00000151790	TDO2
933	16	ENSG00000132604	TERF2
934	5	ENSG00000164362	TERT
935	4	ENSG00000168769	TET2
936	20	ENSG00000125780	TGM3
937	11	ENSG00000180176	TH
938	19	ENSG00000161277	THAP8
939	15	ENSG00000137801	THBS1
940	17	ENSG00000126351	THRA
941	17	ENSG00000146872	TLK2
942	2	ENSG00000168955	TM4SF20
943	16	ENSG00000205084	TMEM231
944	X	ENSG00000185973	TMLHE
945	9	ENSG00000041982	TNC
946	4	ENSG00000168884	TNIP2
947	1	ENSG00000120332	TNN
948	7	ENSG00000064419	TNPO3
949	22	ENSG00000100354	TNRC6B
950	1	ENSG00000173726	TOMM20

951	20	ENSG00000198900	TOP1
952	22	ENSG00000100038	TOP3B
953	12	ENSG00000139287	TPH2
954	2	ENSG00000115705	TPO
955	2	ENSG00000171853	TRAPPC12
956	1	ENSG00000197323	TRIM33
957	5	ENSG00000038382	TRIO
958	2	ENSG00000153827	TRIP12
959	1	ENSG00000116747	TROVE2
960	11	ENSG00000137672	TRPC6
961	15	ENSG00000134160	TRPM1
962	9	ENSG00000165699	TSC1
963	16	ENSG00000103197	TSC2
964	19	ENSG00000121297	TSHZ3
965	2	ENSG00000211460	TSN
966	X	ENSG00000156298	TSPAN7
967	17	ENSG00000167721	TSR1
968	8	ENSG00000129696	TTI2
969	2	ENSG00000155657	TTN
970	12	ENSG00000167552	TUBA1A
971	15	ENSG00000275835	TUBGCP5
972	11	ENSG00000077498	TYR
973	4	ENSG00000033178	UBA6
974	7	ENSG00000186591	UBE2H
975	15	ENSG00000114062	UBE3A
976	12	ENSG00000151148	UBE3B
977	7	ENSG00000009335	UBE3C
978	15	ENSG00000138629	UBL7
979	7	ENSG00000157741	UBN2
980	2	ENSG00000144357	UBR3
981	8	ENSG00000104517	UBR5
982	14	ENSG00000012963	UBR7
983	10	ENSG00000178473	UCN3
984	9	ENSG00000198722	UNC13B
985	2	ENSG00000144406	UNC80
986	22	ENSG00000100024	UPB1
987	10	ENSG00000151461	UPF2
988	X	ENSG00000125351	UPF3B
989	2	ENSG00000007001	UPP2
990	6	ENSG00000123552	USP45
991	16	ENSG00000187555	USP7
992	Y	ENSG00000114374	USP9Y
993	6	ENSG00000152818	UTRN
994	14	ENSG00000071246	VASH1
995	9	ENSG00000165280	VCP
996	2	ENSG00000127831	VIL1
997	6	ENSG00000146469	VIP
998	9	ENSG00000147852	VLDLR

999	8	ENSG00000132549	VPS13B
1000	X	ENSG00000155659	VSIG4
1001	10	ENSG00000095787	WAC
1002	4	ENSG00000163625	WDFY3
1003	15	ENSG00000140527	WDR93
1004	12	ENSG00000156076	WIF1
1005	X	ENSG00000196632	WNK3
1006	12	ENSG00000125084	WNT1
1007	7	ENSG00000105989	WNT2
1008	16	ENSG00000186153	WWOX
1009	3	ENSG00000168334	XIRP1
1010	3	ENSG00000154767	XPC
1011	2	ENSG00000082898	XPO1
1012	3	ENSG00000163872	YEATS2
1013	5	ENSG00000047188	YTHDC2
1014	17	ENSG00000108953	YWHAE
1015	11	ENSG00000109906	ZBTB16
1016	3	ENSG00000181722	ZBTB20
1017	7	ENSG00000178381	ZFAND2A
1018	14	ENSG00000072121	ZFYVE26
1019	13	ENSG00000121741	ZMYM2
1020	10	ENSG00000015171	ZMYND11
1021	19	ENSG00000105497	ZNF175
1022	17	ENSG00000154957	ZNF18
1023	6	ENSG00000188994	ZNF292
1024	10	ENSG00000189180	ZNF33A
1025	2	ENSG00000144331	ZNF385B
1026	18	ENSG00000215421	ZNF407
1027	14	ENSG00000119725	ZNF410
1028	9	ENSG00000148143	ZNF462
1029	8	ENSG00000197363	ZNF517
1030	19	ENSG00000188321	ZNF559
1031	7	ENSG00000178665	ZNF713
1032	15	ENSG00000196391	ZNF774
1033	19	ENSG00000278129	ZNF8
1034	2	ENSG00000170396	ZNF804A
1035	4	ENSG00000151612	ZNF827
1036	1	ENSG00000162415	ZSWIM5
1037	15	ENSG00000174442	ZWILCH

Intellectual Disability

Gene Number	Chromosome	Gene ID	Gene Name
1	12	ENSG00000094914	AAAS
2	15	ENSG00000103591	AAGAB
3	6	ENSG00000124608	AARS2
4	7	ENSG00000008311	AASS
5	9	ENSG00000165029	ABCA1

6	7	ENSG00000179869	ABCA13
7	1	ENSG00000198691	ABCA4
8	2	ENSG00000073734	ABCB11
9	7	ENSG00000004846	ABCB5
10	X	ENSG00000131269	ABCB7
11	17	ENSG00000108846	ABCC3
12	16	ENSG00000091262	ABCC6
13	11	ENSG00000006071	ABCC8
14	12	ENSG00000069431	ABCC9
15	X	ENSG00000101986	ABCD1
16	12	ENSG00000173208	ABCD2
17	14	ENSG00000119688	ABCD4
18	4	ENSG00000118777	ABCG2
19	3	ENSG00000011198	ABHD5
20	17	ENSG00000278540	ACACA
21	12	ENSG00000076555	ACACB
22	3	ENSG00000177646	ACAD9
23	1	ENSG00000117054	ACADM
24	12	ENSG00000122971	ACADS
25	10	ENSG00000196177	ACADSB
26	17	ENSG00000072778	ACADVL
27	3	ENSG00000114331	ACAP2
28	11	ENSG00000075239	ACAT1
29	22	ENSG00000100412	ACO2
30	17	ENSG00000161533	ACOX1
31	19	ENSG00000102575	ACP5
32	19	ENSG00000142513	ACPT
33	22	ENSG00000100312	ACR
34	16	ENSG00000176715	ACSF3
35	X	ENSG00000068366	ACSL4
36	7	ENSG00000075624	ACTB
37	15	ENSG00000159251	ACTC1
38	17	ENSG00000184009	ACTG1
39	7	ENSG00000077080	ACTL6B
40	14	ENSG00000072110	ACTN1
41	19	ENSG00000130402	ACTN4
42	1	ENSG00000169717	ACTRT2
43	2	ENSG00000115170	ACVR1
44	3	ENSG00000243989	ACY1
45	20	ENSG00000196839	ADA
46	1	ENSG00000134249	ADAM30
47	19	ENSG00000142303	ADAMTS10
48	3	ENSG00000163638	ADAMTS9
49	9	ENSG00000178031	ADAMTSL1
50	9	ENSG00000197859	ADAMTSL2
51	1	ENSG00000160710	ADAR
52	19	ENSG00000213638	ADAT3
53	1	ENSG00000163050	ADCK3

54	8	ENSG00000173137	ADCK5
55	5	ENSG00000078295	ADCY2
56	2	ENSG00000138031	ADCY3
57	3	ENSG00000173175	ADCY5
58	4	ENSG00000087274	ADD1
59	4	ENSG00000198099	ADH4
60	10	ENSG00000156110	ADK
61	20	ENSG00000101126	ADNP
62	2	ENSG00000274286	ADRA2B
63	22	ENSG00000239900	ADSL
64	4	ENSG00000196526	AFAP1
	X	ENSG00000155966	
65	X	ENSG00000281817	AFF2
66	2	ENSG00000144218	AFF3
67	5	ENSG00000072364	AFF4
68	18	ENSG00000141385	AFG3L2
69	4	ENSG00000038002	AGA
70	2	ENSG00000157985	AGAP1
71	6	ENSG00000204305	AGER
72	7	ENSG00000006530	AGK
73	1	ENSG00000162688	AGL
74	9	ENSG00000169692	AGPAT2
75	2	ENSG00000018510	AGPS
76	X	ENSG00000180772	AGTR2
77	20	ENSG00000101444	AHCY
78	1	ENSG00000126705	AHDC1
79	6	ENSG00000135541	AHI1
80	X	ENSG00000156709	AIFM1
81	22	ENSG00000183773	AIFM3
82	4	ENSG00000164022	AIMP1
83	11	ENSG00000110711	AIP
84	17	ENSG00000129221	AIPL1
85	9	ENSG00000106992	AK1
86	1	ENSG00000004455	AK2
87	9	ENSG00000106948	AKNA
88	1	ENSG00000162482	AKR7A3
89	14	ENSG00000142208	AKT1
90	1	ENSG00000117020	AKT3
91	10	ENSG00000059573	ALDH18A1
92	15	ENSG00000184254	ALDH1A3
93	9	ENSG00000137124	ALDH1B1
94	3	ENSG00000144908	ALDH1L1
95	17	ENSG00000072210	ALDH3A2
96	1	ENSG00000159423	ALDH4A1
97	6	ENSG00000112294	ALDH5A1
98	5	ENSG00000164904	ALDH7A1
99	1	ENSG00000143149	ALDH9A1
100	16	ENSG00000149925	ALDOA

101	9	ENSG00000136872	ALDOB
102	16	ENSG00000033011	ALG1
103	22	ENSG00000182858	ALG12
104	X	ENSG00000101901	ALG13
105	9	ENSG00000119523	ALG2
106	3	ENSG00000214160	ALG3
107	1	ENSG00000088035	ALG6
108	11	ENSG00000159063	ALG8
109	2	ENSG00000116127	ALMS1
110	2	ENSG00000163295	ALPI
111	1	ENSG00000162551	ALPL
112	3	ENSG00000178038	ALS2CL
113	12	ENSG00000180318	ALX1
114	1	ENSG00000156150	ALX3
115	11	ENSG00000052850	ALX4
116	X	ENSG00000184675	AMER1
117	1	ENSG00000116337	AMPD2
118	3	ENSG00000145020	AMT
119	4	ENSG00000145362	ANK2
120	10	ENSG00000151150	ANK3
121	5	ENSG00000154122	ANKH
122	16	ENSG00000167522	ANKRD11
123	4	ENSG00000132466	ANKRD17
124	4	ENSG00000151458	ANKRD50
125	3	ENSG00000160746	ANO10
126	11	ENSG00000171714	ANO5
127	2	ENSG00000169604	ANTXR1
128	17	ENSG00000131480	AOC2
129	7	ENSG00000106367	AP1S1
130	X	ENSG00000182287	AP1S2
131	5	ENSG00000132842	AP3B1
132	1	ENSG00000134262	AP4B1
133	15	ENSG00000081014	AP4E1
134	7	ENSG00000221838	AP4M1
135	14	ENSG00000100478	AP4S1
136	12	ENSG00000120868	APAF1
137	11	ENSG00000166181	API5
138	19	ENSG00000105290	APLP1
139	22	ENSG00000100336	APOL4
140	14	ENSG00000256053	APOPT1
141	9	ENSG00000137074	APTX
142	12	ENSG00000167580	AQP2
143	11	ENSG00000186635	ARAP1
144	8	ENSG00000066777	ARFGEF1
145	20	ENSG00000124198	ARFGEF2
146	6	ENSG00000118520	ARG1
147	2	ENSG00000075884	ARHGAP15
148	10	ENSG00000213390	ARHGAP19

149	11	ENSG00000137727	ARHGAP20
150	2	ENSG00000163219	ARHGAP25
151	5	ENSG00000145819	ARHGAP26
152	3	ENSG00000031081	ARHGAP31
153	19	ENSG00000160007	ARHGAP35
154	20	ENSG00000124143	ARHGAP40
155	17	ENSG00000141522	ARHGDIA
156	3	ENSG00000114790	ARHGEF26
157	X	ENSG00000129675	ARHGEF6
158	X	ENSG00000131089	ARHGEF9
159	1	ENSG00000117713	ARID1A
160	6	ENSG00000049618	ARID1B
161	3	ENSG00000113966	ARL6
162	10	ENSG00000165309	ARMC3
163	10	ENSG00000169126	ARMC4
164	15	ENSG00000172379	ARNT2
165	17	ENSG00000141480	ARRB2
166	15	ENSG00000140450	ARRDC4
167	22	ENSG00000100299	ARSA
168	5	ENSG00000113273	ARSB
169	X	ENSG00000157399	ARSE
170	X	ENSG00000004848	ARX
171	8	ENSG00000104763	ASAH1
172	2	ENSG00000151693	ASAP2
173	17	ENSG00000161664	ASB16
174	7	ENSG00000126522	ASL
175	17	ENSG00000108381	ASPA
176	8	ENSG00000198363	ASPH
177	1	ENSG00000066279	ASPM
178	11	ENSG00000162174	ASRGL1
179	9	ENSG00000130707	ASS1
180	3	ENSG00000034533	ASTE1
181	20	ENSG00000171456	ASXL1
182	2	ENSG00000119778	ATAD2B
183	12	ENSG00000170653	ATF7
184	16	ENSG00000166669	ATF7IP2
185	5	ENSG00000152348	ATG10
186	2	ENSG00000138363	ATIC
187	14	ENSG00000198513	ATL1
188	11	ENSG00000149311	ATM
189	12	ENSG00000111676	ATN1
190	5	ENSG00000118322	ATP10B
191	X	ENSG00000101974	ATP11C
192	1	ENSG00000163399	ATP1A1
193	1	ENSG00000018625	ATP1A2
194	19	ENSG00000105409	ATP1A3
195	12	ENSG00000174437	ATP2A2
196	12	ENSG00000070961	ATP2B1

197	X	ENSG00000182220	ATP6AP2
198	12	ENSG00000185344	ATP6V0A2
199	2	ENSG00000116039	ATP6V1B1
200	8	ENSG00000147416	ATP6V1B2
201	X	ENSG00000165240	ATP7A
202	4	ENSG00000124406	ATP8A1
203	13	ENSG00000132932	ATP8A2
204	18	ENSG00000081923	ATP8B1
205	3	ENSG00000175054	ATR
206	X	ENSG00000085224	ATRX
207	6	ENSG00000124788	ATXN1
208	22	ENSG00000130638	ATXN10
209	16	ENSG00000168488	ATXN2L
210	14	ENSG00000066427	ATXN3
211	9	ENSG00000148090	AUH
212	7	ENSG00000158321	AUTS2
213	X	ENSG00000126895	AVPR2
214	1	ENSG00000162885	B3GALNT2
215	1	ENSG00000176022	B3GALT6
216	13	ENSG00000187676	B3GALTL
217	12	ENSG00000139044	B4GALNT3
218	11	ENSG00000182272	B4GALNT4
219	9	ENSG00000086062	B4GALT1
220	5	ENSG00000027847	B4GALT7
221	11	ENSG00000186318	BACE1
222	1	ENSG00000121753	BAI2
223	2	ENSG00000138376	BARD1
224	14	ENSG00000198604	BAZ1A
225	2	ENSG00000123636	BAZ2B
226	10	ENSG00000214413	BBIP1
227	11	ENSG00000174483	BBS1
228	12	ENSG00000179941	BBS10
229	4	ENSG00000181004	BBS12
230	16	ENSG00000125124	BBS2
231	15	ENSG00000140463	BBS4
232	2	ENSG00000163093	BBS5
233	4	ENSG00000138686	BBS7
234	7	ENSG00000122507	BBS9
235	3	ENSG00000114439	BBX
236	19	ENSG00000187244	BCAM
237	X	ENSG00000185825	BCAP31
238	20	ENSG00000064787	BCAS1
239	12	ENSG00000060982	BCAT1
240	19	ENSG00000248098	BCKDHA
241	6	ENSG00000083123	BCKDHB
242	2	ENSG00000119866	BCL11A
243	11	ENSG00000186174	BCL9L
244	6	ENSG00000029363	BCLAF1

245	X	ENSG00000183337	BCOR
246	X	ENSG00000085185	BCORL1
247	2	ENSG00000074582	BCS1L
248	20	ENSG00000125864	BFSP1
249	3	ENSG00000170819	BFSP2
250	3	ENSG00000134107	BHLHE40
251	9	ENSG00000185963	BICD2
252	2	ENSG00000136717	BIN1
253	2	ENSG00000115760	BIRC6
254	15	ENSG00000197299	BLM
255	14	ENSG00000125378	BMP4
256	7	ENSG00000164619	BMPER
257	4	ENSG00000138696	BMPR1B
258	9	ENSG00000173068	BNC2
259	20	ENSG00000167104	BPIFB6
260	7	ENSG00000157764	BRAF
261	13	ENSG00000139618	BRCA2
262	14	ENSG00000185024	BRF1
263	17	ENSG00000136492	BRIP1
264	21	ENSG00000185658	BRWD1
265	X	ENSG00000165288	BRWD3
266	11	ENSG00000168000	BSCL2
267	1	ENSG00000162399	BSND
268	6	ENSG00000183826	BTBD9
269	3	ENSG00000169814	BTD
270	6	ENSG00000124557	BTN1A1
271	5	ENSG00000113303	BTNL8
272	15	ENSG00000156970	BUB1B
273	10	ENSG00000148655	C10orf11
274	10	ENSG00000107815	C10orf2
275	12	ENSG00000111678	C12orf57
276	12	ENSG00000130921	C12orf65
277	17	ENSG00000181013	C17orf47
278	18	ENSG00000206043	C18orf63
279	19	ENSG00000131944	C19orf40
280	19	ENSG00000132016	C19orf57
281	1	ENSG00000162384	C1orf123
282	1	ENSG00000203963	C1orf141
283	20	ENSG00000125531	C20orf195
284	11	ENSG00000168014	C2CD3
285	2	ENSG00000115998	C2orf42
286	2	ENSG00000179270	C2orf71
287	1	ENSG00000123838	C4BPA
288	4	ENSG00000174792	C4orf26
289	4	ENSG00000181215	C4orf50
290	5	ENSG00000197603	C5orf42
291	6	ENSG00000204296	C6orf10
292	8	ENSG00000156172	C8orf37

293	8	ENSG00000104267	CA2
294	X	ENSG00000169239	CA5B
295	8	ENSG00000178538	CA8
296	19	ENSG00000141837	CACNA1A
297	12	ENSG00000151067	CACNA1C
298	3	ENSG00000157388	CACNA1D
299	1	ENSG00000198216	CACNA1E
300	17	ENSG00000006283	CACNA1G
301	16	ENSG00000196557	CACNA1H
302	16	ENSG00000006116	CACNG3
303	14	ENSG00000198668	CALM1
304	15	ENSG00000129007	CALML4
305	5	ENSG00000070808	CAMK2A
306	7	ENSG00000058404	CAMK2B
307	19	ENSG00000076826	CAMSAP3
308	1	ENSG00000171735	CAMTA1
309	17	ENSG00000171302	CANT1
310	1	ENSG00000203697	CAPN8
311	1	ENSG00000116489	CAPZA1
312	17	ENSG00000141527	CARD14
313	12	ENSG00000118307	CASC1
314	15	ENSG00000137812	CASC5
315	X	ENSG00000147044	CASK
316	17	ENSG00000177303	CASKIN2
317	10	ENSG00000165806	CASP7
318	1	ENSG00000132906	CASP9
319	16	ENSG00000129993	CBFA2T3
320	11	ENSG00000110395	CBL
321	7	ENSG00000105879	CBLL1
	21	ENSG00000160200	
322	21	ENSG00000274276	CBS
323	12	ENSG00000094916	CBX5
324	19	ENSG00000132024	CC2D1A
325	4	ENSG00000048342	CC2D2A
326	18	ENSG00000183287	CCBE1
327	17	ENSG00000167131	CCDC103
328	19	ENSG00000105479	CCDC114
329	7	ENSG00000004766	CCDC132
330	1	ENSG00000122483	CCDC18
331	X	ENSG00000101997	CCDC22
332	1	ENSG00000160050	CCDC28B
333	3	ENSG00000173421	CCDC36
334	3	ENSG00000145075	CCDC39
335	17	ENSG00000141519	CCDC40
336	12	ENSG00000139537	CCDC65
337	16	ENSG00000162004	CCDC78
338	2	ENSG00000115355	CCDC88A
339	14	ENSG00000015133	CCDC88C

340	11	ENSG00000179071	CCDC89
341	6	ENSG00000112576	CCND3
342	1	ENSG00000221978	CCNL2
343	12	ENSG00000166226	CCT2
344	1	ENSG00000134256	CD101
345	1	ENSG00000122223	CD244
346	9	ENSG00000120217	CD274
347	17	ENSG00000186074	CD300LF
348	19	ENSG00000117877	CD3EAP
349	20	ENSG00000101017	CD40
350	1	ENSG00000116815	CD58
351	13	ENSG00000102543	CDADC1
352	15	ENSG00000140326	CDAN1
353	1	ENSG00000143776	CDC42BPA
354	14	ENSG00000198752	CDC42BPB
355	17	ENSG00000094804	CDC6
356	16	ENSG00000129910	CDH15
357	10	ENSG00000107736	CDH23
358	16	ENSG00000062038	CDH3
359	16	ENSG00000185324	CDK10
360	7	ENSG00000065883	CDK13
361	20	ENSG00000101391	CDK5RAP1
362	9	ENSG00000136861	CDK5RAP2
363	7	ENSG00000105810	CDK6
364	6	ENSG00000145996	CDKAL1
365	2	ENSG00000205111	CDKL4
366	X	ENSG00000008086	CDKL5
367	11	ENSG00000129757	CDKN1C
368	11	ENSG00000064309	CDON
369	16	ENSG00000167513	CDT1
370	16	ENSG00000166446	CDYL2
371	19	ENSG00000007129	CEACAM21
372	19	ENSG00000105352	CEACAM4
373	19	ENSG00000086548	CEACAM6
374	22	ENSG00000075275	CELSR1
375	1	ENSG00000117724	CENPF
376	13	ENSG00000151849	CENPJ
377	4	ENSG00000174799	CEP135
378	15	ENSG00000103995	CEP152
379	11	ENSG00000110274	CEP164
380	3	ENSG00000174007	CEP19
381	18	ENSG00000101639	CEP192
382	12	ENSG00000198707	CEP290
383	7	ENSG00000106477	CEP41
384	11	ENSG00000166037	CEP57
385	3	ENSG00000182923	CEP63
386	12	ENSG00000173588	CEP83
387	1	ENSG00000130695	CEP85

388	17	ENSG00000258890	CEP95
389	19	ENSG00000223802	CERS1
390	15	ENSG00000128849	CGNL1
391	19	ENSG00000167670	CHAF1A
392	13	ENSG00000198824	CHAMP1
393	10	ENSG00000070748	CHAT
394	15	ENSG00000173575	CHD2
395	17	ENSG00000170004	CHD3
396	12	ENSG00000111642	CHD4
397	8	ENSG00000171316	CHD7
398	11	ENSG00000149554	CHEK1
399	22	ENSG00000100288	CHKB
400	X	ENSG00000188419	CHM
401	16	ENSG00000131165	CHMP1A
402	X	ENSG00000101938	CHRDL1
403	11	ENSG00000180720	CHRM4
404	11	ENSG00000129749	CHRNA10
405	20	ENSG00000101204	CHRNA4
406	2	ENSG00000135902	CHRND
407	15	ENSG00000169105	CHST14
408	10	ENSG00000122863	CHST3
409	15	ENSG00000131873	CHSY1
410	10	ENSG00000213341	CHUK
411	15	ENSG00000136425	CIB2
412	12	ENSG00000122966	CIT
413	2	ENSG00000169607	CKAP2L
414	1	ENSG00000016490	CLCA1
415	X	ENSG00000073464	CLCN4
416	1	ENSG00000011021	CLCN6
417	16	ENSG00000103249	CLCN7
418	1	ENSG00000186510	CLCNKA
419	1	ENSG00000184908	CLCNKB
420	21	ENSG00000159261	CLDN14
421	1	ENSG00000164007	CLDN19
422	X	ENSG00000155962	CLIC2
	16	ENSG00000188603	
423	16	ENSG00000261832	CLN3
424	13	ENSG00000102805	CLN5
425	8	ENSG00000182372	CLN8
426	4	ENSG00000109684	CLNK
427	11	ENSG00000162129	CLPB
428	19	ENSG00000125656	CLPP
429	15	ENSG00000166855	CLPX
430	4	ENSG00000249581	CLRN2
431	12	ENSG00000139182	CLSTN3
432	17	ENSG00000141367	CLTC
433	22	ENSG00000070371	CLTCL1
434	16	ENSG00000153815	CMIP

435	3	ENSG00000169714	CNBP
436	X	ENSG00000149970	CNKSR2
437	19	ENSG00000064666	CNN2
438	10	ENSG00000148842	CNNM2
439	2	ENSG00000158158	CNNM4
440	16	ENSG00000125107	CNOT1
441	12	ENSG00000111596	CNOT2
442	19	ENSG00000088038	CNOT3
443	5	ENSG00000155508	CNOT8
444	11	ENSG00000149972	CNTN5
445	7	ENSG00000174469	CNTNAP2
446	16	ENSG00000152910	CNTNAP4
447	2	ENSG00000183513	COA5
448	17	ENSG00000166685	COG1
449	16	ENSG00000103051	COG4
450	13	ENSG00000133103	COG6
451	16	ENSG00000168434	COG7
452	16	ENSG00000213380	COG8
453	6	ENSG00000123500	COL10A1
454	1	ENSG00000060718	COL11A1
455	6	ENSG00000204248	COL11A2
456	21	ENSG00000182871	COL18A1
457	17	ENSG00000108821	COL1A1
458	12	ENSG00000139219	COL2A1
459	2	ENSG00000168542	COL3A1
460	13	ENSG00000187498	COL4A1
461	13	ENSG00000134871	COL4A2
462	13	ENSG00000224821	COL4A2-AS2
463	2	ENSG00000169031	COL4A3
464	5	ENSG00000113163	COL4A3BP
465	2	ENSG00000081052	COL4A4
466	9	ENSG00000130635	COL5A1
467	19	ENSG00000080573	COL5A3
468	3	ENSG00000144810	COL8A1
469	6	ENSG00000112280	COL9A1
470	1	ENSG00000049089	COL9A2
471	20	ENSG00000092758	COL9A3
472	2	ENSG00000118004	COLEC11
473	3	ENSG00000206561	COLQ
474	19	ENSG00000105664	COMP
475	4	ENSG00000173085	COQ2
476	16	ENSG00000088682	COQ9
477	17	ENSG00000006695	COX10
478	17	ENSG00000166260	COX11
479	12	ENSG00000178449	COX14
480	10	ENSG00000014919	COX15
481	1	ENSG00000203667	COX20
482	19	ENSG00000126267	COX6B1

483	X	ENSG00000131174	COX7B
484	4	ENSG00000168993	CPLX1
485	3	ENSG00000196353	CPNE4
486	2	ENSG00000021826	CPS1
487	7	ENSG00000160917	CPSF4
488	1	ENSG00000134376	CRB1
489	3	ENSG00000113851	CRBN
490	2	ENSG00000118260	CREB1
491	16	ENSG00000005339	CREBBP
492	19	ENSG00000006016	CRLF1
493	19	ENSG00000105392	CRX
494	11	ENSG00000121671	CRY2
	21	ENSG00000160202	
495	21	ENSG00000276076	CRYAA
496	17	ENSG00000108255	CRYBA1
497	22	ENSG00000196431	CRYBA4
498	22	ENSG00000100122	CRYBB1
499	22	ENSG00000244752	CRYBB2
500	22	ENSG00000100053	CRYBB3
501	2	ENSG00000163254	CRYGC
502	2	ENSG00000118231	CRYGD
503	20	ENSG00000101266	CSNK2A1
504	8	ENSG00000104218	CSPP1
505	21	ENSG00000160213	CSTB
506	20	ENSG00000101138	CSTF1
507	4	ENSG00000159692	CTBP1
508	10	ENSG00000175029	CTBP2
509	17	ENSG00000178971	CTC1
510	16	ENSG00000102974	CTCF
511	18	ENSG00000060069	CTDP1
512	1	ENSG00000116761	CTH
513	3	ENSG00000168036	CTNNB1
514	17	ENSG00000040531	CTNS
515	16	ENSG00000168925	CTRB1
516	20	ENSG00000064601	CTSA
517	8	ENSG00000164733	CTSB
518	11	ENSG00000117984	CTSD
519	1	ENSG00000143387	CTSK
520	20	ENSG00000101160	CTSZ
521	10	ENSG00000107611	CUBN
522	2	ENSG00000036257	CUL3
523	X	ENSG00000158290	CUL4B
524	6	ENSG00000044090	CUL7
525	6	ENSG00000112659	CUL9
526	12	ENSG00000111249	CUX2
527	10	ENSG00000095485	CWF19L1
528	X	ENSG00000147113	CXorf36
529	18	ENSG00000166347	CYB5A

530	22	ENSG00000100243	CYB5R3
531	8	ENSG00000179091	CYC1
532	5	ENSG00000055163	CYFIP2
533	2	ENSG00000138061	CYP1B1
534	10	ENSG00000095596	CYP26A1
535	2	ENSG00000003137	CYP26B1
536	2	ENSG00000135929	CYP27A1
537	2	ENSG00000186684	CYP27C1
538	4	ENSG00000155016	CYP2U1
539	2	ENSG00000180902	D2HGDH
540	14	ENSG00000100592	DAAM1
541	3	ENSG00000173402	DAG1
542	3	ENSG00000178149	DALRD3
543	2	ENSG00000115866	DARS
544	1	ENSG00000117593	DARS2
545	1	ENSG00000137992	DBT
546	2	ENSG00000115827	DCAF17
547	17	ENSG00000136485	DCAF7
548	11	ENSG00000166341	DCHS1
549	4	ENSG00000197410	DCHS2
550	X	ENSG00000077279	DCX
551	1	ENSG00000153904	DDAH1
552	11	ENSG00000134574	DDB2
553	7	ENSG00000132437	DDC
554	14	ENSG00000100523	DDHD1
555	8	ENSG00000085788	DDHD2
556	1	ENSG00000244038	DDOST
557	1	ENSG00000162733	DDR2
558	12	ENSG00000013573	DDX11
559	6	ENSG00000198563	DDX39B
560	X	ENSG00000215301	DDX3X
561	5	ENSG00000145833	DDX46
562	1	ENSG00000118197	DDX59
563	4	ENSG00000137628	DDX60
564	4	ENSG00000181381	DDX60L
565	11	ENSG00000177030	DEAF1
566	11	ENSG00000184014	DENND5A
567	22	ENSG00000100150	DEPDC5
568	2	ENSG00000204311	DFNB59
569	22	ENSG00000100056	DGCR14
570	22	ENSG00000070413	DGCR2
571	22	ENSG00000183628	DGCR6
572	22	ENSG00000128191	DGCR8
573	1	ENSG00000116133	DHCR24
574	11	ENSG00000172893	DHCR7
575	19	ENSG00000104808	DHDH
576	5	ENSG00000228716	DHFR
577	16	ENSG00000102967	DHODH

578	10	ENSG00000181192	DHTKD1
579	3	ENSG00000132153	DHX30
580	10	ENSG00000089876	DHX32
581	3	ENSG00000174953	DHX36
582	17	ENSG00000108406	DHX40
583	13	ENSG00000139734	DIAPH3
584	15	ENSG00000166938	DIS3L
585	2	ENSG00000144535	DIS3L2
586	1	ENSG00000162946	DISC1
587	X	ENSG00000130826	DKC1
588	19	ENSG00000104901	DKKL1
589	11	ENSG00000150768	DLAT
590	7	ENSG00000091140	DLD
591	X	ENSG00000082458	DLG3
592	17	ENSG00000132535	DLG4
593	10	ENSG00000151208	DLG5
594	18	ENSG00000170579	DLGAP1
595	19	ENSG00000090932	DLL3
596	X	ENSG00000198947	DMD
597	4	ENSG00000152592	DMP1
598	19	ENSG00000104936	DMPK
599	9	ENSG00000064218	DMRT3
600	5	ENSG00000172869	DMXL1
601	15	ENSG00000104093	DMXL2
602	10	ENSG00000138346	DNA2
603	19	ENSG00000167646	DNAAF3
604	12	ENSG00000197653	DNAH10
605	7	ENSG00000105877	DNAH11
606	3	ENSG00000174844	DNAH12
607	17	ENSG00000187775	DNAH17
608	5	ENSG00000039139	DNAH5
609	2	ENSG00000115423	DNAH6
610	2	ENSG00000118997	DNAH7
611	6	ENSG00000124721	DNAH8
612	17	ENSG00000171595	DNAI2
613	10	ENSG00000136770	DNAJC1
614	3	ENSG00000205981	DNAJC19
615	1	ENSG00000116675	DNAJC6
616	9	ENSG00000106976	DNM1
617	19	ENSG00000130816	DNMT1
618	2	ENSG00000119772	DNMT3A
619	20	ENSG00000088305	DNMT3B
620	2	ENSG00000123992	DNPEP
621	16	ENSG00000149927	DOC2A
622	3	ENSG00000088538	DOCK3
623	19	ENSG00000130158	DOCK6
624	9	ENSG00000107099	DOCK8
625	9	ENSG00000175283	DOLK

626	19	ENSG00000104885	DOT1L
627	11	ENSG00000172269	DPAGT1
628	20	ENSG00000000419	DPM1
629	2	ENSG00000197635	DPP4
630	19	ENSG00000142002	DPP9
631	1	ENSG00000188641	DPYD
632	1	ENSG00000232542	DPYD-IT1
633	8	ENSG00000147647	DPYS
634	2	ENSG00000157851	DPYSL5
635	11	ENSG00000149295	DRD2
636	21	ENSG00000184029	DSCR4
637	6	ENSG00000096696	DSP
638	4	ENSG00000152591	DSPP
639	6	ENSG00000151914	DST
640	1	ENSG00000133059	DSTYK
641	5	ENSG00000169570	DTWD2
642	11	ENSG00000110042	DTX4
643	15	ENSG00000140274	DUOXA2
644	17	ENSG00000276023	DUSP14
645	1	ENSG00000107404	DVL1
646	18	ENSG00000141627	DYM
647	14	ENSG00000197102	DYNC1H1
648	16	ENSG00000135720	DYNC1LI2
649	11	ENSG00000187240	DYNC2H1
650	21	ENSG00000157540	DYRK1A
651	19	ENSG00000105204	DYRK1B
652	15	ENSG00000256061	DYX1C1
653	10	ENSG00000108001	EBF3
654	X	ENSG00000147155	EBP
655	1	ENSG00000117298	ECE1
656	2	ENSG00000171551	ECEL1
657	19	ENSG00000104823	ECH1
658	1	ENSG00000121310	ECHDC2
659	6	ENSG00000203734	ECT2L
660	X	ENSG00000158813	EDA
661	15	ENSG00000179151	EDC3
662	13	ENSG00000136160	EDNRB
663	11	ENSG00000074266	EED
664	20	ENSG00000101210	EEF1A2
665	20	ENSG00000215529	EFCAB8
666	X	ENSG00000090776	EFNB1
667	17	ENSG00000108883	EFTUD2
668	3	ENSG00000206120	EGFEM1P
669	2	ENSG00000115504	EHBP1
670	9	ENSG00000181090	EHMT1
671	2	ENSG00000172071	EIF2AK3
672	22	ENSG00000100129	EIF3L
673	17	ENSG00000141543	EIF4A3

674	3	ENSG00000114867	EIF4G1
675	11	ENSG00000110321	EIF4G2
676	3	ENSG00000163577	EIF5A2
677	20	ENSG00000242372	EIF6
678	17	ENSG00000006744	ELAC2
679	7	ENSG00000049540	ELN
680	6	ENSG00000118402	ELOVL4
681	7	ENSG00000164778	EN2
682	1	ENSG00000154380	ENAH
683	12	ENSG00000111674	ENO2
684	13	ENSG00000120658	ENOX1
685	6	ENSG00000197594	ENPP1
686	3	ENSG00000163378	EOGT
687	22	ENSG00000100393	EP300
688	2	ENSG00000135999	EPC2
689	18	ENSG00000152223	EPG5
690	4	ENSG00000145242	EPHA5
691	1	ENSG00000133216	EPHB2
692	8	ENSG00000261150	EPPK1
693	1	ENSG00000198758	EPS8L3
694	17	ENSG00000121053	EPX
695	2	ENSG00000178568	ERBB4
696	19	ENSG00000012061	ERCC1
697	19	ENSG00000104884	ERCC2
698	2	ENSG00000163161	ERCC3
699	16	ENSG00000175595	ERCC4
700	13	ENSG00000134899	ERCC5
701	10	ENSG00000225830	ERCC6
702	X	ENSG00000186871	ERCC6L
703	5	ENSG00000049167	ERCC8
704	19	ENSG00000105722	ERF
705	8	ENSG00000147475	ERLIN2
706	16	ENSG00000134398	ERN2
707	8	ENSG00000171320	ESCO2
708	12	ENSG00000135476	ESPL1
709	1	ENSG00000187017	ESPN
710	14	ENSG00000119715	ESRRB
711	5	ENSG00000120705	ETF1
712	15	ENSG00000140374	ETFA
713	19	ENSG00000105379	ETFB
714	4	ENSG00000171503	ETFDH
715	19	ENSG00000105755	ETHE1
716	4	ENSG00000072840	EVC
717	4	ENSG00000173040	EVC2
718	1	ENSG00000067208	EVI5
719	6	ENSG00000112685	EXOC2
720	17	ENSG00000182473	EXOC7
721	9	ENSG00000107371	EXOSC3

722	19	ENSG00000077348	EXOSC5
723	8	ENSG00000182197	EXT1
724	11	ENSG00000151348	EXT2
725	8	ENSG00000104313	EYA1
726	6	ENSG00000112319	EYA4
727	7	ENSG00000106462	EZH2
728	11	ENSG00000180210	F2
729	13	ENSG00000057593	F7
730	11	ENSG00000221968	FADS3
731	15	ENSG00000103876	FAH
732	5	ENSG00000145569	FAM105A
733	11	ENSG00000166801	FAM111A
734	7	ENSG00000122591	FAM126A
735	5	ENSG00000154153	FAM134B
736	5	ENSG00000113391	FAM172A
737	10	ENSG00000119906	FAM178A
738	1	ENSG00000116199	FAM20B
739	7	ENSG00000177706	FAM20C
740	4	ENSG00000189157	FAM47E
741	X	ENSG00000262919	FAM58A
742	20	ENSG00000042062	FAM65C
743	9	ENSG00000165716	FAM69B
744	11	ENSG00000077458	FAM76B
745	8	ENSG00000180921	FAM83H
746	4	ENSG00000251669	FAM86EP
747	8	ENSG00000182366	FAM87A
748	2	ENSG00000119812	FAM98A
749	16	ENSG00000187741	FANCA
750	X	ENSG00000181544	FANCB
751	9	ENSG00000158169	FANCC
752	3	ENSG00000144554	FANCD2
753	6	ENSG00000112039	FANCE
754	11	ENSG00000183161	FANCF
755	9	ENSG00000221829	FANCG
756	15	ENSG00000140525	FANCI
757	11	ENSG00000197601	FAR1
758	6	ENSG00000145982	FARS2
759	2	ENSG00000118246	FASTKD2
760	4	ENSG00000196159	FAT4
761	19	ENSG00000105202	FBL
762	15	ENSG00000166147	FBN1
763	5	ENSG00000138829	FBN2
764	9	ENSG00000165140	FBP1
765	16	ENSG00000099364	FBXL19
766	6	ENSG00000112234	FBXL4
767	2	ENSG00000138081	FBXO11
768	18	ENSG00000141665	FBXO15
769	8	ENSG00000214050	FBXO16

770	12	ENSG00000135108	FBXO21
771	15	ENSG00000167196	FBXO22
772	16	ENSG00000103264	FBXO31
773	X	ENSG00000102302	FGD1
774	5	ENSG00000070193	FGF10
775	3	ENSG00000114279	FGF12
776	13	ENSG00000102466	FGF14
777	5	ENSG00000156427	FGF18
778	11	ENSG00000186895	FGF3
779	4	ENSG00000138675	FGF5
780	8	ENSG00000077782	FGFR1
781	10	ENSG00000066468	FGFR2
782	4	ENSG00000068078	FGFR3
783	4	ENSG00000127418	FGFRL1
784	1	ENSG00000091483	FH
785	4	ENSG00000137460	FHDC1
786	X	ENSG00000022267	FHL1
787	6	ENSG00000112367	FIG4
788	7	ENSG00000132436	FIGNL1
789	7	ENSG00000106080	FKBP14
790	9	ENSG00000119321	FKBP15
791	19	ENSG00000181027	FKRP
792	9	ENSG00000106692	FKTN
793	1	ENSG00000143631	FLG
794	X	ENSG00000196924	FLNA
795	3	ENSG00000136068	FLNB
796	5	ENSG00000037280	FLT4
797	1	ENSG00000162769	FLVCR1
798	14	ENSG00000119686	FLVCR2
799	1	ENSG00000155816	FMN2
800	X	ENSG00000102081	FMR1
801	1	ENSG00000137942	FNBP1L
802	1	ENSG00000143107	FNDC7
803	11	ENSG00000110195	FOLR1
804	6	ENSG00000054598	FOXC1
805	16	ENSG00000176692	FOXC2
806	9	ENSG00000178919	FOXE1
807	1	ENSG00000186790	FOXE3
808	16	ENSG00000103241	FOXF1
809	14	ENSG00000176165	FOXG1
810	17	ENSG00000141568	FOXK2
811	17	ENSG00000109101	FOXN1
812	12	ENSG00000139445	FOXN4
813	3	ENSG00000114861	FOXP1
814	7	ENSG00000128573	FOXP2
815	X	ENSG00000049768	FOXP3
816	11	ENSG00000110074	FOXRED1
817	4	ENSG00000138759	FRAS1

818	9	ENSG00000164946	FREM1
819	13	ENSG00000150893	FREM2
820	4	ENSG00000109536	FRG1
821	X	ENSG00000165694	FRMD7
822	11	ENSG00000126391	FRMD8
823	9	ENSG00000070601	FRMPD1
824	13	ENSG00000073910	FRY
825	4	ENSG00000075539	FRYL
826	2	ENSG00000170820	FSHR
827	21	ENSG00000160282	FTCD
828	19	ENSG00000087086	FTL
829	16	ENSG00000140718	FTO
830	X	ENSG00000068438	FTSJ1
831	1	ENSG00000179163	FUCA1
832	3	ENSG00000163820	FYCO1
833	8	ENSG00000164930	FZD6
834	4	ENSG00000138757	G3BP2
835	17	ENSG00000171298	GAA
836	17	ENSG00000170296	GABARAP
837	9	ENSG00000136928	GABBR2
838	5	ENSG00000022355	GABRA1
839	X	ENSG00000011677	GABRA3
840	5	ENSG00000145864	GABRB2
841	15	ENSG00000166206	GABRB3
842	2	ENSG00000128683	GAD1
843	4	ENSG00000178950	GAK
844	14	ENSG00000054983	GALC
845	1	ENSG00000117308	GALE
846	17	ENSG00000108479	GALK1
847	12	ENSG00000130035	GALNT8
848	9	ENSG00000213930	GALT
849	19	ENSG00000130005	GAMT
850	16	ENSG00000261609	GAN
851	9	ENSG00000165219	GAPVD1
852	22	ENSG00000185340	GAS2L1
853	17	ENSG00000270765	GAS2L2
854	X	ENSG00000102145	GATA1
855	3	ENSG00000179348	GATA2
856	8	ENSG00000136574	GATA4
857	18	ENSG00000141448	GATA6
858	15	ENSG00000171766	GATM
859	9	ENSG00000070610	GBA2
860	3	ENSG00000114480	GBE1
861	19	ENSG00000105607	GCDH
862	14	ENSG00000131979	GCH1
863	7	ENSG00000106633	GCK
864	2	ENSG00000084734	GCKR
865	6	ENSG00000137270	GCM1

866	16	ENSG00000140905	GCSH
867	20	ENSG00000125965	GDF5
868	8	ENSG00000156466	GDF6
869	X	ENSG00000203879	GDI1
870	17	ENSG00000131095	GFAP
871	3	ENSG00000168827	GFM1
872	6	ENSG00000145990	GFOD1
873	16	ENSG00000141098	GFOD2
874	16	ENSG00000103365	GGA2
875	17	ENSG00000278311	GGNBP2
876	5	ENSG00000112964	GHR
877	16	ENSG00000131153	GINS2
878	1	ENSG00000137960	GIPC2
879	6	ENSG00000152661	GJA1
880	6	ENSG00000135355	GJA10
881	13	ENSG00000121743	GJA3
882	1	ENSG00000121634	GJA8
883	13	ENSG00000165474	GJB2
884	13	ENSG00000121742	GJB6
885	1	ENSG00000198835	GJC2
886	X	ENSG00000198814	GK
887	3	ENSG00000170266	GLB1
888	9	ENSG00000178445	GLDC
889	9	ENSG00000119392	GLE1
890	2	ENSG00000074047	GLI2
891	7	ENSG00000106571	GLI3
892	1	ENSG00000174332	GLIS1
893	9	ENSG00000107249	GLIS3
894	1	ENSG00000174842	GLMN
895	6	ENSG00000112164	GLP1R
896	17	ENSG00000065325	GLP2R
897	5	ENSG00000145888	GLRA1
898	X	ENSG00000101958	GLRA2
899	10	ENSG00000148672	GLUD1
900	1	ENSG00000135821	GLUL
901	3	ENSG00000168237	GLYCTK
902	5	ENSG00000196743	GM2A
903	2	ENSG00000144591	GMPPA
904	3	ENSG00000173540	GMPPB
905	7	ENSG00000127955	GNAI1
906	1	ENSG00000065135	GNAI3
907	16	ENSG00000087258	GNAO1
908	9	ENSG00000156052	GNAQ
909	20	ENSG00000087460	GNAS
910	22	ENSG00000128266	GNAZ
911	9	ENSG00000159921	GNE
912	1	ENSG00000116906	GNPAT
913	12	ENSG00000111670	GNPTAB

914	16	ENSG00000090581	GNPTG
915	4	ENSG00000109163	GNRHR
916	12	ENSG00000135677	GNS
917	14	ENSG00000066455	GOLGA5
918	3	ENSG00000173230	GOLGB1
919	1	ENSG00000120370	GORAB
920	17	ENSG00000108433	GOSR2
921	22	ENSG00000203618	GP1BB
922	10	ENSG00000119927	GPAM
923	1	ENSG00000092978	GPATCH2
924	2	ENSG00000063660	GPC1
925	X	ENSG00000147257	GPC3
926	14	ENSG00000171723	GPHN
927	19	ENSG00000105220	GPI
928	19	ENSG00000125734	GPR108
929	4	ENSG00000152990	GPR125
930	3	ENSG00000154165	GPR15
931	17	ENSG00000277399	GPR179
932	16	ENSG00000205336	GPR56
933	5	ENSG00000164199	GPR98
934	1	ENSG00000121957	GPSM2
935	19	ENSG00000089351	GRAMD1A
936	1	ENSG00000158055	GRHL3
937	X	ENSG00000125675	GRIA3
938	10	ENSG00000182771	GRID1
939	6	ENSG00000164418	GRIK2
940	16	ENSG00000183454	GRIN2A
941	12	ENSG00000273079	GRIN2B
942	12	ENSG00000155974	GRIP1
943	6	ENSG00000152822	GRM1
944	5	ENSG00000113262	GRM6
945	17	ENSG00000030582	GRN
946	4	ENSG00000215203	GRXCR1
947	X	ENSG00000189369	GSPT2
948	20	ENSG00000100983	GSS
949	6	ENSG00000213780	GTF2H4
950	6	ENSG00000272047	GTF2H5
951	12	ENSG00000070019	GUCY2C
952	17	ENSG00000132518	GUCY2D
953	7	ENSG00000169919	GUSB
954	14	ENSG00000100450	GZMH
955	11	ENSG00000130600	H19
956	5	ENSG00000113648	H2AFY
957	1	ENSG00000163041	H3F3A
958	1	ENSG00000049239	H6PD
959	4	ENSG00000138796	HADH
960	12	ENSG00000084110	HAL
961	19	ENSG00000187664	HAPLN4

962	1	ENSG00000143575	HAX1
963	X	ENSG00000004961	HCCS
964	X	ENSG00000172534	HCFC1
965	5	ENSG00000164588	HCN1
966	2	ENSG00000068024	HDAC4
967	X	ENSG00000094631	HDAC6
968	X	ENSG00000147099	HDAC8
969	1	ENSG00000119285	HEATR1
970	14	ENSG00000092148	HECTD1
971	2	ENSG00000138411	HECW2
972	3	ENSG00000173706	HEG1
973	10	ENSG00000119969	HELLS
974	11	ENSG00000165478	HEPACAM
975	X	ENSG00000089472	HEPH
976	15	ENSG00000128731	HERC2
977	3	ENSG00000163666	HESX1
978	15	ENSG00000213614	HEXA
979	5	ENSG00000049860	HEXB
980	17	ENSG00000169660	HEXDC
981	7	ENSG00000019991	HGF
982	8	ENSG00000165102	HGSNAT
983	2	ENSG00000198130	HIBCH
984	17	ENSG00000131097	HIGD1B
985	5	ENSG00000169567	HINT1
986	12	ENSG00000130787	HIP1R
987	6	ENSG00000276903	HIST1H2AL
988	6	ENSG00000158373	HIST1H2BD
989	6	ENSG00000275714	HIST1H3A
990	6	ENSG00000197061	HIST1H4C
991	6	ENSG00000010818	HIVEP2
992	21	ENSG00000159267	HLCS
993	19	ENSG00000064961	HMG20B
994	12	ENSG00000149948	HMGA2
995	X	ENSG00000029993	HMGB3
996	1	ENSG00000117305	HMGCL
997	1	ENSG00000134240	HMGCS2
998	17	ENSG00000275410	HNF1B
999	1	ENSG00000153187	HNRNPU
1000	5	ENSG00000152413	HOMER1
1001	8	ENSG00000168172	HOOK3
1002	7	ENSG00000105991	HOXA1
1003	12	ENSG00000180818	HOXC10
1004	12	ENSG00000123364	HOXC13
1005	2	ENSG00000128714	HOXD13
1006	12	ENSG00000158104	HPD
1007	X	ENSG00000165704	HPRT1
1008	10	ENSG00000107521	HPS1
1009	10	ENSG00000172987	HPSE2

1010	8	ENSG00000168453	HR
1011	11	ENSG00000174775	HRAS
1012	18	ENSG00000134489	HRH4
1013	1	ENSG00000153936	HS2ST1
1014	X	ENSG00000072506	HSD17B10
1015	5	ENSG00000133835	HSD17B4
1016	21	ENSG00000160207	HSF2BP
1017	16	ENSG00000102878	HSF4
1018	5	ENSG00000113013	HSPA9
1019	5	ENSG00000169271	HSPB3
1020	2	ENSG00000144381	HSPD1
1021	1	ENSG00000142798	HSPG2
1022	X	ENSG00000086758	HUWE1
1023	3	ENSG00000114378	HYAL1
1024	16	ENSG00000157423	HYDIN
1025	11	ENSG00000198331	HYLS1
	X	ENSG00000010404	
1026	X	ENSG00000241489	IDS
1027	4	ENSG00000127415	IDUA
	18	ENSG00000134049	
1028	18	ENSG00000267228	IER3IP1
1029	2	ENSG00000115267	IFIH1
1030	11	ENSG00000206013	IFITM5
1031	12	ENSG00000111537	IFNG
1032	3	ENSG00000163913	IFT122
1033	16	ENSG00000187535	IFT140
1034	2	ENSG00000138002	IFT172
1035	22	ENSG00000100360	IFT27
1036	14	ENSG00000119650	IFT43
1037	3	ENSG00000068885	IFT80
1038	X	ENSG00000089289	IGBP1
1039	15	ENSG00000103742	IGDCC4
1040	12	ENSG00000017427	IGF1
1041	11	ENSG00000167244	IGF2
1042	6	ENSG00000197081	IGF2R
1043	11	ENSG00000132740	IGHMBP2
1044	22	ENSG00000211658	IGLV3-27
1045	X	ENSG00000147255	IGSF1
1046	2	ENSG00000163501	IHH
1047	X	ENSG00000269335	IKBKG
	1	ENSG00000081985	
1048	1	ENSG00000281152	IL12RB2
1049	3	ENSG00000163702	IL17RC
1050	2	ENSG00000115604	IL18R1
1051	X	ENSG00000169306	IL1RAPL1
1052	3	ENSG00000091181	IL5RA
1053	3	ENSG00000145103	ILDR1
1054	11	ENSG00000166333	ILK

1055	19	ENSG00000105135	ILVBL
1056	8	ENSG00000104331	IMPAD1
1057	3	ENSG00000178035	IMPDH2
1058	15	ENSG00000128908	INO80
1059	4	ENSG00000109452	INPP4B
1060	9	ENSG00000148384	INPP5E
1061	22	ENSG00000185133	INPP5J
1062	11	ENSG00000165458	INPPL1
1063	11	ENSG00000254647	INS
1064	19	ENSG00000171105	INSR
1065	1	ENSG00000027644	INSRR
1066	8	ENSG00000104613	INTS10
1067	1	ENSG00000198700	IPO9
1068	1	ENSG00000197429	IPP
1069	7	ENSG00000106012	IQCE
1070	3	ENSG00000144711	IQSEC1
1071	X	ENSG00000124313	IQSEC2
1072	1	ENSG00000117595	IRF6
1073	19	ENSG00000124449	IRGC
1074	2	ENSG00000169047	IRS1
1075	7	ENSG00000214960	ISPD
1076	17	ENSG00000005884	ITGA3
1077	2	ENSG00000091409	ITGA6
1078	12	ENSG00000135424	ITGA7
1079	3	ENSG00000144668	ITGA9
1080	17	ENSG00000259207	ITGB3
1081	2	ENSG00000115221	ITGB6
1082	3	ENSG00000150995	ITPR1
1083	15	ENSG00000128928	IVD
1084	6	ENSG00000009765	IYD
1085	9	ENSG00000205442	IZUMO3
1086	20	ENSG00000101384	JAG1
1087	19	ENSG00000105639	JAK3
1088	11	ENSG00000166086	JAM3
1089	22	ENSG00000100221	JOSD1
1090	16	ENSG00000154118	JPH3
1091	9	ENSG00000107104	KANK1
1092	17	ENSG00000120071	KANSL1
1093	8	ENSG00000083168	KAT6A
1094	10	ENSG00000156650	KAT6B
1095	16	ENSG00000140854	KATNB1
1096	3	ENSG00000187715	KBTBD12
1097	15	ENSG00000234438	KBTBD13
1098	12	ENSG00000111262	KCNA1
1099	1	ENSG00000177301	KCNA2
1100	20	ENSG00000158445	KCNB1
1101	19	ENSG00000131398	KCNC3
1102	1	ENSG00000116396	KCNC4

1103	1	ENSG00000171385	KCND3
1104	21	ENSG00000159197	KCNE2
1105	2	ENSG00000171126	KCNG3
1106	1	ENSG00000143473	KCNH1
1107	17	ENSG00000089558	KCNH4
1108	11	ENSG00000151704	KCNJ1
1109	1	ENSG00000177807	KCNJ10
1110	11	ENSG00000187486	KCNJ11
1111	22	ENSG00000168135	KCNJ4
1112	21	ENSG00000157542	KCNJ6
1113	2	ENSG00000171303	KCNK3
1114	10	ENSG00000156113	KCNMA1
1115	20	ENSG00000075043	KCNQ2
1116	8	ENSG00000184156	KCNQ3
1117	9	ENSG00000107147	KCNT1
1118	18	ENSG00000134504	KCTD1
1119	7	ENSG00000243335	KCTD7
1120	19	ENSG00000105438	KDELR1
1121	22	ENSG00000100196	KDELR3
1122	5	ENSG00000120733	KDM3B
1123	19	ENSG00000127663	KDM4B
1124	X	ENSG00000126012	KDM5C
1125	X	ENSG00000147050	KDM6A
1126	2	ENSG00000138030	KHK
1127	7	ENSG00000122548	KIAA0087
1128	3	ENSG00000145016	KIAA0226
1129	9	ENSG00000136813	KIAA0368
1130	4	ENSG00000121210	KIAA0922
1131	12	ENSG00000136051	KIAA1033
1132	4	ENSG00000138688	KIAA1109
1133	4	ENSG00000109265	KIAA1211
1134	6	ENSG00000112379	KIAA1244
1135	10	ENSG00000198954	KIAA1279
1136	7	ENSG00000122778	KIAA1549
1137	X	ENSG00000050030	KIAA2022
1138	9	ENSG00000183354	KIAA2026
1139	2	ENSG00000134313	KIDINS220
1140	10	ENSG00000138160	KIF11
1141	6	ENSG00000137177	KIF13A
1142	17	ENSG00000186185	KIF18B
1143	2	ENSG00000130294	KIF1A
1144	16	ENSG00000079616	KIF22
1145	15	ENSG00000137807	KIF23
1146	X	ENSG00000090889	KIF4A
1147	10	ENSG00000170759	KIF5B
1148	2	ENSG00000168280	KIF5C
1149	15	ENSG00000166813	KIF7
1150	1	ENSG00000183853	KIRREL

1151	11	ENSG00000149571	KIRREL3
1152	4	ENSG00000157404	KIT
1153	12	ENSG00000049130	KITLG
1154	4	ENSG00000134962	KLB
1155	19	ENSG00000105610	KLF1
1156	13	ENSG00000118922	KLF12
1157	16	ENSG00000104731	KLHDC4
1158	17	ENSG00000161594	KLHL10
1159	X	ENSG00000174010	KLHL15
1160	3	ENSG00000114648	KLHL18
1161	6	ENSG00000186231	KLHL32
1162	16	ENSG00000135686	KLHL36
1163	3	ENSG00000157119	KLHL40
1164	19	ENSG00000142515	KLK3
1165	12	ENSG00000134539	KLRD1
1166	11	ENSG00000118058	KMT2A
1167	19	ENSG00000272333	KMT2B
1168	12	ENSG00000167548	KMT2D
1169	19	ENSG00000118162	KPTN
1170	12	ENSG00000133703	KRAS
1171	7	ENSG00000133619	KRBA1
1172	17	ENSG00000171346	KRT15
1173	12	ENSG00000189182	KRT77
1174	12	ENSG00000170421	KRT8
1175	12	ENSG00000135443	KRT85
1176	21	ENSG00000186965	KRTAP19-2
1177	2	ENSG00000115919	KYNU
1178	X	ENSG00000198910	L1CAM
1179	14	ENSG00000087299	L2HGDH
1180	18	ENSG00000101680	LAMA1
1181	6	ENSG00000196569	LAMA2
1182	6	ENSG00000112769	LAMA4
1183	1	ENSG00000135862	LAMC1
1184	9	ENSG00000050555	LAMC3
1185	X	ENSG00000005893	LAMP2
1186	22	ENSG00000133424	LARGE
1187	15	ENSG00000166173	LARP6
1188	4	ENSG00000174720	LARP7
1189	5	ENSG00000133706	LARS
1190	1	ENSG00000143815	LBR
1191	15	ENSG00000168806	LCMT2
1192	2	ENSG00000115850	LCT
1193	11	ENSG00000134333	LDHA
1194	4	ENSG00000138795	LEF1
1195	6	ENSG00000161904	LEMD2
1196	12	ENSG00000174106	LEMD3
1197	19	ENSG00000275183	LENG9
1198	4	ENSG00000168924	LETM1

1199	7	ENSG00000106003	LFNG
1200	9	ENSG00000107187	LHX3
1201	1	ENSG00000121454	LHX4
1202	4	ENSG00000121897	LIAS
1203	17	ENSG00000005156	LIG3
1204	13	ENSG00000174405	LIG4
1205	4	ENSG00000064042	LIMCH1
1206	15	ENSG00000169783	LINGO1
1207	6	ENSG00000168216	LMBRD1
1208	1	ENSG00000160789	LMNA
1209	5	ENSG00000113368	LMNB1
1210	9	ENSG00000136944	LMX1B
1211	19	ENSG00000196365	LONP1
1212	10	ENSG00000138131	LOXL4
1213	9	ENSG00000198121	LPAR1
1214	2	ENSG00000134324	LPIN1
1215	3	ENSG00000145012	LPP
1216	4	ENSG00000198589	LRBA
1217	3	ENSG00000186001	LRCH3
1218	12	ENSG00000139263	LRIG3
1219	12	ENSG00000123384	LRP1
1220	2	ENSG00000081479	LRP2
1221	11	ENSG00000134569	LRP4
1222	11	ENSG00000162337	LRP5
1223	2	ENSG00000138095	LRPPRC
1224	6	ENSG00000079691	LRRC16A
1225	3	ENSG00000114248	LRRC31
1226	7	ENSG00000128594	LRRC4
1227	8	ENSG00000129295	LRRC6
1228	3	ENSG00000093167	LRRFIP2
1229	12	ENSG00000133640	LRRIQ1
1230	15	ENSG00000154237	LRRK1
1231	11	ENSG00000184154	LRTOMT
1232	6	ENSG00000226979	LTA
1233	14	ENSG00000119681	LTBP2
1234	17	ENSG00000108848	LUC7L3
1235	2	ENSG00000248672	LY75-CD302
1236	1	ENSG00000122224	LY9
1237	8	ENSG00000197353	LYPD2
1238	1	ENSG00000143669	LYST
1239	3	ENSG00000163818	LZTFL1
1240	22	ENSG00000099949	LZTR1
1241	11	ENSG00000110514	MADD
1242	4	ENSG00000090316	MAEA
1243	16	ENSG00000178573	MAF
1244	20	ENSG00000204103	MAFB
1245	15	ENSG00000254585	MAGEL2
1246	9	ENSG00000177239	MAN1B1

1247	19	ENSG00000104774	MAN2B1
1248	4	ENSG00000109323	MANBA
1249	X	ENSG00000189221	MAOA
1250	15	ENSG00000169032	MAP2K1
1251	19	ENSG00000126934	MAP2K2
1252	5	ENSG00000095015	MAP3K1
1253	6	ENSG00000135341	MAP3K7
1254	14	ENSG00000006432	MAP3K9
1255	22	ENSG00000185386	MAPK11
1256	15	ENSG00000137802	MAPKBP1
1257	17	ENSG00000186868	MAPT
1258	1	ENSG00000116141	MARK1
1259	2	ENSG00000247626	MARS2
1260	3	ENSG00000127241	MASP1
1261	1	ENSG00000086015	MAST2
1262	10	ENSG00000151224	MAT1A
1263	1	ENSG00000162510	MATN1
1264	2	ENSG00000132031	MATN3
1265	3	ENSG00000180611	MB21D2
1266	2	ENSG00000204406	MBD5
1267	X	ENSG00000012174	MBTPS2
1268	18	ENSG00000185231	MC2R
1269	3	ENSG00000078070	MCCC1
1270	5	ENSG00000131844	MCCC2
1271	2	ENSG00000124370	MCEE
1272	13	ENSG00000126217	MCF2L
1273	1	ENSG00000143384	MCL1
1274	21	ENSG00000215424	MCM3AP-AS1
1275	19	ENSG00000090674	MCOLN1
1276	8	ENSG00000147316	MCPH1
1277	X	ENSG00000169057	MECP2
1278	X	ENSG00000184634	MED12
1279	17	ENSG00000108510	MED13
1280	12	ENSG00000123066	MED13L
1281	22	ENSG00000099917	MED15
1282	4	ENSG00000118579	MED28
1283	5	ENSG00000081189	MEF2C
1284	1	ENSG00000116604	MEF2D
1285	5	ENSG00000145794	MEGF10
1286	19	ENSG00000105429	MEGF8
1287	15	ENSG00000134138	MEIS2
1288	15	ENSG00000188095	MESP2
1289	7	ENSG00000105976	MET
1290	17	ENSG00000181038	METTL23
	11	ENSG00000235718	
1291	11	ENSG00000259159	MFRP
1292	4	ENSG00000164073	MFSD8
1293	7	ENSG00000257335	MGAM

1294	14	ENSG00000168282	MGAT2
1295	2	ENSG00000152127	MGAT5
1296	20	ENSG00000125871	MGME1
1297	12	ENSG00000111341	MGP
1298	14	ENSG00000150526	MIA2
1299	X	ENSG00000101871	MID1
1300	X	ENSG00000080561	MID2
1301	13	ENSG00000215417	MIR17HG
1302	10	ENSG00000266676	MIR4297
1303	3	ENSG00000187098	MITF
1304	20	ENSG00000125863	MKKS
1305	15	ENSG00000179455	MKRN3
1306	15	ENSG00000260978	MKRN3-AS1
1307	17	ENSG00000011143	MKS1
1308	22	ENSG00000100427	MLC1
1309	3	ENSG00000076242	MLH1
1310	6	ENSG00000130396	MLLT4
1311	7	ENSG00000009950	MLXIPL
1312	16	ENSG00000103150	MLYCD
1313	4	ENSG00000151611	MMAA
1314	12	ENSG00000139428	MMAB
1315	1	ENSG00000132763	MMACHC
1316	2	ENSG00000168288	MMADHC
1317	11	ENSG00000137745	MMP13
1318	12	ENSG00000123342	MMP19
1319	20	ENSG00000100985	MMP9
1320	22	ENSG00000169184	MN1
1321	7	ENSG00000130675	MNX1
1322	6	ENSG00000124615	MOCS1
1323	5	ENSG00000164172	MOCS2
1324	17	ENSG00000129255	MPDU1
1325	9	ENSG00000107186	MPDZ
1326	15	ENSG00000178802	MPI
1327	7	ENSG00000168303	MPLKIP
1328	7	ENSG00000105926	MPP6
1329	2	ENSG00000115204	MPV17
1330	1	ENSG00000153029	MR1
1331	22	ENSG00000185608	MRPL40
1332	2	ENSG00000135900	MRPL44
1333	5	ENSG00000113048	MRPS27
1334	11	ENSG00000156738	MS4A1
1335	2	ENSG00000116062	MSH6
1336	3	ENSG00000174579	MSL2
1337	X	ENSG00000005302	MSL3
1338	16	ENSG00000162006	MSLNL
1339	4	ENSG00000163132	MSX1
1340	5	ENSG00000120149	MSX2
1341	MT	ENSG00000198899	MT-ATP6

1342	14	ENSG00000182979	MTA1
1343	9	ENSG00000099810	MTAP
1344	15	ENSG00000103707	MTFMT
1345	1	ENSG00000177000	MTHFR
1346	X	ENSG00000171100	MTM1
1347	11	ENSG00000087053	MTMR2
1348	22	ENSG00000100330	MTMR3
1349	17	ENSG00000108389	MTMR4
1350	6	ENSG00000135297	MTO1
1351	1	ENSG00000198793	MTOR
1352	1	ENSG00000116984	MTR
1353	5	ENSG00000124275	MTRR
1354	4	ENSG00000138823	MTTP
1355	7	ENSG00000205277	MUC12
1356	19	ENSG00000181143	MUC16
1357	3	ENSG00000145113	MUC4
1358	11	ENSG00000215182	MUC5AC
1359	11	ENSG00000117983	MUC5B
1360	6	ENSG00000146085	MUT
1361	12	ENSG00000110921	MVK
1362	13	ENSG00000005810	MYCBP2
1363	2	ENSG00000134323	MYCN
1364	17	ENSG00000006788	MYH13
1365	17	ENSG00000109063	MYH3
1366	22	ENSG00000100345	MYH9
1367	17	ENSG00000091536	MYO15A
1368	17	ENSG00000266714	MYO15B
1369	12	ENSG00000166866	MYO1A
1370	15	ENSG00000157483	MYO1E
1371	19	ENSG00000142347	MYO1F
1372	7	ENSG00000136286	MYO1G
1373	10	ENSG00000095777	MYO3A
1374	15	ENSG00000197535	MYO5A
1375	18	ENSG00000167306	MYO5B
1376	10	ENSG00000138119	MYOF
1377	1	ENSG00000142661	MYOM3
1378	1	ENSG00000162601	MYSM1
1379	2	ENSG00000186487	MYT1L
1380	16	ENSG00000102921	N4BP1
1381	X	ENSG00000102030	NAA10
1382	4	ENSG00000164134	NAA15
1383	22	ENSG00000198951	NAGA
1384	17	ENSG00000108784	NAGLU
1385	17	ENSG00000161653	NAGS
1386	13	ENSG00000102452	NALCN
1387	7	ENSG00000105835	NAMPT
1388	11	ENSG00000137513	NARS2
1389	3	ENSG00000160796	NBEAL2

1390	8	ENSG00000104320	NBN
1391	11	ENSG00000151503	NCAPD3
1392	4	ENSG00000109805	NCAPG
1393	12	ENSG00000123338	NCKAP1L
1394	12	ENSG00000167566	NCKAP5L
1395	2	ENSG00000115053	NCL
1396	17	ENSG00000141027	NCOR1
1397	16	ENSG00000072864	NDE1
1398	15	ENSG00000182636	NDN
1399	X	ENSG00000124479	NDP
1400	5	ENSG00000070614	NDST1
1401	X	ENSG00000125356	NDUFA1
1402	2	ENSG00000130414	NDUFA10
1403	19	ENSG00000174886	NDUFA11
1404	12	ENSG00000184752	NDUFA12
1405	5	ENSG00000131495	NDUFA2
1406	12	ENSG00000139180	NDUFA9
1407	15	ENSG00000137806	NDUFAF1
1408	5	ENSG00000164182	NDUFAF2
1409	3	ENSG00000178057	NDUFAF3
1410	6	ENSG00000123545	NDUFAF4
1411	20	ENSG00000101247	NDUFAF5
1412	8	ENSG00000156170	NDUFAF6
1413	2	ENSG00000119013	NDUFB3
1414	3	ENSG00000065518	NDUFB4
1415	8	ENSG00000147684	NDUFB9
1416	2	ENSG00000023228	NDUFS1
1417	1	ENSG00000158864	NDUFS2
1418	11	ENSG00000213619	NDUFS3
1419	5	ENSG00000164258	NDUFS4
1420	5	ENSG00000145494	NDUFS6
1421	19	ENSG00000115286	NDUFS7
1422	11	ENSG00000110717	NDUFS8
1423	11	ENSG00000167792	NDUFV1
1424	18	ENSG00000178127	NDUFV2
1425	2	ENSG00000183091	NEB
1426	10	ENSG00000078114	NEBL
1427	18	ENSG00000049759	NEDD4L
1428	4	ENSG00000137601	NEK1
1429	3	ENSG00000114670	NEK11
1430	6	ENSG00000204386	NEU1
1431	1	ENSG00000162614	NEXN
1432	17	ENSG00000196712	NF1
1433	16	ENSG00000102908	NFAT5
1434	1	ENSG00000162599	NFIA
1435	19	ENSG00000008441	NFIX
1436	10	ENSG00000077150	NFKB2
1437	2	ENSG00000169599	NFU1

1438	1	ENSG00000134259	NGF
1439	3	ENSG00000151092	NGLY1
1440	5	ENSG00000145912	NHP2
1441	X	ENSG00000188158	NHS
1442	6	ENSG00000135540	NHSL1
1443	14	ENSG00000087303	NID2
1444	14	ENSG00000100503	NIN
1445	5	ENSG00000164190	NIPBL
1446	22	ENSG00000184117	NIPSNAP1
1447	3	ENSG00000010322	NISCH
1448	3	ENSG00000114857	NKTR
1449	14	ENSG00000136352	NKX2-1
1450	20	ENSG00000125820	NKX2-2
1451	5	ENSG00000183072	NKX2-5
1452	4	ENSG00000109705	NKX3-2
1453	3	ENSG00000169760	NLGN1
1454	X	ENSG00000196338	NLGN3
1455	X	ENSG00000146938	NLGN4X
1456	11	ENSG00000182261	NLRP10
1457	1	ENSG00000162711	NLRP3
1458	19	ENSG00000171487	NLRP5
1459	1	ENSG00000173614	NMNAT1
1460	5	ENSG00000112992	NNT
1461	7	ENSG00000106410	NOBOX
1462	10	ENSG00000173145	NOC3L
1463	10	ENSG00000156574	NODAL
1464	17	ENSG00000183691	NOG
1465	X	ENSG00000147140	NONO
1466	15	ENSG00000182117	NOP10
1467	12	ENSG00000111641	NOP2
1468	14	ENSG00000196943	NOP9
1469	12	ENSG00000089250	NOS1
1470	1	ENSG00000134250	NOTCH2
1471	19	ENSG00000074181	NOTCH3
1472	15	ENSG00000185823	NPAP1
1473	19	ENSG00000130751	NPAS1
1474	14	ENSG00000151322	NPAS3
1475	18	ENSG00000141458	NPC1
1476	14	ENSG00000119655	NPC2
1477	2	ENSG00000144061	NPHP1
1478	3	ENSG00000113971	NPHP3
1479	1	ENSG00000131697	NPHP4
1480	19	ENSG00000161270	NPHS1
1481	1	ENSG00000116218	NPHS2
1482	17	ENSG00000182446	NPLOC4
1483	9	ENSG00000159899	NPR2
1484	X	ENSG00000169297	NR0B1
1485	5	ENSG00000175745	NR2F1

1486	2	ENSG00000153234	NR4A2
1487	9	ENSG00000148200	NR6A1
1488	1	ENSG00000213281	NRAS
1489	21	ENSG00000180530	NRIP1
1490	10	ENSG00000099250	NRP1
1491	2	ENSG00000179915	NRXN1
1492	11	ENSG00000110076	NRXN2
1493	14	ENSG00000021645	NRXN3
1494	5	ENSG00000165671	NSD1
1495	X	ENSG00000147383	NSDHL
1496	5	ENSG00000037474	NSUN2
1497	10	ENSG00000076685	NT5C2
1498	7	ENSG00000122643	NT5C3A
1499	1	ENSG00000198400	NTRK1
1500	14	ENSG00000151413	NUBPL
1501	12	ENSG00000111581	NUP107
1502	19	ENSG00000213024	NUP62
1503	14	ENSG00000205978	NYNRIN
1504	X	ENSG00000188937	NYX
1505	15	ENSG00000180304	OAZ2
1506	1	ENSG00000154358	OBSCN
1507	2	ENSG00000124006	OBSL1
1508	15	ENSG00000104044	OCA2
1509	5	ENSG00000197822	OCLN
1510	X	ENSG00000122126	OCRL
1511	2	ENSG00000115758	ODC1
1512	X	ENSG00000046651	OFD1
1513	X	ENSG00000147162	OGT
1514	10	ENSG00000197430	OPALIN
1515	11	ENSG00000183715	OPCML
1516	X	ENSG00000079482	OPHN1
1517	11	ENSG00000254737	OR10G4
1518	19	ENSG00000186723	OR10H1
1519	1	ENSG00000173285	OR10K1
1520	11	ENSG00000180475	OR10Q1
1521	16	ENSG00000168158	OR2C1
1522	1	ENSG00000198128	OR2L3
1523	1	ENSG00000175143	OR2T1
	11	ENSG00000172208	
1524	11	ENSG00000279556	OR4X2
1525	11	ENSG00000172769	OR5B3
1526	11	ENSG00000149133	OR5F1
1527	11	ENSG00000255012	OR5M1
1528	1	ENSG00000197532	OR6Y1
1529	19	ENSG00000170923	OR7G2
1530	1	ENSG00000085840	ORC1
1531	6	ENSG00000135336	ORC3
1532	2	ENSG00000115947	ORC4

1533	16	ENSG00000091651	ORC6
1534	20	ENSG00000130703	OSBPL2
1535	6	ENSG00000081087	OSTM1
1536	X	ENSG00000036473	OTC
1537	2	ENSG00000115155	OTOF
1538	12	ENSG00000165899	OTOGL
1539	14	ENSG00000165588	OTX2
1540	12	ENSG00000187950	OVCH1
1541	5	ENSG00000083720	OXCT1
1542	11	ENSG00000149380	P4HA3
1543	8	ENSG00000070756	PABPC1
1544	11	ENSG00000175115	PACS1
1545	14	ENSG00000179364	PACS2
1546	17	ENSG00000007168	PAFAH1B1
1547	12	ENSG00000171759	PAH
1548	X	ENSG00000077264	PAK3
1549	16	ENSG00000083093	PALB2
1550	4	ENSG00000129116	PALLD
1551	16	ENSG00000217930	PAM16
1552	20	ENSG00000125779	PANK2
1553	10	ENSG00000148832	PAOX
1554	10	ENSG00000198682	PAPSS2
1555	6	ENSG00000185345	PARK2
1556	16	ENSG00000140694	PARN
1557	1	ENSG00000143799	PARP1
1558	3	ENSG00000173200	PARP15
1559	5	ENSG00000151883	PARP8
1560	3	ENSG00000138496	PARP9
1561	12	ENSG00000177425	PAWR
1562	20	ENSG00000125813	PAX1
1563	10	ENSG00000075891	PAX2
1564	2	ENSG00000135903	PAX3
1565	11	ENSG00000007372	PAX6
1566	2	ENSG00000125618	PAX8
1567	14	ENSG00000198807	PAX9
1568	11	ENSG00000173599	PC
1569	10	ENSG00000166228	PCBD1
1570	13	ENSG00000175198	PCCA
1571	3	ENSG00000114054	PCCB
1572	4	ENSG00000138650	PCDH10
1573	X	ENSG00000165194	PCDH19
1574	5	ENSG00000204965	PCDHA5
1575	5	ENSG00000171815	PCDHB1
1576	5	ENSG00000120328	PCDHB12
1577	5	ENSG00000253159	PCDHGA12
1578	5	ENSG00000262576	PCDHGA4
1579	5	ENSG00000262209	PCDHGB3
1580	11	ENSG00000165494	PCF11

1581	17	ENSG00000277258	PCGF2
1582	7	ENSG00000186472	PCLO
1583	21	ENSG00000160299	PCNT
1584	2	ENSG00000128655	PDE11A
1585	5	ENSG00000113448	PDE4D
1586	1	ENSG00000178104	PDE4DIP
1587	17	ENSG00000185527	PDE6G
1588	22	ENSG00000100311	PDGFB
1589	5	ENSG00000113721	PDGFRB
1590	X	ENSG00000131828	PDHA1
1591	11	ENSG00000110435	PDHX
1592	16	ENSG00000185615	PDIA2
1593	15	ENSG00000167004	PDIA3
1594	3	ENSG00000065485	PDIA5
1595	8	ENSG00000164951	PDP1
1596	13	ENSG00000083642	PDS5B
1597	10	ENSG00000148459	PDSS1
1598	6	ENSG00000164494	PDSS2
1599	13	ENSG00000139515	PDX1
1600	16	ENSG00000179889	PDXDC1
1601	8	ENSG00000134020	PEBP4
1602	2	ENSG00000115425	PECR
1603	19	ENSG00000198300	PEG3
1604	19	ENSG00000124299	PEPD
1605	19	ENSG00000229833	PET100
1606	7	ENSG00000127980	PEX1
1607	1	ENSG00000157911	PEX10
1608	17	ENSG00000108733	PEX12
1609	2	ENSG00000162928	PEX13
1610	1	ENSG00000142655	PEX14
1611	11	ENSG00000121680	PEX16
1612	1	ENSG00000162735	PEX19
1613	8	ENSG00000164751	PEX2
1614	22	ENSG00000215193	PEX26
1615	6	ENSG00000034693	PEX3
1616	12	ENSG00000139197	PEX5
1617	6	ENSG00000124587	PEX6
1618	6	ENSG00000112357	PEX7
1619	10	ENSG00000067057	PFKP
1620	11	ENSG00000148985	PGAP2
1621	X	ENSG00000102144	PGK1
1622	1	ENSG00000079739	PGM1
1623	6	ENSG00000013375	PGM3
1624	11	ENSG00000082175	PGR
1625	6	ENSG00000112137	PHACTR1
1626	17	ENSG00000109118	PHF12
1627	8	ENSG00000129292	PHF20L1
1628	11	ENSG00000135365	PHF21A

1629	X	ENSG00000156531	PHF6
1630	X	ENSG00000172943	PHF8
1631	1	ENSG00000092621	PHGDH
1632	4	ENSG00000109132	PHOX2B
1633	7	ENSG00000006576	PHTF2
	4	ENSG00000038210	
1634	4	ENSG00000281028	PI4K2B
1635	18	ENSG00000154864	PIEZO2
1636	17	ENSG00000108474	PIGL
1637	9	ENSG00000165282	PIGO
1638	20	ENSG00000124155	PIGT
1639	1	ENSG00000060642	PIGV
1640	1	ENSG00000133056	PIK3C2B
1641	3	ENSG00000121879	PIK3CA
1642	5	ENSG00000145675	PIK3R1
	19	ENSG00000105647	
1643	19	ENSG00000268173	PIK3R2
1644	12	ENSG00000090975	PITPNM2
1645	4	ENSG00000164093	PITX2
1646	10	ENSG00000107859	PITX3
1647	16	ENSG00000008710	PKD1
1648	7	ENSG00000158683	PKD1L1
1649	10	ENSG00000107593	PKD2L1
1650	6	ENSG00000170927	PKHD1
1651	8	ENSG00000205038	PKHD1L1
1652	1	ENSG00000065243	PKN2
1653	3	ENSG00000144837	PLA1A
1654	16	ENSG00000103066	PLA2G15
1655	22	ENSG00000184381	PLA2G6
1656	2	ENSG00000153246	PLA2R1
1657	20	ENSG00000182621	PLCB1
1658	20	ENSG00000101333	PLCB4
1659	10	ENSG00000138193	PLCE1
1660	1	ENSG00000149527	PLCH2
1661	19	ENSG00000105223	PLD3
1662	8	ENSG00000178209	PLEC
1663	1	ENSG00000143850	PLEKHA6
1664	11	ENSG00000166689	PLEKHA7
1665	2	ENSG00000115762	PLEKHB2
1666	1	ENSG00000187583	PLEKHN1
1667	4	ENSG00000142731	PLK4
1668	1	ENSG00000083444	PLOD1
1669	3	ENSG00000152952	PLOD2
1670	7	ENSG00000106397	PLOD3
1671	X	ENSG00000123560	PLP1
1672	10	ENSG00000120594	PLXDC2
1673	3	ENSG00000114554	PLXNA1
1674	3	ENSG00000004399	PLXND1

1675	15	ENSG00000140464	PML
1676	16	ENSG00000140650	PMM2
1677	19	ENSG00000039650	PNKP
1678	14	ENSG00000198805	PNP
1679	19	ENSG00000032444	PNPLA6
1680	2	ENSG00000138035	PNPT1
1681	3	ENSG00000164087	POC1A
1682	3	ENSG00000114631	PODXL2
1683	1	ENSG00000143442	POGZ
1684	X	ENSG00000101868	POLA1
1685	19	ENSG00000062822	POLD1
1686	15	ENSG00000140521	POLG
1687	17	ENSG00000256525	POLG2
1688	6	ENSG00000171453	POLR1C
1689	13	ENSG00000186184	POLR1D
1690	10	ENSG00000148606	POLR3A
1691	12	ENSG00000013503	POLR3B
1692	1	ENSG00000085998	POMGNT1
1693	8	ENSG00000185900	POMK
1694	13	ENSG00000132963	POMP
1695	9	ENSG00000130714	POMT1
1696	14	ENSG00000009830	POMT2
1697	7	ENSG00000127948	POR
1698	X	ENSG00000102312	PORCN
1699	3	ENSG00000064835	POU1F1
1700	2	ENSG00000198914	POU3F3
1701	X	ENSG00000196767	POU3F4
1702	17	ENSG00000170836	PPM1D
1703	1	ENSG00000143224	PPOX
1704	19	ENSG00000104881	PPP1R13L
1705	7	ENSG00000158528	PPP1R9A
1706	17	ENSG00000108819	PPP1R9B
1707	19	ENSG00000105568	PPP2R1A
1708	3	ENSG00000073711	PPP2R3A
1709	6	ENSG00000112640	PPP2R5D
1710	10	ENSG00000107758	PPP3CB
1711	1	ENSG00000131238	PPT1
1712	X	ENSG00000102103	PQBP1
1713	11	ENSG00000170325	PRDM10
1714	17	ENSG00000108946	PRKAR1A
1715	14	ENSG00000184304	PRKD1
1716	8	ENSG00000253729	PRKDC
1717	2	ENSG00000180228	PRKRA
1718	21	ENSG00000160310	PRMT2
1719	2	ENSG00000115718	PROC
1720	22	ENSG00000100033	PRODH
1721	19	ENSG00000250799	PRODH2
1722	2	ENSG00000155066	PROM2

1723	5	ENSG00000175325	PROP1
1724	19	ENSG00000105618	PRPF31
1725	17	ENSG00000174231	PRPF8
1726	6	ENSG00000112619	PRPH2
1727	X	ENSG00000147224	PRPS1
1728	9	ENSG00000130723	PRRC2B
1729	16	ENSG00000167371	PRRT2
1730	4	ENSG00000164099	PRSS12
1731	2	ENSG00000237412	PRSS56
1732	9	ENSG00000106772	PRUNE2
1733	10	ENSG00000197746	PSAP
1734	9	ENSG00000135069	PSAT1
1735	5	ENSG00000146005	PSD2
1736	14	ENSG00000080815	PSEN1
1737	6	ENSG00000204264	PSMB8
1738	17	ENSG00000108344	PSMD3
1739	6	ENSG00000180822	PSMG4
1740	7	ENSG00000146733	PSPH
1741	9	ENSG00000185920	PTCH1
1742	1	ENSG00000117425	PTCH2
1743	X	ENSG00000165186	PTCHD1
1744	6	ENSG00000244694	PTCHD4
1745	8	ENSG00000156471	PTDSS1
1746	10	ENSG00000171862	PTEN
1747	19	ENSG00000160013	PTGIR
1748	3	ENSG00000160801	PTH1R
1749	12	ENSG00000087494	PTHLH
1750	12	ENSG00000179295	PTPN11
1751	1	ENSG00000143851	PTPN7
1752	20	ENSG00000132670	PTPRA
1753	19	ENSG00000105426	PTPRS
1754	20	ENSG00000196090	PTPRT
1755	17	ENSG00000141378	PTRH2
1756	11	ENSG00000150787	PTS
1757	2	ENSG00000055917	PUM2
1758	5	ENSG00000185129	PURA
1759	12	ENSG00000177192	PUS1
1760	1	ENSG00000143217	PVRL4
1761	15	ENSG00000279050	PWAR1
1762	15	ENSG00000259905	PWRN1
1763	2	ENSG00000130508	PXDN
1764	17	ENSG00000183010	PYCR1
1765	1	ENSG00000143811	PYCR2
1766	14	ENSG00000100504	PYGL
1767	4	ENSG00000151552	QDPR
1768	16	ENSG00000103485	QPRT
1769	10	ENSG00000099246	RAB18
1770	6	ENSG00000112210	RAB23

1771	15	ENSG00000069974	RAB27A
1772	8	ENSG00000104388	RAB2A
1773	18	ENSG00000168461	RAB31
1774	X	ENSG00000155961	RAB39B
1775	2	ENSG00000115839	RAB3GAP1
1776	1	ENSG00000118873	RAB3GAP2
1777	1	ENSG00000152061	RABGAP1L
1778	1	ENSG00000137955	RABGGTB
1779	7	ENSG00000136238	RAC1
1780	8	ENSG00000164754	RAD21
1781	15	ENSG00000051180	RAD51
1782	3	ENSG00000164080	RAD54L2
1783	3	ENSG00000132155	RAF1
1784	17	ENSG00000108557	RAI1
1785	20	ENSG00000188559	RALGAPA2
1786	2	ENSG00000153201	RANBP2
1787	3	ENSG00000077092	RARB
1788	5	ENSG00000113643	RARS
1789	6	ENSG00000146282	RARS2
1790	5	ENSG00000145715	RASA1
1791	9	ENSG00000165105	RASEF
1792	4	ENSG00000138670	RASGEF1B
1793	15	ENSG00000172575	RASGRP1
1794	10	ENSG00000107551	RASSF4
1795	1	ENSG00000266094	RASSF5
1796	18	ENSG00000134438	RAX
1797	1	ENSG00000162521	RBBP4
1798	16	ENSG00000122257	RBBP6
1799	18	ENSG00000101773	RBBP8
1800	16	ENSG00000078328	RBFOX1
1801	X	ENSG00000182872	RBM10
1802	20	ENSG00000244462	RBM12
1803	14	ENSG00000100461	RBM23
1804	7	ENSG00000106344	RBM28
1805	7	ENSG00000184863	RBM33
1806	4	ENSG00000163694	RBM47
1807	1	ENSG00000265241	RBM8A
1808	2	ENSG00000153250	RBMS1
1809	22	ENSG00000100387	RBX1
1810	19	ENSG00000080511	RDH8
1811	8	ENSG00000160957	RECQL4
1812	5	ENSG00000129625	REEP5
1813	2	ENSG00000162924	REL
1814	7	ENSG00000189056	RELN
1815	12	ENSG00000111404	RERGL
1816	10	ENSG00000165731	RET
1817	17	ENSG00000169733	RFNG
1818	3	ENSG00000163933	RFT1

1819	3	ENSG00000131378	RFTN1
1820	9	ENSG00000080298	RFX3
1821	6	ENSG00000185002	RFX6
1822	15	ENSG00000181827	RFX7
1823	1	ENSG00000121446	RGSL1
1824	16	ENSG00000007384	RHBDF1
1825	8	ENSG00000008853	RHOBTB2
1826	12	ENSG00000111785	RIC8B
1827	5	ENSG00000164327	RICTOR
1828	21	ENSG00000183421	RIPK4
1829	1	ENSG00000143622	RIT1
1830	15	ENSG00000140522	RLBP1
1831	9	ENSG00000269900	RMRP
1832	19	ENSG00000104889	RNASEH2A
1833	13	ENSG00000136104	RNASEH2B
1834	11	ENSG00000172922	RNASEH2C
1835	6	ENSG00000026297	RNASET2
1836	X	ENSG00000125352	RNF113A
1837	18	ENSG00000101695	RNF125
1838	17	ENSG00000181481	RNF135
1839	5	ENSG00000145860	RNF145
1840	11	ENSG00000173456	RNF26
1841	11	ENSG00000023191	RNH1
1842	2	ENSG00000264229	RNU4ATAC
1843	3	ENSG00000169855	ROBO1
1844	3	ENSG00000185008	ROBO2
1845	11	ENSG00000154134	ROBO3
1846	16	ENSG00000067836	ROGDI
1847	9	ENSG00000169071	ROR2
1848	8	ENSG00000104237	RP1
1849	16	ENSG00000205018	RP11-830F9.6
1850	X	ENSG00000102218	RP2
1851	1	ENSG00000116745	RPE65
1852	14	ENSG00000092200	RPGRIP1
1853	16	ENSG00000103494	RPGRIP1L
1854	12	ENSG00000089169	RPH3A
1855	4	ENSG00000109475	RPL34
1856	1	ENSG00000163125	RPRD2
1857	19	ENSG00000105372	RPS19
1858	2	ENSG00000143947	RPS27A
1859	X	ENSG00000177189	RPS6KA3
1860	1	ENSG00000215853	RPTN
1861	17	ENSG00000141564	RPTOR
1862	3	ENSG00000156990	RPUSD3
1863	8	ENSG00000048392	RRM2B
1864	11	ENSG00000132275	RRP8
1865	11	ENSG00000048649	RSF1
1866	16	ENSG00000171490	RSL1D1

1867	21	ENSG00000160188	RSPH1
1868	19	ENSG00000104941	RSPH6A
1869	20	ENSG00000101282	RSPO4
1870	20	ENSG00000258366	RTEL1
1871	15	ENSG00000137815	RTF1
1872	17	ENSG00000185924	RTN4RL1
1873	11	ENSG00000186907	RTN4RL2
1874	18	ENSG00000176225	RTTN
1875	21	ENSG00000159216	RUNX1
1876	6	ENSG00000124813	RUNX2
1877	19	ENSG00000183207	RUVBL2
1878	3	ENSG00000163602	RYBP
1879	19	ENSG00000196218	RYR1
1880	1	ENSG00000198626	RYR2
1881	19	ENSG00000125910	S1PR4
1882	13	ENSG00000151835	SACS
1883	16	ENSG00000103449	SALL1
1884	20	ENSG00000101115	SALL4
1885	7	ENSG00000205413	SAMD9
1886	20	ENSG00000101347	SAMHD1
1887	2	ENSG00000136715	SAP130
1888	1	ENSG00000156876	SASS6
1889	2	ENSG00000119042	SATB2
1890	7	ENSG00000126524	SBDS
1891	22	ENSG00000100241	SBF1
1892	11	ENSG00000133812	SBF2
1893	11	ENSG00000109929	SC5D
1894	21	ENSG00000156304	SCAF4
1895	3	ENSG00000114650	SCAP
1896	4	ENSG00000138760	SCARB2
1897	22	ENSG00000244486	SCARF2
1898	4	ENSG00000184178	SCFD2
1899	3	ENSG00000168356	SCN11A
1900	2	ENSG00000144285	SCN1A
1901	19	ENSG00000105711	SCN1B
1902	2	ENSG00000136531	SCN2A
1903	2	ENSG00000153253	SCN3A
1904	17	ENSG00000007314	SCN4A
1905	12	ENSG00000196876	SCN8A
1906	1	ENSG00000162572	SCNN1D
1907	17	ENSG00000133028	SCO1
1908	22	ENSG00000130489	SCO2
1909	1	ENSG00000054282	SDCCAG8
1910	5	ENSG00000073578	SDHA
1911	19	ENSG00000205138	SDHAF1
1912	7	ENSG00000146555	SDK1
1913	17	ENSG00000069188	SDK2
1914	16	ENSG00000103184	SEC14L5

1915	9	ENSG00000148396	SEC16A
1916	20	ENSG00000101310	SEC23B
1917	10	ENSG00000107651	SEC23IP
1918	7	ENSG00000075223	SEMA3C
1919	7	ENSG00000170381	SEMA3E
1920	15	ENSG00000138623	SEMA7A
1921	3	ENSG00000119231	SENP5
1922	4	ENSG00000109618	SEPSECS
1923	6	ENSG00000122335	SERAC1
1924	18	ENSG00000166634	SERPINB12
1925	6	ENSG00000124570	SERPINB6
1926	22	ENSG00000099937	SERPIND1
1927	9	ENSG00000119335	SET
1928	18	ENSG00000152217	SETBP1
1929	12	ENSG00000139718	SETD1B
1930	3	ENSG00000181555	SETD2
1931	21	ENSG00000185917	SETD4
1932	3	ENSG00000168137	SETD5
1933	1	ENSG00000143379	SETDB1
1934	11	ENSG00000168066	SF1
1935	1	ENSG00000183431	SF3A3
1936	1	ENSG00000143368	SF3B4
1937	10	ENSG00000183605	SFXN4
1938	7	ENSG00000127990	SGCE
1939	20	ENSG00000101049	SGK2
1940	14	ENSG00000126821	SGPP1
1941	17	ENSG00000181523	SGSH
1942	22	ENSG00000100359	SGSM3
1943	5	ENSG00000197860	SGTB
1944	9	ENSG00000095370	SH2D3C
1945	4	ENSG00000109686	SH3D19
1946	5	ENSG00000174705	SH3PXD2B
1947	11	ENSG00000162105	SHANK2
1948	22	ENSG00000251322	SHANK3
1949	15	ENSG00000185634	SHC4
1950	7	ENSG00000164690	SHH
1951	19	ENSG00000160410	SHKBP1
1952	10	ENSG00000108061	SHOC2
1953	X	ENSG00000185960	SHOX
1954	X	ENSG00000158352	SHROOM4
1955	19	ENSG00000129450	SIGLEC9
1956	11	ENSG00000160584	SIK3
1957	5	ENSG00000120725	SIL1
1958	15	ENSG00000169375	SIN3A
1959	19	ENSG00000127511	SIN3B
1960	20	ENSG00000101307	SIRPB1
1961	14	ENSG00000126778	SIX1
1962	2	ENSG00000138083	SIX3

1963	19	ENSG00000177045	SIX5
1964	17	ENSG00000141293	SKAP1
1965	1	ENSG00000157933	SKI
1966	3	ENSG00000136603	SKIL
1967	6	ENSG00000204351	SKIV2L
1968	1	ENSG00000162723	SLAMF9
1969	4	ENSG00000120519	SLC10A7
1970	15	ENSG00000074803	SLC12A1
1971	15	ENSG00000140199	SLC12A6
1972	7	ENSG00000081800	SLC13A1
1973	1	ENSG00000155380	SLC16A1
1974	X	ENSG00000147100	SLC16A2
1975	6	ENSG00000119899	SLC17A5
1976	1	ENSG00000117479	SLC19A2
1977	2	ENSG00000135917	SLC19A3
1978	5	ENSG00000197375	SLC22A5
1979	20	ENSG00000185052	SLC24A3
1980	13	ENSG00000102743	SLC25A15
1981	3	ENSG00000178537	SLC25A20
1982	3	ENSG00000144659	SLC25A38
1983	4	ENSG00000151729	SLC25A4
1984	5	ENSG00000155850	SLC26A2
1985	1	ENSG00000174502	SLC26A9
1986	9	ENSG00000167114	SLC27A4
1987	1	ENSG00000117394	SLC2A1
1988	20	ENSG00000197496	SLC2A10
1989	4	ENSG00000109667	SLC2A9
1990	1	ENSG00000196660	SLC30A10
1991	2	ENSG00000115194	SLC30A3
1992	3	ENSG00000169359	SLC33A1
1993	X	ENSG00000102100	SLC35A2
1994	1	ENSG00000117620	SLC35A3
1995	11	ENSG00000181830	SLC35C1
1996	1	ENSG00000116704	SLC35D1
1997	2	ENSG00000115084	SLC35F5
1998	5	ENSG00000186335	SLC36A2
1999	10	ENSG00000148482	SLC39A12
2000	11	ENSG00000165915	SLC39A13
2001	14	ENSG00000165794	SLC39A2
2002	14	ENSG00000029364	SLC39A9
2003	11	ENSG00000168003	SLC3A2
2004	1	ENSG00000158715	SLC45A3
2005	17	ENSG00000076351	SLC46A1
2006	4	ENSG00000080493	SLC4A4
2007	20	ENSG00000101276	SLC52A3
2008	17	ENSG00000154025	SLC5A10
2009	16	ENSG00000158865	SLC5A11
2010	19	ENSG00000105641	SLC5A5

2011	2	ENSG00000138074	SLC5A6
2012	3	ENSG00000157103	SLC6A1
2013	12	ENSG00000010379	SLC6A13
2014	1	ENSG00000197106	SLC6A17
2015	5	ENSG00000174358	SLC6A19
2016	3	ENSG00000163817	SLC6A20
2017	5	ENSG00000142319	SLC6A3
2018	11	ENSG00000165970	SLC6A5
2019	X	ENSG00000130821	SLC6A8
2020	1	ENSG00000196517	SLC6A9
2021	14	ENSG00000155465	SLC7A7
2022	5	ENSG00000066230	SLC9A3
2023	X	ENSG00000198689	SLC9A6
2024	12	ENSG00000205754	SLCO1B7
2025	16	ENSG00000188827	SLX4
2026	18	ENSG00000141646	SMAD4
2027	15	ENSG00000137834	SMAD6
2028	9	ENSG00000080503	SMARCA2
2029	19	ENSG00000127616	SMARCA4
2030	2	ENSG00000138375	SMARCAL1
2031	22	ENSG00000099956	SMARCB1
2032	12	ENSG00000066117	SMARCD1
2033	17	ENSG00000073584	SMARCE1
2034	X	ENSG00000072501	SMC1A
2035	10	ENSG00000108055	SMC3
2036	14	ENSG00000100796	SMEK1
2037	14	ENSG00000198732	SMOC1
2038	11	ENSG00000166311	SMPD1
2039	X	ENSG00000102172	SMS
2040	22	ENSG00000183963	SMTN
2041	17	ENSG00000188176	SMTNL2
2042	9	ENSG00000122692	SMU1
2043	20	ENSG00000132639	SNAP25
2044	22	ENSG00000099940	SNAP29
2045	15	ENSG00000201831	SNORD115-1
2046	15	ENSG00000207063	SNORD116-1
2047	20	ENSG00000101298	SNPH
2048	2	ENSG00000144028	SNRNP200
2049	19	ENSG00000077312	SNRPA
2050	20	ENSG00000125835	SNRPB
2051	15	ENSG00000128739	SNRPN
2052	17	ENSG00000002919	SNX11
2053	15	ENSG00000157734	SNX22
2054	6	ENSG00000112320	SOBP
2055	20	ENSG00000149639	SOGA1
	6	ENSG00000214338	
2056	6	ENSG00000255330	SOGA3
2057	21	ENSG00000159140	SON

2058	11	ENSG00000137642	SORL1
2059	2	ENSG00000115904	SOS1
2060	22	ENSG00000100146	SOX10
2061	8	ENSG00000164736	SOX17
2062	3	ENSG00000181449	SOX2
2063	3	ENSG00000242808	SOX2-OT
2064	X	ENSG00000134595	SOX3
2065	12	ENSG00000134532	SOX5
2066	17	ENSG00000125398	SOX9
2067	8	ENSG00000104450	SPAG1
2068	5	ENSG00000113140	SPARC
2069	2	ENSG00000021574	SPAST
2070	20	ENSG00000158480	SPATA2
2071	6	ENSG00000124664	SPDEF
2072	22	ENSG00000100014	SPECC1L
2073	1	ENSG00000065526	SPEN
2074	15	ENSG00000104133	SPG11
2075	16	ENSG00000197912	SPG7
2076	19	ENSG00000063176	SPHK2
2077	9	ENSG00000106723	SPIN1
2078	X	ENSG00000204271	SPIN3
2079	5	ENSG00000133710	SPINK5
2080	5	ENSG00000152377	SPOCK1
2081	19	ENSG00000005206	SPPL2B
2082	2	ENSG00000116096	SPR
2083	15	ENSG00000166068	SPRED1
2084	1	ENSG00000171621	SPSB1
2085	9	ENSG00000197694	SPTAN1
2086	14	ENSG00000070182	SPTB
2087	2	ENSG00000115306	SPTBN1
2088	11	ENSG00000173898	SPTBN2
2089	16	ENSG00000080603	SRCAP
2090	4	ENSG00000128039	SRD5A3
2091	17	ENSG00000072310	SREBF1
2092	1	ENSG00000266028	SRGAP2
2093	3	ENSG00000196220	SRGAP3
2094	1	ENSG00000133226	SRRM1
2095	12	ENSG00000139767	SRRM4
2096	Y	ENSG00000184895	SRY
2097	17	ENSG00000141298	SSH2
2098	12	ENSG00000123096	SSPN
2099	12	ENSG00000185482	STAC3
2100	3	ENSG00000118007	STAG1
2101	X	ENSG00000101972	STAG2
2102	2	ENSG00000124356	STAMBP
2103	8	ENSG00000147465	STAR
2104	2	ENSG00000115415	STAT1
2105	2	ENSG00000138378	STAT4

2106	1	ENSG00000123473	STIL
2107	4	ENSG00000109689	STIM2
2108	15	ENSG00000137868	STRA6
2109	X	ENSG00000101846	STS
2110	11	ENSG00000134910	STT3A
2111	3	ENSG00000163527	STT3B
2112	16	ENSG00000103266	STUB1
2113	9	ENSG00000136854	STXBP1
2114	13	ENSG00000136143	SUCLA2
2115	2	ENSG00000163541	SUCLG1
2116	10	ENSG00000107882	SUFU
2117	3	ENSG00000144455	SUMF1
2118	12	ENSG00000139531	SUOX
2119	17	ENSG00000109111	SUPT6H
2120	9	ENSG00000148290	SURF1
2121	1	ENSG00000143502	SUSD4
2122	1	ENSG00000159164	SV2A
2123	9	ENSG00000165124	SVEP1
2124	X	ENSG00000008056	SYN1
2125	3	ENSG00000157152	SYN2
2126	6	ENSG00000131018	SYNE1
2127	14	ENSG00000054654	SYNE2
2128	6	ENSG00000197283	SYNGAP1
2129	21	ENSG00000159082	SYNJ1
2130	X	ENSG00000102003	SYP
2131	22	ENSG00000100324	TAB1
2132	12	ENSG00000166863	TAC3
2133	10	ENSG00000138162	TACC2
2134	17	ENSG00000136463	TACO1
2135	X	ENSG00000147133	TAF1
2136	8	ENSG00000064313	TAF2
2137	6	ENSG00000137413	TAF8
2138	2	ENSG00000115183	TANC1
2139	17	ENSG00000170921	TANC2
2140	16	ENSG00000198650	TAT
2141	X	ENSG00000102125	TAZ
2142	4	ENSG00000065882	TBC1D1
2143	19	ENSG00000104946	TBC1D17
2144	20	ENSG00000125875	TBC1D20
2145	6	ENSG00000065491	TBC1D22B
2146	16	ENSG00000162065	TBC1D24
2147	13	ENSG00000136111	TBC1D4
2148	1	ENSG00000116957	TBCE
2149	3	ENSG00000177565	TBL1XR1
2150	16	ENSG00000183751	TBL3
2151	2	ENSG00000136535	TBR1
2152	11	ENSG00000154144	TBRG1
2153	22	ENSG00000184058	TBX1

2154	7	ENSG00000164532	TBX20
	X	ENSG00000122145	
2155	X	ENSG00000281700	TBX22
2156	12	ENSG00000135111	TBX3
2157	17	ENSG00000121075	TBX4
2158	12	ENSG00000089225	TBX5
2159	7	ENSG00000059377	TBXAS1
2160	15	ENSG00000140262	TCF12
2161	22	ENSG00000100207	TCF20
2162	19	ENSG00000071564	TCF3
2163	18	ENSG00000196628	TCF4
2164	10	ENSG00000148737	TCF7L2
2165	22	ENSG00000185339	TCN2
2166	5	ENSG00000070814	TCOF1
2167	12	ENSG00000168778	TCTN2
2168	10	ENSG00000119977	TCTN3
2169	10	ENSG00000095627	TDRD1
2170	2	ENSG00000218819	TDRD15
2171	12	ENSG00000197905	TEAD4
2172	11	ENSG00000109927	TECTA
2173	9	ENSG00000120156	TEK
2174	17	ENSG00000125409	TEKT3
2175	16	ENSG00000153060	TEKT5
2176	5	ENSG00000164362	TERT
2177	6	ENSG00000137203	TFAP2A
2178	6	ENSG00000008196	TFAP2B
2179	X	ENSG00000068323	TFE3
2180	7	ENSG00000105967	TFEC
2181	3	ENSG00000072274	TFRC
2182	8	ENSG00000042832	TG
2183	13	ENSG00000088451	TGDS
2184	19	ENSG00000105329	TGFB1
2185	9	ENSG00000106799	TGFBR1
2186	3	ENSG00000163513	TGFBR2
2187	18	ENSG00000177426	TGIF1
2188	20	ENSG00000166948	TGM6
2189	11	ENSG00000180176	TH
2190	16	ENSG00000131652	THOC6
2191	17	ENSG00000126351	THRA
2192	3	ENSG00000151090	THRB
2193	19	ENSG00000104980	TIMM44
2194	X	ENSG00000126953	TIMM8A
2195	9	ENSG00000119139	TJP2
2196	19	ENSG00000105289	TJP3
2197	16	ENSG00000166548	TK2
2198	3	ENSG00000163931	TKT
2199	19	ENSG00000065717	TLE2
2200	15	ENSG00000140332	TLE3

2201	17	ENSG00000146872	TLK2
2202	4	ENSG00000174125	TLR1
2203	12	ENSG00000064115	TM7SF3
2204	15	ENSG00000166069	TMCO5A
2205	17	ENSG00000185332	TMEM105
2206	4	ENSG00000168936	TMEM129
2207	11	ENSG00000149483	TMEM138
2208	4	ENSG00000170006	TMEM154
2209	4	ENSG00000134851	TMEM165
2210	19	ENSG00000105518	TMEM205
2211	2	ENSG00000119777	TMEM214
2212	11	ENSG00000187049	TMEM216
2213	16	ENSG00000205084	TMEM231
2214	5	ENSG00000186952	TMEM232
2215	2	ENSG00000155755	TMEM237
2216	1	ENSG00000205090	TMEM240
2217	3	ENSG00000169964	TMEM42
2218	3	ENSG00000181458	TMEM45A
2219	12	ENSG00000118600	TMEM5
2220	1	ENSG00000143001	TMEM61
2221	6	ENSG00000137216	TMEM63B
2222	14	ENSG00000165548	TMEM63C
2223	8	ENSG00000164953	TMEM67
2224	8	ENSG00000175606	TMEM70
2225	2	ENSG00000153214	TMEM87B
2226	16	ENSG00000129925	TMEM8A
2227	21	ENSG00000154646	TMPRSS15
2228	22	ENSG00000187045	TMPRSS6
2229	12	ENSG00000133687	TMTC1
2230	14	ENSG00000185215	TNFAIP2
2231	1	ENSG00000157873	TNFRSF14
2232	3	ENSG00000061938	TNK2
2233	8	ENSG00000173273	TNKS
2234	11	ENSG00000149115	TNKS1BP1
2235	10	ENSG00000107854	TNKS2
2236	1	ENSG00000120332	TNN
2237	7	ENSG00000064419	TNPO3
2238	7	ENSG00000182095	TNRC18
2239	16	ENSG00000090905	TNRC6A
2240	6	ENSG00000168477	TNXB
2241	17	ENSG00000141232	TOB1
2242	3	ENSG00000077097	TOP2B
2243	9	ENSG00000136827	TOR1A
2244	8	ENSG00000198846	TOX
2245	3	ENSG00000073282	TP63
2246	7	ENSG00000196511	TPK1
2247	2	ENSG00000115705	TPO
2248	11	ENSG00000166340	TPP1

2249	3	ENSG00000188001	TPRG1
2250	9	ENSG00000176058	TPRN
2251	11	ENSG00000175104	TRAF6
2252	3	ENSG00000182606	TRAK1
2253	X	ENSG00000196459	TRAPPC2
2254	8	ENSG00000167632	TRAPPC9
	3	ENSG00000213689	
2255	3	ENSG00000280804	TREX1
2256	9	ENSG00000119401	TRIM32
2257	17	ENSG00000108395	TRIM37
2258	11	ENSG00000132256	TRIM5
2259	11	ENSG00000166007	TRIM51HP
2260	11	ENSG00000121236	TRIM6
2261	14	ENSG00000100505	TRIM9
2262	5	ENSG00000038382	TRIO
2263	22	ENSG00000100106	TRIOBP
2264	19	ENSG00000125733	TRIP10
2265	14	ENSG00000100815	TRIP11
2266	2	ENSG00000153827	TRIP12
2267	4	ENSG00000145331	TRMT10A
2268	22	ENSG00000099899	TRMT2A
2269	X	ENSG00000072315	TRPC5
2270	15	ENSG00000134160	TRPM1
2271	21	ENSG00000142185	TRPM2
2272	11	ENSG00000070985	TRPM5
2273	9	ENSG00000119121	TRPM6
2274	15	ENSG00000092439	TRPM7
2275	8	ENSG00000104447	TRPS1
2276	12	ENSG00000111199	TRPV4
2277	7	ENSG00000196367	TRRAP
2278	9	ENSG00000165699	TSC1
2279	16	ENSG00000103197	TSC2
2280	3	ENSG00000154743	TSEN2
2281	17	ENSG00000182173	TSEN54
2282	12	ENSG00000123297	TSFM
2283	1	ENSG00000134200	TSHB
2284	14	ENSG00000165409	TSHR
2285	19	ENSG00000126467	TSKS
2286	8	ENSG00000171045	TSNARE1
2287	7	ENSG00000106025	TSPAN12
2288	X	ENSG00000156298	TSPAN7
2289	6	ENSG00000189241	TSPYL1
2290	6	ENSG00000146216	TTBK1
2291	17	ENSG00000011295	TTC19
2292	2	ENSG00000123607	TTC21B
2293	2	ENSG00000018699	TTC27
2294	5	ENSG00000198677	TTC37
2295	9	ENSG00000155158	TTC39B

2296	2	ENSG00000068724	TTC7A
2297	14	ENSG00000165533	TTC8
2298	11	ENSG00000162222	TTC9C
2299	8	ENSG00000129696	TTI2
2300	2	ENSG00000155657	TTN
2301	12	ENSG00000167552	TUBA1A
2302	22	ENSG00000183785	TUBA8
2303	6	ENSG00000137267	TUBB2A
2304	6	ENSG00000137285	TUBB2B
	16	ENSG00000198211	
2305	16	ENSG00000258947	TUBB3
2306	19	ENSG00000104833	TUBB4A
2307	22	ENSG00000128159	TUBGCP6
2308	8	ENSG00000104723	TUSC3
2309	11	ENSG00000149016	TUT1
2310	7	ENSG00000122691	TWIST1
2311	1	ENSG00000117862	TXNDC12
2312	11	ENSG00000077498	TYR
2313	9	ENSG00000107165	TYRP1
2314	X	ENSG00000130985	UBA1
2315	X	ENSG00000077721	UBE2A
2316	3	ENSG00000170142	UBE2E1
2317	15	ENSG00000114062	UBE3A
2318	12	ENSG00000151148	UBE3B
2319	1	ENSG00000130939	UBE4B
2320	13	ENSG00000122042	UBL3
2321	15	ENSG00000159459	UBR1
2322	8	ENSG00000104517	UBR5
2323	1	ENSG00000116750	UCHL5
2324	2	ENSG00000241635	UGT1A1
2325	5	ENSG00000145626	UGT3A1
2326	15	ENSG00000140474	ULK3
2327	21	ENSG00000177398	UMODL1
2328	3	ENSG00000114491	UMPS
2329	2	ENSG00000144406	UNC80
2330	22	ENSG00000100024	UPB1
2331	19	ENSG00000005007	UPF1
2332	10	ENSG00000151461	UPF2
2333	X	ENSG00000125351	UPF3B
2334	3	ENSG00000010256	UQCRC1
2335	16	ENSG00000140740	UQCRC2
2336	5	ENSG00000164405	UQCRQ
2337	3	ENSG00000159650	UROC1
2338	10	ENSG00000188690	UROS
2339	18	ENSG00000101557	USP14
2340	X	ENSG00000124486	USP9X
2341	4	ENSG00000163945	UVSSA
2342	16	ENSG00000103043	VAC14

2343	1	ENSG00000173218	VANGL1
2344	18	ENSG00000101558	VAPA
2345	10	ENSG00000148704	VAX1
2346	12	ENSG00000111424	VDR
2347	17	ENSG00000136451	VEZF1
2348	3	ENSG00000136059	VILL
2349	6	ENSG00000146469	VIP
2350	14	ENSG00000151445	VIPAS39
2351	16	ENSG00000167397	VKORC1
2352	9	ENSG00000147852	VLDLR
2353	8	ENSG00000132549	VPS13B
2354	1	ENSG00000048707	VPS13D
2355	15	ENSG00000184056	VPS33B
2356	8	ENSG00000155975	VPS37A
2357	15	ENSG00000166887	VPS39
2358	17	ENSG00000141252	VPS53
2359	14	ENSG00000100749	VRK1
2360	2	ENSG00000028116	VRK2
2361	14	ENSG00000119614	VSX2
2362	10	ENSG00000151532	VTI1A
2363	10	ENSG00000165816	VWA2
2364	7	ENSG00000146530	VWDE
2365	12	ENSG00000110799	VWF
2366	10	ENSG00000095787	WAC
2367	10	ENSG00000128815	WDFY4
2368	2	ENSG00000143951	WDPCP
2369	10	ENSG00000120008	WDR11
2370	4	ENSG00000157796	WDR19
2371	9	ENSG00000148225	WDR31
2372	2	ENSG00000118965	WDR35
2373	X	ENSG00000196998	WDR45
2374	7	ENSG00000126870	WDR60
2375	19	ENSG00000075702	WDR62
2376	15	ENSG00000177082	WDR73
2377	17	ENSG00000167716	WDR81
2378	11	ENSG00000166483	WEE1
2379	4	ENSG00000109501	WFS1
2380	4	ENSG00000109685	WHSC1
2381	19	ENSG00000011451	WIZ
2382	12	ENSG00000060237	WNK1
2383	9	ENSG00000165238	WNK2
2384	12	ENSG00000125084	WNT1
2385	12	ENSG00000169884	WNT10B
2386	17	ENSG00000108379	WNT3
2387	3	ENSG00000114251	WNT5A
2388	3	ENSG00000154764	WNT7A
2389	5	ENSG00000061492	WNT8A
2390	8	ENSG00000165392	WRN

2391	11	ENSG00000184937	WT1
2392	5	ENSG00000113645	WWC1
2393	16	ENSG00000186153	WWOX
2394	3	ENSG00000168334	XIRP1
2395	2	ENSG00000163092	XIRP2
2396	9	ENSG00000136936	XPA
2397	3	ENSG00000154767	XPC
2398	22	ENSG00000196236	XPNPEP3
2399	1	ENSG00000143324	XPR1
2400	16	ENSG00000103489	XYLT1
2401	11	ENSG00000137693	YAP1
2402	11	ENSG00000174851	YIF1A
2403	17	ENSG00000108953	YWHAE
2404	14	ENSG00000126804	ZBTB1
2405	11	ENSG00000109906	ZBTB16
2406	1	ENSG00000179456	ZBTB18
2407	3	ENSG00000181722	ZBTB20
2408	3	ENSG00000241560	ZBTB20-AS1
2409	6	ENSG00000112365	ZBTB24
2410	1	ENSG00000185278	ZBTB37
2411	1	ENSG00000177888	ZBTB41
2412	X	ENSG00000126970	ZC4H2
2413	2	ENSG00000204186	ZDBF2
2414	X	ENSG00000188706	ZDHHC9
2415	2	ENSG00000169554	ZEB2
2416	8	ENSG00000091656	ZFHX4
2417	6	ENSG00000204644	ZFP57
2418	14	ENSG00000072121	ZFYVE26
2419	4	ENSG00000159733	ZFYVE28
2420	13	ENSG00000043355	ZIC2
2421	X	ENSG00000156925	ZIC3
2422	19	ENSG00000269699	ZIM2
2423	1	ENSG00000084073	ZMPSTE24
2424	1	ENSG00000146463	ZMYM4
2425	10	ENSG00000015171	ZMYND11
2426	20	ENSG00000125846	ZNF133
2427	2	ENSG00000115568	ZNF142
2428	11	ENSG00000166478	ZNF143
2429	19	ENSG00000167635	ZNF146
2430	19	ENSG00000105497	ZNF175
2431	3	ENSG00000186448	ZNF197
2432	17	ENSG00000010244	ZNF207
2433	11	ENSG00000149054	ZNF215
2434	20	ENSG00000171940	ZNF217
2435	19	ENSG00000256294	ZNF225
2436	20	ENSG00000131061	ZNF341
2437	1	ENSG00000160094	ZNF362
2438	10	ENSG00000138311	ZNF365

2439	X	ENSG00000147124	ZNF41
2440	7	ENSG00000181444	ZNF467
2441	7	ENSG00000185177	ZNF479
2442	18	ENSG00000101493	ZNF516
2443	8	ENSG00000197363	ZNF517
2444	19	ENSG00000198597	ZNF536
2445	19	ENSG00000118156	ZNF541
2446	19	ENSG00000251369	ZNF550
2447	15	ENSG00000166716	ZNF592
2448	1	ENSG00000122482	ZNF644
2449	X	ENSG00000251192	ZNF674
2450	X	ENSG00000147180	ZNF711
2451	12	ENSG00000139651	ZNF740
2452	16	ENSG00000170100	ZNF778
2453	7	ENSG00000204946	ZNF783
2454	19	ENSG00000223547	ZNF844
2455	20	ENSG00000124201	ZNFX1
2456	10	ENSG00000019995	ZRANB1
2457	2	ENSG00000121988	ZRANB3
2458	6	ENSG00000158691	ZSCAN12
2459	5	ENSG00000130449	ZSWIM6
2460	10	ENSG00000122952	ZWINT
2461	1	ENSG00000036549	ZZZ3

Schizophrenia

Gene Number	Chromosome	Gene ID	Gene Name
1	12	ENSG00000150967	ABCB9
2	14	ENSG00000280188	AC005477.1
3	16	ENSG00000102977	ACD
4	16	ENSG00000166743	ACSM1
5	20	ENSG00000101442	ACTR5
6	2	ENSG00000114948	ADAM23
7	5	ENSG00000145536	ADAMTS16
8	5	ENSG00000049192	ADAMTS6
9	15	ENSG00000156218	ADAMTSL3
10	11	ENSG00000148926	ADM
11	22	ENSG00000100077	ADRBK2
12	15	ENSG00000166748	AGBL1
13	14	ENSG00000142208	AKT1
14	1	ENSG00000117020	AKT3
15	3	ENSG00000023330	ALAS1
16	16	ENSG00000149925	ALDOA
17	3	ENSG00000242849	ALDOAP1
18	11	ENSG00000110497	AMBRA1
19	11	ENSG00000166025	AMOTL1
20	10	ENSG00000151150	ANK3
21	17	ENSG00000185722	ANKFY1

22	12	ENSG00000167612	ANKRD33
23	2	ENSG00000065413	ANKRD44
24	15	ENSG00000230778	ANKRD63
25	12	ENSG00000185046	ANKS1B
26	11	ENSG00000171714	ANO5
27	1	ENSG00000143401	ANP32E
28	4	ENSG00000163297	ANTXR2
29	9	ENSG00000135046	ANXA1
30	1	ENSG00000117362	APH1A
31	22	ENSG00000128335	APOL2
32	22	ENSG00000100336	APOL4
33	14	ENSG00000256053	APOPT1
34	11	ENSG00000175220	ARHGAP1
35	3	ENSG00000031081	ARHGAP31
36	X	ENSG00000089820	ARHGAP4
37	10	ENSG00000138175	ARL3
38	12	ENSG00000182196	ARL6IP4
39	11	ENSG00000133794	ARNTL
40	10	ENSG00000214435	AS3MT
41	16	ENSG00000174939	ASPHD1
42	9	ENSG00000148219	ASTN2
43	11	ENSG00000175224	ATG13
44	1	ENSG00000018625	ATP1A2
45	12	ENSG00000174437	ATP2A2
46	2	ENSG00000230482	ATP5G2P3
47	19	ENSG00000105341	ATP5SL
48	17	ENSG00000171953	ATPAF2
49	3	ENSG00000163635	ATXN7
50	9	ENSG00000148090	AUH
51	14	ENSG00000166170	BAG5
52	6	ENSG00000204463	BAG6
53	4	ENSG00000153064	BANK1
54	3	ENSG00000163930	BAP1
55	14	ENSG00000127152	BCL11B
56	1	ENSG00000116128	BCL9
57	2	ENSG00000152430	BOLL
58	22	ENSG00000100425	BRD1
59	11	ENSG00000233436	BTBD18
60	6	ENSG00000124508	BTN2A2
61	6	ENSG00000026950	BTN3A1
62	6	ENSG00000186470	BTN3A2
63	10	ENSG00000166275	C10orf32
64	11	ENSG00000211450	C11orf31
65	11	ENSG00000185742	C11orf87
66	12	ENSG00000179088	C12orf42
67	12	ENSG00000130921	C12orf65
	12	ENSG00000257242	
	12	ENSG00000279037	

68	12	ENSG00000280112	C12orf79
69	16	ENSG00000159761	C16orf86
70	16	ENSG00000167194	C16orf92
71	1	ENSG00000203709	C1orf132
72	1	ENSG00000118292	C1orf54
73	2	ENSG00000162972	C2orf47
74	2	ENSG00000178074	C2orf69
75	2	ENSG00000182600	C2orf82
76	3	ENSG00000163632	C3orf49
77	4	ENSG00000056050	C4orf27
78	1	ENSG00000118298	CA14
79	8	ENSG00000178538	CA8
80	12	ENSG00000151067	CACNA1C
81	22	ENSG00000100346	CACNA1I
82	10	ENSG00000165995	CACNB2
83	17	ENSG00000075429	CACNG5
84	10	ENSG00000185933	CALHM1
85	10	ENSG00000138172	CALHM2
86	10	ENSG00000183128	CALHM3
87	7	ENSG00000183166	CALN1
88	17	ENSG00000260785	CASC17
89	3	ENSG00000145075	CCDC39
90	12	ENSG00000183273	CCDC60
91	18	ENSG00000166510	CCDC68
92	5	ENSG00000170458	CD14
93	1	ENSG00000117335	CD46
94	5	ENSG00000158402	CDC25C
95	16	ENSG00000140945	CDH13
96	12	ENSG00000111328	CDK2AP1
97	14	ENSG00000100526	CDKN3
98	19	ENSG00000007129	CEACAM21
99	2	ENSG00000115816	CEBPZ
100	22	ENSG00000100162	CENPM
101	16	ENSG00000102901	CENPT
102	1	ENSG00000143702	CEP170
103	2	ENSG00000188452	CERKL
104	22	ENSG00000100399	CHADL
105	1	ENSG00000133048	CHI3L1
106	11	ENSG00000180720	CHRM4
107	15	ENSG00000080644	CHRNA3
108	15	ENSG00000169684	CHRNA5
109	15	ENSG00000117971	CHRNB4
110	19	ENSG00000124302	CHST8
111	19	ENSG00000160161	CILP2
112	11	ENSG00000175216	CKAP5
113	14	ENSG00000166165	CKB
114	19	ENSG00000105205	CLC
115	4	ENSG00000109572	CLCN3

116	3	ENSG00000163347	CLDN1
117	11	ENSG00000172409	CLP1
118	9	ENSG00000122705	CLTA
119	8	ENSG00000120885	CLU
120	12	ENSG00000174600	CMKLR1
121	X	ENSG00000149970	CNKSR2
122	10	ENSG00000148842	CNNM2
123	16	ENSG00000125107	CNOT1
124	3	ENSG00000144619	CNTN4
125	11	ENSG00000149972	CNTN5
126	7	ENSG00000174469	CNTNAP2
127	2	ENSG00000155052	CNTNAP5
128	4	ENSG00000188517	COL25A1
129	3	ENSG00000144810	COL8A1
130	5	ENSG00000145781	COMMD10
131	22	ENSG00000093010	COMT
132	2	ENSG00000115520	COQ10B
133	6	ENSG00000231162	COX11P1
134	1	ENSG00000197721	CR1L
135	11	ENSG00000157613	CREB3L1
136	X	ENSG00000198223	CSF2RA
137	8	ENSG00000183117	CSMD1
138	15	ENSG00000259726	CSPG4P11
139	16	ENSG00000102974	CTCF
140	18	ENSG00000060069	CTDP1
141	5	ENSG00000044115	CTNNA1
142	2	ENSG00000066032	CTNNA2
143	10	ENSG00000183230	CTNNA3
144	11	ENSG00000198561	CTNND1
145	16	ENSG00000141086	CTRL
146	5	ENSG00000205279	CTXN3
147	2	ENSG00000036257	CUL3
148	10	ENSG00000107562	CXCL12
149	17	ENSG00000167740	CYB5D2
150	X	ENSG00000233548	CYCSP44
151	10	ENSG00000148795	CYP17A1
152	2	ENSG00000003137	CYP26B1
153	22	ENSG00000100197	CYP2D6
154	22	ENSG00000226450	CYP2D8P
155	12	ENSG00000110887	DAO
156	13	ENSG00000182346	DAOA
157	1	ENSG00000143164	DCAF6
158	12	ENSG00000151065	DCP1B
159	7	ENSG00000132437	DDC
160	9	ENSG00000230360	DDX10P2
161	16	ENSG00000182810	DDX28
162	7	ENSG00000105928	DFNA5
163	7	ENSG00000157680	DGKI

164	11	ENSG00000149091	DGKZ
165	1	ENSG00000162946	DISC1
166	1	ENSG00000274121	DISC2
167	8	ENSG00000164741	DLC1
168	3	ENSG00000114841	DNAH1
169	3	ENSG00000205981	DNAJC19
170	5	ENSG00000256453	DND1
171	16	ENSG00000149927	DOC2A
172	16	ENSG00000167261	DPEP2
173	16	ENSG00000141096	DPEP3
174	2	ENSG00000197635	DPP4
175	1	ENSG00000188641	DPYD
176	1	ENSG00000232878	DPYD-AS1
177	1	ENSG00000235777	DPYD-AS2
178	1	ENSG00000232542	DPYD-IT1
179	11	ENSG00000149295	DRD2
180	3	ENSG00000151577	DRD3
181	17	ENSG00000108591	DRG2
182	16	ENSG00000038358	EDC4
183	2	ENSG00000115468	EFHD1
184	15	ENSG00000259404	EFTUD1P1
185	5	ENSG00000120738	EGR1
186	11	ENSG00000135373	EHF
187	2	ENSG00000233426	EIF3FP3
188	1	ENSG00000075151	EIF4G3
189	1	ENSG00000162618	ELTD1
190	16	ENSG00000124074	ENKD1
191	1	ENSG00000074800	ENO1
192	22	ENSG00000100393	EP300
193	2	ENSG00000135999	EPC2
194	8	ENSG00000120915	EPHX2
195	3	ENSG00000187672	ERC2
196	16	ENSG00000175595	ERCC4
197	11	ENSG00000149564	ESAM
198	16	ENSG00000103067	ESRP2
199	5	ENSG00000120705	ETF1
200	6	ENSG00000112685	EXOC2
201	11	ENSG00000180210	F2
202	1	ENSG00000226766	FABP7P1
203	22	ENSG00000177096	FAM109B
204	8	ENSG00000169122	FAM110B
205	13	ENSG00000204442	FAM155A
206	5	ENSG00000120709	FAM53C
207	16	ENSG00000149926	FAM57B
208	1	ENSG00000154511	FAM69A
209	2	ENSG00000115392	FANCL
210	2	ENSG00000116120	FARSB
211	3	ENSG00000237806	FAUP2

212	2	ENSG00000138081	FBXO11
213	1	ENSG00000143226	FCGR2A
214	1	ENSG00000203747	FCGR3A
215	15	ENSG00000182511	FES
216	11	ENSG00000149557	FEZ1
217	1	ENSG00000172456	FGGY
218	2	ENSG00000182263	FIGN
219	3	ENSG00000239516	FLYWCH1P1
220	7	ENSG00000122687	FTSJ2
221	15	ENSG00000140564	FURIN
222	6	ENSG00000172461	FUT9
223	9	ENSG00000165060	FXN
224	3	ENSG00000114416	FXR1
225	6	ENSG00000204681	GABBR1
226	6	ENSG00000146276	GABRR1
227	5	ENSG00000164574	GALNT10
228	19	ENSG00000167491	GATAD2A
229	4	ENSG00000227725	GCOM2
230	16	ENSG00000102886	GDPD3
231	16	ENSG00000141098	GFOD2
232	5	ENSG00000146013	GFRA3
233	17	ENSG00000141034	GID4
234	2	ENSG00000204120	GIGYF2
235	1	ENSG00000187513	GJA4
236	9	ENSG00000122694	GLIPR2
237	12	ENSG00000151948	GLT1D1
238	3	ENSG00000016864	GLT8D1
239	3	ENSG00000168237	GLYCTK
240	19	ENSG00000089639	GMIP
241	9	ENSG00000159921	GNE
242	3	ENSG00000163938	GNL3
243	15	ENSG00000184206	GOLGA6L4
244	15	ENSG00000230373	GOLGA6L5P
245	2	ENSG00000063660	GPC1
246	4	ENSG00000150625	GPM6A
247	2	ENSG00000183671	GPR1
248	1	ENSG00000077585	GPR137B
249	6	ENSG00000224586	GPX5
250	6	ENSG00000198704	GPX6
251	11	ENSG00000023171	GRAMD1B
252	7	ENSG00000106070	GRB10
253	5	ENSG00000155511	GRIA1
254	21	ENSG00000171189	GRIK1
255	1	ENSG00000163873	GRIK3
256	16	ENSG00000183454	GRIN2A
257	7	ENSG00000198822	GRM3
258	2	ENSG00000121964	GTDC1
259	8	ENSG00000234770	GULOP

260	19	ENSG00000187664	HAPLN4
261	11	ENSG00000180423	HARBI1
262	5	ENSG00000170445	HARS
263	5	ENSG00000112855	HARS2
264	5	ENSG00000164588	HCN1
265	2	ENSG00000068024	HDAC4
266	1	ENSG00000054392	HHAT
267	16	ENSG00000149929	HIRIP3
268	6	ENSG00000196787	HIST1H2AG
269	6	ENSG00000124635	HIST1H2BJ
270	6	ENSG00000185130	HIST1H2BL
271	6	ENSG00000196735	HLA-DQA1
272	6	ENSG00000196126	HLA-DRB1
273	6	ENSG00000196301	HLA-DRB9
274	3	ENSG00000244101	HMGN1P10
275	17	ENSG00000262333	HNRNPA1P16
276	6	ENSG00000249853	HS3ST5
277	1	ENSG00000173110	HSPA6
278	5	ENSG00000113013	HSPA9
279	2	ENSG00000144381	HSPD1
280	2	ENSG00000115541	HSPE1
281	2	ENSG00000270757	HSPE1-MOB4
282	13	ENSG00000102468	HTR2A
283	21	ENSG00000142149	HUNK
284	1	ENSG00000137965	IFI44
285	1	ENSG00000137959	IFI44L
286	9	ENSG00000096872	IFT74
287	13	ENSG00000032742	IFT88
288	11	ENSG00000080854	IGSF9B
289	5	ENSG00000113141	IK
290	2	ENSG00000115008	IL1A
291	2	ENSG00000115590	IL1R2
292	X	ENSG00000185291	IL3RA
293	7	ENSG00000184903	IMMP2L
294	10	ENSG00000148798	INA
295	16	ENSG00000169592	INO80E
296	2	ENSG00000168918	INPP5D
297	7	ENSG00000186480	INSIG1
298	15	ENSG00000136381	IREB2
299	1	ENSG00000168264	IRF2BP2
300	3	ENSG00000055957	ITIH1
301	3	ENSG00000162267	ITIH3
302	3	ENSG00000055955	ITIH4
303	3	ENSG00000239799	ITIH4-AS1
304	2	ENSG00000198399	ITSN2
305	11	ENSG00000183340	JRKL
306	16	ENSG00000262962	KARSP3
307	20	ENSG00000158445	KCNB1

308	2	ENSG00000115474	KCNJ13
309	17	ENSG00000123700	KCNJ2
	3	ENSG00000197584	
310	3	ENSG00000275163	KCNMB2
311	8	ENSG00000164794	KCNV1
312	16	ENSG00000174943	KCTD13
313	5	ENSG00000120733	KDM3B
314	1	ENSG00000066135	KDM4A
315	1	ENSG00000162849	KIF26B
316	2	ENSG00000168280	KIF5C
317	14	ENSG00000126214	KLC1
318	15	ENSG00000183655	KLHL25
319	7	ENSG00000239569	KMT2E-AS1
320	22	ENSG00000100395	L3MBTL2
321	16	ENSG00000213398	LCAT
322	19	ENSG00000226025	LGALS17A
323	15	ENSG00000259527	LINC00052
324	6	ENSG00000235570	LINC00533
325	22	ENSG00000205704	LINC00634
326	15	ENSG00000259728	LINC00933
327	7	ENSG00000228393	LINC01004
328	2	ENSG00000205054	LINC01121
329	19	ENSG00000064547	LPAR2
330	4	ENSG00000150471	LPHN3
331	12	ENSG00000123384	LRP1
332	17	ENSG00000171962	LRRC48
333	2	ENSG00000124831	LRRFIP1
334	1	ENSG00000162620	LRRIQ3
335	8	ENSG00000175324	LSM1
336	11	ENSG00000187398	LUZP2
337	2	ENSG00000150556	LYPD6B
338	3	ENSG00000157093	LYZL4
339	7	ENSG00000002822	MAD1L1
340	5	ENSG00000112893	MAN2A1
341	15	ENSG00000196547	MAN2A2
342	14	ENSG00000006432	MAP3K9
343	2	ENSG00000071054	MAP4K4
344	16	ENSG00000102882	MAPK3
345	2	ENSG00000247626	MARS2
346	19	ENSG00000129933	MAU2
347	5	ENSG00000171444	MCC
348	11	ENSG00000110492	MDK
349	X	ENSG00000169057	MECP2
350	11	ENSG00000156603	MED19
351	5	ENSG00000081189	MEF2C
352	5	ENSG00000248309	MEF2C-AS1
353	8	ENSG00000275264	MIR1204
354	12	ENSG00000221365	MIR1228

355	22	ENSG00000221160	MIR1281
356	10	ENSG00000221767	MIR1307
357	11	ENSG00000208009	MIR130A
358	3	ENSG00000207926	MIR135A1
359	1	ENSG00000277990	MIR137
360	1	ENSG00000225206	MIR137HG
361	22	ENSG00000207932	MIR33A
362	22	ENSG00000263463	MIR378I
363	8	ENSG00000199127	MIR383
364	12	ENSG00000265526	MIR4304
365	18	ENSG00000264571	MIR4529
366	1	ENSG00000265201	MIR4677
367	X	ENSG00000221466	MIR548AJ2
368	19	ENSG00000207821	MIR640
369	3	ENSG00000199150	MIRLET7G
370	22	ENSG00000196588	MKL1
371	8	ENSG00000156103	MMP16
372	2	ENSG00000124003	MOGAT1
373	12	ENSG00000051825	MPHOSPH9
374	7	ENSG00000105926	MPP6
375	11	ENSG00000120458	MSANTD2
376	3	ENSG00000174579	MSL2
377	8	ENSG00000175806	MSRA
378	1	ENSG00000177000	MTHFR
379	3	ENSG00000272573	MUSTN1
380	17	ENSG00000091536	MYO15A
381	13	ENSG00000041515	MYO16
382	22	ENSG00000133454	MYO18B
383	12	ENSG00000166866	MYO1A
384	12	ENSG00000166886	NAB2
385	22	ENSG00000198951	NAGA
386	13	ENSG00000102452	NALCN
387	19	ENSG00000130287	NCAN
388	3	ENSG00000158092	NCK1
389	4	ENSG00000164100	NDST3
390	19	ENSG00000186010	NDUFA13
391	5	ENSG00000131495	NDUFA2
392	12	ENSG00000185633	NDUFA4L2
393	22	ENSG00000184983	NDUFA6
394	4	ENSG00000137601	NEK1
395	3	ENSG00000114904	NEK4
396	2	ENSG00000115488	NEU2
397	16	ENSG00000072736	NFATC3
398	7	ENSG00000050344	NFE2L3
399	9	ENSG00000165030	NFIL3
400	4	ENSG00000109320	NFKB1
401	1	ENSG00000233557	NFU1P2
402	2	ENSG00000066248	NGEF

403	3	ENSG00000010322	NISCH
404	6	ENSG00000188580	NKAIN2
405	6	ENSG00000189134	NKAPL
406	X	ENSG00000146938	NLGN4X
407	16	ENSG00000140853	NLRC5
408	5	ENSG00000132911	NMUR2
409	1	ENSG00000198929	NOS1AP
410	19	ENSG00000142546	NOSIP
411	6	ENSG00000204301	NOTCH4
412	14	ENSG00000151322	NPAS3
413	2	ENSG00000213104	NPM1P46
414	8	ENSG00000157168	NRG1
415	10	ENSG00000185737	NRG3
416	11	ENSG00000154146	NRGN
417	16	ENSG00000188038	NRN1L
418	10	ENSG00000099250	NRP1
419	10	ENSG00000241058	NSUN6
420	10	ENSG00000076685	NT5C2
421	3	ENSG00000168268	NT5DC2
422	15	ENSG00000140538	NTRK3
423	14	ENSG00000151413	NUBPL
424	7	ENSG00000106268	NUDT1
425	16	ENSG00000102898	NUTF2
426	12	ENSG00000182379	NXPH4
427	12	ENSG00000111325	OGFOD2
428	22	ENSG00000213790	OLA1P1
429	12	ENSG00000179919	OR10A7
430	12	ENSG00000175398	OR10P1
431	6	ENSG00000219262	OR2E1P
432	11	ENSG00000255303	OR5BA1P
433	12	ENSG00000205330	OR6C1
434	12	ENSG00000179695	OR6C2
435	12	ENSG00000205329	OR6C3
436	12	ENSG00000179626	OR6C4
437	12	ENSG00000188324	OR6C6
438	12	ENSG00000205328	OR6C65
439	12	ENSG00000205327	OR6C68
440	12	ENSG00000184954	OR6C70
441	12	ENSG00000197706	OR6C74
442	12	ENSG00000187857	OR6C75
443	12	ENSG00000185821	OR6C76
444	12	ENSG00000170605	OR9K2
445	7	ENSG00000070882	OSBPL3
446	1	ENSG00000264522	OTUD7B
	15	ENSG00000137843	
447	15	ENSG00000259288	PAK6
448	4	ENSG00000129116	PALLD
449	10	ENSG00000148498	PARD3

450	16	ENSG00000102981	PARD6A
451	3	ENSG00000163939	PBRM1
452	19	ENSG00000105717	PBX4
453	4	ENSG00000251321	PCAT4
454	3	ENSG00000114054	PCCB
	13	ENSG00000197991	
455	13	ENSG00000280165	PCDH20
456	5	ENSG00000204970	PCDHA1
457	5	ENSG00000250120	PCDHA10
458	5	ENSG00000204969	PCDHA2
459	5	ENSG00000255408	PCDHA3
460	5	ENSG00000204967	PCDHA4
461	5	ENSG00000204965	PCDHA5
462	5	ENSG00000081842	PCDHA6
463	5	ENSG00000204963	PCDHA7
464	5	ENSG00000204962	PCDHA8
465	5	ENSG00000204961	PCDHA9
	2	ENSG00000227418	
466	2	ENSG00000278406	PCGEM1
467	10	ENSG00000156374	PCGF6
468	10	ENSG00000148843	PDCD11
469	5	ENSG00000113448	PDE4D
470	1	ENSG00000174827	PDZK1
471	6	ENSG00000137338	PGBD1
472	3	ENSG00000010318	PHF7
473	X	ENSG00000172943	PHF8
474	2	ENSG00000153823	PID1
475	11	ENSG00000011405	PIK3C2A
476	12	ENSG00000139144	PIK3C2G
477	12	ENSG00000090975	PITPNM2
478	X	ENSG00000181191	PJA1
479	11	ENSG00000165495	PKNOX2
480	16	ENSG00000103066	PLA2G15
481	9	ENSG00000137055	PLAA
482	15	ENSG00000137841	PLCB2
483	15	ENSG00000259307	PLCB2-AS1
484	1	ENSG00000149527	PLCH2
485	2	ENSG00000115896	PLCL1
486	1	ENSG00000023902	PLEKHO1
487	7	ENSG00000128567	PODXL
488	15	ENSG00000255529	POLR2M
489	6	ENSG00000158553	POM121L2
490	6	ENSG00000184486	POU3F2
491	4	ENSG00000138777	PPA2
492	12	ENSG00000139220	PPFIA2
493	3	ENSG00000164088	PPM1M
494	14	ENSG00000088808	PPP1R13B
495	20	ENSG00000101445	PPP1R16B

496	5	ENSG00000156475	PPP2R2B
497	3	ENSG00000073711	PPP2R3A
498	16	ENSG00000149923	PPP4C
499	5	ENSG00000132356	PRKAA1
500	1	ENSG00000067606	PRKCZ
501	14	ENSG00000184304	PRKD1
502	2	ENSG00000115825	PRKD3
503	22	ENSG00000100033	PRODH
504	19	ENSG00000126464	PRR12
505	6	ENSG00000204469	PRRC2A
506	19	ENSG00000126460	PRRG2
507	6	ENSG00000112812	PRSS16
508	16	ENSG00000159792	PSKH1
509	15	ENSG00000041357	PSMA4
510	16	ENSG00000205220	PSMB10
511	3	ENSG00000163636	PSMD6
512	1	ENSG00000117569	PTBP2
513	20	ENSG00000124212	PTGIS
514	7	ENSG00000105894	PTN
515	1	ENSG00000142949	PTPRF
516	7	ENSG00000155093	PTPRN2
517	12	ENSG00000151490	PTPRO
518	1	ENSG00000060656	PTPRU
519	7	ENSG00000091127	PUS7
520	8	ENSG00000249859	PVT1
521	2	ENSG00000115828	QPCT
522	12	ENSG00000179912	R3HDM2
523	4	ENSG00000250040	RAF1P1
524	17	ENSG00000108557	RAI1
525	16	ENSG00000141084	RANBP10
526	22	ENSG00000100401	RANGAP1
527	5	ENSG00000113319	RASGRF2
528	19	ENSG00000142552	RCN3
529	9	ENSG00000122707	RECK
530	5	ENSG00000132563	REEP2
531	2	ENSG00000115386	REG1A
532	2	ENSG00000172023	REG1B
533	2	ENSG00000143954	REG3G
534	7	ENSG00000189056	RELN
535	X	ENSG00000102032	RENBP
536	1	ENSG00000142599	RERE
537	3	ENSG00000163933	RFT1
538	2	ENSG00000162944	RFTN2
539	14	ENSG00000182732	RGS6
540	12	ENSG00000150977	RILPL2
541	6	ENSG00000079841	RIMS1
542	20	ENSG00000132669	RIN2
543	16	ENSG00000159753	RLTPR

544	9	ENSG00000137075	RNF38
545	3	ENSG00000169855	ROBO1
546	3	ENSG00000185008	ROBO2
547	9	ENSG00000169071	ROR2
548	15	ENSG00000069667	RORA
549	1	ENSG00000226128	RPL26P9
550	16	ENSG00000240435	RPS12P27
551	19	ENSG00000126458	RRAS
552	22	ENSG00000040608	RTN4R
553	7	ENSG00000244490	RWDD4P1
554	9	ENSG00000186350	RXRA
555	2	ENSG00000119042	SATB2
556	12	ENSG00000139697	SBNO1
557	19	ENSG00000126461	SCAF1
558	2	ENSG00000115884	SDC1
559	1	ENSG00000054282	SDCCAG8
560	1	ENSG00000120341	SEC16B
561	1	ENSG00000228057	SEC63P1
562	7	ENSG00000075213	SEMA3A
563	3	ENSG00000010319	SEMA3G
564	22	ENSG00000100167	Sep-03
565	11	ENSG00000149131	SERPING1
566	12	ENSG00000183955	SETD8
567	16	ENSG00000174938	SEZ6L2
568	2	ENSG00000115524	SF3B1
569	3	ENSG00000163935	SFMBT1
570	10	ENSG00000156398	SFXN2
571	8	ENSG00000185053	SGCZ
572	6	ENSG00000118515	SGK1
573	2	ENSG00000163082	SGPP2
574	17	ENSG00000141258	SGSM2
575	22	ENSG00000251322	SHANK3
576	22	ENSG00000234965	SHISA8
577	12	ENSG00000182199	SHMT2
578	1	ENSG00000117090	SLAMF1
579	5	ENSG00000064651	SLC12A2
580	16	ENSG00000124067	SLC12A4
581	6	ENSG00000124568	SLC17A1
582	6	ENSG00000124564	SLC17A3
583	11	ENSG00000091664	SLC17A6
584	9	ENSG00000106688	SLC1A1
585	6	ENSG00000137266	SLC22A23
586	22	ENSG00000215347	SLC25A5P1
587	1	ENSG00000174502	SLC26A9
588	6	ENSG00000146411	SLC2A12
589	20	ENSG00000101438	SLC32A1
590	11	ENSG00000110660	SLC35F2
591	3	ENSG00000168917	SLC35G2

592	16	ENSG00000103042	SLC38A7
593	4	ENSG00000138821	SLC39A8
594	1	ENSG00000162426	SLC45A1
595	2	ENSG00000144290	SLC4A10
596	16	ENSG00000103064	SLC7A6
597	16	ENSG00000103061	SLC7A6OS
598	15	ENSG00000176463	SLCO3A1
599	5	ENSG00000205359	SLCO6A1
600	9	ENSG00000080503	SMARCA2
601	22	ENSG00000183172	SMDT1
602	16	ENSG00000157106	SMG1
603	17	ENSG00000070366	SMG6
604	3	ENSG00000168273	SMIM4
605	6	ENSG00000065609	SNAP91
606	8	ENSG00000147481	SNTG1
607	11	ENSG00000120451	SNX19
608	7	ENSG00000106266	SNX8
609	3	ENSG00000242808	SOX2-OT
610	2	ENSG00000144451	SPAG16
611	2	ENSG00000196141	SPATS2L
612	3	ENSG00000114902	SPCS1
613	2	ENSG00000144228	SPOPL
614	9	ENSG00000090054	SPTLC1
615	2	ENSG00000068784	SRBD1
616	17	ENSG00000072310	SREBF1
617	22	ENSG00000198911	SREBF2
618	7	ENSG00000135250	SRPK2
619	17	ENSG00000167720	SRR
620	10	ENSG00000229256	ST13P13
621	8	ENSG00000008513	ST3GAL1
622	3	ENSG00000010327	STAB1
623	12	ENSG00000185482	STAC3
624	3	ENSG00000118007	STAG1
625	12	ENSG00000166888	STAT6
626	4	ENSG00000109689	STIM2
627	11	ENSG00000134910	STT3A
628	19	ENSG00000105705	SUGP1
629	2	ENSG00000138068	SULT6B1
630	3	ENSG00000157152	SYN2
631	6	ENSG00000131018	SYNE1
632	12	ENSG00000166863	TAC3
633	10	ENSG00000148835	TAF5
634	16	ENSG00000149930	TAOK2
635	3	ENSG00000131374	TBC1D5
636	16	ENSG00000149922	TBX6
637	7	ENSG00000059377	TBXAS1
638	22	ENSG00000100207	TCF20
639	18	ENSG00000196628	TCF4

640	3	ENSG00000241438	TDGF1P6
641	2	ENSG00000163235	TGFA
642	16	ENSG00000168286	THAP11
643	3	ENSG00000163634	THOC7
644	3	ENSG00000240549	THOC7-AS1
645	15	ENSG00000104067	TJP1
646	9	ENSG00000196781	TLE1
647	15	ENSG00000140332	TLE3
648	3	ENSG00000239732	TLR9
649	19	ENSG00000213996	TM6SF2
650	5	ENSG00000113119	TMCO6
651	3	ENSG00000213533	TMEM110
652	3	ENSG00000248592	MEM110-MUSTN1
653	12	ENSG00000151952	TMEM132D
654	12	ENSG00000166881	TMEM194A
655	3	ENSG00000186329	TMEM212
656	16	ENSG00000149932	TMEM219
657	12	ENSG00000133687	TMTC1
658	11	ENSG00000213593	TMX2
659	18	ENSG00000141655	TNFRSF11A
660	22	ENSG00000159958	TNFRSF13C
661	3	ENSG00000154310	TNIK
662	3	ENSG00000114854	TNNC1
663	17	ENSG00000175662	TOM1L2
664	14	ENSG00000131323	TRAF3
665	3	ENSG00000168016	TRANK1
666	6	ENSG00000234127	TRIM26
667	11	ENSG00000166326	TRIM44
668	10	ENSG00000171206	TRIM8
669	14	ENSG00000166166	TRMT61A
670	8	ENSG00000171045	TSNARE1
671	16	ENSG00000102904	TSNAXIP1
672	11	ENSG00000157570	TSPAN18
673	17	ENSG00000167721	TSR1
674	19	ENSG00000178093	TSSK6
675	5	ENSG00000113638	TTC33
676	9	ENSG00000175764	TTLL11
677	8	ENSG00000104723	TUSC3
678	3	ENSG00000247596	TWF2
679	2	ENSG00000162971	TYW5
680	15	ENSG00000189136	UBE2Q2P1
681	10	ENSG00000173915	USMG5
682	13	ENSG00000152484	USP12
683	1	ENSG00000136631	VPS45
684	2	ENSG00000028116	VRK2
685	11	ENSG00000019102	VSIG2
686	10	ENSG00000166272	WBP1L
687	22	ENSG00000183066	WBP2NL

688	4	ENSG00000071127	WDR1
689	5	ENSG00000120314	WDR55
690	15	ENSG00000166415	WDR72
691	3	ENSG00000164091	WDR82
692	8	ENSG00000147548	WHSC1L1
693	16	ENSG00000186153	WWOX
694	14	ENSG00000126215	XRCC3
695	19	ENSG00000250067	YJEFN3
696	16	ENSG00000090238	YPEL3
697	11	ENSG00000166793	YPEL4
698	3	ENSG00000181722	ZBTB20
699	2	ENSG00000204186	ZDBF2
700	11	ENSG00000156599	ZDHHC5
701	2	ENSG00000169554	ZEB2
702	16	ENSG00000184939	ZFP90
703	8	ENSG00000169946	ZFPM2
704	14	ENSG00000100711	ZFYVE21
705	4	ENSG00000159733	ZFYVE28
706	6	ENSG00000189298	ZKSCAN3
707	6	ENSG00000187626	ZKSCAN4
708	5	ENSG00000146007	ZMAT2
709	10	ENSG00000108175	ZMIZ1
710	1	ENSG00000196418	ZNF124
711	6	ENSG00000096654	ZNF184
712	11	ENSG00000166261	ZNF202
713	11	ENSG00000175213	ZNF408
714	19	ENSG00000198597	ZNF536
715	8	ENSG00000120963	ZNF706
716	2	ENSG00000170396	ZNF804A
717	20	ENSG00000124203	ZNF831
718	6	ENSG00000158691	ZSCAN12
719	15	ENSG00000176371	ZSCAN2
720	6	ENSG00000187987	ZSCAN23
721	6	ENSG00000235109	ZSCAN31
722	5	ENSG00000130449	ZSWIM6
723	17	ENSG00000074755	ZZEF1
