# Supplementary material for: THC exposure of human iPSC neurons impacts genes associated with neuropsychiatric disorders
Source: Transl Psychiatry. 2018 Apr 25;8:89. doi: 10.1038/s41398-018-0137-3 (PMC5915454; doi:10.1038/s41398-018-0137-3)
Supplement: Supplementary file 9 — Supplementary Table 8 [file 41398_2018_137_MOESM9_ESM.pdf]

**Supplementary Table 8: Genes associated with mitochondrial and ion channel function altered in schizophrenia and in response to THC treatments**

| Gene class | Acute THC dose | Chronic THC doses | Schizophrenia |
|---|---|---|---|
| | | | |
| | | | |
| **Mitochondrial genes** | | | |
| | | | |
| MT-ATP6 | ✔ | | |
| UQCRBP1 | ✔ | | |
| SDHA | ✔ | | |
| DDIT4 | ✔ | | |
| DDX28 | | ✔ | |
| DNAJA3 | | ✔ | |
| HAX1 | | ✔ | |
| KIAA1683 | | ✔ | |
| TRIAP1 | | ✔ | |
| RARS | | ✔ | |
| CS | | ✔ | |
| CYB5R3 | | ✔ | |
| CYC1 | | ✔ | |
| DIABLO | | ✔ | |
| GBF1 | | ✔ | |
| HADH | | ✔ | |
| MRPL28 | | ✔ | |
| MRPS18B | | ✔ | |
| PTRF | | ✔ | |
| SDHB | | ✔ | |
| UQCRC2 | | ✔ | |
| YRDC | | ✔ | |
| MARS2 | | ✔ | |
| MAVS | | ✔ | |
| SLC25A4 | | ✔ | |
| MRPL17 | | ✔ | |
| MT-ND4 | ✔ | ✔ | |
| MT-CO1 | ✔ | ✔ | |
| MT-CO2 | ✔ | ✔ | |
| MT-CO3 | ✔ | ✔ | |
| MT-ND4L | ✔ | ✔ | |
| ADPRHL2 | ✔ | ✔ | |
| GRPEL1 | ✔ | ✔ | |
| ALDH18A1 | ✔ | ✔ | |
| COQ10B | ✔ | ✔ | |
| COX7A2 | ✔ | ✔ | |
| DMPK | ✔ | ✔ | |
| HAGH | ✔ | ✔ | |

| | | | |
|---|---|---|---|
| MTHFD2 | ✔ | ✔ | |
| MGST1 | ✔ | ✔ | |
| MTFP1 | ✔ | ✔ | |
| MRPL14 | ✔ | ✔ | |
| MRPL49 | ✔ | ✔ | |
| MRPL54 | ✔ | ✔ | |
| MRPS7 | ✔ | ✔ | |
| PMAIP1 | ✔ | ✔ | |
| PDK2 | ✔ | ✔ | |
| RGS2 | ✔ | ✔ | |
| RPS3 | ✔ | ✔ | |
| SGK1 | ✔ | ✔ | |
| SLC25A32 | ✔ | ✔ | |
| TFB2M | ✔ | ✔ | |
| ME3 | | | ✔ |
| | | | |
| **WNT genes** | | | |
| | | | |
| APC | ✔ | ✔ | |
| DDIT3 | ✔ | ✔ | |
| HIC1 | ✔ | ✔ | |
| BTRC | ✔ | ✔ | |
| DDB1 | ✔ | ✔ | |
| DKK3 | ✔ | ✔ | |
| SOX5 | ✔ | ✔ | |
| SOX12 | ✔ | ✔ | |
| UBC | ✔ | ✔ | |
| ZBED3 | ✔ | | |
| PTPRZ1 | ✔ | | |
| WNT16 | | ✔ | |
| FZD2 | | ✔ | |
| GSK3B | | ✔ | |
| TNKS | | ✔ | |
| CSNK2A2 | | ✔ | |
| BCL9 | | ✔ | |
| EP300 | | ✔ | |
| DKK1 | | ✔ | |
| FZD1 | | ✔ | |
| CDH10 | | ✔ | |
| TSC1 | | ✔ | |
| TCF4 | | | ✔ |
| ACVR1 | | | ✔ |
| DAB2 | | | ✔ |
| NOTUM | | | ✔ |
| RSPO3 | | | ✔ |
| TRABD2B | | | ✔ |

| | | | |
|---|---|---|---|
| WIF1 | | | ✔ |
| WNT10A | | | ✔ |
| WNT5A | | | ✔ |
| WNT6 | | | ✔ |
| KREMEN1 | | | ✔ |
| ROR1 | | | ✔ |
| RNF43 | | | ✔ |
| SOSTDC1 | | | ✔ |
| TLE2 | | | ✔ |
| WLS | | | ✔ |
| | | | |
| **Ion channel genes** | | | |
| | | | |
| CACNG7 | ✔ | ✔ | |
| GRID2 | ✔ | ✔ | |
| GRIK1 | ✔ | ✔ | |
| ITPR2 | ✔ | ✔ | |
| LRRC8E | ✔ | ✔ | |
| KCNN3 | ✔ | ✔ | |
| KCNK15 | ✔ | | |
| KCNF1 | ✔ | | |
| KCNA4 | ✔ | ✔ | |
| KCNE4 | ✔ | ✔ | |
| KCNJ10 | ✔ | ✔ | |
| RYR3 | ✔ | ✔ | |
| TRPV1 | ✔ | ✔ | |
| TMEM38A | ✔ | | |
| FXYD6 | | ✔ | |
| ASIC1 | | ✔ | |
| CHRNA9 | | ✔ | |
| KCNT2 | | ✔ | |
| KCNK6 | | ✔ | |
| KCNJ2 | | ✔ | |
| KCNQ1 | | ✔ | |
| TMEM63A | | ✔ | |
| ATP1A2 | | | ✔ |
| BEST1 | | | ✔ |
| CALHM2 | | | ✔ |
| CACNA2D1 | | | ✔ |
| CACNG6 | | | ✔ |
| CACNA1G | | | ✔ |
| CLCA2 | | | ✔ |
| CLIC2 | | | ✔ |
| CHRNA1 | | | ✔ |
| CLDN10 | | | ✔ |
| GABRE | | | ✔ |

| | | | |
|---|---|---|---|
| GABRQ | | | ✔ |
| GRIK2 | | | ✔ |
| HFE | | | ✔ |
| PKD1L3 | | | ✔ |
| KCNMB2 | | | ✔ |
| KCNU1 | | | ✔ |
| KCNA1 | | | ✔ |
| KCNA5 | | | ✔ |
| KCNA6 | | | ✔ |
| KCNAB1 | | | ✔ |
| KCNE3 | | | ✔ |
| KCNH2 | | | ✔ |
| P2RX7 | | | ✔ |
| SFXN3 | | | ✔ |
| SCN2B | | | ✔ |
| TRPV4 | | | ✔ |
| TMC1 | | | ✔ |
| CNTN2 | | | ✔ |
