# Supplementary material for: THC exposure of human iPSC neurons impacts genes associated with neuropsychiatric disorders
Source: Transl Psychiatry. 2018 Apr 25;8:89. doi: 10.1038/s41398-018-0137-3 (PMC5915454; doi:10.1038/s41398-018-0137-3)
Supplement: Supplementary file 10 — Supplementary Table 9 [file 41398_2018_137_MOESM10_ESM.pdf]

Supplementary Table 9: Significant gene expression changes in response to activation following THC treatments

Significantly altered genes following KCl activation between control and acute THC treated iPSC-derived neurons.

Ensembl gene ID	logFC	logCPM	PValue	FDR	hgnc_symbol	Chromosome	start position	end position	strand
ENSG00000068028	1.266426053	4.322005324	7.18E-07	0.001873926	RASSF1	3	50329782	50340980	-1
ENSG00000070495	1.230446853	5.051611894	1.13E-05	0.011656817	JMJD6	17	76712832	76726799	-1
ENSG00000088451	-1.576374496	3.397171643	5.39E-07	0.001664573	TGDS	13	94574051	94596257	-1
ENSG00000100811	0.757612831	6.896510317	1.18E-05	0.011810869	YY1	14	100238298	100282792	1
ENSG00000106003	-2.12007621	3.841000412	1.64E-05	0.014630961	LFNG	7	2512529	2529177	1
ENSG00000116954	1.265460559	5.545589586	3.53E-07	0.001437724	RRAGC	1	38838198	38859823	-1
ENSG00000134294	2.034366779	9.658319329	3.30E-11	3.74E-07	SLC38A2	12	46358189	46372867	-1
ENSG00000141040	-1.552164988	4.437734035	7.88E-12	2.67E-07	ZNF287	17	16551387	16569206	-1
ENSG00000144120	-1.758453284	2.427672294	7.63E-06	0.009248032	TMEM177	2	119679167	119686507	1
ENSG00000147789	-1.117755361	4.553614633	3.03E-06	0.004987117	ZNF7	8	144827464	144847509	1
ENSG00000149781	3.004857911	0.150597537	2.85E-05	0.023027734	FERMT3	11	64206678	64223886	1
ENSG00000150347	1.438509735	7.50990393	3.31E-06	0.005100382	ARID5B	10	61901300	62096944	1
ENSG00000150457	1.212985646	5.348942231	9.55E-05	0.04815705	LATS2	13	20973032	21061547	-1
ENSG00000157184	-0.962223513	4.651100643	6.57E-05	0.038958442	CPT2	1	53196429	53214197	1
ENSG00000159388	-1.026180561	5.469524308	4.08E-05	0.029496713	BTG2	1	203305491	203309602	1
ENSG00000162396	-1.900233985	2.656259236	1.14E-09	9.64E-06	PARS2	1	54756898	54764514	-1
ENSG00000162757	-2.43752931	2.801233188	2.51E-11	3.74E-07	C1orf74	1	209779208	209784559	-1
ENSG00000163083	-1.553537957	3.72905897	6.23E-07	0.001763353	INHBB	2	120346143	120351808	1
ENSG00000163914	5.640903863	-1.073776942	4.20E-05	0.029719271	RHO	3	129528640	129535169	1
ENSG00000164296	-1.592705174	3.727747895	1.62E-05	0.014630961	TIGD6	5	149993118	150001167	-1
ENSG00000166189	-1.208977961	4.00329844	2.72E-05	0.022491819	HPS6	10	102065390	102068038	1
ENSG00000166192	-1.359259527	3.160098491	5.68E-05	0.037068748	SENP8	15	72114258	72143688	1
ENSG00000166704	-0.879883255	5.219931354	7.95E-05	0.043242095	ZNF606	19	57977053	58003349	-1
ENSG00000168661	-1.294105324	3.236495627	3.41E-05	0.026915717	ZNF30	19	34926903	34945170	1
NA	-1.480538777	1.755300351	7.43E-05	0.042010884	NA	NA	NA	NA	NA
ENSG00000174796	-1.15859168	3.944248976	3.06E-06	0.004987117	THAP6	4	75513946	75550473	1
ENSG00000177352	-1.532586743	3.608950005	5.12E-06	0.006932223	CCDC71	3	49162535	49166321	-1
ENSG00000179476	-2.325720927	2.165270354	2.65E-09	1.80E-05	C14orf28	14	44897295	44907257	1
ENSG00000180938	-1.801796309	1.548549666	9.65E-05	0.04815705	ZNF572	8	124973298	124979389	1
ENSG00000181264	-1.004758796	4.285380715	4.36E-06	0.006172807	TMEM136	11	120325129	120333682	1
ENSG00000181450	-1.376439312	5.305390636	1.28E-06	0.003114816	ZNF678	1	227563543	227677443	1
ENSG00000182308	2.562410292	0.819925282	6.31E-05	0.038801935	DCAF4L1	4	41981696	41986467	1
ENSG00000182405	-1.190268448	2.909950107	1.71E-05	0.014904794	PGBD4	15	34102073	34108684	1
ENSG00000186496	-1.566936115	3.706722947	3.09E-06	0.004987117	ZNF396	18	35366697	35377337	-1
ENSG00000186812	-0.694397626	5.808362009	7.97E-05	0.043242095	ZNF397	18	35241030	35267133	1
ENSG00000189431	-1.355038535	2.600164441	1.07E-05	0.011328782	RASSF10	11	13009577	13012106	1
ENSG00000196214	-1.016586309	4.728240733	1.94E-06	0.004169471	ZNF766	19	52269571	52296046	1
ENSG00000196659	-1.369328733	4.921732905	9.22E-05	0.047909481	TTC30B	2	177548998	177553014	-1
ENSG00000196981	-1.266304027	4.146493362	8.82E-06	0.009982887	WDR5B	3	122412332	122416051	-1
ENSG00000197128	-0.948854099	5.791986663	2.01E-06	0.004169471	ZNF772	19	57466663	57477570	-1
ENSG00000197841	-0.936491552	5.446267753	3.68E-05	0.027534272	ZNF181	19	34734155	34745378	1
ENSG00000198346	-1.186605448	3.76725239	4.24E-06	0.006172807	ZNF813	19	53467735	53496255	1
NA	-3.498534833	-0.835834304	6.72E-05	0.038958442	NA	NA	NA	NA	NA
ENSG00000215251	1.102659708	5.587243263	3.90E-07	0.001437724	FASTKD5	20	3146519	3159897	-1
NA	2.153336411	1.115519842	9.01E-05	0.047781819	NA	NA	NA	NA	NA
ENSG00000224420	2.674794961	0.948013737	6.71E-06	0.008441816	ADM5	19	49688664	49690575	1
NA	-1.560061702	2.045581534	1.07E-05	0.011328782	NA	NA	NA	NA	NA
NA	4.146609849	-0.005254581	9.32E-05	0.047909481	NA	NA	NA	NA	NA
NA	2.080276507	0.785088558	5.53E-05	0.036833325	NA	NA	NA	NA	NA
NA	-1.203414387	3.226204354	8.74E-06	0.009982887	NA	NA	NA	NA	NA
NA	1.531060786	1.680984283	3.52E-05	0.027135813	NA	NA	NA	NA	NA
NA	0.861210647	4.942773702	6.77E-05	0.038958442	NA	NA	NA	NA	NA
ENSG00000249459	1.417063374	4.07377362	6.40E-05	0.038801935	ZNF286B	17	18658429	18682262	-1
NA	2.860703038	0.356330783	5.31E-06	0.006932223	NA	NA	NA	NA	NA
NA	2.275357849	1.257899129	2.09E-06	0.004169471	NA	NA	NA	NA	NA
ENSG00000257390	3.902738233	1.154331377	4.24E-07	0.001437724		12	55757275	55827546	-1
NA	-1.608641144	2.241042932	1.87E-05	0.015853134	NA	NA	NA	NA	NA
ENSG00000259529	1.68698259	1.884396689	5.86E-05	0.037533347		14	24151218	24167402	1
NA	-3.009662018	0.382394403	2.80E-06	0.004987117	NA	NA	NA	NA	NA
NA	5.520636182	-1.075279098	8.03E-05	0.043242095	NA	NA	NA	NA	NA
NA	2.272069958	1.854028231	3.73E-05	0.027534272	NA	NA	NA	NA	NA
NA	5.453055704	-0.747707641	4.98E-05	0.033794518	NA	NA	NA	NA	NA
ENSG00000269343	-0.977121098	4.102909917	0.00010103	0.049701322	ZNF587B	19	57819741	57846238	1
NA	-1.647086966	2.118481669	6.36E-05	0.038801935	NA	NA	NA	NA	NA
NA	3.079141642	-0.389027524	4.58E-05	0.031722328	NA	NA	NA	NA	NA
ENSG00000274349	-1.778687103	3.51361967	3.69E-07	0.001437724	ZNF658	9	66856426	66932141	1
NA	-2.257863472	1.545505763	1.30E-05	0.01256374	NA	NA	NA	NA	NA
NA	-5.108090507	0.053578598	1.61E-05	0.014630961	NA	NA	NA	NA	NA
NA	2.819370644	0.93238859	7.96E-08	0.000450478	NA	NA	NA	NA	NA

Significantly altered genes following KCl activation between control and chronic THC treated iPSC-derived neurons.

Ensembl gene ID	logFC	logCPM	PValue	FDR	hgnc_symbol	chromosome	start position	end position	strand
ENSG00000068028	1.233191871	4.322005324	7.32E-07	0.001427753	RASSF1	3	50329782	50340980	-1
ENSG00000070495	1.256630991	5.051611894	5.73E-06	0.00486508	JMJD6	17	76712832	76726799	-1
ENSG00000088451	-1.265888954	3.397171643	3.10E-05	0.01668535	TGDS	13	94574051	94596257	-1
ENSG00000099622	-1.107934899	7.95099751	4.77E-06	0.004379423	CIRBP	19	1259384	1274880	1
ENSG00000100811	0.811682913	6.896510317	2.42E-06	0.002930465	YY1	14	100238298	100282792	1
ENSG00000105219	2.881007799	-0.943194879	0.00013782	0.045811488	CNTD2	19	40222208	40226690	-1
ENSG00000105732	1.004168123	3.879608457	0.00013988	0.045811488	ZNF574	19	42068477	42081565	1
ENSG00000106003	-1.946928906	3.841000412	6.28E-05	0.027642777	LFNG	7	2512529	2529177	1
ENSG00000109047	6.142956283	-0.654030716	8.68E-08	0.000368128	RCVRN	17	9896320	9905621	-1
ENSG00000109667	3.675544231	0.152030552	6.35E-05	0.027642777	SLC2A9	4	9771153	10054936	-1
ENSG00000116017	1.680868402	2.015887044	4.02E-05	0.02038494	ARID3A	19	925781	975934	1
ENSG00000116560	0.650014894	8.432821734	0.0001036	0.037889282	SFPQ	1	35176378	35193148	-1
ENSG00000116954	1.248453143	5.545589586	3.75E-07	0.000981898	RRAGC	1	38838198	38859823	-1
ENSG00000119737	1.577849445	0.897779254	7.91E-05	0.031959156	GPR75	2	53852913	53859989	-1
ENSG00000120709	1.097147537	7.327946334	8.03E-06	0.006193004	FAM53C	5	138331935	138349729	1
ENSG00000129749	1.93250197	0.731706661	2.88E-05	0.016013445	CHRNA10	11	3665587	3671384	-1
ENSG00000131732	-0.897761176	4.653012968	8.56E-05	0.033717001	ZCCHC9	5	81301590	81313297	1
ENSG00000134294	1.973344418	9.658319329	1.12E-10	1.26E-06	SLC38A2	12	46358189	46372867	-1
ENSG00000135482	-1.120921593	4.25924289	1.38E-06	0.001983576	ZC3H10	12	56118159	56127514	1
ENSG00000136891	0.70945548	5.575513398	0.00010381	0.037889282	TEX10	9	100302077	100352939	-1
ENSG00000137875	5.618840533	-1.224012181	0.00012025	0.042994791	BCL2L10	15	52109263	52112775	-1
ENSG00000141040	-1.537006495	4.437734035	8.63E-12	1.47E-07	ZNF287	17	16551387	16569206	-1
ENSG00000143622	0.683337869	6.733252396	0.00013925	0.045811488	RIT1	1	155897808	155911404	-1
ENSG00000144120	-2.085145968	2.427672294	1.79E-07	0.000608693	TMEM177	2	119679167	119686507	1
ENSG00000147789	-0.994123902	4.553614633	2.25E-05	0.01318374	ZNF7	8	144827464	144847509	1
ENSG00000149781	2.461687034	0.150597537	1.36E-05	0.008540197	FERMT3	11	64206678	64223886	1
ENSG00000150347	1.209833339	7.50990393	8.24E-05	0.032910677	ARID5B	10	61901300	62096944	1
ENSG00000155868	-0.923296016	4.401139836	4.26E-05	0.020641149	MED7	5	157137412	157159019	-1
ENSG00000157184	-0.922840289	4.651100643	0.00010249	0.037889282	CPT2	1	53196429	53214197	1
ENSG00000159388	-1.091852161	5.469524308	1.09E-05	0.00746772	BTG2	1	203305491	203309602	1
ENSG00000162396	-2.188288315	2.656259236	6.86E-13	2.33E-08	PARS2	1	54756898	54764514	-1
ENSG00000162757	-2.134407598	2.801233188	6.08E-10	5.16E-06	C1orf74	1	209779208	209784559	-1
ENSG00000163083	-1.246252477	3.72905897	5.12E-05	0.023163819	INHBB	2	120346143	120351808	1
ENSG00000164011	-1.923073058	2.679557967	2.76E-07	0.000852263	ZNF691	1	42846573	42852477	1
ENSG00000164296	-1.719276004	3.727747895	3.23E-06	0.003554035	TIGD6	5	149993118	150001167	-1
ENSG00000165355	1.080071082	5.556371951	4.21E-05	0.020641149	FBXO33	14	39397669	39432500	-1
ENSG00000166189	-1.247716575	4.00329844	1.22E-05	0.00795593	HPS6	10	102065390	102068038	1
ENSG00000166192	-1.458183764	3.160098491	9.51E-06	0.007176869	SENP8	15	72114258	72143688	1
ENSG00000166704	-0.882998939	5.219931354	6.52E-05	0.028002141	ZNF606	19	57977053	58003349	-1
ENSG00000170681	1.630238067	2.002359279	2.27E-06	0.002848623	MURC	9	100578079	100587906	1
ENSG00000171806	-1.214253642	3.274128686	3.09E-05	0.01668535	METTL18	1	169792529	169794966	-1
ENSG00000174010	0.871655034	5.73399767	4.37E-05	0.020907447	KLHL15	X	23983720	24027186	-1
ENSG00000175536	-2.329066825	0.394678881	7.15E-05	0.029963722	LIPT2	11	74491712	74493733	-1
NA	-3.419284612	-0.547044513	3.73E-05	0.0194688	NA	NA	NA	NA	NA
ENSG00000177352	-1.349620606	3.608950005	4.58E-05	0.021128314	CCDC71	3	49162535	49166321	-1
ENSG00000177426	-1.069779106	6.758011402	4.61E-05	0.021128314	TGIF1	18	3411608	3459978	1
ENSG00000179476	-1.891497172	2.165270354	3.78E-07	0.000981898	C14orf28	14	44897295	44907257	1
NA	5.521943496	-1.140473034	1.12E-05	0.00746772	NA	NA	NA	NA	NA
ENSG00000180938	-2.582143039	1.548549666	2.42E-08	0.000117271	ZNF572	8	124973298	124979389	1
ENSG00000181264	-1.231594467	4.285380715	1.32E-08	7.48E-05	TMEM136	11	120325129	120333682	1
ENSG00000181450	-1.405194564	5.305390636	7.11E-07	0.001427753	ZNF678	1	227563543	227677443	1
ENSG00000182308	2.340284662	0.819925282	7.56E-05	0.031074963	DCAF4L1	4	41981696	41986467	1
ENSG00000183340	-0.948002859	4.144970474	0.00012033	0.042994791	JRKL	11	96389989	96507574	1
ENSG00000186496	-1.45913099	3.706722947	9.81E-06	0.007193606	ZNF396	18	35366697	35377337	-1
ENSG00000186812	-0.730036647	5.808362009	2.74E-05	0.015522337	ZNF397	18	35241030	35267133	1
ENSG00000189431	-1.336389578	2.600164441	3.95E-06	0.003940279	RASSF10	11	13009577	13012106	1
ENSG00000196214	-0.908628493	4.728240733	1.41E-05	0.008562781	ZNF766	19	52269571	52296046	1
ENSG00000196345	-1.957399605	1.099854716	3.39E-06	0.003601105	ZKSCAN7	3	44555193	44594173	1
ENSG00000196981	-1.240803901	4.146493362	1.04E-05	0.007379498	WDR5B	3	122412332	122416051	-1
ENSG00000197128	-0.994264977	5.791986663	5.46E-07	0.001236262	ZNF772	19	57466663	57477570	-1
ENSG00000197566	-1.097172553	4.374550624	3.25E-06	0.003554035	ZNF624	17	16620737	16653856	-1
ENSG00000197841	-0.978158009	5.446267753	1.38E-05	0.008540197	ZNF181	19	34734155	34745378	1
ENSG00000198346	-0.99121364	3.76725239	7.60E-05	0.031074963	ZNF813	19	53467735	53496255	1
ENSG00000204323	-2.190718559	1.489180655	8.64E-05	0.033717001	SMIM5	17	75633434	75641404	1
NA	-1.746589756	1.074133235	0.00014036	0.045811488	NA	NA	NA	NA	NA
NA	3.888598693	-1.084158077	0.00013428	0.045811488	NA	NA	NA	NA	NA
ENSG00000215251	1.126739343	5.587243263	1.68E-07	0.000608693	FASTKD5	20	3146519	3159897	-1
ENSG00000224420	2.601483353	0.948013737	2.67E-06	0.003130105	ADM5	19	49688664	49690575	1
NA	3.908810116	-0.616880823	4.53E-05	0.021128314	NA	NA	NA	NA	NA
NA	4.433945352	-0.005254581	1.12E-05	0.00746772	NA	NA	NA	NA	NA
NA	6.720008116	-1.29643623	0.00013937	0.045811488	NA	NA	NA	NA	NA
NA	1.5813908	1.680984283	4.99E-06	0.004454039	NA	NA	NA	NA	NA
NA	3.259217729	-0.562554461	4.00E-05	0.02038494	NA	NA	NA	NA	NA
NA	3.30935082	-0.326125752	4.65E-06	0.004379423	NA	NA	NA	NA	NA

NA	5.078829063	-1.583122351	0.00013536	0.045811488	NA	NA	NA	NA	NA
NA	1.054792995	4.942773702	1.07E-06	0.001647578	NA	NA	NA	NA	NA
NA	-3.670303609	0.45057878	2.52E-09	1.71E-05	NA	NA	NA	NA	NA
NA	3.601890705	-0.360076642	9.66E-05	0.036862482	NA	NA	NA	NA	NA
ENSG00000249459	1.589887706	4.07377362	6.58E-06	0.005443729	ZNF286B	17	18658429	18682262	-1
NA	1.859348082	1.494482802	6.98E-05	0.029604562	NA	NA	NA	NA	NA
NA	3.258550009	0.356330783	8.17E-07	0.001449725	NA	NA	NA	NA	NA
NA	2.21061305	1.257899129	1.99E-06	0.002596331	NA	NA	NA	NA	NA
NA	-1.609938645	2.047055402	7.93E-06	0.006193004	NA	NA	NA	NA	NA
NA	-1.640405392	0.992703299	0.00014737	0.047641186	NA	NA	NA	NA	NA
NA	2.882610655	1.484702978	1.76E-05	0.010506036	NA	NA	NA	NA	NA
NA	-2.727751338	0.010954644	4.15E-06	0.00402448	NA	NA	NA	NA	NA
NA	-3.600788877	-0.628777628	5.16E-06	0.004493852	NA	NA	NA	NA	NA
ENSG00000262304	5.893222834	-0.225332348	3.32E-05	0.017587044		17	3585149	3636249	-1
NA	-0.97137291	4.375290641	7.37E-06	0.005958451	NA	NA	NA	NA	NA
NA	4.451405858	0.759247704	3.68E-06	0.003780332	NA	NA	NA	NA	NA
NA	1.251601765	2.983671966	2.34E-05	0.013458816	NA	NA	NA	NA	NA
NA	5.136760213	-1.003392582	0.00012824	0.044878657	NA	NA	NA	NA	NA
ENSG00000267699	-5.881488518	-0.247828384	1.05E-06	0.001647578		18	50968019	51058144	1
NA	-2.938273036	-0.264675156	4.11E-05	0.020524585	NA	NA	NA	NA	NA
NA	2.360238105	-0.198656453	0.00010115	0.037889282	NA	NA	NA	NA	NA
NA	-2.018358781	0.549883903	1.35E-05	0.008540197	NA	NA	NA	NA	NA
NA	-2.080223747	0.967983606	9.96E-06	0.007193606	NA	NA	NA	NA	NA
NA	-1.942635203	1.615046619	1.87E-06	0.002538161	NA	NA	NA	NA	NA
NA	1.913258379	0.989924238	0.00012552	0.044381551	NA	NA	NA	NA	NA
NA	2.290854091	1.108251271	4.05E-07	0.000981898	NA	NA	NA	NA	NA
NA	-3.060702337	-0.280926945	6.20E-05	0.027642777	NA	NA	NA	NA	NA
ENSG00000274349	-1.674030304	3.51361967	8.54E-07	0.001449725	ZNF658	9	66856426	66932141	1
NA	-2.512856109	1.545505763	7.57E-07	0.001427753	NA	NA	NA	NA	NA
NA	-3.507893738	-0.383015779	1.40E-06	0.001983576	NA	NA	NA	NA	NA
NA	3.850828439	-1.154214383	8.89E-05	0.034281652	NA	NA	NA	NA	NA
