# Supplementary material for: THC exposure of human iPSC neurons impacts genes associated with neuropsychiatric disorders
Source: Transl Psychiatry. 2018 Apr 25;8:89. doi: 10.1038/s41398-018-0137-3 (PMC5915454; doi:10.1038/s41398-018-0137-3)
Supplement: Supplementary file 11 — Supplementary Table 10 [file 41398_2018_137_MOESM11_ESM.pdf]

Supplementary Table 10: Pearson correlations show high consistency between our control and activated dataset (Batches 1 and 3) with that of Roussos et al (Batch 2).

	GM04506_KCl_Batch2	GM04506_Ctrl_Batch2	GM09319_KCl_Batch2	GM09319_Ctrl_Batch2	GM09429_KCl_Batch2	GM09429_Ctrl_Batch2
GM03651_Ctrl_Batch1	0.972	0.9772	0.9719	0.9776	0.9714	0.9717
GM03651_KCl_Batch1	0.9807	0.9749	0.9786	0.9761	0.9744	0.9724
GM03440_Ctrl_Batch3	0.9747	0.9746	0.9709	0.9734	0.9648	0.9681
GM3440_KCl_Batch3	0.9809	0.9741	0.978	0.9738	0.9717	0.9684
